# Clock genes and diurnal transcriptome dynamics in summer and winter in the gymnosperm Japanese cedar (*Cryptomeria japonica* (L.f.) D.Don)

**DOI:** 10.1186/s12870-014-0308-1

**Published:** 2014-11-18

**Authors:** Mine Nose, Atsushi Watanabe

**Affiliations:** Forest Tree Breeding Center, Forestry and Forest Products Research Institute, Ibaraki, 319-1301 Japan; Faculty of Agriculture, Kyushu University, Fukuoka, 812-8581 Japan

**Keywords:** Clock, Conifer, Diurnal rhythm, Gene network, Photoreceptor, Season, Transcriptome, Winter disruption

## Abstract

**Background:**

The circadian clock and diurnal dynamics of the transcriptome are presumed to play important roles in the regulation of physiological, biological and developmental processes synchronized with diurnal and annual cycles of plant environments. However, little is known about the circadian clock and its regulation in gymnosperms, including conifers. Here we present the diurnal transcriptome dynamics of Japanese cedar (*Cryptomeria japonica* (L.f.) D.Don) in both active (summer) and dormant (winter) periods.

**Results:**

Microarray analysis revealed significant differences in transcripts between summer and winter, and diurnal transcriptome dynamics only in the summer. About 7.7% of unique genes (556 out of 7,254) on the microarray were periodically expressed in summer. Expression patterns of some genes, especially light-related genes, did not show significant oscillation in Japanese cedar, thus differing from those reported in angiosperms. Gene network analysis of the microarray data revealed a network associated with the putative core clock genes (*CjLHYa*, *CjLHYb*, *CjTOC1*, *CjGI* and *CjZTL*), which were also isolated, indicating their importance in the diurnal regulation of the transcriptome.

**Conclusion:**

This study revealed the existence of core clock genes and diurnal rhythms of the transcriptome in summer in Japanese cedar. Dampening of diurnal rhythms in winter indicated seasonal change in the rhythms according to environmental conditions. The data also revealed genes that showed different expression patterns compared to angiosperms, suggesting a unique gene regulatory network in conifers. This study provides fundamental data to understand transcriptional regulatory mechanisms in conifers.

**Electronic supplementary material:**

The online version of this article (doi:10.1186/s12870-014-0308-1) contains supplementary material, which is available to authorized users.

## Background

In conifers, as in other plant species, many physiological and biological processes are synchronized with the day/night cycle of their environment, such as photosynthesis, shoot elongation, growth in height, and xylem pressure potential of saplings [1-4]. At the cellular level, daily dynamics of xylem cell radial growth, volumetric changes, and supply of cell wall components have been observed [5-8]. In addition, trees native to temperate and boreal regions show an annual active-dormant cycle, which affects aspects of physiology such as growth in height and photosynthetic capacity [3,9-14]. These diurnal and seasonal changes are considered important traits for survival and growth in environments that vary daily and throughout the year.

Transcriptome dynamics plays important roles for diurnal and seasonal adaptation in plants to synchronize them with environmental changes, and may be under clock control [15-17]. Signal transduction mechanisms due to changes in light are well studied in the model angiosperm *Arabidopsis thaliana*. Light signals are perceived and transduced via photoreceptor phytochromes and cryptochromes to the central oscillators of the clock, which consist of three interlocked feedback loops [18-21]. The first loop, called the central loop, consists of TOC1 (TIMING OF CAB EXPRESSION 1, also known as PRR1 or PSEUDO-RESPONSE REGULATOR 1), LHY (LATE ELONGATED HYPOCOTYL) and CCA1 (CIRCADIAN CLOCK ASSOCIATED 1). LHY and CCA1 proteins bind to a region in the *TOC1* promoter that is critical for its regulation by the clock [22], and TOC1 represses expression of *LHY* and *CCA1* [21,23]. The second loop, called the morning loop, consists of LHY, CCA1, PRR7 and PRR9. LHY and CCA1 induce expression of PRR7 and PRR9, while PRR7 and PRR9 repress expression of LHY and CCA1 [24,25]. The third loop, the evening loop, consists of GI (GIGANTEA), TOC1 and evening complex proteins LUX (LUX ARRHYTHMO), ELF3 and ELF4 (EARLY FLOWERING 3 and 4) [21]. Stability of GI and degradation of TOC1 are controlled by the blue light receptor ZTL (ZEITLUPE) [26-28], and the ZTL protein is stabilized by GI in blue light [29]. The activity of evening complex protein ELF3 is regulated by light through degradation by the ubiquitin E3 ligase COP1 (CONSTITUTIVE PHOTOMORPHOGENIC 1) [21]. The expression dynamics of some transcripts is under circadian clock control. Depending on the experiment and calculation method, 2 to 16% of genes have been reported as being circadian regulated in *Arabidopsis* [30-33]. Expression of photosynthesis genes peaks near the middle of the subjective day and phenylpropanoid biosynthesis genes peak before subjective dawn [30]. Genes encoding starch-mobilizing enzymes, genes implicated in cell elongation and genes related to hormone are also circadian-regulated [33,34].

Recently, homologues of *CCA1*, *GI*, *ZTL*, and *PRR1* were isolated from the conifer *Picea abies,* and analysis of ectopic expression of the four genes in *Arabidopsis* indicated that the protein functions of *PaCCA1*, *PaGI* and *PaZTL* are partly conserved [35]. This suggested the existence of the three-loop network in coniferous species as well. However, Gyllenstrand *et al.* reported that cycling of clock genes of *P. abies* is rapidly dampened in free-running conditions, in contrast to observations of clock gene expression in most other plant species [36]. Since angiosperms and gymnosperms are considered to have separated evolutionarily 300 million years ago [37], it would not be surprising if conifers had different control mechanisms. The clock and its relationship to diurnal dynamics of the transcriptome are still largely unknown in conifers. Also, differences in diurnal transcriptome dynamics between periods of growth and dormancy have not been extensively investigated, although such differences may play an important role in perennial plants.

Japanese cedar (*Cryptomeria japonica* (L.f.) D.Don) is a major forestry species in Japan. Studying the diurnal and seasonal regulation of its transcriptome is fundamental to understand environmental adaptation mechanisms, and unavoidable to advance research into important characteristics controlled by diurnal and seasonal rhythms, such as wood formation, growth in height, and flowering. Moreover, studying Japanese cedar is interesting from the view of evolution of the clock, since *Cryptomeria* is a gymnosperm and is an evolutionarily old conifer genus with fossils dating back to the Cretaceous period [38]. In this study, we focused on diurnal transcriptome dynamics in summer (Jul) and winter (Dec). We first collected sequence data for genes expressed in shoots to design a microarray for Japanese cedar using three different methods (Additional file 1): Two suppression subtractive hybridization (SSH) libraries and one normalized complementary DNA (cDNA) library were created to obtain sequence data for genes expressed especially in the daytime and nighttime in summer. Next-generation sequencing (NGS) was performed to obtain exhaustive sequence data on genes expressed throughout the day and year. Microarray analysis identified diurnal transcriptome dynamics in summer, when tree growth is greatest, while dynamic changes were not detected in winter, when trees went dormant. Gene network analysis of the microarray data revealed new insights into temporal regulation of transcripts in conifers, including clock genes that might influence diurnal transcriptome dynamics. Moreover, we isolated putative homologues of the core clock (*LHY*, *CCA1*, *TOC1*, *GI* and *ZTL*) and photoreceptor genes, and identified their expression patterns and the position of Japanese cedar within the phylogenetic tree of the plant kingdom. This study provided fundamental gene expression data that will help to understand molecular mechanisms of diurnal and seasonal adaptation in conifers.

## Results

### Collecting sequence data from Japanese cedar shoots and designing a microarray

Two SSH libraries and one normalized cDNA library were constructed to obtain gene sequences expressed specifically during the day and night in summer (Additional file 1). A forward library (SSH12) containing genes expressed predominantly at midday was constructed by subtracting driver RNA isolated from shoots at midnight from tester RNA isolated from shoots at midday. A reverse library (SSH24) containing genes expressed predominantly at midnight was constructed by subtracting driver RNA isolated from shoots at midday from tester RNA isolated from shoots at midnight. SSH12 and SSH24 respectively consisted of 595 and 594 expressed sequence tags (ESTs) varying in length from 89 to 799 bp with an average length of 488 bp. These ESTs were assembled into 969 sequences, with 33 contigs sharing ESTs from both libraries. However, we found no significantly upregulated genes at either midday or midnight. The BLASTX algorithm was used to search for the top hits of each sequence in the *Arabidopsis* protein database with an e-value cutoff of e-10, leading to 325 annotated EST sequences from SSH12 and 354 from SSH24 that were categorized by GO annotation (Additional file 2A). The normalized cDNA library was constructed from an RNA mixture extracted from shoots collected at midday and midnight to obtain gene sequences expressed extensively in the daytime and nighttime in the summer (Additional file 1). We obtained 2,653 cDNA sequences varying in length from 149 to 828 bp with an average length of 655 bp. The 2,653 cDNA sequences were assembled into 2,333 sequences including 264 contigs. GO categorization was carried out using the 2,133 annotated sequences from the 2,653 sequences (Additional file 2B).

NGS was carried out on an RNA mixture isolated from shoots of diurnal and seasonal series of samples to obtain sequences of genes expressed throughout the day and year (Additional file 1). We obtained 116 Mbp of sequencing data in the form of 273,104 reads averaging 426 bp in length that passed the quality filter of GS RunProcessor. Adapter sequences were trimmed, and reads shorter than 50 bp were removed from the sequence data. Subsequently, the reads that matched *Arabidopsis* retrotransposons and simple sequence repeats (SSRs) of Japanese cedar registered in the Sugi Genome Database were excluded from the NGS data with the aim of removing unnecessary sequences prior to assembly. The frequency distribution of 111 Mbp of 265,962 reads is illustrated in Additional file 3A. These reads were entered as assemblies run in the GS De Novo Assembler, and 265,962 reads were placed into 7,613 contigs (over 100 bp) and 45,112 singletons. Further assembly was performed to predict putative transcript sequences, and the 7,613 contigs were placed into 6,890 isotigs. The frequency distribution of isotigs is illustrated in Additional file 3B. Gene descriptions of isotigs and singletons were predicted by BLASTX, and the GO categorization of 10,275 targets from NGS that hit unique *Arabidopsis* gene IDs with an e-value cutoff of e-10 is provided in Additional file 2C.

Microarray probes were designed based on sequences from the SSH and cDNA libraries and the NGS isotigs. NGS singletons (length >400 bp) that showed high homology to any *Arabidopsis* gene with an e-value threshold of e-40, and singletons with hits to *Arabidopsis* genes related to circadian rhythms, photosynthesis, or hormones listed in the KEGG pathway (the Kyoto Encyclopedia of Genes and Genomes, http://www.genome.jp/kegg/pathway.html) without any e-value cutoff were preferentially selected as probe candidates. Identical sequences (sequence identity >95%, overlap >90%) were eliminated from the proven candidates, and finally, a microarray consisting of four probe sets corresponding to 15,728 sequences (targets) was designed. A summary of the original libraries containing the 15,728 sequences is in Additional file 1.

### General overview of transcriptome

**Figure 1:**
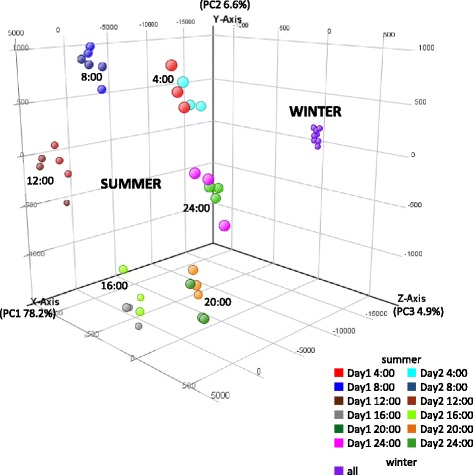
**Principal component analysis of microarray data.** The plot illustrates the principal components of all 36 summer samples and 8 of the winter samples.

Shoot samples were collected every four hours from 4:00 for two days (12 time points) in summer (Jul 30 and 31). We collected samples from three cuttings at each time point as biological replicates. All 36 summer samples were analyzed using a microarray and grouped into 12 categories according to their sampling time. Also, 8 selected winter samples (4:00/8:00/12:00/16:00/20:00/24:00 on Dec 22, and 12:00/24:00 on Dec 23 with no replicates) were analyzed by the microarray. Since no targets showed any significant differences between 12:00 and 24:00, we estimated that very small or no periodic changes in expression occurred in winter, and all data for winter samples were grouped together. The 13 total groups (12 summer groups and 1 winter group) were compared in all possible combinations, and 14,342 targets, corresponding to 6,838 unique genes, were observed to be significantly differentially expressed in one or more groups. Principal component analysis (PCA) of the 6,838 unique genes demonstrated that transcriptome differences between summer and winter were represented by principal component 1 (PC1, 78.2%), and diurnal transcriptional changes in the summer by PC2 (6.6%) and PC3 (4.9%, Figure 1).

### Identification and clustering of cycling genes in summer

**Figure 2:**
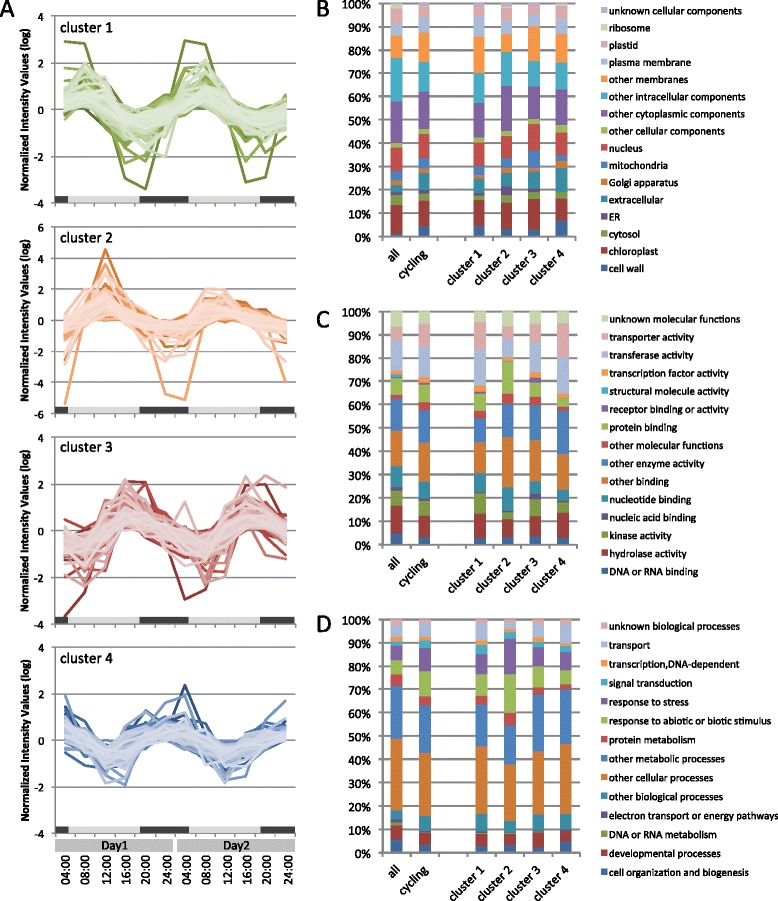
**Clustering and gene ontology (GO) annotation of the cycling genes in summer.** The 556 cycling genes in summer were classified into four clusters (clusters 1 to 4) by their expression patterns in microarray data **(A)**, and categorized by GO annotation into the major functional categories of cellular component **(B)**, molecular function **(C)** and biological process **(D)**. Each cluster corresponds to a gene group derived from **(A)**. Gray and black bars below graph **(A)** respectively represent natural length of day and night (measured between sunrise and sunset) reported by the National Astronomical Observatory of Japan. ‘all’ indicates all genes on the microarray and ‘cycling’ indicates all 556 cycling genes.

Statistical analysis by the GeneCycle package [39] indicated that 999 targets on the microarray were periodically expressed over a 1-day cycle with a two-fold difference in summer (Additional file 4). Of the 999 targets, 817 targets corresponding to 556 unique genes (7.7% of unique genes in microarray) were annotated by BLASTX analysis to *Arabidopsis* proteins, while the other 182 targets were not. According to the ranking of fold changes in peak-to-trough amplitude, targets of core clock genes (*LHY*, *PRR7* and *GI*) were within the upper 10 (Additional file 4). Putative genes for heat shock proteins, chlorophyll *a/b* binding family proteins (ELIP1 and ELIP2), dentin sialophosphoprotein-related protein, cycling CDF factor 2 (CDF2) and B-box type zinc finger family protein also showed large oscillations with more than 15-fold changes. There were 27 unannotated targets within the upper 100. GO analysis indicated that the 556 cycling genes had more than a two-fold higher percentage of genes with functions in the ‘cell wall’ (4.3%) and ‘extracellular’ (7.2%) cellular component categories than the entire set of genes on the microarray (Figure 2B). The 556 cycling genes were classified into four clusters based on similarity of their expression patterns, and each cluster consisted of genes that showed peak expression in the morning (cluster 1), at noon (cluster 2), in the evening (cluster 3) and at night (cluster 4) (Figure 2A, Additional file 5). Comparing the clusters in the cellular component category (Figure 2B), cluster 4 contained a higher proportion of transcripts related to ‘cell wall’ (7.0%), with the other clusters containing 3.1 to 4.4%. Cluster 3 contained a higher proportion of genes functioning in the ‘ER’ (3.6%), while the other clusters contained up to 1.4%. Cluster 3 contained more than a three-fold higher proportion of genes functioning in the ‘mitochondria’ (7.6%) compared with cluster 4 (2.4%). In the molecular function category (Figure 2C), clusters 1 and 4 contained approximately two-fold more genes related to ‘transporter activity’ (11.6% and 14.5% respectively) than cluster 2 (5.3%), and cluster 2 contained approximately four-fold more genes in the ‘protein binding’ (13.9%) category than cluster 4 (3.3%). In the biological process category (Figure 2D), cluster 2 contained more genes with functions in ‘response to abiotic or biotic stimulus’ (16.5%) and ‘response to stress’ (15.1%), and fewer genes related to ‘transport’ (2.8%) than the other clusters.

### Summer gene network

**Figure 3:**
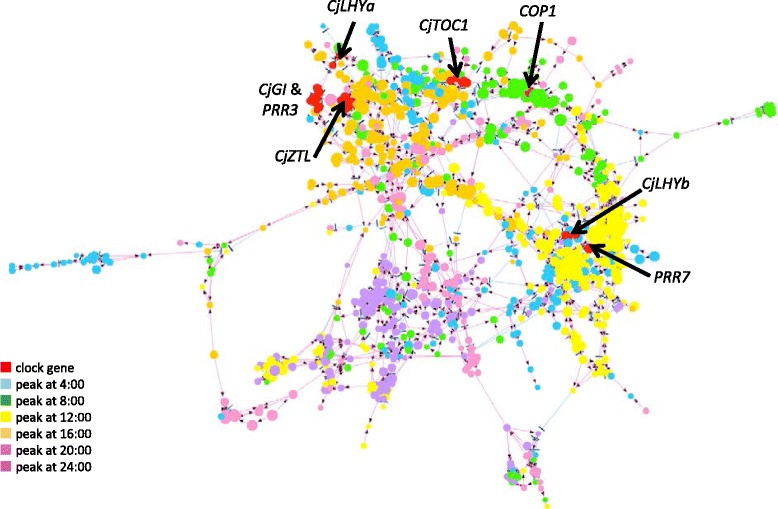
**Estimated gene network including core clock genes.** The gene network was estimated by the SiGN-BN program from the 1,000 targets with the highest coefficient of variation, and 886 targets connected to the edge with bootstrap probability higher than 0.7 are illustrated here. Red nodes indicate core clock genes, and the other colors indicate the peak time of expression of the target. Since some genes were analyzed by several microarray probes, there were several red nodes for clock genes.

Gene network analysis was carried out using the 1,000 targets with the highest coefficient of variation in the normalized datasets of 36 summer samples (Additional file 6). We found that all of the 1,000 targets constituted one gene network. Targets with a large number of children may be core genes for transcriptional regulation. The target with the top BLASTX hit to a chaperone DnaJ-domain superfamily protein had the largest number of children (128 targets), followed by a target that hit a DNAJ heat-shock N-terminal domain-containing protein (123 targets, Additional file 6). Another 50 targets, such as putative genes for deoxyxylulose-5-phosphate synthase (CLA1), maternal effect embryo arrest 14 (MEE14), sigma factor E (SIGE), pyruvate phosphate dikinase (SEX1), cytochrome P450 family member (CYP76C3) and CDF2 also had more than 50 children (Additional file 6). We extracted 2,604 edges that showed bootstrap probability higher than 0.7 and 886 related targets corresponding to 447 unique genes from the entire gene network for more reliable data (Figure 3). The network file is available from Additional file 7. We focused on the clock genes that are components of the new conceptual framework for the *Arabidopsis* clock provided by Pokhilko *et al.* [21]. The five genes isolated (*CjLHYa*, *CjLHYb*, *CjTOC1*, *CjGI* and *CjZTL*) and putative *PRR3*, *PRR7* and *COP1* genes (e-values 9e-42, 7e-82 and 3e-75, respectively) were included in this extracted gene network. Although *PRR3* was not considered a member of the *Arabidopsis* clock framework by Pokhilko *et al.*, we included *PRR3* in Japanese cedar, since the function of the PRR family is still unknown in conifers. In the estimated network, the four clock genes (*CjLHYa*, *CjGI, CjZTL* and putative *PRR3*) were located close together in the gene network. *CjLHYa* and putative *PRR3* were direct child genes of *CjGI* with bootstrap probabilities of 0.739 and 0.942, respectively. *CjZTL* was a child of the gene encoding a DNA/RNA polymerase superfamily protein (HI9HAF202CL26P, e-value 3e-44), which was a child of *CjGI*. The two clock genes, *CjLHYb* and putative *PRR7*, were both children of *EXORDIUM LIKE 3* (isotig03899, e-value 7e-82) and a gene for chaperone DnaJ-domain superfamily protein (isotig00872, e-value 4e-24). *CjLHYb* was a child of the unannotated target SSH24-3-25_002_A04, which was a child of *PRR7*.

### Transcriptome differences between summer and winter

**Figure 4:**
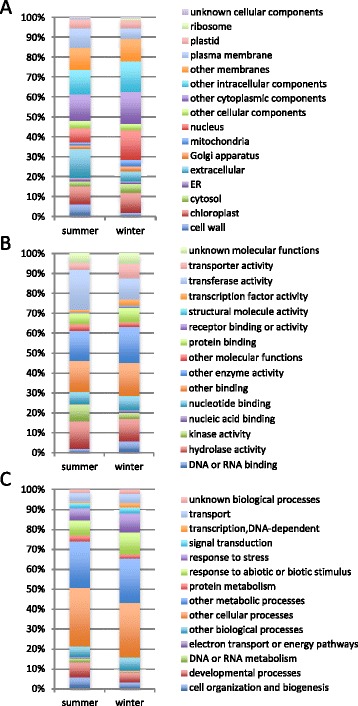
**Gene ontology (GO) categorization of genes differentially expressed in summer and winter.** Genes showing more than a four-fold difference in expression between summer and winter are categorized by GO annotation of major functional categories: **(A)** cellular component, **(B)** molecular function and **(C)** biological process.

By comparing microarray data from summer and winter regardless of sampling time, 13,318 targets showed significant differences in expression level. Of these, 1,329 targets corresponding to 759 unique genes showed more than a four-fold difference, consisting of 475 genes upregulated in summer and 284 in winter. The top 100 differentially expressed targets are listed in Additional file 8. Putative genes for tetraspanin8 (TET8), glucose-methanol-choline oxidoreductase family protein, expansin A8 (EXPA8) and peroxidase superfamily protein (RCI3) were upregulated more than 200-fold in summer, while putative genes for BURP domain-containing protein (RD22) and ELIP1 were upregulated in winter. GO categorization indicated that the proportion of genes associated with ‘extracellular’ (14.5%) and ‘cell wall’ (6.0%) in the cellular component category (Figure 4A), with ‘kinase activity’ (8.7%) in the molecular function category (Figure 4B), and with ‘DNA or RNA metabolism’ (2.1%) in the biological process category (Figure 4C) was more than two-fold larger in summer. On the other hand, ‘nucleus’ (14.9%) and ‘mitochondria’ (3.4%) in the cellular component category (Figure 4A), ‘transporter activity’ (7.3%), ‘DNA or RNA binding’ (5.8%) and ‘transcription factor activity’ (2.9%) in the molecular function category (Figure 4B), and ‘transcription, DNA-dependent’ (2.1%) in the biological process category accounted for more than a two-fold larger proportion in winter (Figure 4C).

### Identification of putative photoreceptor and clock-related genes from Japanese cedar

**Figure 5:**
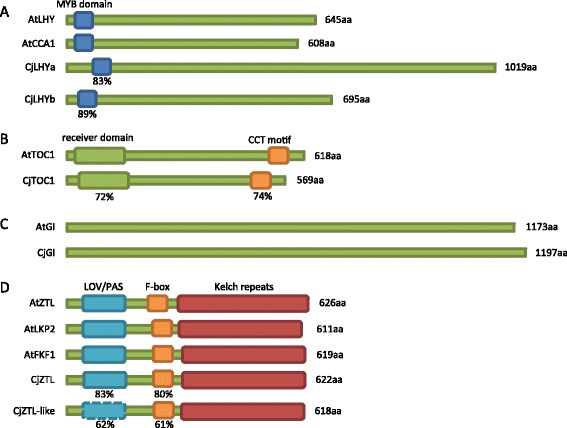
**Domain structure of LHY and CCA1 (A), TOC1 (B), GI (C), and ZTL, LKP2 and FKF1 (D) in** ***Arabidopsis thaliana*** **(At) and** ***Cryptomeria japonica*** **(Cj).** The amino acid similarity of each domain is presented as a percentage. CjLHYa and CjLHYb were both compared to AtLHY, and CjZTL and CjZTL-like were compared to AtZTL. No LOV/PAS domain was detected in CjZTL-like (broken line) by a Pfam search. The NCBI accession numbers of the *Arabidopsis* proteins are NP_001030924 (AtLHY), NP_850460 (AtCCA1), NP_200946 (AtTOC1), NP_564180 (AtGI), NP_001154783 (AtZTL), AEC06826 (AtLKP2) and AAF32298 (AtFKF1).

**Figure 6:**
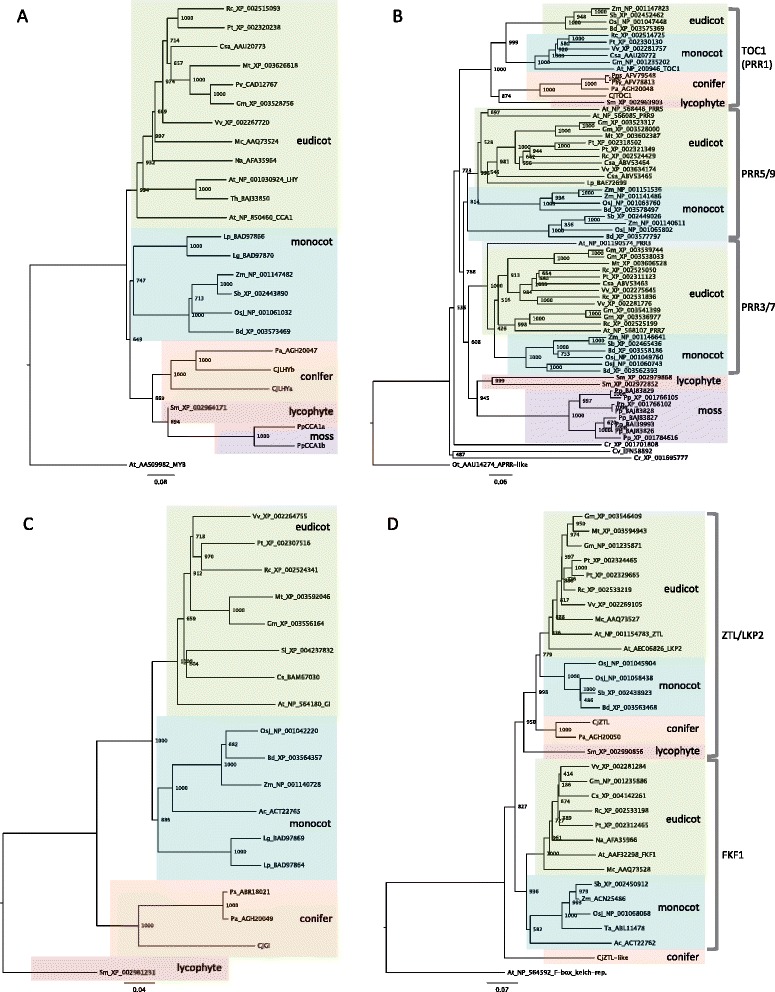
**Phylogenetic analysis of LHY and CCA1 (A), PRR family (B), GI (C), and ZTL, LKP2 and FKF1 (D) in plants.** The neighbor-joining method [77] was used to construct the phylogenetic trees. The names of genes isolated from Japanese cedar (*Cryptomeria japonica*) start with Cj. Other species names are abbreviated as follows: Ac, *Allium cepa* (onion); At, *Arabidopsis thaliana* (thale cress); Bd, *Brachypodium distachyon* (purple false brome); Cr, *Chlamydomonas reinhardtii* (green alga); Cs, *Chrysanthemum seticuspe* f. *boreale* (chrysanthemum); Csa, *Castanea sativa* (chestnut); Cv, *Chlamydomonas variabilis* (green alga); Gm, *Glycine max* (soybean); Lg, *Lemna gibba* (gibbous duckweed); Lp, *Lemna paucicostata* (duckweed); Mc, *Mesembryanthemum crystallinum* (common iceplant); Mt, *Medicago truncatula* (barrel medic); Na, *Nicotiana attenuata* (coyote tobacco); Ot, *Ostreococcus tauri* (picoplankton); Osj, *Oryza sativa* (Japanese rice); Pa, *Picea abies* (Norway spruce); Pp, *Physcomitrella patens* subsp. *patens* (moss); Pps, *Pinus pinaster* (maritime pine); Ps, *Picea sitchensis* (Sitka spruce); Psy, *Pinus sylvestris* (Scots pine); Pt, *Populus trichocarpa* (black cottonwood); Pv, *Phaseolus vulgaris* (common bean); Rc, *Ricinus communis* (castor bean); Sb, *Sorghum bicolor* (sorghum); Sl, *Solanum lycopersicum* (tomato); Sm, *Selaginella moellendorffii* (lycophyte); Ta, *Triticum aestivum* (bread wheat); Th, *Thellungiella halophila* (salt cress); Vv, *Vitis vinifera* (wine grape); Zm, *Zea mays* (maize). The number following the species name indicates its NCBI accession number. The amino acid sequences of PpCCA1a and PpCCA1b are from Okada *et al.* [41]. *Arabidopsis* MYB protein (AAS09982), *O. tauri* APRR-like protein (AAU14274), *S. moellendorffii* GI protein (XP_002961231) and *Arabidopsis* F-box kelch-repeat protein (NP_564592) were used as the outgroups of each phylogenetic tree.

We isolated six homologues of clock genes from Japanese cedar. The two homologues of *LHY* and *CCA1* were named *CjLHYa* and *CjLHYb* [DNA Data Bank of Japan (DDBJ): AB894539 and AB894540]. They showed high homology in a single myb domain [40] with *Arabidopsis* LHY at the amino acid level (83% and 89%, respectively, Figure 5A) and two homologues in the moss *Physcomitrella patens*, PpCCA1a and PpCCA1b (89% to 100%, Additional file 9A) [41]. However, the amino acid sequences of the other regions were highly divergent. We constructed a phylogenetic tree using amino acid sequences for LHY and CCA1 homologues from plants (Figure 6A). Genes from seed plants divided into the three clusters of eudicots, monocots and conifers, and CjLHYa and CjLHYb are positioned within the coniferous cluster. A homologue of *Arabidopsis* TOC1, a member of the PRRs, was identified in Japanese cedar and named CjTOC1 (e-value 6e-77) [DDBJ: AB894541]. The amino acid sequence identity of a receiver domain and a CONSTANS/CONSTANS-LIKE/TOC1 (CCT) motif [42] were 72 and 74%, respectively (Figure 5B, Additional file 9B). A phylogenetic tree of PRRs from plant species showed three clusters consisting of homologues of PRR1, PRR3/PRR7 and PRR5/PRR9 (Figure 6B). CjTOC1 belongs to the PRR1 cluster with homologues of other conifers, *P. abies*, *Pinus sylvestris* and *Pinus pinaster.* The amino acid sequence of CjGI isolated from Japanese cedar revealed high sequence homology with GI of *Arabidopsis* and a lycophyte (*Selaginella moellendorffii*) GI, with an e-value of 0.0 [DDBJ: AB894538] (Additional file 9C). A phylogenetic tree of GI showed three clusters consisting of homologues of monocots, eudicots and conifers (Figure 6C). The isolated CjGI belongs to a conifer cluster with homologues from *P. abies* and *Picea sitchensis*. CjZTL and CjZTL-like showed high amino acid sequence similarity to *Arabidopsis* ZTL, both having an e-value of 0.0 [DDBJ: AB894543 and AB894542] (Additional file 9D). The homology of a LOV/PAS domain and an F-box domain [43] was 83% and 80% respectively for CjZTL, and 62% and 61% for CjZTL-like with respect to *Arabidopsis* ZTL (Figure 5D). Six kelch repeat sequences were also detected from both CjZTL and CjZTL-like by a domain search using the Pfam database with a threshold e-value of e-10. We constructed a phylogenetic tree with the other blue light receptors, LKP2 (LOV KELCH PROTEIN 2) and FKF1 (FLAVIN BINDING, KELCH REPEAT, F-BOX). The plant ZTL/LKP2/FKF1 genes were classified into two groups, ZTL/LKP2 and FKF1 (Figure 6D). CjZTL belonged to the ZTL/LKP2 group and CjZTL-like was isolated from both groups.

**Figure 7:**
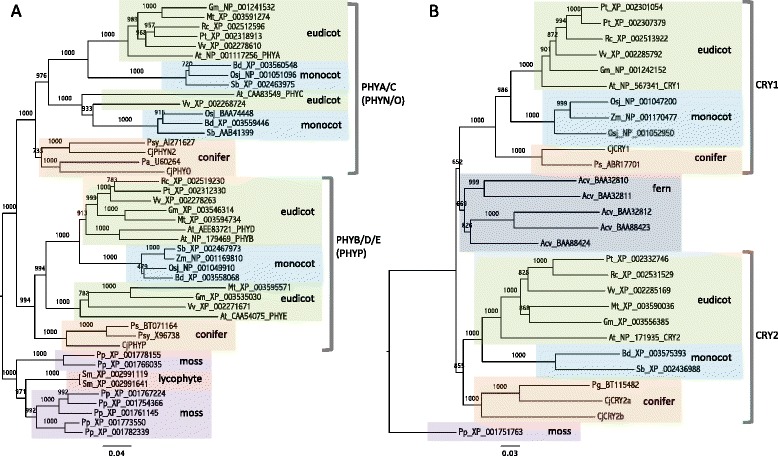
**Phylogenetic analysis of photoreceptor phytochrome (A) and cryptochrome (B) genes in plants.** The neighbor-joining method [77] was used to construct the phylogenetic trees. The names of genes isolated from Japanese cedar (*Cryptomeria japonica*) start with Cj. Other species names are abbreviated as follows: Acv, *Adiantum capillus-veneris* (fern); At, *Arabidopsis thaliana* (thale cress); Bd, *Brachypodium distachyon* (purple false brome); Gm, *Glycine max* (soybean); Mt, *Medicago truncatula* (barrel medic); Osj, *Oryza sativa* (Japanese rice); Pa, *Picea abies* (Norway spruce); Pg, *Picea glauca* (white spruce); Pp, *Physcomitrella patens* subsp. *patens* (moss); Ps, *Picea sitchensis* (Sitka spruce); Psy, *Pinus sylvestris* (Scots pine); Pt, *Populus trichocarpa* (black cottonwood); Rc, *Ricinus communis* (castor bean); Sb, *Sorghum bicolor* (sorghum); Sm, *Selaginella moellendorffii* (lycophyte); Vv, *Vitis vinifera* (wine grape); Zm, *Zea mays* (maize). The number following the species name indicates its NCBI accession number. Trees were rooted with phytochrome and cryptochrome of the moss and lycophyte.

Full-length sequences of three phytochrome genes [DDBJ: AB894547 (*CjPHYN*_*2*_), AB894548 (*CjPHYO*) and AB894549 (*CjPHYP*)] and three cryptochrome genes [DDBJ: AB894544 (*CjCRY1*), AB894545 (*CjCRY2a*) and AB894546 (*CjCRY2b*)] were isolated from Japanese cedar. All three showed high homology to *Arabidopsis* phytochromes (e-value 0.0). CjCRY1 was highly homologous to *Arabidopsis* CRY1 (e-value 0.0), and CjCRY2a and CjCRY2b were highly homologous to *Arabidopsis* CRY2 (e-values of e-176 and 0.0, respectively) at the amino acid level. A phylogenetic tree using amino acid sequences of plant phytochromes indicated that after seed plants diverged from mosses and lycophytes, genes from seed plants clustered into two groups consisting of PHYA/C and PHYB/D/E (Figure 7A). CjPHYN_2_ and CjPHYO belong to the PHYA/C cluster, and CjPHYP belongs to the PHYB/D/E cluster. A phylogenetic tree of cryptochromes indicated that genes from seed plants diverged into two clusters, CRY1 and CRY2, and cryptochromes of ferns created a unique cluster (Figure 7B). The cluster of CRY1 and CRY2 of seed plants diverged into three groups consisting of eudicots, monocots and conifers. CjCRY1 was classified into the CRY1 cluster, and CjCRY2a and CjCRY2b were classified into the CRY2 cluster.

### Diurnal rhythms in transcription of clock-related genes

**Figure 8:**
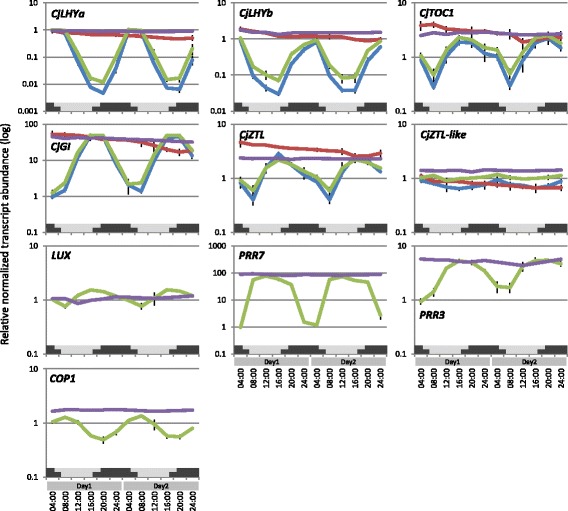
**Diurnal changes in relative transcript abundance in summer (Jul 30–31, 2012) and winter (Dec 22–23, 2011) analyzed by microarray and qPCR.** The microarray data of summer samples (green line) and qPCR data of summer (blue line) and winter (red line) samples represent the means from three biological replicates at 12 time points. The microarray data of winter samples (purple line) are from 8 selected time points (4:00/8:00/12:00/16:00/20:00/24:00 on day 1 and 12:00/24:00 on day 2) without a biological replication. The data obtained for each time point were compared with the data obtained for shoots collected at 4:00 on Jul 30, 2012. Gray and black bars below graph respectively represent natural length of day and night (measured between sunrise and sunset) in summer (upper) and winter (lower), provided by the National Astronomical Observatory of Japan. The genes isolated in this study have the prefix ‘Cj’ in their names.

We analyzed expression patterns of 12 transcripts of putative clock-related and photoreceptor genes isolated in this study by quantitative PCR (qPCR) to estimate the reliability of microarray data. Very similar results (up- or downregulation) were obtained for the transcripts using both techniques for expression analysis (Figure 8, Additional file 10), suggesting that the data obtained in this study are reliable. The microarray and qPCR data revealed significant oscillations in expression of *CjLHYa*, *CjLHYb*, *CjTOC1*, *CjGI* and *CjZTL* in summer, except for *CjZTL-like* (Figure 8). The level of transcription of putative *LUX* (e-value 3e-42), an evening complex protein [21], reached a peak at 16:00 (Figure 8). Putative PRR member genes *PRR3* and *PRR7* also showed diurnal expression patterns. The transcriptional level of putative *PRR7* remained at the maximum value from 8:00 to 20:00, and that of *PRR3* reached a peak at 16:00 and subsequently declined (Figure 8). Transcriptional levels of putative *COP1* reached a peak at 8:00 (Figure 8). In the winter, transcriptional levels of the core clock genes did not oscillate (Figure 8). The expression levels of *CjLHYa*, *CjLHYb*, *CjTOC1*, *CjGI*, *CjZTL*, *PRR7*, *PRR3* and *COP1* in winter were similar to their maximum expression level in the summer. Among the six photoreceptor genes isolated, only *CjPHYP* and *CjCRY1* showed diurnal oscillations of small amplitude that peaked at 4:00 (Additional file 10). By comparing the transcriptional levels between summer and winter regardless of sampling time, we observed more than a four-fold increase in *CjCRY2a* expression in winter.

## Discussion

The existence of diurnal transcriptome dynamics in summer was clearly demonstrated by PCA of microarray data (Figure 1). About 7.7% of unique genes (556 out of 7,254) showed diurnal rhythms with more than two-fold changes in peak-to-trough amplitude (Additional file 5). Although different calculation programs were used to detect cycling genes, almost the same proportion of cycling genes (217 out of 2,608, or 8%) in *Eucalyptus* planted in the field in early spring has been reported [44]. In *Populus* trees, 18% of genes on a microarray exhibited a diurnally influenced expression pattern [45]. On the other hand, 182 targets that showed significant oscillation with more than a two-fold difference in diurnal amplitude in Japanese cedar had no BLASTX hits against *Arabidopsis* proteins (Additional file 4). These targets might include genes specific to conifers that take part in unique regulation of diurnal rhythms.

We classified the 556 cycling genes into four clusters based on their expression pattern, and each cluster showed a different proportion of GO categories (Figure 2). This may be an indication of the relationship between diurnal transcriptome dynamics and diurnal changes in physiological and biological conditions. The rate of growth in height of Japanese cedar began to increase after midday, reached a peak around dawn, and subsequently decreased (Additional file 11C). This diurnal rhythm in the growing pattern of Japanese cedar matched that reported by Gyokusen [4], and was consistent with reported volumetric changes in differentiating cells [7]. Hosoo *et al.* reported that diurnal periodicity in the supply of cell wall components to developing second walls is associated with changes in light intensity during the photoperiodic cycle [7]. Our microarray data demonstrated that genes related to cell wall components account for a disproportionately large percentage of cycling genes (4.3%) relative to all genes on the microarray (1.2%), and the proportion increased during the nighttime (Figure 2B). Three genes putatively encoding expansins, which are linked to cell enlargement and cell wall changes, oscillated and reached peak expression at noon in this study (cluster 2, Additional file 5). Putative genes encoding xyloglucan endotransglucosylase/hydrolase, which also plays important roles in cell growth, also oscillated, but the peak times of expression varied (Additional file 5). In *Arabidopsis*, hormone-related genes believed to be responsible for hormone biosynthesis and signal transduction are co-expressed at the time of day when the hypocotyl growth rate is maximal, in the morning under short-day conditions [46]. To determine whether the same phenomenon exists in Japanese cedar, we analyzed the expression patterns of the hormone-related genes that represent hormone biosynthesis and signaling pathways: the phytohormone genes listed by Michael et al. [44], the genes involved in hormone signaling listed in the KEGG database, and the genes of the hormone synthetic pathway listed in the RIKEN Plant Hormone Research Network (http://hormones.psc.riken.jp). Of the 556 cycling genes, 22 genes that may be related to growth hormones (auxins, gibberellins, cytokinins and brassinosteroids) showed diurnal rhythms (Additional file 5). Of these 22 genes, 16 belonged to cluster 1 or cluster 2 (Figure 2A, Additional file 5). This result indicates that most growth hormone-related genes show expression peaks from morning to noon, although the growth rate of Japanese cedar is maximal in the evening, unlike *Arabidopsis* (Additional file 11C) [4,7,46].

Many genes categorized under ‘response to stress’ and ‘response to abiotic and biotic stimulus’ based on GO annotation had peak expression at noon (Figure 2D cluster 2). Since summer temperatures during the experimental period reached nearly 30°C and the photosynthetically active radiation exceeded 2000 μmol/m^2^/sec at midday (Additional files 11A and B), the trees might have been subjected to stress. More than 20 putative genes for heat shock proteins, which function as molecular chaperones to help to cope with heat stress, showed cyclical transcriptional levels and most had peak expression at noon (Figure 2A cluster 2, Additional file 5). The plant hormones abscisic acid (ABA) and ethylene are believed to be important components in the crosstalk between stress signaling pathways. In Japanese cedar, 11 putative ABA-related genes were diurnally regulated, including putative homologues of genes that are also induced by drought stress in *Arabidopsis* (*CYP707A3*, *CYP707A4*, *NCED3*) [47-49], and 6 of them reached peak expression during the day (Figure 2A cluster 2, Additional file 5). Putative genes for ethylene receptors ERS1 (ethylene response sensor 1) and ETR1 (ethylene sensor) had peak expression at morning (Figure 2A cluster 1, Additional file 5). Also, expression of *ERF6* (*ethylene responsive element binding factor 6*), which is a central regulator of leaf growth under water limiting conditions in *Arabidopsis* [50], was upregulated at evening (Figure 2A cluster 3, Additional file 5). Covington *et al.* investigated circadian microarray data, revealing that plant hormone and multiple stress response pathways are influenced by the circadian clock [34]. More than 40% of ABA-induced genes are circadian-regulated in *Arabidopsis*, and the majority of these genes reach peak transcriptional levels during the subjective morning in *Arabidopsis*. Also, of the genes induced by oxidative stress or reactive oxygen species production under various stress conditions, such as bright light, drought, or extreme temperature, 34% are clock-regulated [34]. It is difficult to know from this study alone whether the expression of these stress-related genes in Japanese cedar is controlled by the integration of environmental cues or the circadian clock. However, Japanese cedar regulated transcripts of stress-related genes, which might have helped to adjust to the severe daytime environment.

The genes we observed showing variation in expression during two consecutive days in the summer were part of one big network, indicating that their expression was closely coordinated. For more reliable results, we extracted edges of the network that showed more than 0.7 bootstrap probability and their related targets from the entire gene network (Figure 3, Additional file 7). Although further studies are necessary to evaluate this network, this one gives many hints for selecting genes that play an important role in the regulation of diurnal transcriptome dynamics in conifers. The presence of putative core clock genes in this extracted network shows their importance in the diurnal regulation of the transcriptome. The five genes isolated (*CjLHYa*, *CjLHYb*, *CjTOC1*, *CjGI* and *CjZTL*) that were predicted homologues of *Arabidopsis* core clock genes because of their position within phylogenetic trees and similarity in their domains (Figures 5 and 6) were included in the extracted network. Each homologue of Japanese cedar constituted a coniferous cluster with that of *P. abies* reported by Karlgren *et al.* [35]. Expression patterns (up- or downregulation in morning or evening) of *CjLHYa*, *CjLHYb*, *CjTOC1* and *CjGI* (Figure 8) were similar to those in *Arabidopsis* under long-day conditions, according to the diurnal database on the website of the Mockler lab, Donald Danforth Plant Science Center (http://diurnal.mocklerlab.org). The two homologous genes, *CjLHYa* and *CjLHYb*, had high homology in their amino acid sequences; however, their transcriptional expression patterns were slightly different. While transcripts of *CjLHYa* reached a peak from 4:00 to 8:00, *CjLHYb* reached a peak at 4:00 and began to decline at 8:00 (Figure 8), and while transcriptional levels of *CjLHYa* showed more than a 100-fold difference between the maximum and minimum, that of *CjLHYb* showed only a 10-fold difference. Gene network analysis indicated that these two homologous genes are located at different positions (Figure 3), and suggested that they might have different roles in the network and regulate different genes at different times. The blue light receptor gene *ZTL* is constitutively expressed but displays circadian fluctuation at the protein level in *Arabidopsis* [51]. Also, *PaZTL* in *P. abies* does not oscillate under light/dark cycles [36]. However, we observed that *CjZTL* transcripts oscillate in Japanese cedar (Figure 8), as also reported for *EtZTL* in *Eucalyptus* in field conditions and for *McZTL* in iceplant (*Mesembryanthemum crystallinum*) under light/dark cycles and free-running conditions [44,52]. The other blue light receptor, *FKF1*, is clock regulated at the transcript level, while *LKP2* is not in *Arabidopsis* [53,54]. Since the expression pattern (oscillating or not) of these genes showed no phylogenetic relationship (Figure 6D), differences in their expression might have resulted from independent events occurring during evolution. Alternatively, the differences in expression may be caused by differences in environmental or developmental conditions.

Interestingly, most genes for photoreceptors and photosynthesis-related genes did not show any significant oscillation in Japanese cedar (Additional file 10), although light is one of the most important factors that influence diurnal rhythms and most such genes show diurnal or circadian expression patterns in angiosperms. In *Arabidopsis*, photoreceptor phytochromes and cryptochromes are involved in setting the clock by transducing the light signal to the core clock, and all photoreceptor genes are regulated by the clock [55]. The sequences of *CjPHYO* and *CjPHYP* had high homology to the partial sequences of *phyO* (AJ286622) and *phyP* (AJ286623) of Japanese cedar reported by Schmidt and Schneider-Poetsch [56]. However, the sequence of *CjPHYN*_*2*_ did not match *phyN*_*1*_ (AJ286624), as also reported by Schmidt and Schneider-Poetsch [56]. Since independent duplications have led to two copies of *PHYN* in conifers except for the *Pinaceae* [57], *CjPHYN*_*2*_ might be another copy of *PHYN*. Expression analysis indicated that of the three phytochrome genes, only *CjPHYP* oscillated with small amplitude (Additional file 10). Moreover, among the three isolated cryptochrome genes, only *CjCRY1* oscillated with small amplitude. The oscillating photoreceptors *CjPHYP* and *CjCRY* were genetically distinct from the other homologues, which did not oscillate (Figure 7). Genes encoding the chlorophyll *a/b*-binding polypeptides of the light harvesting system (*Lhca/b*, also called *cab*) are controlled by the circadian clock in some plant species [58-61]. Eleven *Lhca/b* genes in *Arabidopsis* reveal circadian expression [30], and all 19 *Lhca/b* genes in tomato (*Solanum lycopersicum*) reveal diurnal and circadian expression [59]. Also, *Lhcb* mRNA in protonema cells of the moss *P. patens* and *LHCA1* mRNA in the green alga *Chlamydomonas reinhardtii* oscillate during light–dark cycles [60,61]. We obtained 13 full open reading frame (ORF) sequences from the NGS sequences of Japanese cedar that had high similarity to *Arabidopsis Lhca/b* genes based on the top BLASTX hit (Additional file 12). All 13 sequences showed e-values lower than 5e-74. A phylogenetic tree was constructed with homologues from *Arabidopsis*, Japanese rice, a lycophyte (*S. moellendroffii*) and a moss (*P. patens*), using distinct relatives of *Lhca/b* genes in *Arabidopsis*, *ELIP*1 and *ELIP2* [62], as an outgroup. The tree indicated 12 clusters consisting of 13 groups, Lhca1 through 6 and Lhcb1 through 7 (Additional file 12). One or two of the *Lhc* genes of Japanese cedar were classified into each group, except for the Lhcb7 group. Although the distinct relatives of *Lhca/b* genes, *ELIP*1 and *ELIP2* [62], showed significant oscillation (Additional file 4: Table S4), only four of the eight *Lhcb* genes (*CjLHCB1b*, *CjLHCB3*, *CjLHCB4a* and *CjLHCB6*) showed daily oscillations, but they were of small amplitude, and none of the *Lhca* genes oscillated. Genes encoding the photosystem I and II reaction centers did not cycle with more than two-fold changes in Japanese cedar, unlike *Arabidopsis* [30]*.* The *phyD* gene of moss also showed a diurnal rhythm, with peaks observed in the light phase [63]. In conifers, light-independent expression of photosynthetic genes seems to be a general phenomenon [64]. *Lhcb* and *psb*A (photosystem II subunit A) mRNA levels show only small diurnal fluctuations under light/dark cycles and no circadian rhythm under continuous light or dark conditions in Douglas fir (*Pseudotsuga menziesii*) [65]. By analyzing dark-grown seedlings, expression of *Lhcb* genes has been shown to occur in darkness in various conifer species [64-69]. Expression of PHYA/C-related genes in dark-grown *P. abies* seedlings has also been reported [69]. The mechanisms of regulation of these light-related genes in conifers might be different from those in angiosperms.

The microarray data showed massive transcriptional changes between summer and winter (Figure 1). The genes functioning in ‘developmental process’ and ‘cell organization and biogenesis’ were upregulated in summer (Figure 4), including gibberellin-related genes (gibberellin-regulated family protein and GA requiring 3) and genes related to cell division and elongation (cyclin, expansin and xyloglucan endotransglucosylase/hydrolase) (Additional file 8). In the winter, expression of genes for basic chitinase, osmotin, ELIP1 and late embryogenesis abundant protein 14 (LEA14), reportedly markers of cold hardiness in *P. sylvestris* [70], were also induced (Additional file 8). Diurnal transcriptome dynamics were not detected in the winter, and transcription of the core clock genes was constitutively high (Figure 8). Winter disruption of clock genes has also been observed in chestnut (*Castanea sativa*) [71,72]. Low temperature (4°C) reportedly reduces the amplitude of cycles for clock components in *Arabidopsis* and chestnut, and the cycles of output genes are dampened or disrupted in *Arabidopsis* [72,73]. A similar phenomenon might occur in Japanese cedar in winter. In *Arabidopsis*, core clock components CCA1 and LHY regulate expression of the CBF (C-REPEAT BINDING FACTOR) pathway, which is highly conserved among plants and has a major role in plant freezing tolerance [74]. Reducing the expression of *LHY* genes by RNA interference compromises freezing tolerance in *Populus* trees [16]. The expression of clock genes may be influenced by seasonal environmental changes, and consequently, may lead to activation of downstream pathways that contribute to freezing tolerance, which is important for survival of tree species in winter.

## Conclusion

Studies of diurnal and circadian mechanisms in plants have until recently focused on the model species *Arabidopsis*, with limited data available outside angiosperms. To the best of our knowledge, this study is the first exploration of diurnal transcriptome dynamics in gymnosperms. Microarray analysis showed significant differences in transcriptome dynamics between summer and winter. It also showed diurnal transcriptome dynamics in summer and revealed that 7.7% of the genes on the microarray were rhythmically expressed, while the rhythm was disrupted in winter. The cycling genes in summer constructed a gene network with the core clock genes, which may contribute to adaptation to diurnal and seasonal environmental changes. In summer, hormone-related genes tended to be upregulated from morning to noon and stress-related genes were upregulated at noon. These results indicated that the transcripts differed according to the sampling time, and that time at collection should be considered for analysis of transcription, because it may influence the results. Phylogenetic analysis indicated that conifers have genetically distinct clock genes from angiosperms. Conifers may contain unique diurnal regulation mechanisms. Interestingly, unlike angiosperms, photoreceptors and photosynthesis-related genes did not show significant oscillation in Japanese cedar. We also identified 182 cycling targets (sequences) that did not have BLASTX homologues in *Arabidopsis*. These proteins might play an important role in controlling diurnal rhythms unique to conifers. Independent studies are necessary for gymnosperms, and this study provides fundamental data to understand diurnal transcriptional regulation in conifers.

## Methods

### Plant material and RNA extraction

For library construction, samples were collected from Japanese cedars planted in 2008 (which were two years old at that time) at the Forest Tree Breeding Center (FTBC), Forestry and Forest Products Research Institute (Hitachi, Ibaraki, Japan). For SSH and cDNA library construction, shoots were collected at midday (12:00) and midnight (24:00) on Jun 2, 2010 from the same four individuals at each time point (8 samples total). A mixture of lateral branch apices 10 cm long collected from three different branches was referred to as a shoot sample. For NGS, diurnal time series samples were collected at four hour intervals from 4:00 to 24:00 on Jul 2, 2011 from three individuals at each time point (8 samples total). Seasonal time series samples were also collected at 10:00 at eight intervals covering a year (Dec 27, 2010, Feb 4, Apr 4, May 20, Jul 11, Aug 24, Oct 7, Nov 22, 2011) from three individuals at each time point (24 samples total). All samples for seasonal time series were collected from different individuals.

For microarray construction, qPCR and RACE (rapid amplification of cDNA ends) samples were collected from 36 potted three-year-old cuttings placed in an outdoor location at the FTBC. Shoot samples for a diurnal time series were collected every four hours for two days in winter (from 4:00, Dec 22 to 24:00, Dec 23, 2011) and summer (from 4:00, Jul 30 to 24:00, Jul 31, 2012). The day length (between sunrise and sunset) was approximately 9:42 hours in the winter and 14:06 in the summer, estimated by calculations on the National Astronomical Observatory of Japan website (http://eco.mtk.nao.ac.jp/cgi-bin/koyomi/koyomix.cgi). We collected samples from three cuttings at each time point as biological replicates. All samples were collected from different cuttings. Diurnal changes in air temperature, photosynthetically active radiation and growth in tree height are presented in Additional file 11.

All samples were immediately frozen in liquid nitrogen and stored at −80°C until use. Total RNA was extracted from 500 mg samples as Gehrig *et al.* reported [75] using an RNeasy Plant Mini Kit (Qiagen, Hilden, Germany) or RNeasy Midi Kit (Qiagen), and a DNase digestion was performed on-column using an RNase-free DNase Set (Qiagen). A NanoDrop 1000 spectrophotometer (Thermo Scientific, Waltham, MA, USA) was used to accurately measure RNA concentration. RNA integrity was assessed by an Agilent 2100 bioanalyzer (Agilent Technologies, Mississauga, ON, Canada), and only total RNA showing an RNA Integrity Number above eight was used.

### Library construction

Two SSH libraries were constructed using a Clontech PCR-Select cDNA Subtraction kit (Takara Bio, Shiga, Japan). Total RNA extracted from samples from four individuals was pooled in equal amounts, with one pool for samples taken at midday and one pool for midnight, and 100 ng of the resulting mixtures was used for library construction. A forward library (SSH12) was constructed by subtracting driver RNA isolated from shoots at midnight from tester RNA isolated from shoots at midday. A reverse library (SSH24) was constructed by subtracting driver RNA isolated from shoots at midday from tester RNA isolated from shoots at midnight. The SSH products were purified using a QIAquick PCR purification kit (Qiagen) and ligated into the pT4 Blue T-vector (Novagen, Los Angeles, CA, USA). Blue/white selection was conducted on plates containing ampicillin, isopropyl-D-thiogalactopyranoside and X-gal. Clones were randomly selected and single-pass sequenced using a U19 primer that matched vector sequence. Per library, 864 clones were sequenced using an ABI PRISM 3130 Genetic Analyzer (Applied Biosystems, Foster City, CA, USA). The resulting sequences were trimmed and edited manually to identify the cloning vector sequences, poly(A) sequence, and adaptor sequences used in the SSH procedure and regions of low-quality sequence using Sequencher version 4.10.1 software (Gene Codes Corp., Ann Arbor, MI, USA). Good-quality sequences longer than 89 bp were selected for further analysis. A total of 1,189 EST sequences were submitted to DDBJ [HX950378 through HX951566].

A normalized cDNA library was constructed using a total RNA mixture consisting of equal amounts of total RNA isolated from samples of the same four individuals collected at midday and midnight. Poly(A) + mRNA was isolated from 450 μg of the total RNA mixture using an Oligotex-dT30 < Super > mRNA Purification Kit (Takara Bio). The normalized cDNA library was constructed from 200 ng of the poly(A) + mRNA using a TRIMMER cDNA Normalization Kit (Evrogen, Moscow, Russia) and SMART cDNA Library Construction Kit (Takara Bio). Carbenicillin-resistant colonies were collected randomly for single-pass sequencing with a primer (5′-TCCGAGATCTGGACGAGC-3′) that recognizes vector sequences from the 5′-end of the inserts. The resulting sequences were trimmed and edited manually. Good-quality sequences longer than 100 bp were used for further analysis. A total of 2,653 cDNA sequences were submitted to DDBJ [HX951567 through HX954219].

NGS was carried out using an RNA mixture of diurnal and seasonal series. First, total RNA isolated from 18 samples of a diurnal time series and from 24 samples of a seasonal time series was respectively mixed in separate pools. Subsequently, the diurnal and seasonal total RNA mixtures were mixed in equal proportions and used as an RNA sample. Poly(A) + mRNA was isolated from 115 μg of the total RNA mixture using a MicroPoly(A)Purist Kit (Ambion, Austin, TX, USA). A cDNA library was constructed from 720 ng poly(A) + mRNA using the Primer Random, cDNA Synthesis System, and GS-FLX Titanium Rapid Library Preparation kits according to the protocols of the manufacturer (Roche, Basel, Switzerland), and then sequenced on the Roche GS-FLX system. The raw 454 sequence files in SFF format were base-called using GS Run Processor version 2.6 (Roche) to obtain clean ESTs. Adapter trimming and removal of poly(A/T) tails, low-complexity repetitive sequences and short sequences (<50 bp) were performed. BLASTN searches of the passed-filter reads were performed against SSRs of Japanese cedar registered in the Sugi Genome Database (http://www.ffpri.affrc.go.jp/labs/cjgenome/) and *Arabidopsis* retrotransposons registered in TAIR (The Arabidopsis Information Resource; http://www.arabidopsis.org), and the reads satisfying matching conditions (alignment length ≤200 bp; identity ≤90%) were excluded. The passed-filter reads were assembled using GS De Novo Assembler version 2.6 (Roche) with the default setting of cDNA project mode, and putative transcript sequences were predicted by assembling reads into isotigs. Files containing these sequences and their quality scores have been deposited at DDBJ [DRA001261].

Gene annotations represent the top-scoring BLASTX hits for each sequence’s predicted protein product as a query against the TAIR *Arabidopsis* protein database TAIR10-pep-20101214 with a threshold e-value of e-10. BLASTX searches were performed using the CLC Genomic Workbench version 4.1.1 software package (CLC bio, Aarhus, Denmark) for sequence data from SSH and normalized cDNA libraries, and the NCBI (National Center for Biotechnology Information) BLAST v2.2.25 algorithm (stand-alone; http://www.ncbi.nlm.nih.gov/guide/data-software/#downloads_) for the NGS data. Functional categorization was performed on the TAIR website based on GO annotation.

### Gene expression profiling by microarray

A NimbleGen Custom Eukaryotic Gene Expression 4 × 72 K Array produced by Roche NimbleGen (Madison, WI, USA) was used for microarray analysis. To identify diurnal rhythms of transcripts, all 36 summer samples and 8 selected winter samples (4:00/8:00/12:00/16:00/20:00/24:00 on Dec 22, and 12:00/24:00 on Dec 23) were analyzed using the microarray. Total RNA (10 μg) was transcribed to double-stranded cDNA using a SuperScript double-stranded cDNA synthesis kit (Invitrogen, Carlsbad, CA, USA) in the presence of 100 pmol oligo(dT)20 primer (Invitrogen) in accordance with the NimbleGen gene expression analysis protocol. Double-stranded cDNA was cleaned and labeled by a NimbleGen One-Color Labeling Kit. The microarray was hybridized at 42°C for 17 h with 3 μg of Cy3-labeled double-stranded cDNA in the NimbleGen hybridization system. Following hybridization, the microarray was washed using the NimbleGen Wash Buffer Kit. The slides were scanned at 2 μm/pixel resolution using a NimbleGen MS 200 scanner. The microarray design and data have been submitted to NCBI GEO [GSE53945].

### Microarray data treatment and statistical analysis

Scanned images (TIFF format) were imported into NimbleScan software version 2.6 for grid alignment and robust multi-array average normalization. To enable direct comparisons of transcript profiles, median log_2_-transformed ratios for each time point were normalized to the baseline using GeneSpring version 12.5 software (Agilent Technologies).

PCA was performed using the normalized dataset of 36 summer and 8 winter samples by GeneSpring software. The microarray data of summer samples were categorized into 12 groups according to the sampling time. Since no target on the microarray showed a significant difference between 12:00 and 24:00 using the GeneSpring moderated t-test (p >0.05), we estimated that there were few or no cycling genes in winter, and the data for all 8 winter samples were grouped together. The 13 groups were compared for all possible combinations using the GeneSpring pairwise comparison (one-way ANOVA, p-value ≤0.05, Benjamini and Hochberg multiple-testing correction) to select differentially expressed genes. To avoid analyzing targets from an identical gene, targets with hits to a unique *Arabidopsis* gene ID with a lower e-value after a BLASTX search were selected as unique genes from the differentially expressed genes for PCA.

Periodically expressed targets that showed statistically significant differences in expression (false discovery rate q-value ≤0.05) in the normalized datasets of 36 summer samples were identified using the GeneCycle R package [39]. Subsequently, we compared all possible combinations of average expression value at each time point, and selected expressed targets with more than two-fold differences in at least one pairwise comparison from cycling genes. Unique genes were selected from these targets, and classified into four clusters by GeneSpring software (algorithm: k-means, similarity measure: differential, maximum number of iterations: 10,000).

We estimated a probabilistic network of relationships between the 1,000 targets with the highest coefficient of variation in the normalized datasets of 36 summer samples using a Bayesian network estimation program, SiGN-BN (http://sign.hgc.jp/signbn/index.html) [76], implemented on the supercomputer system at the Human Genome Center, Institute of Medical Science, University of Tokyo (https://supcom.hgc.jp/english). The estimated gene network was analyzed using a gene network analysis platform, Cell Illustrator (https://cio.bioillustrator.com/cionlineserver/apps/usersman/main).

To identify transcriptome differences between summer and winter, the microarray data of 36 summer and 8 winter samples were grouped respectively without reference to sampling time, and compared by a moderated t-test (p-value ≤0.05, Benjamini and Hochberg multiple-testing correction) to select genes differentially expressed between summer and winter. Subsequently, targets that showed more than a four-fold difference and hit unique *Arabidopsis* gene IDs with lower e-values were selected and compared between summer and winter by GO categorization.

### Isolation of core clock and photoreceptor genes from Japanese cedar

Nucleic acid sequences of *Arabidopsis* core clock components [TAIR: AT1G01060 (*LHY*), AT2G46830 (*CCA1*), AT5G61380 (*TOC1*), AT1G22770 (*GI*) and AT5G57360 (*ZTL*)] and photoreceptors [AT1G09570 (*PHYA*), AT2G18790 (*PHYB*), AT4G08920 (*CRY1*) and AT1G04400 (*CRY2*)] were obtained from the KEGG pathway database. A TBLASTN search was performed against the NGS data on the CLC Genomic Workbench using the *Arabidopsis* sequences to identify putative homologues in Japanese cedar. Also, a TBLASTN search was performed against another NGS data set of transcripts expressed in treetops of Japanese cedar (Nose *et al.* unpublished data). Homologous contigs and singletons were reassembled using Sequencher software, and 5′- and 3′-RACE primers were designed using Oligo software package version 7 (National Biosciences Inc., Cascade, CO, USA, Additional file 13A). Total RNA extracted from the shoots collected at the six time points in Jul 31, 2012 were mixed in equal amounts. The first-strand cDNA sample was synthesized from 500 ng of the total RNA mixture using a SMARTer RACE cDNA Amplification Kit (Takara Bio), and then diluted by adding 100 μl Tricine-EDTA buffer. The PCR mixture (20 μl) consisted of 0.4 μl KOD-Plus polymerase (1.0 unit/μl, Toyobo, Osaka, Japan), 2.0 μl 10× buffer for KOD-Plus polymerase, 2.0 μl 2.0 mM dNTPs, 0.8 μl 25 mM MgSO_4_, 4.0 μl 1.0 μM RACE primer, 2.0 μl 10× universal primer A mix and 1.0 μl diluted 5′ or 3′ cDNA sample. The reaction conditions were optimized for each primer pair and consisted of initial denaturation at 94°C for 2 min, 20–35 cycles of 94°C for 15 sec, 60°C for 30 sec, 68°C for 1.5-4.0 min, and final extension at 68°C for 5 min (Additional file 13A). A 15 μl aliquot of the PCR product was electrophoresed on a 1.2% agarose gel and the fragment of expected length was extracted and purified using a QIAEX II Gel Extraction Kit (Qiagen) according to the manufacturer’s instructions. Nested PCR was performed when no band was detected in the first PCR. The nested PCR was essentially performed as described above, with 4.0 μl 1.0 μM second RACE primer (Additional file 13A), 0.4 μl 10 μM Nested Universal Primer A and 1.0 μl of the first PCR product diluted 1/100 with sterilized water. The phosphorylation reaction mixture (10 μl) consisted of 2.0 μl purified PCR product, 0.2 μl T4 polynucleotide kinase (Takara Bio), 1.0 μl 10× T4 polynucleotide kinase buffer and 0.1 μl 100 mM ATP, and was incubated for 30 min at 37°C. The phosphorylated product was purified using a QiaQuick PCR Purification Kit (Qiagen), ligated to vector pBSK using DNA Ligation Kit version 2.1 (Takara Bio), and then transformed into ECOS competent *Escherichia coli* DH5α (Nippongene, Tokyo, Japan). Ampicillin-resistant colonies were selected and the plasmid was purified using a Plasmid MiniPrep Kit (Millipore, Bedford, MA, USA). Sequencing was carried out with U19 and M13 reverse primers. A PCR primer pair was designed to amplify a full-length putative ORF region of the core clock and photoreceptor genes using the Oligo software program based on the sequence of RACE products. First-strand cDNA was synthesized from 500 ng of the same total RNA used for the RACE reaction using a PrimeScript 1st strand cDNA Synthesis Kit (Takara Bio). The PCR was basically performed as described above using 1.0 μl of the cDNA diluted 1/100 with sterilized water; the modified conditions are listed in Additional file 13B. Gel purification, cloning and sequencing were performed as described above. The sequences were assembled and aligned with Sequencher software. The aligned sequences were translated into amino acid sequences using CLC Main Workbench version 6.5 software (CLC bio), and a BLASTP search against the TAIR protein database was performed to confirm the cloned genes. Phylogenetic analysis was performed with the amino acid sequences of homologous genes from a wide variety of plant species registered in the NCBI and KEGG databases using ClustalW version 2.1 software on the DDBJ website (http://clustalw.ddbj.nig.ac.jp/index.php?lang=ja) in default mode. The neighbor-joining method [77] was used to construct the phylogenetic trees.

### qPCR of clock and photoreceptor genes

qPCR was carried out for 12 genes isolated in this study that apparently encode putative core clock components or photoreceptors. A gene-specific primer pair was designed within each ORF region (Additional file 13C). First-strand cDNA was synthesized from 500 ng of total RNA extracted from the diurnal series of 36 summer and from total RNA of the 36 winter samples using a High Capacity RNA-to-cDNA Kit (Life Technologies, Carlsbad, CA, USA). qPCR was performed with *Power* SYBR Green PCR Master Mix (Life Technologies) and a StepOnePlus Real-Time PCR system (Life Technologies), as described in the manufacturer’s instructions. A 6 μl aliquot of cDNA diluted 1/24 with sterilized water was used in a reaction volume of 20 μl per well. Melting curve analysis was performed from 60 to 95°C, with data captured every 0.3°C to ensure amplification of a single product. Reaction efficiency was checked using standard curves based on a four-fold dilution series of cDNA synthesized from 500 ng of total RNA (1 to 1/256 dilution). Each sample was tested independently and in triplicate using all primers. Transcript abundance was normalized to ubiquitin registered in the ForestGEN database (http://forestgen.ffpri.affrc.go.jp/ja/info_cj.html) [Cj. 2620] using the ∆∆Ct method [78], and the data obtained for each time point were compared with the data obtained for shoots collected at 4:00 on Jul 30, 2012.

### Availability of supporting data

NGS data [DRA001261], EST sequences of SSH and cDNA libraries [HX950378 through HX954219] and sequences of clock related genes [AB894538 through AB894549] are available in the DDBJ. The microarray design and data are available in the NCBI GEO [GSE53945].

## Additional files

Additional file 1:
**Summary of SSH, cDNA and NGS data.**
Additional file 2:
**Gene ontology assignment for SSH, cDNA and NGS data.** (A) Proportion of annotated ESTs from SSH libraries of Japanese cedar. Forward (SSH12) and reverse (SSH24) libraries represented genes expressed predominantly at midday and midnight in summer. (B) Proportion of annotated ESTs from the normalized cDNA library of Japanese cedar sampled in summer. (C) Proportion of annotated isotigs and singletons from the NGS data of Japanese cedar sampled throughout the day and year. Additional file 3:
**Frequency distribution of reads (A) and length of assembled isotigs (B) from NGS data.**
Additional file 4:
**The 999 targets selected that showed diurnal rhythms with more than two-fold differences in peak-to-trough amplitude.** ^1^The putative function of the sequences was predicted according to the highest BLASTX hits with an e-value cutoff of e-10. Only sequences that hit a unique *Arabidopsis* gene ID are listed here. ^2^p-value and false discovery rate (q-value) show the results of statistical analysis by GeneCycle [39]. Only genes with a q-value ≤0.05 are listed here. ^3^Fold change indicates the ratio of maximal and minimal expression in summer. ^4^ The times when maximum and minimum expression were observed by microarray. Additional file 5:
**The 556 cycling genes used for cluster analysis.** ^1^The putative function of the sequences was predicted according to the highest BLASTX hits with an e-value cutoff of e-10. Only sequences that hit a unique *Arabidopsis* gene ID are listed here. ^2^Fold change indicates the ratio of maximal and minimal expression in summer. ^3^Cluster number indicates a similarity in expression patterns defined by cluster analysis. See Figure 2A for the expression pattern of each cluster. ^4^Putative hormone-related genes representing hormone biosynthesis and signaling pathways are listed here (ABA, abscisic acid; AUX, auxin; BR, brassinosteroids; CK, cytokinins; ETH, ethylene; GA, gibberellin; JA, jasmonic acid). Additional file 6:
**Targets with the highest coefficient of variations used for gene network analysis.** ^1^The putative function of the sequences was predicted according to the highest BLASTX hits with an e-value cutoff of e-10. ^2^Putative clock gene with node colored red in the estimated gene network (Figure 4). ^3^Number of children, parents and all edges were estimated by the SiGN-BN Bayesian network estimation program (http://sign.hgc.jp/signbn/index.html) [76]. ^4^The time when the target reached maximum expression. Additional file 7:
**Estimated gene network of microarray data.** This gene network was estimated by the SiGN-BN program [76] based on 1,000 targets with the highest coefficient of variations in summer. We extracted 2,604 edges that showed bootstrap probability higher than 0.7 and 886 related targets. Detailed information on the targets (annotations, number of children and parents, and peak time) is listed in Additional file 6. This file is in csml format for Cell Illustrator software. Additional file 8:
**The top 100 targets differentially expressed in summer and winter.** ^1^The putative function of the sequences was predicted according to the highest BLASTX hits with an e-value cutoff of e-10. Additional file 9:
**Amino acid sequence alignment of LHY and CCA1 (A), TOC1 (B), GI (C), and ZTL, LKP2 and FKF1 (D).** The species names are abbreviated as follows: At, *Arabidopsis thaliana*; Cj, *Cryptomeria japonica*; Pp, *Physcomitrella patens* subsp. *patens*; Sm, *Selaginella moellendorffii.* (A) NCBI accession numbers of the proteins are AtLHY (NP_001030924) and AtCCA1 (NP_850460). PpCCA1a and PpCCA1b are from Okada *et al.* [41]. The amino acid sequences of the domains (underlined) are from Wang *et al.* [40]. (B) NCBI accession numbers of the proteins are NP_200946 (AtTOC1) and XP_002963903 (SmTOC1). The amino acid sequences of the domains (underlined) are from Strayer *et al.* [42]. (C) NCBI accession numbers of the proteins are NP_564180 (AtGI) and XP_002961231 (SmGI). (D) NCBI accession numbers of the proteins are NP_001154783 (AtZTL), AEC06826 (AtLKP2), AAF32298 (FKF1) and XP_002990856 (SmFKF1-2). The amino acid sequences of the domains (underlined) are from Somers *et al.* [43]. Additional file 10:
**Diurnal expression patterns of photoreceptor genes in summer and winter.** Diurnal changes in relative transcript abundance in summer (Jul 30–31, 2012) and winter (Dec 22–23, 2011) were analyzed by microarray and qPCR. The microarray data of summer samples (green line) and qPCR data of summer (blue line) and winter (red line) samples represent the mean from three biological replicates of 12 time points. The microarray data of winter samples (purple line) are shown at 8 selected time points (4:00/8:00/12:00/16:00/20:00/24:00 on day 1 and 12:00/24:00 on day 2) without biological replication. The data obtained for each time point were compared with the data obtained for shoots collected at 4:00 on Jul 30, 2012. Gray and black bars below graph represent length of natural day and night (between sunrise and sunset), respectively, in summer (upper) and winter (lower) as reported by the National Astronomical Observatory of Japan. Additional file 11:
**Diurnal changes in temperature, photosynthetically active radiation and growth in height of Japanese cedar.** (A) Changes in temperature over two days in winter (Dec 22–23, 2011) and summer (Jul 30–31, 2012). Temperature data were collected every 10 min in Hitachi (36°34′N 140°38′E 34 m, about 15 km from the sampling site) and were provided by the Japan Meteorological Agency (http://www.jma.go.jp/jma/index.html). (B) Changes in photosynthetically active radiation over two days in summer. Values were obtained every 20 min from the photosynthetically active radiation smart sensor working with the HOBO Weather Station logger (Onset Computer Corp., Bourne, MA, USA) at the sampling site. (C) Change in growth in height over five days (Jul 26–30, 2012). Images of a treetop were captured every hour by a WG-II digital camera (Pentax, Tokyo, Japan). Growth in height was estimated by measuring the images with ImageJ64 software (http://rsbweb.nih.gov/ij/). Additional file 12:
**Phylogenetic tree of LHCa/b in** ***Arabidopsis*** **, rice, moss and Japanese cedar.** LHCa/b gene sequences of Japanese cedar were extracted from the NGS data. Species names are abbreviated as follows: At, *Arabidopsis thaliana* (thale cress); Cj, Japanese cedar (*Cryptomeria japonica*); Osj, *Oryza sativa* (Japanese rice); Pp, *Physcomitrella patens* subsp. *patens* (moss). The number following the species name indicates NCBI accession number. The neighbor-joining method [77] was used to construct the phylogenetic trees. Trees were rooted with *Arabidopsis* ELIP1 and ELIP2. Additional file 13:
**RACE, PCR and qPCR primers for core clock and photoreceptor genes.**
