# Supplementary material for: Clock genes and diurnal transcriptome dynamics in summer and winter in the gymnosperm Japanese cedar (Cryptomeria japonica (L.f.) D.Don)
Source: BMC Plant Biol. 2014 Nov 18;14:308. doi: 10.1186/s12870-014-0308-1 (PMC4245765; doi:10.1186/s12870-014-0308-1)
Supplement: Additional file 1: — Summary of SSH, cDNA and NGS data. [file 12870_2014_308_MOESM1_ESM.pdf]

Additional file 1. **Summary of SSH, cDNA and NGS data.**

library name	method	sampling time	genes expressed at	number of sequence	number of sequence used in microarray
SSH12	SSH	Jun 2, 2010 (12:00, 24:00)	mainly midday in summer	595 sequences	324 sequences
SSH24			mainly midnight in summer	594 sequences	338 sequences
cDNA	normarized cDNA		midday and midnight in summer	2,653 sequences	1,854 sequences
NGS	Roche 454	Diurnal: Jul 2, 2011 (4:00, 8:00, 12:00, 16:00, 20:00, 24:00)<br>Seasonal: Dec 27, 2010, Feb 4, Apr 4, May 20, Jul 11, Aug 24, Oct 7, Nov 22, 2011 (10:00)	throughout the day and year	6,890 isotigs<br>45,112 singletons	6,578 isotigs<br>6,634 singletons
