# Supplementary material for: Clock genes and diurnal transcriptome dynamics in summer and winter in the gymnosperm Japanese cedar (Cryptomeria japonica (L.f.) D.Don)
Source: BMC Plant Biol. 2014 Nov 18;14:308. doi: 10.1186/s12870-014-0308-1 (PMC4245765; doi:10.1186/s12870-014-0308-1)
Supplement: Additional file 2: — Gene ontology assignment for SSH, cDNA and NGS data. (A) Proportion of annotated ESTs from SSH libraries of Japanese cedar. Forward (SSH12) and reverse (SSH24) libraries represented genes expressed predominantly at midday and midnight in summer. (B) Proportion of annotated ESTs from the normalized cDNA library of Japanese cedar sampled in summer. (C) Proportion of annotated isotigs and singletons from the NGS data of Japanese cedar sampled throughout the day and year. [file 12870_2014_308_MOESM2_ESM.pdf]

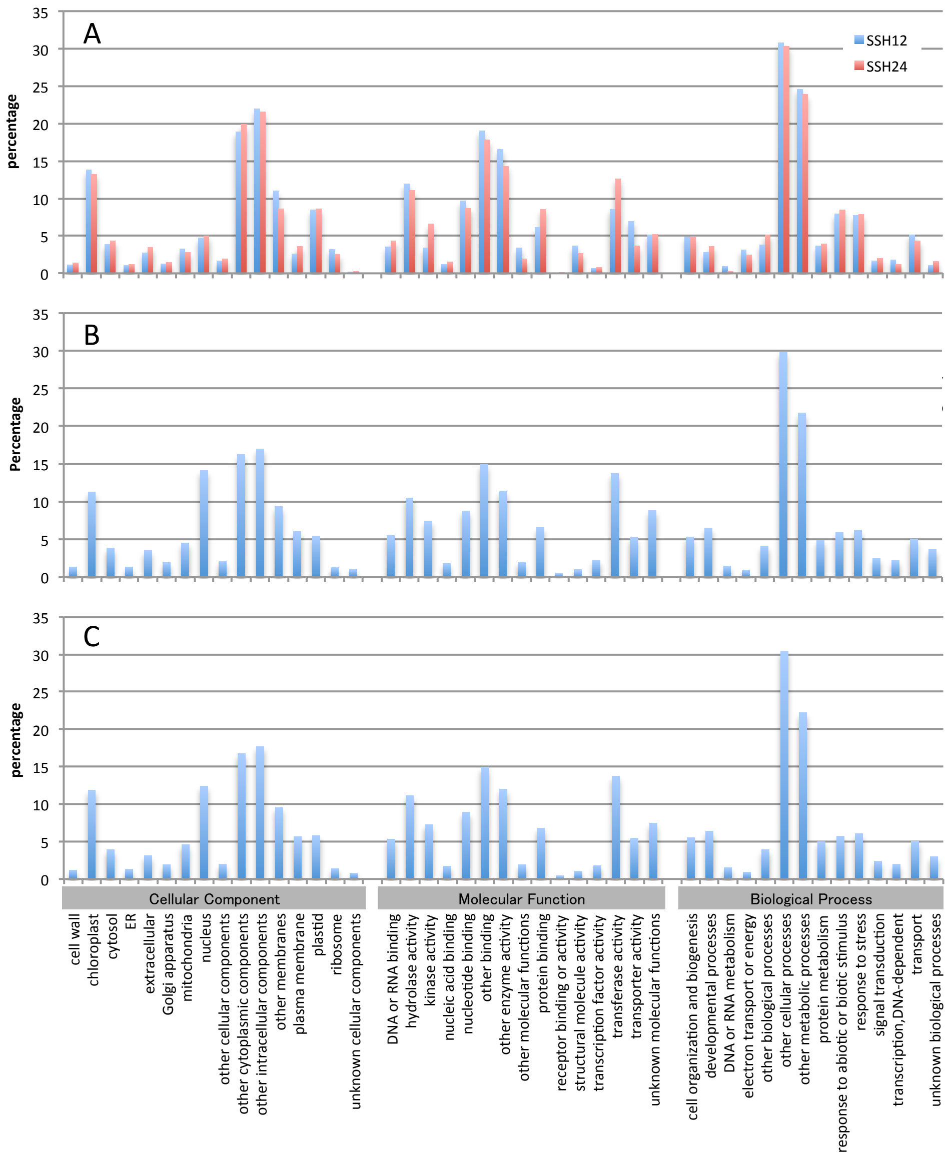

Additional file 2. Gene ontology assignment for SSH, cDNA and NGS data.

(A) Proportion of annotated ESTs from SSH libraries of Japanese cedar. Forward (SSH12) and reverse (SSH24) libraries represented genes expressed predominantly at midday and midnight in summer.

(B) Proportion of annotated ESTs from the normalized cDNA library of Japanese cedar sampled in summer.

(C) Proportion of annotated isotigs and singletons from the NGS data of Japanese cedar sampled throughout the day and year.
