# Supplementary material for: Clock genes and diurnal transcriptome dynamics in summer and winter in the gymnosperm Japanese cedar (Cryptomeria japonica (L.f.) D.Don)
Source: BMC Plant Biol. 2014 Nov 18;14:308. doi: 10.1186/s12870-014-0308-1 (PMC4245765; doi:10.1186/s12870-014-0308-1)
Supplement: Additional file 3: — Frequency distribution of reads (A) and length of assembled isotigs (B) from NGS data. [file 12870_2014_308_MOESM3_ESM.pdf]

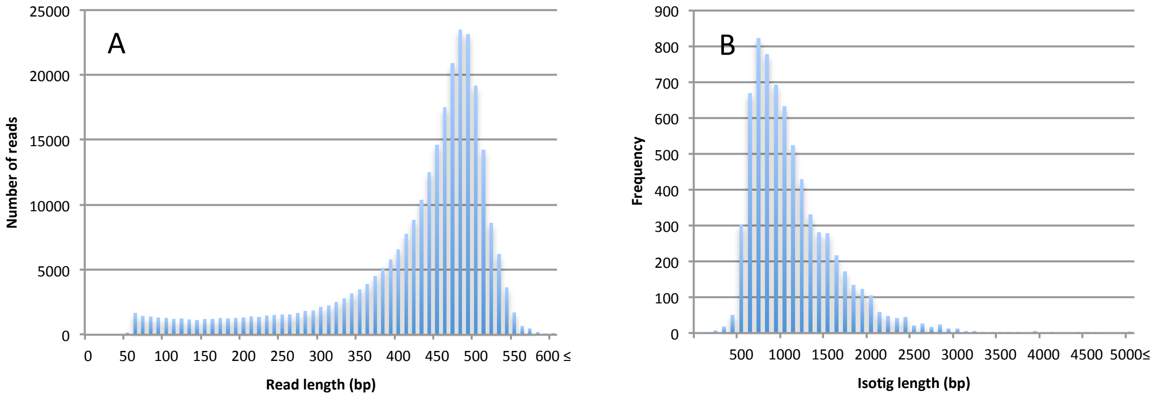

Additional file 3. Frequency distribution of reads (A) and length of assembled isotigs (B) from NGS data.
