# Supplementary material for: Clock genes and diurnal transcriptome dynamics in summer and winter in the gymnosperm Japanese cedar (Cryptomeria japonica (L.f.) D.Don)
Source: BMC Plant Biol. 2014 Nov 18;14:308. doi: 10.1186/s12870-014-0308-1 (PMC4245765; doi:10.1186/s12870-014-0308-1)
Supplement: Additional file 4: — The 999 targets selected that showed diurnal rhythms with more than two-fold differences in peak-to-trough amplitude. 1The putative function of the sequences was predicted according to the highest BLASTX hits with an e-value cutoff of e-10. Only sequences that hit a unique Arabidopsis gene ID are listed here. 2p-value and false discovery rate (q-value) show the results of statistical analysis by GeneCycle [39]. Only genes with a q-value ≤0.05 are listed here. 3Fold change indicates the ratio of maximal and minimal expression in summer. 4 The times when maximum and minimum expression were observed by microarray. [file 12870_2014_308_MOESM4_ESM.pdf]

Additional file 4. **The 999 targets selected that showed diurnal rhythms with more than two-fold differences in peak-to-trough amplitude.**

No.	SEQ_ID	BLASTX[1]				GeneCycle[2]		fold change[3]	max[4]	min[4]
		Accession	Symbols	Description	e-value	p-value	q-value			
1	HI9HAF203DGNH4	AT1G01060	LHY	Homeodomain-like superfamily protein	2.0E-17	5.0E-16	5.3E-13	129.6	4:00	20:00
2	HI9HAF203C2ZM8	AT1G01060	LHY	Homeodomain-like superfamily protein	2.0E-17	5.2E-16	5.3E-13	95.5	4:00	20:00
3	HI9HAF202B62CT	AT1G01060	LHY	Homeodomain-like superfamily protein	8.0E-24	1.1E-15	1.4E-12	90.9	4:00	20:00
4	HI9HAF202BX81J	AT1G01060	LHY	Homeodomain-like superfamily protein	9.0E-34	5.5E-16	7.0E-13	82.1	4:00	20:00
5	isotig01218	AT5G02810	PRR7	pseudo-response regulator 7	0.0	1.2E-08	5.0E-07	65.8	12:00	4:00
6	HI9HAF203DC17O	AT5G59720	HSP18.2	heat shock protein 18.2	0.0	5.0E-06	7.3E-05	64.2	12:00	24:00
7	HI9HAF202CA57Y	AT1G22770	GI	gigantea protein (GI)	0.0	1.9E-14	1.1E-11	54.8	16:00	4:00
8	isotig04596	AT5G12020	HSP17.6II	17.6 kDa class II heat shock protein	4.0E-41	4.4E-13	1.4E-10	52.3	12:00	24:00
9	HI9HAF202CJUSS	AT1G22770	GI	gigantea protein (GI)	9.8E-45	5.2E-14	2.7E-11	46.7	16:00	4:00
10	isotig00993	–	–	–	–	1.5E-10	1.2E-08	42.9	8:00	24:00
11	HI9HAF203DEFZZ	AT1G22770	GI	gigantea protein (GI)	6.0E-25	3.4E-14	2.7E-11	42.3	16:00	4:00
12	HI9HAF203DARNP	AT1G22770	GI	gigantea protein (GI)	1.0E-11	2.6E-14	2.7E-11	41.2	16:00	4:00
13	HI9HAF202B4Y9S	AT1G22770	GI	gigantea protein (GI)	6.0E-25	4.7E-14	2.7E-11	36.4	16:00	4:00
14	HI9HAF202CLX46	AT1G22770	GI	gigantea protein (GI)	0.0	9.3E-15	1.1E-11	35.8	16:00	4:00
15	isotig05665	AT1G22770	GI	gigantea protein (GI)	0.0	9.2E-14	2.7E-11	34.7	16:00	4:00
16	isotig01031	AT3G46230	ATHSP17.4	heat shock protein 17.4	5.0E-43	1.7E-07	3.3E-06	33.1	12:00	24:00
17	isotig02884	–	–	–	–	3.4E-10	2.3E-08	32.0	8:00	24:00
18	isotig01537	AT1G01060	LHY	Homeodomain-like superfamily protein	1.0E-12	5.0E-16	5.3E-13	31.9	4:00	20:00
19	isotig03902	AT5G64170	–	dentin sialophosphoprotein-related	5.0E-11	9.4E-17	5.3E-13	31.5	8:00	20:00
20	isotig00231	–	–	–	–	2.9E-10	2.3E-08	29.1	16:00	4:00
21	isotig05105	–	–	–	–	2.8E-10	1.8E-08	29.0	16:00	4:00
22	isotig00992	–	–	–	–	4.1E-10	2.4E-08	27.4	8:00	24:00
23	isotig02783	AT2G26150	ATHSFA2	heat shock transcription factor A2	0.0	8.7E-06	1.5E-04	26.7	20:00	8:00
24	SSH12-7-75_006_C10	–	–	–	–	6.4E-09	2.0E-07	26.2	12:00	4:00
25	isotig05838	–	–	–	–	3.0E-10	2.3E-08	24.9	8:00	24:00
26	Shoot-056-25	–	–	–	–	1.9E-14	1.1E-11	24.8	8:00	20:00
27	isotig05469	AT5G59720	HSP18.2	heat shock protein 18.2	1.0E-41	1.6E-07	3.2E-06	24.6	12:00	24:00
28	isotig05923	–	–	–	–	4.0E-10	2.4E-08	22.2	16:00	8:00
29	isotig04903	AT4G27670	HSP21	heat shock protein 21	0.0	5.6E-10	3.1E-08	21.3	12:00	4:00
30	isotig01030	AT3G46230	ATHSP17.4	heat shock protein 17.4	3.0E-43	8.1E-07	1.8E-05	20.9	12:00	24:00
31	isotig05853	AT3G46230	ATHSP17.4	heat shock protein 17.4	0.0	2.7E-12	6.6E-10	20.0	12:00	24:00
32	isotig04743	AT3G22840	ELIP1	Chlorophyll A-B binding family protein	2.0E-37	4.6E-03	3.8E-02	19.3	12:00	4:00
33	isotig00466	AT5G52640	HSP81-1	heat shock protein 90.1	0.0	9.8E-08	2.6E-06	18.9	12:00	24:00
34	isotig06420	–	–	–	–	8.0E-04	8.1E-03	18.5	20:00	4:00
35	HI9HAF203DOXJZ	AT5G39660	CDF2	cycling DOF factor 2	4.0E-41	5.7E-15	1.1E-11	17.8	8:00	16:00
36	isotig04474	AT4G14690	ELIP2	Chlorophyll A-B binding family protein	4.0E-42	9.5E-08	2.6E-06	17.2	12:00	4:00
37	isotig03449	AT5G54470	–	B-box type zinc finger family protein	2.0E-18	8.9E-13	6.0E-10	16.4	8:00	24:00
38	isotig06607	AT3G22840	ELIP1	Chlorophyll A-B binding family protein	2.0E-19	5.8E-10	3.1E-08	16.1	12:00	4:00
39	isotig04281	AT3G22840	ELIP1	Chlorophyll A-B binding family protein	5.0E-34	2.0E-03	1.9E-02	15.9	8:00	20:00
40	HI9HAF203DFI5U	AT1G07850	–	Protein of unknown function (DUF604)	2.0E-41	1.4E-10	1.1E-08	15.4	4:00	20:00
41	isotig05861	–	–	–	–	1.2E-09	5.9E-08	15.0	16:00	8:00
42	Shoot-026-47	–	–	–	–	9.2E-10	5.0E-08	14.7	16:00	8:00
43	Shoot-054-07	–	–	–	–	6.6E-11	7.7E-09	14.3	4:00	16:00
44	Shoot-057-45	AT5G17300	RVE1	Homeodomain-like superfamily protein	5.0E-38	1.1E-13	2.7E-11	14.3	4:00	16:00
45	isotig06131	–	–	–	–	6.7E-16	1.4E-12	14.3	8:00	16:00
46	SSH24-7-44_008_D06	AT4G14622	CPuORF60	conserved peptide upstream open reading frame 60	4.0E-32	9.3E-08	2.3E-06	14.2	20:00	12:00
47	Shoot-057-21	–	–	–	–	2.6E-09	1.3E-07	13.7	16:00	4:00
48	isotig06028	–	–	–	–	3.2E-08	1.0E-06	13.5	12:00	4:00
49	isotig03688	–	–	–	–	6.1E-05	8.3E-04	13.2	20:00	12:00
50	isotig03787	–	–	–	–	8.8E-13	1.9E-10	13.2	4:00	16:00
51	HI9HAF203C266F	AT3G19270	CYP707A4	cytochrome P450, family 707, subfamily A, polypeptide 4	9.0E-41	5.9E-09	2.0E-07	12.8	12:00	4:00
52	Shoot-049-85	–	–	–	–	4.8E-06	7.3E-05	12.8	4:00	12:00
53	isotig00762	AT4G25000	ATAMY1	alpha-amylase-like	0.0	2.6E-08	1.0E-06	12.4	16:00	8:00
54	SSH24-3-25_002_A04	–	–	–	–	1.5E-12	6.6E-10	12.4	4:00	16:00
55	isotig00872	AT3G14200	–	Chaperone DnaJ-domain superfamily protein	4.0E-24	1.4E-13	8.3E-11	12.1	12:00	4:00
56	isotig06867	–	–	–	–	3.1E-09	1.3E-07	11.5	16:00	8:00
57	isotig02597	AT5G64170	–	dentin sialophosphoprotein-related	2.0E-12	1.4E-05	2.2E-04	11.3	12:00	4:00
58	Shoot-026-13	–	–	–	–	1.8E-06	3.7E-05	11.1	4:00	12:00
59	HI9HAF202CJNDC	–	–	–	–	2.0E-04	2.3E-03	11.0	20:00	4:00
60	isotig04131	AT4G11650	ATOSM34	osmotin 34	0.0	2.0E-03	1.9E-02	10.8	16:00	8:00
61	isotig04359	AT4G14690	ELIP2	Chlorophyll A-B binding family protein	7.0E-36	2.6E-06	6.5E-05	10.5	12:00	24:00
62	SSH24-6-07_013_G01	–	–	–	–	1.0E-13	2.7E-11	10.4	4:00	16:00
63	HI9HAF203CY2MA	AT1G73220	AtOCT1	organic cation/carnitine transporter1	0.0	4.5E-10	2.4E-08	9.9	8:00	20:00
64	isotig05247	AT3G21150	BBX32	B-box 32	5.0E-16	5.6E-13	1.4E-10	9.9	8:00	20:00
65	HI9HAF203DEENJ	AT5G02500	HSC70-1	heat shock cognate protein 70-1	0.0	6.6E-08	1.9E-06	9.8	12:00	4:00
66	Shoot-039-84	AT4G29520	–	–	0.0	2.2E-05	4.0E-04	9.8	12:00	24:00
67	isotig05235	AT5G12020	HSP17.6II	17.6 kDa class II heat shock protein	4.0E-38	2.0E-13	8.3E-11	9.5	12:00	24:00
68	isotig05771	AT5G12020	HSP17.6II	17.6 kDa class II heat shock protein	2.0E-33	1.2E-09	5.9E-08	9.3	12:00	24:00
69	isotig00738	AT5G06570	–	alpha/beta-Hydrolases superfamily protein	7.0E-44	1.3E-03	1.3E-02	9.2	16:00	4:00
70	isotig04059	AT2G21100	–	Disease resistance-responsive (dirigent-like protein) family protein	1.4E-45	3.3E-11	3.9E-09	9.1	16:00	4:00
71	HI9HAF202B43DT	AT5G13490	AAC2	ADP/ATP carrier 2	0.0	2.6E-03	2.3E-02	9.1	4:00	16:00
72	isotig05126	AT3G10910	–	RING/U-box superfamily protein	1.0E-12	6.1E-09	2.0E-07	9.0	8:00	16:00
73	isotig05544	AT2G45580	CYP76C3	cytochrome P450, family 76, subfamily C, polypeptide 3	3.0E-39	7.5E-13	1.4E-10	8.8	16:00	4:00
74	isotig04041	AT4G27670	HSP21	heat shock protein 21	0.0	5.3E-10	3.1E-08	8.7	12:00	4:00
75	isotig03433	AT3G54500	–	–	2.0E-30	3.1E-06	6.9E-05	8.6	12:00	4:00
76	HI9HAF202BXV4N	AT2G32120	HSP70T-2	heat-shock protein 70T-2	2.0E-42	4.3E-10	2.4E-08	8.5	12:00	4:00
77	isotig03899	AT5G51550	EXL3	EXORDIUM like 3	0.0	2.3E-11	2.5E-09	8.4	4:00	12:00
78	isotig03495	AT3G22840	ELIP1	Chlorophyll A-B binding family protein	3.0E-40	2.9E-03	2.4E-02	8.4	12:00	20:00
79	HI9HAF202BX9NX	AT1G64890	–	Major facilitator superfamily protein	7.0E-44	1.4E-10	1.1E-08	8.3	16:00	8:00
80	isotig00773	–	–	–	–	1.6E-07	3.2E-06	8.3	20:00	8:00
81	HI9HAF202BX0CC	AT1G64890	–	Major facilitator superfamily protein	5.0E-44	1.2E-10	8.8E-09	8.2	16:00	8:00
82	isotig00878	AT1G48130	ATPER1	1-cysteine peroxiredoxin 1	0.0	2.0E-05	3.4E-04	8.2	16:00	8:00
83	HI9HAF203DK482	AT5G06530	–	ABC-2 type transporter family protein	0.0	5.6E-12	1.1E-09	8.2	4:00	16:00
84	isotig03466	–	–	–	–	3.1E-10	2.3E-08	8.0	12:00	4:00
85	isotig06221	AT5G45340	CYP707A3	cytochrome P450, family 707, subfamily A, polypeptide 3	0.0	5.9E-09	2.0E-07	8.0	12:00	4:00
86	HI9HAF202B0QPY	AT5G42020	BIP	Heat shock protein 70 (Hsp 70) family protein	2.0E-41	3.1E-08	1.0E-06	8.0	12:00	24:00
87	Shoot-024-80	AT5G64170	–	dentin sialophosphoprotein-related	8.0E-19	7.1E-06	1.1E-04	7.9	12:00	4:00
88	HI9HAF202CBCF6	AT5G42020	BIP	Heat shock protein 70 (Hsp 70) family protein	2.0E-41	5.0E-08	1.5E-06	7.6	12:00	24:00
89	isotig04546	AT5G51440	–	HSP20-like chaperones superfamily protein	1.0E-30	3.8E-12	6.6E-10	7.6	12:00	24:00
90	Shoot-008-10_004_B02	AT5G08640	FLS	flavonol synthase 1	2.0E-40	1.5E-06	2.7E-05	7.4	16:00	4:00
91	isotig04207	AT5G49920	–	Octicosapeptide/Phox/Bem1p family protein	1.0E-23	4.5E-06	6.9E-05	7.4	20:00	4:00
92	isotig03376	AT3G12500	ATHCHIB	basic chitinase	0.0	1.7E-07	3.3E-06	7.4	16:00	8:00
93	isotig00772	AT1G71980	–	Protease-associated (PA) RING/U-box zinc finger family protein	0.0	1.8E-08	7.3E-07	7.4	20:00	8:00
94	Shoot-023-83	–	–	–	–	3.9E-08	1.4E-06	7.4	20:00	8:00
95	HI9HAF202CI8XY	AT2G32120	HSP70T-2	heat-shock protein 70T-2	2.0E-41	6.3E-10	3.1E-08	7.2	12:00	4:00
96	isotig03734	AT2G21320	–	B-box zinc finger family protein	2.0E-27	3.5E-12	6.6E-10	7.2	8:00	24:00
97	isotig05231	AT3G22840	ELIP1	Chlorophyll A-B binding family protein	8.0E-37	5.3E-03	4.3E-02	7.1	12:00	4:00
98	Shoot-008-90_004_B12	AT3G15810	–	Protein of unknown function (DUF567)	6.0E-18	4.0E-14	2.7E-11	7.1	12:00	4:00
99	isotig05855	AT3G15810	–	Protein of unknown function (DUF567)	1.0E-35	5.0E-13	1.4E-10	7.1	12:00	20:00
100	Shoot-047-23	AT2G38640	–	Protein of unknown function (DUF567)	3.0E-19	6.9E-13	1.4E-10	7.1	12:00	20:00

101	HI9HAF203CYISG	AT4G29520	–	–	0.0	5.6E-06	9.5E-05	7.1	12:00	24:00
102	isotig06627	–	–	–	–	4.3E-16	5.3E-13	7.0	16:00	4:00
103	HI9HAF202B2W3I	AT1G56410	ERD2	heat shock protein 70 (Hsp 70) family protein	0.0	2.9E-08	1.0E-06	6.9	12:00	4:00
104	isotig02211	AT1G23760	JP630	BURP domain-containing protein	2.0E-25	2.3E-03	2.3E-02	6.9	20:00	12:00
105	isotig06868	–	–	–	–	1.4E-07	3.2E-06	6.8	20:00	8:00
106	HI9HAF202B0P7R	AT1G73220	AtOCT1	organic cation/carnitine transporter1	8.0E-43	1.2E-09	5.9E-08	6.8	8:00	20:00
107	isotig06362	–	–	–	–	1.0E-04	1.1E-03	6.7	12:00	20:00
108	HI9HAF203DIF6L	AT5G02500	HSC70-1	heat shock cognate protein 70-1	0.0	3.3E-07	9.7E-06	6.6	12:00	4:00
109	HI9HAF202B8ZAL	AT5G08370	AtAGAL2	alpha-galactosidase 2	0.0	1.2E-03	1.3E-02	6.6	4:00	12:00
110	isotig05830	AT4G03290	–	EF hand calcium-binding protein family	6.0E-17	2.4E-06	6.5E-05	6.6	12:00	20:00
111	HI9HAF203C3I81	AT5G08370	AtAGAL2	alpha-galactosidase 2	0.0	2.5E-03	2.3E-02	6.5	4:00	12:00
112	isotig03680	AT5G55250	IAMT1	IAA carboxylmethyltransferase 1	3.0E-29	3.2E-10	2.3E-08	6.5	8:00	24:00
113	isotig04915	AT2G47180	AtGolS1	galactinol synthase 1	0.0	3.2E-10	2.3E-08	6.4	12:00	24:00
114	HI9HAF203CZ6FR	–	–	–	–	3.0E-11	3.9E-09	6.3	16:00	4:00
115	HI9HAF202CC49V	AT5G06530	–	ABC-2 type transporter family protein	1.4E-45	5.6E-12	7.9E-10	6.2	4:00	16:00
116	isotig05436	AT3G22840	ELIP1	Chlorophyll A-B binding family protein	1.0E-35	3.3E-03	2.9E-02	6.1	12:00	4:00
117	HI9HAF202CI8OC	AT1G20190	ATEXPA11	expansin 11	0.0	1.6E-07	3.2E-06	6.0	12:00	4:00
118	HI9HAF203C8YEA	AT1G20190	ATEXPA11	expansin 11	0.0	1.4E-07	3.2E-06	6.0	12:00	4:00
119	Shoot-049-21	AT1G76940	–	RNA-binding (RRM/RBD/RNP motifs) family protein	4.0E-27	2.2E-09	1.3E-07	6.0	12:00	8:00
120	HI9HAF202B3ZN6	AT4G00110	GAE3	UDP-D-glucuronate 4-epimerase 3	0.0	4.5E-11	5.2E-09	6.0	4:00	12:00
121	SSH12-5-49_001_A07	–	–	–	–	4.6E-12	6.6E-10	6.0	8:00	20:00
122	HI9HAF203DIMRN	AT4G00110	GAE3	UDP-D-glucuronate 4-epimerase 3	0.0	2.5E-10	1.8E-08	5.9	4:00	12:00
123	isotig04746	AT1G69830	ATAMY3	alpha-amylase-like 3	2.0E-39	4.0E-09	1.9E-07	5.9	20:00	12:00
124	isotig01124	AT1G76140	–	Prolyl oligopeptidase family protein	4.0E-41	1.7E-05	2.3E-04	5.8	16:00	4:00
125	isotig02040	AT5G65280	GCL1	GCR2-like 1	0.0	4.3E-12	6.6E-10	5.8	16:00	8:00
126	HI9HAF203DR7YA	AT1G76140	–	Prolyl oligopeptidase family protein	0.0	1.7E-05	2.3E-04	5.7	16:00	4:00
127	isotig06531	AT1G76940	–	RNA-binding (RRM/RBD/RNP motifs) family protein	1.0E-25	6.8E-10	3.2E-08	5.7	16:00	8:00
128	Shoot-012-36_007_D05	–	–	–	–	1.1E-10	8.8E-09	5.7	12:00	24:00
129	HI9HAF203C7O97	AT5G02810	PRR7	pseudo-response regulator 7	4.0E-39	3.1E-09	1.3E-07	5.6	16:00	4:00
130	SSH24-4-29_010_E04	–	–	–	–	1.4E-06	2.5E-05	5.6	24:00	12:00
131	isotig02108	AT5G40390	SIP1	Raffinose synthase family protein	0.0	8.8E-06	1.5E-04	5.6	12:00	20:00
132	Shoot-048-89	AT4G21960	PRXR1	Peroxidase superfamily protein	0.0	5.8E-03	4.3E-02	5.6	16:00	24:00
133	HI9HAF203DMYYA	AT1G20380	–	Prolyl oligopeptidase family protein	2.9E-44	1.2E-05	1.9E-04	5.5	16:00	4:00
134	HI9HAF203DI21I	–	–	–	–	1.2E-08	6.6E-07	5.5	20:00	8:00
135	isotig06700	AT4G39700	–	Heavy metal transport/detoxification superfamily protein	3.0E-11	9.8E-11	8.8E-09	5.5	8:00	16:00
136	HI9HAF203C2WNO	AT5G60100	PRR3	pseudo-response regulator 3	9.0E-42	3.6E-09	1.9E-07	5.4	20:00	4:00
137	isotig04566	AT3G43660	–	Vacuolar iron transporter (VIT) family protein	2.0E-40	1.5E-10	1.2E-08	5.4	8:00	20:00
138	isotig04175	–	–	–	–	5.0E-07	1.4E-05	5.4	12:00	20:00
139	isotig05762	AT2G45560	CYP76C1	cytochrome P450, family 76, subfamily C, polypeptide 1	0.0	2.7E-11	3.1E-09	5.3	16:00	8:00
140	HI9HAF202BS1QV	AT4G25420	GA5	2-oxoglutarate (2OG) and Fe(II)-dependent oxygenase superfamily protein	0.0	1.2E-06	2.1E-05	5.3	8:00	20:00
141	isotig06594	–	–	–	–	4.7E-11	7.7E-09	5.3	12:00	24:00
142	HI9HAF202B99QE	AT5G13930	CHS	Chalcone and stilbene synthase family protein	7.0E-45	1.4E-03	1.4E-02	5.2	16:00	4:00
143	HI9HAF202CLAJI	AT3G12500	ATHCHIB	basic chitinase	0.0	3.8E-07	9.7E-06	5.2	16:00	8:00
144	isotig00661	–	–	–	–	1.6E-07	3.2E-06	5.2	16:00	8:00
145	HI9HAF202BZ69E	–	–	–	–	2.3E-09	1.3E-07	5.2	20:00	8:00
146	isotig06728	–	–	–	–	3.9E-04	4.6E-03	5.2	4:00	12:00
147	HI9HAF203C39DV	AT4G10770	ATOPT7	oligopeptide transporter 7	0.0	2.7E-08	1.0E-06	5.2	4:00	16:00
148	HI9HAF203DJEF7	AT5G06530	–	ABC-2 type transporter family protein	0.0	9.1E-12	1.7E-09	5.2	4:00	16:00
149	Shoot-059-71	AT3G45960	ATEXLA3	expansin-like A3	0.0	4.3E-11	5.2E-09	5.2	4:00	16:00
150	isotig00986	AT3G62600	ATERDJ3B	DNAJ heat shock family protein	2.0E-29	4.2E-05	4.9E-04	5.2	12:00	20:00
151	HI9HAF203DAPBQ	AT2G36190	AtcwINV4	cell wall invertase 4	3.0E-42	1.4E-07	3.2E-06	5.1	20:00	8:00
152	HI9HAF203DIEJ2	AT5G61380	TOC1	CCT motif -containing response regulator protein	9.0E-21	9.0E-09	3.6E-07	5.1	20:00	8:00
153	Shoot-044-15	AT5G61380	TOC1	CCT motif -containing response regulator protein	1.0E-24	8.4E-08	2.3E-06	5.1	20:00	8:00
154	isotig01316	AT3G12580	HSP70	heat shock protein 70	0.0	3.8E-12	6.6E-10	5.1	12:00	24:00
155	isotig06156	–	–	–	–	5.4E-08	1.9E-06	5.0	16:00	8:00
156	SSH24-2-06_011_F01	–	–	–	–	5.1E-09	1.9E-07	5.0	16:00	8:00
157	isotig06624	AT4G14830	HSP1	–	7.0E-14	3.7E-09	1.9E-07	5.0	12:00	24:00
158	isotig05100	AT5G09590	MTHSC70-2	mitochondrial HSO70 2	0.0	5.8E-07	1.4E-05	4.9	12:00	24:00
159	isotig05425	AT3G08970	ATERDJ3A	DNAJ heat shock N-terminal domain-containing protein	1.0E-43	6.1E-09	2.0E-07	4.9	12:00	24:00
160	isotig00873	AT3G14200	–	Chaperone DnaJ-domain superfamily protein	1.0E-23	4.1E-13	1.4E-10	4.8	12:00	4:00
161	HI9HAF202BTYDK	AT2G38310	PYL4	PYR1-like 4	0.0	6.3E-09	2.0E-07	4.8	16:00	4:00
162	HI9HAF203C1W1J	AT3G11410	ATPP2CA	protein phosphatase 2CA	1.0E-34	1.5E-05	2.2E-04	4.8	16:00	4:00
163	HI9HAF203DKAG0	AT1G75450	CKX5	cytokinin oxidase 5	0.0	6.6E-05	9.4E-04	4.8	16:00	4:00
164	isotig05650	–	–	–	–	2.3E-04	2.7E-03	4.8	20:00	4:00
165	isotig06573	AT2G30870	ATGSTF10	glutathione S-transferase PHI 10	0.0	3.7E-08	1.4E-06	4.8	16:00	8:00
166	Shoot-007-70_011_F09	AT3G62600	ATERDJ3B	DNAJ heat shock family protein	0.0	3.9E-06	6.9E-05	4.8	12:00	20:00
167	HI9HAF202BSJ1B	AT2G34650	PID	Protein kinase superfamily protein	8.0E-43	1.2E-07	3.1E-06	4.7	12:00	4:00
168	isotig05273	AT5G08640	FLS1	flavonol synthase 1	0.0	1.3E-06	2.5E-05	4.6	20:00	4:00
169	Shoot-050-73	AT2G44830	–	Protein kinase superfamily protein	2.0E-26	1.9E-09	1.1E-07	4.6	16:00	8:00
170	isotig02943	AT1G62500	–	Bifunctional inhibitor/lipid-transfer protein/seed storage 2S albumin superfamily protein	8.0E-25	7.3E-07	1.4E-05	4.6	20:00	12:00
171	HI9HAF202BXUX7	–	–	–	–	5.7E-08	1.9E-06	4.6	4:00	16:00
172	Shoot-024-70	AT4G10490	–	2-oxoglutarate (2OG) and Fe(II)-dependent oxygenase superfamily protein	0.0	1.2E-07	3.1E-06	4.6	8:00	16:00
173	Shoot-056-18	AT2G12646	–	PLATZ transcription factor family protein	0.0	3.2E-10	2.3E-08	4.5	12:00	4:00
174	HI9HAF202CJSRE	AT2G38310	PYL4	PYR1-like 4	3.0E-34	3.5E-09	1.9E-07	4.5	16:00	8:00
175	isotig01419	AT2G13610	–	ABC-2 type transporter family protein	0.0	8.1E-14	2.7E-11	4.5	8:00	20:00
176	isotig01631	AT1G25560	TEM1	AP2/B3 transcription factor family protein	0.0	1.9E-10	1.8E-08	4.5	8:00	20:00
177	isotig02051	AT5G64510	TIN1	–	0.0	1.3E-09	6.7E-08	4.5	12:00	24:00
178	isotig04542	–	–	–	–	8.5E-10	5.0E-08	4.4	12:00	4:00
179	Shoot-005-74_004_B10	AT2G12646	–	PLATZ transcription factor family protein	0.0	2.5E-10	1.8E-08	4.4	12:00	4:00
180	Shoot-049-60	–	–	–	–	5.9E-10	3.1E-08	4.4	16:00	4:00
181	HI9HAF203DMPHF	AT1G69830	ATAMY3	alpha-amylase-like 3	0.0	5.0E-09	1.9E-07	4.4	20:00	12:00
182	HI9HAF203CYNP6	AT4G26590	ATOPT5	oligopeptide transporter 5	2.0E-43	1.7E-08	7.3E-07	4.4	4:00	16:00
183	Shoot-017-36_007_D05	AT1G18660	–	zinc finger (C3HC4-type RING finger) family protein	2.0E-40	4.3E-09	1.9E-07	4.4	8:00	20:00
184	isotig05611	AT5G20720	CPN20	chaperonin 20	0.0	9.3E-11	8.7E-09	4.4	12:00	24:00
185	HI9HAF202B8FUT	–	–	–	–	1.5E-10	1.1E-08	4.3	16:00	8:00
186	Shoot-017-90_004_B12	–	–	–	–	7.4E-04	6.3E-03	4.3	20:00	12:00
187	HI9HAF202CBT07	AT5G09970	CYP78A7	cytochrome P450, family 78, subfamily A, polypeptide 7	1.0E-42	2.3E-08	1.0E-06	4.3	12:00	24:00
188	HI9HAF202BYAVK	AT2G29380	HAI3	highly ABA-induced PP2C gene 3	6.0E-17	1.6E-05	2.3E-04	4.2	16:00	4:00
189	HI9HAF202BVW5Y	AT2G45560	CYP76C1	cytochrome P450, family 76, subfamily C, polypeptide 1	1.0E-41	1.3E-05	1.9E-04	4.2	16:00	8:00
190	isotig01056	AT4G03210	XTH9	xyloglucan endotransglucosylase/hydrolase 9	0.0	1.7E-06	2.7E-05	4.2	24:00	12:00
191	isotig00857	AT5G38710	–	Methylenetetrahydrofolate reductase family protein	0.0	3.4E-08	1.1E-06	4.2	4:00	16:00
192	HI9HAF202BV9JR	AT4G10490	–	2-oxoglutarate (2OG) and Fe(II)-dependent oxygenase superfamily protein	0.0	1.0E-06	2.1E-05	4.2	8:00	16:00
193	Shoot-021-70_011_F09	AT5G56300	GAMT2	gibberellic acid methyltransferase 2	3.0E-24	8.2E-05	9.4E-04	4.2	8:00	16:00
194	isotig01143	–	–	–	–	5.5E-13	1.4E-10	4.2	8:00	20:00
195	Shoot-052-12	AT2G40460	–	Major facilitator superfamily protein	0.0	7.7E-11	7.7E-09	4.2	8:00	20:00
196	HI9HAF202CAXIE	AT5G09590	MTHSC70-2	mitochondrial HSO70 2	0.0	8.8E-08	2.3E-06	4.2	12:00	24:00
197	Shoot-039-26	–	–	–	–	9.5E-07	2.1E-05	4.1	12:00	4:00
198	isotig06361	AT4G17490	ATERF6	ethylene responsive element binding factor 6	7.0E-15	1.6E-07	3.2E-06	4.1	16:00	4:00
199	isotig03407	AT1G35140	PHI-1	Phosphate-responsive 1 family protein	0.0	2.3E-11	3.1E-09	4.1	4:00	12:00
200	isotig05759	AT2G27830	–	–	6.0E-17	1.3E-05	1.9E-04	4.1	4:00	12:00

201	HI9HAF203DKAPN	AT5G46050	ATPTR3	peptide transporter 3	0.0	7.4E-11	7.7E-09	4.1	8:00	20:00
202	isotig01364	AT2G35710	-	Nucleotide-diphospho-sugar transferases superfamily protein	0.0	8.2E-10	5.0E-08	4.0	16:00	8:00
203	HI9HAF202BUFP0	AT3G18110	EMB1270	Pentatricopeptide repeat (PPR) superfamily protein	9.0E-41	1.6E-08	7.3E-07	4.0	20:00	8:00
204	HI9HAF202CG9E7	AT2G06050	OPR3	oxophytodienoate-reductase 3	8.0E-24	1.1E-06	2.1E-05	4.0	4:00	12:00
205	HI9HAF203DB8LO	AT2G06050	OPR3	oxophytodienoate-reductase 3	3.0E-24	5.4E-07	1.4E-05	4.0	4:00	12:00
206	SSH24-4-94_012_F12	-	-	-	-	2.6E-10	1.8E-08	4.0	4:00	12:00
207	HI9HAF202BTXTW	AT3G18110	EMB1270	Pentatricopeptide repeat (PPR) superfamily protein	4.1E-44	2.0E-09	1.1E-07	4.0	16:00	12:00
208	isotig00619	AT3G56630	CYP94D2	cytochrome P450, family 94, subfamily D, polypeptide 2	0.0	4.4E-10	2.4E-08	4.0	4:00	20:00
209	Shoot-021-79_014_G10	AT1G75000	-	GNS1/SUR4 membrane protein family	0.0	1.0E-13	2.7E-11	4.0	8:00	20:00
210	isotig06283	AT4G25810	XTR6	xyloglucan endotransglycosylase 6	0.0	4.7E-06	6.9E-05	3.9	4:00	8:00
211	HI9HAF202BRDCF	AT5G57360	ZTL	Galactose oxidase/kelch repeat superfamily protein	5.0E-41	1.7E-09	6.7E-08	3.9	16:00	8:00
212	isotig00965	-	-	-	-	2.8E-05	4.4E-04	3.9	16:00	8:00
213	isotig01109	-	-	-	-	8.5E-11	8.7E-09	3.9	16:00	8:00
214	HI9HAF202CF8CB	AT4G02780	GA1	Terpenoid cyclases/Protein prenyltransferases superfamily protein	6.0E-20	3.7E-04	4.2E-03	3.9	4:00	16:00
215	isotig05161	AT3G45970	ATEXLA1	expansin-like A1	0.0	7.2E-12	1.5E-09	3.9	4:00	16:00
216	HI9HAF202B43TO	AT2G26650	AKT1	K+ transporter 1	0.0	1.6E-10	1.7E-08	3.9	8:00	16:00
217	HI9HAF203CY63L	AT5G46240	KAT1	potassium channel in Arabidopsis thaliana 1	0.0	1.8E-10	1.8E-08	3.9	8:00	16:00
218	isotig04640	AT1G76990	ACR3	ACT domain repeat 3	3.0E-39	2.0E-13	8.3E-11	3.9	8:00	16:00
219	isotig00882	AT1G23740	-	Oxidoreductase, zinc-binding dehydrogenase family protein	0.0	1.2E-03	1.3E-02	3.9	12:00	20:00
220	SSH24-2-25_002_A04	-	-	-	-	1.7E-03	1.8E-02	3.9	12:00	24:00
221	Shoot-023-14	AT3G22840	ELIP1	Chlorophyll A-B binding family protein	1.4E-45	8.8E-05	1.1E-03	3.8	12:00	4:00
222	Shoot-054-22	-	-	-	-	2.0E-08	9.1E-07	3.8	12:00	8:00
223	HI9HAF203C40LU	AT5G38260	-	Protein kinase superfamily protein	4.0E-42	8.5E-08	2.3E-06	3.8	16:00	8:00
224	HI9HAF203C8DWS	AT4G38650	-	Glycosyl hydrolase family 10 protein	6.0E-42	7.0E-09	3.3E-07	3.8	16:00	8:00
225	isotig00856	AT3G30775	ERD5	Methylenetetrahydrofolate reductase family protein	2.0E-17	4.4E-09	1.9E-07	3.8	4:00	16:00
226	HI9HAF202CEBY0	AT5G46240	KAT1	potassium channel in Arabidopsis thaliana 1	0.0	2.9E-10	1.8E-08	3.8	8:00	16:00
227	HI9HAF203C8J9K	AT4G10490	-	2-oxoglutarate (2OG) and Fe(II)-dependent oxygenase superfamily protein	4.2E-45	2.1E-07	6.2E-06	3.8	8:00	16:00
228	HI9HAF203C15CV	AT5G38710	-	Methylenetetrahydrofolate reductase family protein	1.0E-43	6.1E-09	2.0E-07	3.8	8:00	20:00
229	Shoot-046-67	AT2G03140	-	alpha/beta-Hydrolases superfamily protein	3.0E-34	3.9E-04	4.6E-03	3.8	8:00	20:00
230	isotig04710	AT3G07090	-	PPPDE putative thiol peptidase family protein	0.0	6.6E-06	1.1E-04	3.8	12:00	20:00
231	SSH24-8-03_005_C01	-	-	-	-	1.3E-04	1.5E-03	3.8	12:00	20:00
232	isotig04935	AT3G09640	APX2	ascorbate peroxidase 2	0.0	5.3E-09	1.9E-07	3.8	12:00	24:00
233	HI9HAF202CGABV	AT4G32300	SD2-5	S-domain-2 5	2.0E-43	3.5E-07	9.7E-06	3.7	16:00	8:00
234	HI9HAF203DGFEU	AT5G57360	ZTL	Galactose oxidase/kelch repeat superfamily protein	0.0	1.2E-09	6.7E-08	3.7	16:00	8:00
235	isotig05467	AT5G23240	-	DNAJ heat shock N-terminal domain-containing protein	1.0E-16	1.3E-10	8.8E-09	3.7	16:00	8:00
236	Shoot-039-41	-	-	-	-	5.0E-11	7.7E-09	3.7	16:00	8:00
237	SSH24-8-71_013_G09	-	-	-	-	1.3E-11	1.7E-09	3.7	4:00	12:00
238	HI9HAF202CAVXN	AT5G19730	-	Pectin lyase-like superfamily protein	0.0	1.4E-03	1.3E-02	3.7	24:00	12:00
239	SSH24-3-76_008_D10	-	-	-	-	5.0E-07	1.4E-05	3.7	4:00	16:00
240	SSH12-6-57_002_A08	AT5G42760	-	Leucine carboxyl methyltransferase	8.0E-19	9.2E-07	2.1E-05	3.7	8:00	16:00
241	HI9HAF203CY149	AT4G34760	-	SAUR-like auxin-responsive protein family	6.0E-23	4.2E-06	6.9E-05	3.7	8:00	20:00
242	isotig02675	AT2G45570	CYP76C2	cytochrome P450, family 76, subfamily C, polypeptide 2	0.0	4.3E-04	5.0E-03	3.7	8:00	20:00
243	isotig04121	AT5G24120	SIGE	sigma factor E	0.0	3.2E-12	6.6E-10	3.7	8:00	20:00
244	Shoot-006-27_006_C04	AT5G17540	-	HXXXD-type acyl-transferase family protein	1.0E-21	1.7E-08	7.3E-07	3.7	12:00	24:00
245	isotig01574	AT1G27680	APL2	ADPGLC-PPase large subunit	0.0	1.6E-05	2.3E-04	3.6	16:00	4:00
246	isotig00695	AT3G19430	-	late embryogenesis abundant protein-related / LEA protein-related	0.0	1.7E-11	1.7E-09	3.6	16:00	8:00
247	isotig03041	AT5G49120	-	Protein of unknown function (DUF581)	4.0E-14	2.6E-09	1.3E-07	3.6	16:00	8:00
248	isotig03686	AT1G75020	LPAT4	lysophosphatidyl acyltransferase 4	0.0	3.5E-10	2.3E-08	3.6	16:00	8:00
249	isotig04037	AT1G55370	NDF5	NDH-dependent cyclic electron flow 5	2.0E-34	5.5E-09	1.9E-07	3.6	16:00	8:00
250	isotig04246	AT5G57360	ZTL	Galactose oxidase/kelch repeat superfamily protein	1.0E-38	3.1E-09	1.3E-07	3.6	16:00	8:00
251	isotig04335	AT2G48020	-	Major facilitator superfamily protein	0.0	3.5E-08	1.4E-06	3.6	16:00	8:00
252	isotig04536	AT5G57360	ZTL	Galactose oxidase/kelch repeat superfamily protein	0.0	4.9E-09	1.9E-07	3.6	16:00	8:00
253	SSH24-8-92_008_D12	AT1G14520	MIOX1	myo-inositol oxygenase 1	0.0	2.4E-07	6.2E-06	3.6	16:00	8:00
254	HI9HAF203DDZYO	AT3G18110	EMB1270	Pentatricopeptide repeat (PPR) superfamily protein	0.0	2.5E-08	1.0E-06	3.6	20:00	8:00
255	SSH24-3-61_010_E08	AT3G04290	ATLTL1	Li-tolerant lipase 1	0.0	1.2E-06	2.5E-05	3.6	24:00	8:00
256	HI9HAF203C7NW7	AT1G79460	GA2	Terpenoid cyclases/Protein prenyltransferases superfamily protein	5.0E-19	3.5E-03	3.1E-02	3.6	4:00	12:00
257	HI9HAF202CFO1O	AT5G19730	-	Pectin lyase-like superfamily protein	0.0	1.9E-03	1.8E-02	3.6	24:00	12:00
258	HI9HAF202B5INI	-	-	-	-	2.9E-10	1.8E-08	3.6	4:00	16:00
259	isotig00398	AT5G13870	EXGT-A4	xyloglucan endotransglucosylase/hydrolase 5	0.0	4.3E-06	6.9E-05	3.6	8:00	20:00
260	isotig01742	AT4G21990	2-Apr	APS reductase 3	0.0	2.3E-09	1.3E-07	3.6	8:00	20:00
261	isotig02665	AT3G09350	Fes1A	Fes1A	0.0	1.5E-07	3.2E-06	3.6	12:00	20:00
262	isotig06632	AT3G24500	MBF1C	multiprotein bridging factor 1C	0.0	2.2E-08	9.1E-07	3.6	12:00	20:00
263	HI9HAF202B3WGH	AT5G03160	ATP58IPK	homolog of mamallian P58IPK	9.0E-41	2.4E-05	4.0E-04	3.6	12:00	24:00
264	isotig00301	-	-	-	-	8.2E-07	1.8E-05	3.6	12:00	24:00
265	isotig01354	AT5G02500	HSC70-1	heat shock cognate protein 70-1	0.0	1.1E-11	1.7E-09	3.6	12:00	24:00
266	Shoot-004-26_004_B04	AT4G22740	-	glycine-rich protein	3.0E-25	1.8E-07	3.3E-06	3.6	12:00	24:00
267	isotig01805	AT5G28540	BIP1	heat shock protein 70 (Hsp 70) family protein	0.0	5.7E-09	1.9E-07	3.5	12:00	4:00
268	Shoot-050-65	-	-	-	-	8.2E-11	8.7E-09	3.5	16:00	4:00
269	HI9HAF202CAYYO	AT5G57360	ZTL	Galactose oxidase/kelch repeat superfamily protein	0.0	3.5E-09	1.9E-07	3.5	16:00	8:00
270	HI9HAF202CFXZK	AT3G12500	ATHCHIB	basic chitinase	0.0	1.0E-06	2.1E-05	3.5	16:00	8:00
271	HI9HAF203DR2XC	AT5G57360	ZTL	Galactose oxidase/kelch repeat superfamily protein	0.0	8.0E-09	3.3E-07	3.5	16:00	8:00
272	HI9HAF203DBE55	AT2G41480	-	Peroxidase superfamily protein	9.0E-25	5.9E-07	1.4E-05	3.5	20:00	8:00
273	HI9HAF203DOO3U	AT1G71695	-	Peroxidase superfamily protein	4.0E-42	1.9E-07	4.3E-06	3.5	20:00	8:00
274	isotig01057	AT4G03210	XTH9	xyloglucan endotransglucosylase/hydrolase 9	1.0E-39	3.1E-09	1.3E-07	3.5	20:00	8:00
275	isotig03520	-	-	-	-	1.2E-11	1.7E-09	3.5	4:00	12:00
276	HI9HAF202B7HJR	AT3G16857	ARR1	response regulator 1	8.0E-13	2.2E-10	1.8E-08	3.5	4:00	16:00
277	isotig02073	AT3G48990	-	AMP-dependent synthetase and ligase family protein	0.0	2.6E-08	1.0E-06	3.5	4:00	16:00
278	Shoot-020-01_001_A01	AT1G61820	BGLU46	beta glucosidase 46	0.0	5.5E-05	6.4E-04	3.5	4:00	16:00
279	isotig01008	AT5G56300	GAMT2	gibberellic acid methyltransferase 2	0.0	2.3E-05	4.0E-04	3.5	8:00	16:00
280	HI9HAF202B82XC	AT5G38710	-	Methylenetetrahydrofolate reductase family protein	7.0E-42	2.2E-09	1.3E-07	3.5	8:00	20:00
281	HI9HAF202CKRK8	AT5G03555	-	permease, cytosine/purines, uracil, thiamine, allantoin family protein	0.0	2.6E-11	3.1E-09	3.5	8:00	20:00
282	isotig04967	AT3G56290	-	-	7.0E-45	5.6E-11	7.7E-09	3.5	8:00	20:00
283	Shoot-059-56	AT5G16010	-	3-oxo-5-alpha-steroid 4-dehydrogenase family protein	2.0E-36	4.6E-12	6.6E-10	3.5	8:00	20:00
284	Shoot-012-62_012_F08	AT5G11260	HY5	Basic-leucine zipper (bZIP) transcription factor family protein	3.0E-21	3.4E-12	6.6E-10	3.5	12:00	20:00
285	HI9HAF203C8QS4	AT4G30780	-	-	4.0E-42	1.8E-10	1.8E-08	3.4	12:00	4:00
286	HI9HAF203DLCSA	AT3G23990	HSP60	heat shock protein 60	0.0	2.2E-06	6.5E-05	3.4	12:00	4:00
287	isotig01294	AT5G42020	BIP	Heat shock protein 70 (Hsp 70) family protein	0.0	2.1E-07	6.2E-06	3.4	12:00	4:00
288	isotig00868	AT2G26710	BAS1	Cytochrome P450 superfamily protein	0.0	1.6E-11	1.7E-09	3.4	16:00	4:00
289	HI9HAF202BQTSA	AT4G38650	-	Glycosyl hydrolase family 10 protein	3.0E-41	4.2E-09	1.9E-07	3.4	16:00	8:00
290	HI9HAF202CE0PV	AT5G14040	PHT3;1	phosphate transporter 3;1	0.0	1.1E-08	4.3E-07	3.4	16:00	8:00
291	Shoot-010-48	AT5G47390	-	myb-like transcription factor family protein	0.0	8.6E-05	1.1E-03	3.4	20:00	8:00
292	isotig06395	AT2G39540	-	Gibberellin-regulated family protein	7.0E-23	1.8E-06	3.7E-05	3.4	4:00	12:00
293	HI9HAF203DKXQU	AT5G19730	-	Pectin lyase-like superfamily protein	0.0	7.8E-04	8.1E-03	3.4	24:00	12:00
294	Shoot-057-11	AT5G46600	-	Aluminium activated malate transporter family protein	8.0E-27	4.0E-03	3.1E-02	3.4	24:00	12:00
295	isotig06245	-	-	-	-	1.8E-10	1.8E-08	3.4	4:00	16:00
296	isotig06121	-	-	-	-	4.5E-05	6.4E-04	3.4	4:00	20:00
297	HI9HAF203CWVTZ	AT1G01390	-	UDP-Glycosyltransferase superfamily protein	1.0E-41	1.1E-13	2.8E-11	3.4	8:00	20:00
298	isotig04004	AT3G51860	CAX3	cation exchanger 3	0.0	9.9E-05	1.1E-03	3.4	8:00	20:00
299	HI9HAF203DPNWC	AT1G15210	PDR7	pleiotropic drug resistance 7	4.0E-42	1.9E-06	3.7E-05	3.4	12:00	20:00
300	HI9HAF203CXCAO	AT1G14360	ATUTR3	UDP-galactose transporter 3	0.0	2.9E-04	2.7E-03	3.4	12:00	24:00

301	isotig03847	AT1G26770	ATEXPA10	expansin A10	0.0	3.1E-08	1.0E-06	3.4	12:00	24:00
302	isotig06422	AT2G47710	–	Adenine nucleotide alpha hydrolases-like superfamily protein	0.0	7.9E-09	3.3E-07	3.4	12:00	24:00
303	SSH12-7-83_005_C11	AT1G44760	–	Adenine nucleotide alpha hydrolases-like superfamily protein	4.0E-30	5.7E-05	6.4E-04	3.4	12:00	24:00
304	isotig06497	AT3G14067	–	Subtilase family protein	2.0E-40	1.3E-15	1.4E-12	3.3	20:00	4:00
305	HI9HAF202BU4R0	AT1G49380	–	cytochrome c biogenesis protein family	0.0	1.3E-08	6.6E-07	3.3	16:00	8:00
306	HI9HAF202CKUUU	AT4G18250	–	receptor serine/threonine kinase, putative	3.0E-41	5.3E-05	6.4E-04	3.3	16:00	8:00
307	HI9HAF202CLCMA	AT5G57360	ZTL	Galactose oxidase/kelch repeat superfamily protein	0.0	2.5E-09	1.3E-07	3.3	16:00	8:00
308	SSH12-5-35_005_C05	AT1G49380	–	cytochrome c biogenesis protein family	3.0E-30	1.0E-08	4.3E-07	3.3	16:00	8:00
309	HI9HAF202BZIYP	AT3G18110	EMB1270	Pentatricopeptide repeat (PPR) superfamily protein	0.0	2.7E-08	1.0E-06	3.3	20:00	8:00
310	HI9HAF203DROMK	AT1G71695	–	Peroxidase superfamily protein	0.0	4.0E-03	3.1E-02	3.3	20:00	8:00
311	isotig05458	AT1G70000	–	myb-like transcription factor family protein	8.0E-29	1.6E-04	1.8E-03	3.3	20:00	8:00
312	isotig06749	AT5G49920	–	Octicosapeptide/Phox/Bem1p family protein	1.0E-31	1.1E-05	1.8E-04	3.3	4:00	16:00
313	Shoot-046-32	AT4G24540	AGL24	AGAMOUS-like 24	5.0E-32	6.0E-10	3.1E-08	3.3	4:00	16:00
314	HI9HAF203DBVBD	AT4G34760	–	SAUR-like auxin-responsive protein family	6.0E-23	1.8E-06	3.7E-05	3.3	8:00	20:00
315	HI9HAF203DC7VQ	–	–	–	–	1.6E-05	2.3E-04	3.3	8:00	20:00
316	isotig06033	AT4G35100	PIP3	plasma membrane intrinsic protein 3	4.0E-34	8.0E-13	1.4E-10	3.3	8:00	20:00
317	isotig06792	AT1G01420	UGT72B3	UDP-glucosyl transferase 72B3	5.0E-13	7.3E-13	1.4E-10	3.3	8:00	20:00
318	Shoot-055-88	–	–	–	–	1.2E-07	3.1E-06	3.3	8:00	20:00
319	HI9HAF202B4YW1	AT2G26910	PDR4	pleiotropic drug resistance 4	0.0	1.2E-06	2.1E-05	3.3	12:00	20:00
320	HI9HAF202CGCUN	AT3G23990	HSP60	heat shock protein 60	0.0	4.6E-06	6.9E-05	3.3	12:00	24:00
321	HI9HAF203DRD6C	AT3G23990	HSP60	heat shock protein 60	0.0	1.2E-05	1.9E-04	3.3	12:00	24:00
322	HI9HAF202BVBDX	AT2G41190	–	Transmembrane amino acid transporter family protein	0.0	3.8E-09	1.9E-07	3.2	12:00	4:00
323	HI9HAF203DQAN0	AT1G68750	ATPPC4	phosphoenolpyruvate carboxylase 4	0.0	6.3E-07	1.4E-05	3.2	16:00	4:00
324	isotig02760	AT5G20190	–	Tetratricopeptide repeat (TPR)-like superfamily protein	7.0E-35	4.3E-11	5.2E-09	3.2	16:00	4:00
325	HI9HAF203C68XH	AT1G22540	–	Major facilitator superfamily protein	5.6E-45	7.1E-10	3.2E-08	3.2	16:00	8:00
326	HI9HAF203DCBF0	AT5G14040	PHT3;1	phosphate transporter 3;1	0.0	1.7E-08	7.3E-07	3.2	16:00	8:00
327	Shoot-049-08	–	–	–	–	7.1E-11	7.7E-09	3.2	16:00	8:00
328	HI9HAF203DC47B	AT1G71695	–	Peroxidase superfamily protein	0.0	5.6E-03	4.3E-02	3.2	20:00	8:00
329	isotig00855	AT3G54420	ATEP3	homolog of carrot EP3-3 chitinase	5.0E-39	6.3E-04	6.3E-03	3.2	20:00	8:00
330	isotig06315	AT4G19160	–	–	0.0	2.9E-07	8.8E-06	3.2	20:00	8:00
331	HI9HAF202BUXOK	AT1G64660	ATMGL	methionine gamma-lyase	3.0E-18	4.1E-07	1.0E-05	3.2	4:00	12:00
332	HI9HAF202BXWKV	AT3G62660	GATL7	galacturonosyltransferase-like 7	0.0	7.6E-07	1.4E-05	3.2	4:00	12:00
333	Shoot-004-35_005_C05	AT3G10210	–	SEC14 cytosolic factor family protein / phosphoglyceride transfer family protein	8.0E-28	1.7E-06	2.7E-05	3.2	20:00	12:00
334	HI9HAF202BWSJY	AT5G60900	RLK1	receptor-like protein kinase 1	0.0	1.2E-12	6.6E-10	3.2	4:00	16:00
335	HI9HAF203DKXCX	AT1G67710	ARR11	response regulator 11	6.0E-22	2.4E-09	1.3E-07	3.2	4:00	16:00
336	SSH24-5-91_006_C12	AT5G38212	–	Potential natural antisense gene, locus overlaps with AT5G38210	3.0E-12	3.7E-13	1.4E-10	3.2	4:00	16:00
337	HI9HAF203DBT8H	AT1G77120	ADH1	alcohol dehydrogenase 1	0.0	5.3E-10	3.1E-08	3.2	8:00	16:00
338	Shoot-001-71_013_G09	AT4G32000	–	Protein kinase superfamily protein	0.0	1.7E-04	1.8E-03	3.2	8:00	16:00
339	HI9HAF202BS4XF	AT5G03555	–	permease, cytosine/purines, uracil, thiamine, allantoin family protein	2.9E-44	1.2E-11	1.7E-09	3.2	8:00	20:00
340	HI9HAF202CJU90	–	–	–	–	3.8E-05	4.9E-04	3.2	8:00	20:00
341	HI9HAF203DCCSP	AT5G40390	SIP1	Raffinose synthase family protein	3.0E-42	5.1E-06	7.3E-05	3.2	12:00	20:00
342	HI9HAF203DDGDD	AT3G55550	–	Concanavalin A-like lectin protein kinase family protein	0.0	2.5E-08	1.0E-06	3.2	12:00	20:00
343	HI9HAF203DLL9G	AT5G24790	–	Protein of unknown function, DUF599	9.0E-42	1.0E-03	1.2E-02	3.2	12:00	20:00
344	HI9HAF203DNGNA	AT3G55550	–	Concanavalin A-like lectin protein kinase family protein	1.4E-45	3.1E-08	1.0E-06	3.2	12:00	20:00
345	isotig05128	AT1G69530	ATEXPA1	expansin A1	0.0	1.6E-07	3.2E-06	3.2	12:00	24:00
346	HI9HAF202CIX0P	AT3G23990	HSP60	heat shock protein 60	0.0	3.0E-06	6.9E-05	3.1	12:00	4:00
347	isotig00118	AT3G22840	ELIP1	Chlorophyll A-B binding family protein	4.0E-39	4.5E-03	3.5E-02	3.1	12:00	4:00
348	isotig04180	AT3G43120	–	SAUR-like auxin-responsive protein family	2.0E-22	6.0E-09	2.0E-07	3.1	12:00	4:00
349	HI9HAF202BURL0	AT4G18250	–	receptor serine/threonine kinase, putative	2.0E-41	2.5E-05	4.0E-04	3.1	16:00	4:00
350	HI9HAF203DIBLL	AT3G50660	DWF4	Cytochrome P450 superfamily protein	0.0	1.6E-04	1.8E-03	3.1	16:00	4:00
351	HI9HAF202BVD0N	AT1G49380	–	cytochrome c biogenesis protein family	0.0	1.4E-08	6.6E-07	3.1	16:00	8:00
352	HI9HAF202CHAVD	AT3G48530	KING1	SNF1-related protein kinase regulatory subunit gamma 1	1.0E-41	1.2E-09	5.9E-08	3.1	16:00	8:00
353	isotig04982	AT4G31970	CYP82C2	cytochrome P450, family 82, subfamily C, polypeptide 2	8.0E-43	1.1E-03	1.3E-02	3.1	4:00	12:00
354	Shoot-006-19_005_C03	AT4G31970	CYP82C2	cytochrome P450, family 82, subfamily C, polypeptide 2	8.0E-37	5.5E-04	6.1E-03	3.1	4:00	12:00
355	HI9HAF202B8HFI	AT3G10210	–	SEC14 cytosolic factor family protein / phosphoglyceride transfer family protein	0.0	1.5E-05	2.2E-04	3.1	20:00	12:00
356	HI9HAF202CLFKF	–	–	–	–	4.2E-04	4.9E-03	3.1	24:00	12:00
357	HI9HAF203DNZO6	AT4G02780	GA1	Terpenoid cyclases/Protein prenyltransferases superfamily protein	6.0E-20	3.6E-04	4.2E-03	3.1	4:00	16:00
358	Shoot-058-94	–	–	–	–	7.2E-07	1.4E-05	3.1	4:00	16:00
359	SSH24-5-73_002_A10	AT5G01740	–	Nuclear transport factor 2 (NTF2) family protein	2.0E-27	3.1E-09	1.3E-07	3.1	4:00	16:00
360	HI9HAF202B0S1U	AT5G05600	–	2-oxoglutarate (2OG) and Fe(II)-dependent oxygenase superfamily protein	0.0	2.0E-09	1.1E-07	3.1	4:00	20:00
361	HI9HAF203C1GCN	AT5G03555	–	permease, cytosine/purines, uracil, thiamine, allantoin family protein	0.0	6.8E-11	7.7E-09	3.1	8:00	20:00
362	HI9HAF203CYY76	AT2G18010	–	SAUR-like auxin-responsive protein family	4.0E-14	6.6E-05	8.3E-04	3.1	8:00	20:00
363	HI9HAF203DM1OJ	AT3G16520	UGT88A1	UDP-glucosyl transferase 88A1	5.0E-43	2.3E-12	6.6E-10	3.1	8:00	20:00
364	isotig04853	AT4G34350	CLB6	4-hydroxy-3-methylbut-2-enyl diphosphate reductase	6.0E-43	4.1E-13	1.4E-10	3.1	12:00	20:00
365	isotig05870	AT3G14440	NCED3	nine-cis-epoxycarotenoid dioxygenase 3	0.0	4.1E-10	2.4E-08	3.1	12:00	20:00
366	isotig00299	–	–	–	–	3.7E-06	6.9E-05	3.1	12:00	24:00
367	HI9HAF202B5WN1	AT1G78390	NCED9	nine-cis-epoxycarotenoid dioxygenase 9	2.0E-32	1.8E-05	3.0E-04	3.0	12:00	4:00
368	isotig05965	AT5G25280	–	serine-rich protein-related	1.0E-15	3.2E-08	1.0E-06	3.0	16:00	4:00
369	SSH12-5-32_016_H04	AT3G15850	FAD5	fatty acid desaturase 5	4.0E-26	2.1E-13	8.6E-11	3.0	16:00	4:00
370	HI9HAF203C83LV	AT2G20790	–	clathrin adaptor complexes medium subunit family protein	0.0	1.9E-08	7.3E-07	3.0	16:00	8:00
371	HI9HAF203DRR2U	AT1G60590	–	Pectin lyase-like superfamily protein	1.4E-45	2.9E-04	2.7E-03	3.0	16:00	8:00
372	SSH24-1-89_002_A12	AT4G33625	–	–	9.0E-24	5.9E-09	2.0E-07	3.0	16:00	8:00
373	isotig05093	AT5G48480	–	Lactoylglutathione lyase / glyoxalase I family protein	8.0E-21	2.5E-07	6.2E-06	3.0	20:00	8:00
374	HI9HAF203C1O6D	AT2G44500	–	O-fucosyltransferase family protein	0.0	8.2E-09	3.3E-07	3.0	4:00	12:00
375	isotig04273	AT5G64260	EXL2	EXORDIUM like 2	0.0	1.9E-09	1.1E-07	3.0	4:00	12:00
376	SSH24-3-15_014_G02	–	–	–	–	1.1E-10	8.8E-09	3.0	4:00	12:00
377	isotig06387	–	–	–	–	1.3E-04	1.5E-03	3.0	20:00	12:00
378	SSH12-9-28_008_D04	–	–	–	–	1.7E-04	1.8E-03	3.0	20:00	12:00
379	HI9HAF203DI5XB	AT5G19730	–	Pectin lyase-like superfamily protein	0.0	1.2E-03	1.3E-02	3.0	24:00	12:00
380	HI9HAF203DQ59H	AT1G10550	XTH33	xyloglucan:xyloglucosyl transferase 33	1.4E-45	7.4E-10	3.9E-08	3.0	24:00	12:00
381	Shoot-012-28_008_D04	AT1G19510	ATRL5	RAD-like 5	5.0E-13	7.8E-04	8.1E-03	3.0	24:00	12:00
382	Shoot-045-43	–	–	–	–	1.6E-06	2.7E-05	3.0	24:00	12:00
383	HI9HAF203DIAPG	AT1G67710	ARR11	response regulator 11	9.0E-29	3.7E-10	2.4E-08	3.0	4:00	16:00
384	isotig02964	AT4G16260	–	Glycosyl hydrolase superfamily protein	0.0	1.3E-03	1.3E-02	3.0	4:00	16:00
385	isotig04228	AT5G01740	–	Nuclear transport factor 2 (NTF2) family protein	1.0E-28	1.8E-09	1.1E-07	3.0	4:00	16:00
386	isotig01416	AT4G05200	CRK25	cysteine-rich RLK (RECEPTOR-like protein kinase) 25	0.0	2.3E-10	1.8E-08	3.0	8:00	16:00
387	isotig03941	AT5G67360	ARA12	Subtilase family protein	0.0	7.0E-10	3.2E-08	3.0	8:00	16:00
388	isotig04068	–	–	–	–	3.6E-10	2.3E-08	3.0	8:00	16:00
389	HI9HAF202B8ET8	AT3G51895	SULTR3;1	sulfate transporter 3;1	0.0	2.9E-11	3.1E-09	3.0	8:00	20:00
390	HI9HAF202BWF9P	AT1G14360	ATUTR3	UDP-galactose transporter 3	0.0	3.3E-04	2.8E-03	3.0	12:00	24:00
391	isotig06099	AT2G47180	AtGolS1	galactinol synthase 1	0.0	5.4E-08	1.9E-06	3.0	12:00	24:00
392	HI9HAF202B0AVA	AT3G23990	HSP60	heat shock protein 60	0.0	2.0E-07	6.2E-06	2.9	12:00	4:00
393	HI9HAF203CZCIQ	AT3G25230	ROF1	rotamase FKBP 1	5.0E-41	7.3E-08	1.9E-06	2.9	12:00	4:00
394	HI9HAF203DE338	AT3G25230	ROF1	rotamase FKBP 1	0.0	3.2E-08	1.0E-06	2.9	12:00	4:00
395	isotig00300	–	–	–	–	3.0E-05	4.9E-04	2.9	12:00	4:00
396	isotig04760	AT2G37970	SOUL-1	SOUL heme-binding family protein	0.0	2.5E-14	1.1E-11	2.9	12:00	4:00
397	HI9HAF202BS5O6	AT2G18150	–	Peroxidase superfamily protein	2.0E-16	2.2E-10	1.8E-08	2.9	16:00	4:00
398	isotig02233	AT3G15850	FAD5	fatty acid desaturase 5	0.0	4.5E-12	6.6E-10	2.9	16:00	4:00
399	SSH24-8-75_006_C10	AT3G59730	–	Concanavalin A-like lectin protein kinase family protein	3.0E-14	4.9E-04	5.0E-03	2.9	4:00	8:00
400	HI9HAF202CMB2V	AT4G15560	CLA1	Deoxyxylulose-5-phosphate synthase	2.0E-43	1.3E-11	1.7E-09	2.9	16:00	8:00

401	HI9HAF203DJS7S	AT4G15560	CLA1	Deoxyxylulose-5-phosphate synthase	0.0	1.6E-11	1.7E-09	2.9	16:00	8:00
402	isotig05097	AT3G25600	−	Calcium-binding EF-hand family protein	7.0E-22	1.2E-06	2.1E-05	2.9	16:00	8:00
403	Shoot-030-40	−	−	−	−	1.9E-06	3.7E-05	2.9	16:00	8:00
404	isotig04031	−	−	−	−	7.5E-06	1.1E-04	2.9	20:00	8:00
405	HI9HAF203C9FZX	AT1G64660	ATMGL	methionine gamma-lyase	0.0	3.1E-08	1.0E-06	2.9	4:00	12:00
406	isotig03530	AT1G69530	ATEXPA1	expansin A1	0.0	1.8E-08	7.3E-07	2.9	4:00	12:00
407	Shoot-029-52	−	−	−	−	3.1E-03	2.9E-02	2.9	20:00	12:00
408	HI9HAF202CEMXR	AT2G42980	−	Eukaryotic aspartyl protease family protein	0.0	1.6E-11	1.7E-09	2.9	4:00	16:00
409	Shoot-029-15	−	−	−	−	1.5E-07	3.2E-06	2.9	4:00	16:00
410	HI9HAF203C89CZ	AT1G76990	ACR3	ACT domain repeat 3	0.0	5.7E-13	1.4E-10	2.9	8:00	16:00
411	isotig06186	−	−	−	−	9.2E-07	2.1E-05	2.9	8:00	16:00
412	isotig00765	AT4G27250	−	NAD(P)-binding Rossmann-fold superfamily protein	1.0E-41	2.9E-06	6.9E-05	2.9	4:00	20:00
413	HI9HAF203C17EN	AT3G57630	−	exostosin family protein	0.0	2.9E-11	3.5E-09	2.9	8:00	20:00
414	HI9HAF203C8NXR	AT3G57630	−	exostosin family protein	0.0	1.2E-10	8.8E-09	2.9	8:00	20:00
415	isotig01511	AT2G16570	ATASE	GLN phosphoribosyl pyrophosphate amidotransferase 1	0.0	4.0E-09	1.9E-07	2.9	8:00	20:00
416	isotig02115	AT5G22090	−	Protein of unknown function (DUF3049)	3.0E-15	4.2E-12	6.6E-10	2.9	8:00	20:00
417	isotig06630	AT5G16010	−	3-oxo-5-alpha-steroid 4-dehydrogenase family protein	5.0E-33	1.4E-12	6.6E-10	2.9	8:00	20:00
418	isotig03850	AT5G01410	PDX1	Aldolase-type TIM barrel family protein	0.0	7.8E-06	1.3E-04	2.9	12:00	20:00
419	Shoot-050-47	AT3G58680	MBF1B	multiprotein bridging factor 1B	1.0E-42	2.0E-08	7.3E-07	2.9	12:00	20:00
420	HI9HAF202CFEVF	AT1G14360	ATUTR3	UDP-galactose transporter 3	0.0	5.5E-04	6.1E-03	2.9	12:00	24:00
421	isotig02916	AT2G45380	−	−	3.0E-27	9.9E-08	2.6E-06	2.9	12:00	24:00
422	HI9HAF202BUGL9	AT5G15450	APG6	casein lytic proteinase B3	0.0	1.4E-05	2.2E-04	2.8	12:00	4:00
423	HI9HAF202CD4C3	AT2G25140	HSP98.7	casein lytic proteinase B4	0.0	5.2E-05	6.4E-04	2.8	12:00	4:00
424	isotig01223	AT4G24190	SHD	Chaperone protein htpG family protein	0.0	2.0E-07	6.2E-06	2.8	12:00	4:00
425	isotig02484	AT5G19875	−	−	3.0E-22	5.5E-11	7.7E-09	2.8	12:00	4:00
426	HI9HAF202CI9W9	AT2G40840	DPE2	disproportionating enzyme 2	1.4E-45	7.3E-10	3.2E-08	2.8	16:00	4:00
427	HI9HAF202CKXRQ	−	−	−	−	2.6E-06	6.5E-05	2.8	16:00	4:00
428	isotig03291	AT5G05340	−	Peroxidase superfamily protein	0.0	1.4E-06	2.5E-05	2.8	16:00	4:00
429	isotig03774	AT2G15890	MEE14	maternal effect embryo arrest 14	8.0E-31	1.5E-14	1.1E-11	2.8	16:00	4:00
430	HI9HAF202B74YU	AT1G49380	−	cytochrome c biogenesis protein family	0.0	6.2E-08	1.9E-06	2.8	16:00	8:00
431	isotig05171	AT2G47770	ATTSPO	TSPO(outer membrane tryptophan-rich sensory protein)-related	4.0E-20	4.7E-10	2.4E-08	2.8	16:00	8:00
432	isotig06359	AT4G33625	−	−	0.0	9.1E-10	5.0E-08	2.8	16:00	8:00
433	Shoot-047-37	AT2G28080	−	UDP-Glycosyltransferase superfamily protein	8.0E-20	1.6E-10	1.2E-08	2.8	16:00	8:00
434	HI9HAF203DOJ1Z	AT1G10760	SEX1	Pyruvate phosphate dikinase, PEP/pyruvate binding domain	0.0	4.8E-09	1.9E-07	2.8	20:00	8:00
435	HI9HAF203DQLPJ	AT1G10760	SEX1	Pyruvate phosphate dikinase, PEP/pyruvate binding domain	3.0E-42	5.4E-09	1.9E-07	2.8	20:00	8:00
436	isotig01977	AT2G42850	CYP718	cytochrome P450, family 718	0.0	8.9E-06	1.5E-04	2.8	20:00	8:00
437	isotig02747	−	−	−	−	3.2E-04	2.8E-03	2.8	20:00	8:00
438	Shoot-045-82	AT5G47390	−	myb-like transcription factor family protein	1.4E-45	1.0E-04	1.1E-03	2.8	20:00	8:00
439	HI9HAF202B60K5	AT1G64660	ATMGL	methionine gamma-lyase	0.0	7.3E-08	1.9E-06	2.8	4:00	12:00
440	HI9HAF202BRGJH	AT1G64660	ATMGL	methionine gamma-lyase	5.0E-23	1.6E-07	3.2E-06	2.8	4:00	12:00
441	HI9HAF203C1K4S	AT2G44500	−	O-fucosyltransferase family protein	1.4E-45	8.4E-09	3.6E-07	2.8	4:00	12:00
442	HI9HAF203DC217	AT1G64660	ATMGL	methionine gamma-lyase	0.0	1.6E-08	7.3E-07	2.8	4:00	12:00
443	HI9HAF203DHGH8	AT1G64660	ATMGL	methionine gamma-lyase	8.0E-25	2.3E-08	1.0E-06	2.8	4:00	12:00
444	Shoot-059-11	−	−	−	−	6.4E-03	4.9E-02	2.8	4:00	12:00
445	Shoot-045-28	AT1G78990	−	HXXXD-type acyl-transferase family protein	0.0	1.7E-04	1.8E-03	2.8	8:00	12:00
446	HI9HAF203DJGMW	AT4G04320	−	malonyl-CoA decarboxylase family protein	0.0	4.7E-08	1.4E-06	2.8	20:00	12:00
447	HI9HAF203DO77V	AT3G47800	−	Galactose mutarotase-like superfamily protein	0.0	1.5E-05	2.3E-04	2.8	20:00	12:00
448	HI9HAF203DH875	AT5G05340	−	Peroxidase superfamily protein	3.0E-32	6.4E-13	1.4E-10	2.8	4:00	16:00
449	isotig04852	−	−	−	−	4.4E-08	1.4E-06	2.8	4:00	16:00
450	isotig06738	AT2G19130	−	S-locus lectin protein kinase family protein	1.0E-28	8.7E-12	1.7E-09	2.8	4:00	16:00
451	Shoot-027-76	AT3G50950	ZAR1	HOPZ-ACTIVATED RESISTANCE 1	7.0E-14	8.7E-11	8.7E-09	2.8	4:00	16:00
452	HI9HAF202BWLCD	AT4G02900	−	ERD (early-responsive to dehydration stress) family protein	0.0	5.2E-08	1.5E-06	2.8	8:00	16:00
453	HI9HAF202CA5QQ	AT1G66340	ETR1	Signal transduction histidine kinase, hybrid-type, ethylene sensor	4.0E-27	7.6E-11	7.7E-09	2.8	8:00	16:00
454	Shoot-009-64_016_H08	AT4G09720	ATRABG3A	RAB GTPase homolog G3A	0.0	5.9E-06	9.5E-05	2.8	24:00	16:00
455	Shoot-046-69	−	−	−	−	9.9E-06	1.5E-04	2.8	4:00	20:00
456	HI9HAF202B9FSD	AT3G57630	−	exostosin family protein	0.0	5.8E-11	7.7E-09	2.8	8:00	20:00
457	HI9HAF202BXWDV	AT1G74950	JAZ2	TIFY domain/Divergent CCT motif family protein	4.0E-16	3.3E-04	2.8E-03	2.8	8:00	20:00
458	HI9HAF203C8SUM	AT1G26780	MYB117	myb domain protein 117	0.0	9.8E-04	1.1E-02	2.8	8:00	20:00
459	HI9HAF203CYA96	−	−	−	−	9.1E-08	2.3E-06	2.8	8:00	20:00
460	SSH12-8-47_014_G06	−	−	−	−	7.4E-06	1.1E-04	2.8	4:00	24:00
461	HI9HAF203DDOCS	AT1G76690	OPR2	12-oxophytodienoate reductase 2	0.0	2.7E-04	2.7E-03	2.8	12:00	24:00
462	HI9HAF202B5TPI	AT5G15450	APG6	casein lytic proteinase B3	2.0E-42	2.3E-05	4.0E-04	2.7	12:00	4:00
463	HI9HAF202BRY0E	AT2G25140	HSP98.7	casein lytic proteinase B4	0.0	8.8E-05	1.1E-03	2.7	12:00	4:00
464	HI9HAF202CAZ5B	AT3G24120	−	Homeodomain-like superfamily protein	1.0E-42	4.2E-11	5.2E-09	2.7	12:00	4:00
465	HI9HAF203DH1YZ	AT3G23990	HSP60	heat shock protein 60	0.0	2.4E-07	6.2E-06	2.7	12:00	4:00
466	HI9HAF202CHFG2	AT1G02460	−	Pectin lyase-like superfamily protein	9.8E-45	2.8E-03	2.4E-02	2.7	16:00	4:00
467	HI9HAF203DIIVI	AT3G15510	ATNAC2	NAC domain containing protein 2	0.0	2.8E-05	4.4E-04	2.7	16:00	4:00
468	Shoot-003-47_014_G06	−	−	−	−	1.3E-11	1.7E-09	2.7	16:00	4:00
469	Shoot-050-49	AT5G52420	−	−	4.0E-42	3.4E-09	1.9E-07	2.7	16:00	4:00
470	Shoot-006-47_014_G06	AT1G73340	−	Cytochrome P450 superfamily protein	1.4E-45	3.0E-10	2.3E-08	2.7	16:00	8:00
471	Shoot-048-54	AT2G44830	−	Protein kinase superfamily protein	0.0	8.0E-10	5.0E-08	2.7	16:00	8:00
472	HI9HAF202B4NVC	AT1G10760	SEX1	Pyruvate phosphate dikinase, PEP/pyruvate binding domain	0.0	4.1E-10	2.4E-08	2.7	20:00	8:00
473	HI9HAF202B6I6Z	AT1G10760	SEX1	Pyruvate phosphate dikinase, PEP/pyruvate binding domain	0.0	1.7E-09	6.7E-08	2.7	20:00	8:00
474	isotig03907	AT1G10760	SEX1	Pyruvate phosphate dikinase, PEP/pyruvate binding domain	0.0	1.5E-08	7.3E-07	2.7	20:00	8:00
475	HI9HAF202B0U35	−	−	−	−	1.8E-08	7.3E-07	2.7	4:00	12:00
476	HI9HAF203DMC7D	AT5G49720	ATGH9A1	glycosyl hydrolase 9A1	0.0	3.7E-07	9.7E-06	2.7	4:00	12:00
477	Shoot-023-66	AT3G59480	−	pfkB-like carbohydrate kinase family protein	2.0E-36	5.3E-03	4.3E-02	2.7	16:00	12:00
478	isotig04997	AT4G27450	−	Aluminium induced protein with YGL and LRDR motifs	0.0	5.0E-06	7.3E-05	2.7	20:00	12:00
479	HI9HAF202B325D	AT2G42980	−	Eukaryotic aspartyl protease family protein	0.0	6.5E-12	1.1E-09	2.7	4:00	16:00
480	HI9HAF202B4Q1X	AT5G05340	−	Peroxidase superfamily protein	9.0E-38	1.2E-13	2.8E-11	2.7	4:00	16:00
481	HI9HAF202CA696	AT2G19130	−	S-locus lectin protein kinase family protein	0.0	8.0E-13	1.7E-10	2.7	4:00	16:00
482	isotig03266	AT5G14700	−	NAD(P)-binding Rossmann-fold superfamily protein	0.0	8.0E-11	8.7E-09	2.7	4:00	16:00
483	HI9HAF203DCF70	AT2G21220	−	SAUR-like auxin-responsive protein family	6.0E-20	3.9E-05	4.9E-04	2.7	8:00	16:00
484	HI9HAF203DMDEZ	AT5G05340	−	Peroxidase superfamily protein	4.0E-38	2.1E-10	1.8E-08	2.7	8:00	16:00
485	Shoot-025-13	AT3G20660	AtOCT4	organic cation/carnitine transporter4	1.4E-45	4.3E-10	2.4E-08	2.7	8:00	16:00
486	HI9HAF203C165B	AT1G59870	PEN3	ABC-2 and Plant PDR ABC-type transporter family protein	2.0E-44	6.5E-05	8.3E-04	2.7	24:00	16:00
487	HI9HAF203DQGN7	−	−	−	−	2.3E-04	2.7E-03	2.7	8:00	20:00
488	isotig04637	AT1G78370	ATGSTU20	glutathione S-transferase TAU 20	0.0	2.2E-11	2.5E-09	2.7	8:00	20:00
489	isotig06012	AT5G03555	−	permease, cytosine/purines, uracil, thiamine, allantoin family protein	0.0	1.9E-10	1.8E-08	2.7	8:00	20:00
490	HI9HAF202B80AD	AT4G29130	ATHXK1	hexokinase 1	0.0	1.7E-05	2.3E-04	2.7	12:00	20:00
491	isotig00631	AT5G41040	−	HXXXD-type acyl-transferase family protein	0.0	1.1E-03	1.3E-02	2.7	12:00	24:00
492	isotig01277	AT5G56030	HSP81-2	heat shock protein 81-2	0.0	2.1E-10	1.8E-08	2.7	12:00	24:00
493	SSH12-2-61_010_E08	−	−	−	−	7.0E-04	6.3E-03	2.7	12:00	24:00
494	HI9HAF203C9D5H	AT4G16660	−	heat shock protein 70 (Hsp 70) family protein	0.0	2.0E-05	3.4E-04	2.6	12:00	4:00
495	isotig05450	AT1G27330	−	Ribosome associated membrane protein RAMP4	1.0E-16	4.9E-04	5.0E-03	2.6	12:00	4:00
496	SSH24-1-05_009_E01	−	−	−	−	2.1E-07	6.2E-06	2.6	12:00	4:00
497	HI9HAF203C0AMS	AT2G27610	−	Tetratricopeptide repeat (TPR)-like superfamily protein	0.0	3.6E-10	2.3E-08	2.6	16:00	4:00
498	isotig04330	−	−	−	−	1.3E-10	8.8E-09	2.6	16:00	4:00
499	SSH24-2-01_001_A01	−	−	−	−	4.8E-12	6.6E-10	2.6	16:00	4:00
500	Shoot-045-36	−	−	−	−	9.1E-08	2.3E-06	2.6	4:00	8:00

501	HI9HAF202BUCFB	AT1G22400	UGT85A1	UDP-Glycosyltransferase superfamily protein	2.0E-14	4.3E-08	1.4E-06	2.6	16:00	8:00
502	HI9HAF202BW9MV	AT4G18750	DOT4	Pentatricopeptide repeat (PPR) superfamily protein	2.0E-42	6.4E-06	9.7E-05	2.6	16:00	8:00
503	HI9HAF202BZ3FJ	AT3G23330	−	Tetratricopeptide repeat (TPR)-like superfamily protein	0.0	5.7E-09	2.0E-07	2.6	16:00	8:00
504	HI9HAF203DFH8Q	AT4G13180	−	NAD(P)-binding Rossmann-fold superfamily protein	0.0	1.9E-08	7.3E-07	2.6	16:00	8:00
505	HI9HAF203DKPZT	AT4G35290	GLUR2	glutamate receptor 2	0.0	2.7E-09	1.3E-07	2.6	16:00	8:00
506	HI9HAF203DLH96	AT3G53360	−	Tetratricopeptide repeat (TPR)-like superfamily protein	5.0E-42	1.0E-09	5.9E-08	2.6	16:00	8:00
507	isotig03141	AT1G77490	TAPX	thylakoidal ascorbate peroxidase	0.0	3.8E-08	1.4E-06	2.6	16:00	8:00
508	isotig04532	AT5G53190	SWEET3	Nodulin MtN3 family protein	8.0E-39	1.6E-04	1.8E-03	2.6	16:00	8:00
509	Shoot-017-21_009_E03	−	−	−	−	5.2E-13	1.4E-10	2.6	16:00	8:00
510	Shoot-058-64	−	−	−	−	7.1E-12	1.1E-09	2.6	16:00	8:00
511	HI9HAF203DIROI	AT4G31940	CYP82C4	cytochrome P450, family 82, subfamily C, polypeptide 4	0.0	3.2E-04	2.8E-03	2.6	4:00	12:00
512	isotig01100	AT1G72360	HRE1	Integrase-type DNA-binding superfamily protein	7.0E-18	1.7E-05	2.3E-04	2.6	4:00	12:00
513	isotig06238	−	−	−	−	6.2E-07	1.4E-05	2.6	4:00	12:00
514	Shoot-020-39_013_G05	AT5G57530	XTH12	xyloglucan endotransglucosylase/hydrolase 12	0.0	5.8E-08	1.9E-06	2.6	4:00	12:00
515	isotig05621	AT4G10260	−	pfkB-like carbohydrate kinase family protein	0.0	4.8E-03	3.8E-02	2.6	16:00	12:00
516	HI9HAF202B6MT8	AT4G17690	−	Peroxidase superfamily protein	0.0	2.5E-05	4.0E-04	2.6	20:00	12:00
517	isotig00990	AT1G28220	ATPUP3	purine permease 3	0.0	1.8E-07	3.3E-06	2.6	20:00	12:00
518	SSH24-4-21_009_E03	−	−	−	−	3.2E-03	2.9E-02	2.6	24:00	12:00
519	HI9HAF202B0AKD	AT3G21620	−	ERD (early-responsive to dehydration stress) family protein	0.0	2.6E-07	6.8E-06	2.6	4:00	16:00
520	HI9HAF203DISBK	AT5G60900	RLK1	receptor-like protein kinase 1	0.0	1.2E-11	1.7E-09	2.6	4:00	16:00
521	isotig03482	AT5G08640	FLS1	flavonol synthase 1	0.0	6.8E-04	6.3E-03	2.6	4:00	16:00
522	isotig04066	AT3G54850	ATPUB14	plant U-box 14	0.0	3.8E-12	6.6E-10	2.6	4:00	16:00
523	isotig04684	AT5G19140	ATAILP1	Aluminium induced protein with YGL and LRDR motifs	0.0	2.2E-04	2.4E-03	2.6	4:00	16:00
524	isotig06134	−	−	−	−	2.1E-05	3.4E-04	2.6	4:00	16:00
525	Shoot-001-50_003_B07	AT1G15780	−	−	3.0E-19	1.0E-05	1.5E-04	2.6	4:00	16:00
526	Shoot-030-15	−	−	−	−	6.5E-06	1.1E-04	2.6	4:00	16:00
527	Shoot-059-64	−	−	−	−	3.5E-05	4.9E-04	2.6	4:00	16:00
528	HI9HAF203DKT1M	AT5G42760	−	Leucine carboxyl methyltransferase	0.0	1.2E-05	1.9E-04	2.6	8:00	16:00
529	HI9HAF203DRHQF	AT5G08380	AtAGAL1	alpha-galactosidase 1	0.0	2.4E-05	4.0E-04	2.6	8:00	16:00
530	isotig00267	AT1G21460	SWEET1	Nodulin MtN3 family protein	0.0	3.6E-03	3.1E-02	2.6	8:00	16:00
531	isotig01958	AT4G38060	−	−	1.0E-10	2.9E-11	3.1E-09	2.6	8:00	16:00
532	SSH12-8-35_005_C05	−	−	−	−	7.5E-06	1.2E-04	2.6	8:00	16:00
533	HI9HAF202B13J6	AT5G56220	−	P-loop containing nucleoside triphosphate hydrolases superfamily protein	1.4E-45	1.7E-03	1.4E-02	2.6	24:00	16:00
534	HI9HAF203C309P	AT1G71695	−	Peroxidase superfamily protein	5.0E-37	7.7E-08	2.3E-06	2.6	24:00	16:00
535	Shoot-003-41_002_A06	AT2G42990	−	GDSL-like Lipase/Acylhydrolase superfamily protein	3.0E-36	4.1E-05	4.9E-04	2.6	24:00	16:00
536	SSH24-8-26_004_B04	AT1G59870	PEN3	ABC-2 and Plant PDR ABC-type transporter family protein	3.0E-26	1.3E-03	1.3E-02	2.6	24:00	16:00
537	HI9HAF202BWYA2	AT2G32950	COP1	Transducin/WD40 repeat-like superfamily protein	0.0	2.4E-14	1.1E-11	2.6	8:00	20:00
538	HI9HAF203DHF0N	AT2G32950	COP1	Transducin/WD40 repeat-like superfamily protein	0.0	3.1E-13	1.4E-10	2.6	8:00	20:00
539	HI9HAF203DQSIW	AT2G32950	COP1	Transducin/WD40 repeat-like superfamily protein	0.0	5.8E-13	1.4E-10	2.6	8:00	20:00
540	isotig03350	AT4G34350	CLB6	4-hydroxy-3-methylbut-2-enyl diphosphate reductase	0.0	1.0E-13	2.7E-11	2.6	8:00	20:00
541	HI9HAF202CKKIQ	AT3G43120	−	SAUR-like auxin-responsive protein family	5.0E-25	5.1E-09	1.9E-07	2.6	12:00	20:00
542	HI9HAF202CMH87	AT5G15250	FTSH6	FTSH protease 6	0.0	4.7E-11	5.2E-09	2.6	12:00	20:00
543	isotig02293	AT1G74310	ATHSP101	heat shock protein 101	0.0	9.1E-07	2.1E-05	2.6	12:00	20:00
544	Shoot-048-02	AT4G28670	−	Protein kinase family protein with domain of unknown function (DUF26)	2.0E-15	5.9E-08	1.9E-06	2.6	12:00	20:00
545	isotig06252	AT3G23990	HSP60	heat shock protein 60	0.0	4.1E-07	1.0E-05	2.6	12:00	24:00
546	Shoot-003-39_013_G05	AT5G51260	−	HAD superfamily, subfamily IIIB acid phosphatase	0.0	3.5E-05	4.9E-04	2.6	12:00	24:00
547	Shoot-013-23_013_G03	AT2G01770	VIT1	vacuolar iron transporter 1	0.0	3.8E-04	4.2E-03	2.6	12:00	24:00
548	HI9HAF202B7X98	AT1G64390	AtGH9C2	glycosyl hydrolase 9C2	0.0	4.3E-06	6.9E-05	2.5	12:00	4:00
549	HI9HAF202CCNWM	AT1G17870	ATEGY3	ethylene-dependent gravitropism-deficient and yellow-green-like 3	7.0E-44	2.2E-07	6.2E-06	2.5	12:00	4:00
550	isotig02203	AT1G64390	AtGH9C2	glycosyl hydrolase 9C2	0.0	4.5E-06	6.9E-05	2.5	12:00	4:00
551	isotig06339	AT4G24220	VEP1	NAD(P)-binding Rossmann-fold superfamily protein	0.0	2.4E-08	1.0E-06	2.5	12:00	4:00
552	Shoot-002-88_015_H11	AT4G24220	VEP1	NAD(P)-binding Rossmann-fold superfamily protein	3.0E-35	1.0E-06	2.1E-05	2.5	12:00	4:00
553	SSH24-2-38_011_F05	−	−	−	−	1.2E-03	1.3E-02	2.5	12:00	4:00
554	HI9HAF202B330Q	AT2G40840	DPE2	disproportionating enzyme 2	1.4E-45	3.9E-09	1.9E-07	2.5	16:00	4:00
555	HI9HAF202CA2WB	AT4G03420	−	Protein of unknown function (DUF789)	9.8E-45	1.8E-06	3.7E-05	2.5	16:00	4:00
556	isotig03567	AT5G14370	−	CCT motif family protein	9.0E-22	4.9E-12	6.6E-10	2.5	16:00	4:00
557	isotig04855	AT2G34860	EDA3	DnaJ/Hsp40 cysteine-rich domain superfamily protein	6.0E-43	1.0E-10	8.8E-09	2.5	16:00	4:00
558	isotig05756	AT4G25640	DTX35	detoxifying efflux carrier 35	0.0	2.6E-11	3.1E-09	2.5	16:00	4:00
559	isotig06524	AT5G52420	−	−	1.0E-26	7.9E-10	4.7E-08	2.5	16:00	4:00
560	HI9HAF202B3L30	AT4G13180	−	NAD(P)-binding Rossmann-fold superfamily protein	0.0	5.0E-08	1.5E-06	2.5	16:00	8:00
561	HI9HAF202B521Z	AT5G15700	−	DNA/RNA polymerases superfamily protein	9.8E-45	1.3E-09	6.7E-08	2.5	16:00	8:00
562	HI9HAF202B6KMR	AT2G33860	ETT	Transcriptional factor B3 family protein / auxin-responsive factor AUX/IAA-related	1.0E-22	1.7E-08	7.3E-07	2.5	16:00	8:00
563	HI9HAF202B79W2	AT1G10760	SEX1	Pyruvate phosphate dikinase, PEP/pyruvate binding domain	0.0	4.2E-10	2.4E-08	2.5	16:00	8:00
564	HI9HAF202B7FEC	AT1G10760	SEX1	Pyruvate phosphate dikinase, PEP/pyruvate binding domain	0.0	9.1E-10	5.0E-08	2.5	16:00	8:00
565	HI9HAF202CJV4W	AT1G07030	−	Mitochondrial substrate carrier family protein	2.0E-44	3.7E-07	9.7E-06	2.5	16:00	8:00
566	HI9HAF202CL26P	AT5G15700	−	DNA/RNA polymerases superfamily protein	2.9E-44	7.8E-12	1.5E-09	2.5	16:00	8:00
567	HI9HAF203DK7OG	AT1G64810	APO1	Arabidopsis thaliana protein of unknown function (DUF794)	0.0	1.6E-10	1.8E-08	2.5	16:00	8:00
568	isotig00839	−	−	−	−	2.3E-10	1.8E-08	2.5	16:00	8:00
569	isotig00930	−	−	−	−	1.1E-03	1.3E-02	2.5	16:00	8:00
570	HI9HAF202B399C	AT1G10760	SEX1	Pyruvate phosphate dikinase, PEP/pyruvate binding domain	4.2E-45	1.4E-09	6.7E-08	2.5	20:00	8:00
571	isotig06109	AT3G15500	ATNAC3	NAC domain containing protein 3	0.0	1.2E-04	1.4E-03	2.5	20:00	8:00
572	HI9HAF202B2VPE	AT1G64660	ATMGL	methionine gamma-lyase	3.0E-36	2.2E-07	6.2E-06	2.5	4:00	12:00
573	isotig03846	AT5G66330	−	Leucine-rich repeat (LRR) family protein	0.0	3.2E-11	3.9E-09	2.5	4:00	12:00
574	isotig03851	AT1G72360	HRE1	Integrase-type DNA-binding superfamily protein	2.0E-21	6.6E-09	3.3E-07	2.5	4:00	12:00
575	HI9HAF203CY3P1	AT1G28690	−	Tetratricopeptide repeat (TPR)-like superfamily protein	0.0	3.9E-07	9.7E-06	2.5	20:00	12:00
576	isotig02733	AT3G17940	−	Galactose mutarotase-like superfamily protein	0.0	3.4E-04	4.2E-03	2.5	20:00	12:00
577	HI9HAF202B25D0	AT3G20440	EMB2729	Alpha amylase family protein	0.0	1.1E-06	2.1E-05	2.5	24:00	12:00
578	Shoot-002-67_005_C09	AT1G22340	AtUGT85A7	UDP-glucosyl transferase 85A7	0.0	1.7E-05	2.3E-04	2.5	24:00	12:00
579	Shoot-003-01_001_A01	−	−	−	−	4.1E-03	3.3E-02	2.5	24:00	12:00
580	Shoot-048-45	−	−	−	−	4.5E-03	3.5E-02	2.5	24:00	12:00
581	Shoot-055-73	AT5G15710	−	Galactose oxidase/kelch repeat superfamily protein	1.0E-12	6.8E-04	6.3E-03	2.5	24:00	12:00
582	HI9HAF203DPU17	−	−	−	−	2.5E-07	6.2E-06	2.5	4:00	16:00
583	isotig06130	−	−	−	−	1.1E-07	3.1E-06	2.5	4:00	16:00
584	Shoot-048-21	AT5G01810	CIPK15	CBL-interacting protein kinase 15	1.0E-34	5.7E-09	1.9E-07	2.5	4:00	16:00
585	HI9HAF203DDY6U	AT5G64120	−	Peroxidase superfamily protein	3.0E-22	1.1E-06	2.1E-05	2.5	8:00	16:00
586	isotig04171	−	−	−	−	1.0E-03	1.2E-02	2.5	8:00	16:00
587	isotig04173	AT4G34980	SLP2	subtilisin-like serine protease 2	0.0	1.1E-09	5.9E-08	2.5	8:00	16:00
588	Shoot-029-31	−	−	−	−	1.1E-06	2.1E-05	2.5	8:00	16:00
589	Shoot-053-22	AT5G62360	−	Plant invertase/pectin methylesterase inhibitor superfamily protein	5.0E-12	9.5E-09	3.6E-07	2.5	8:00	16:00
590	isotig02283	AT4G39660	AGT2	alanine:glyoxylate aminotransferase 2	0.0	1.2E-10	8.8E-09	2.5	24:00	16:00
591	HI9HAF203C6SYR	AT2G32950	COP1	Transducin/WD40 repeat-like superfamily protein	2.8E-45	3.8E-13	1.4E-10	2.5	8:00	20:00
592	isotig00662	AT1G75460	−	ATP-dependent protease La (LON) domain protein	0.0	9.9E-14	2.7E-11	2.5	8:00	20:00
593	isotig04606	−	−	−	−	1.6E-09	6.7E-08	2.5	8:00	20:00
594	isotig02091	AT1G74310	ATHSP101	heat shock protein 101	0.0	1.3E-05	1.9E-04	2.5	12:00	20:00
595	isotig02825	AT2G36870	XTH32	xyloglucan endotransglucosylase/hydrolase 32	0.0	1.1E-08	5.0E-07	2.5	8:00	24:00
596	isotig02598	AT3G14200	−	Chaperone DnaJ-domain superfamily protein	4.0E-28	9.5E-07	2.1E-05	2.5	12:00	24:00
597	isotig04626	AT1G22270	−	Trm112p-like protein	1.0E-41	6.3E-09	2.0E-07	2.5	12:00	24:00
598	isotig05046	AT5G39790	−	5'-AMP-activated protein kinase-related	3.0E-19	1.4E-15	1.1E-11	2.5	12:00	24:00
599	SSH12-3-16_016_H02	AT2G15220	−	Plant basic secretory protein (BSP) family protein	1.0E-15	6.1E-06	9.7E-05	2.5	12:00	24:00
600	HI9HAF203C9PDX	AT3G51280	−	Tetratricopeptide repeat (TPR)-like superfamily protein	0.0	1.4E-05	2.2E-04	2.5	16:00	24:00

601	HI9HAF202BWGZ3	AT4G02780	GA1	Terpenoid cyclases/Protein prenyltransferases superfamily protein	1.0E-28	2.7E-05	4.4E-04	2.4	12:00	4:00
602	HI9HAF203DQMHV	AT2G04030	CR88	Chaperone protein htpG family protein	0.0	3.7E-09	1.9E-07	2.4	12:00	4:00
603	isotig00164	AT4G24780	–	Pectin lyase-like superfamily protein	0.0	2.2E-06	5.1E-05	2.4	12:00	4:00
604	SSH24-3-37_009_E05	–	–	–	–	1.5E-05	2.2E-04	2.4	12:00	4:00
605	HI9HAF202CAVDN	AT3G26744	ICE1	basic helix-loop-helix (bHLH) DNA-binding superfamily protein	0.0	1.7E-11	1.7E-09	2.4	16:00	4:00
606	HI9HAF202CJZVZ	AT1G04690	KAB1	potassium channel beta subunit 1	0.0	1.1E-11	1.7E-09	2.4	16:00	4:00
607	HI9HAF203C74U1	AT3G28345	–	ABC transporter family protein	0.0	7.0E-07	1.4E-05	2.4	16:00	4:00
608	HI9HAF203CYQ64	–	–	–	–	4.1E-06	6.9E-05	2.4	16:00	4:00
609	isotig04692	–	–	–	–	8.9E-10	5.0E-08	2.4	16:00	4:00
610	isotig05890	AT5G52190	–	Sugar isomerase (SIS) family protein	2.0E-37	1.5E-06	2.7E-05	2.4	16:00	4:00
611	SSH24-9-33_001_A05	–	–	–	–	3.8E-07	9.7E-06	2.4	4:00	8:00
612	HI9HAF202BUOWC	AT3G27020	YSL6	YELLOW STRIPE like 6	0.0	2.4E-05	4.0E-04	2.4	16:00	8:00
613	HI9HAF202BZBCB	AT2G33860	ETT	Transcriptional factor B3 family protein / auxin-responsive factor AUX/IAA-related	2.0E-12	5.3E-07	1.4E-05	2.4	16:00	8:00
614	HI9HAF202CEW7U	AT1G68750	ATPPC4	phosphoenolpyruvate carboxylase 4	3.0E-15	4.3E-06	6.9E-05	2.4	16:00	8:00
615	HI9HAF203C5G81	AT3G27020	YSL6	YELLOW STRIPE like 6	0.0	4.0E-05	4.9E-04	2.4	16:00	8:00
616	HI9HAF203DJ75E	AT2G32390	GLR3.5	glutamate receptor 3.5	0.0	4.5E-10	2.4E-08	2.4	16:00	8:00
617	HI9HAF203DOLVW	AT5G15700	–	DNA/RNA polymerases superfamily protein	0.0	5.2E-12	7.9E-10	2.4	16:00	8:00
618	isotig06116	AT4G33170	–	Tetratricopeptide repeat (TPR)-like superfamily protein	0.0	1.3E-10	8.8E-09	2.4	16:00	8:00
619	Shoot-001-69_009_E09	–	–	–	–	1.4E-11	1.7E-09	2.4	16:00	8:00
620	Shoot-010-44	AT4G01037	WTF1	Ubiquitin carboxyl-terminal hydrolase family protein	4.0E-14	2.2E-10	1.8E-08	2.4	16:00	8:00
621	Shoot-030-02	AT1G53090	SPA4	SPA1-related 4	0.0	1.0E-10	8.8E-09	2.4	16:00	8:00
622	Shoot-047-77	–	–	–	–	2.7E-04	2.7E-03	2.4	16:00	8:00
623	Shoot-057-82	AT3G52072	–	other RNA	3.0E-11	3.3E-10	2.3E-08	2.4	16:00	8:00
624	SSH12-5-43_006_C06	–	–	–	–	3.6E-03	3.1E-02	2.4	16:00	8:00
625	HI9HAF203DAMSP	AT1G10760	SEX1	Pyruvate phosphate dikinase, PEP/pyruvate binding domain	0.0	1.6E-09	6.7E-08	2.4	20:00	8:00
626	HI9HAF203DOBMP	AT1G06410	ATTPS7	trehalose-phosphatase/synthase 7	0.0	6.0E-08	1.9E-06	2.4	20:00	8:00
627	isotig03971	AT1G01470	LEA14	Late embryogenesis abundant protein	9.0E-40	1.3E-07	3.2E-06	2.4	20:00	8:00
628	HI9HAF202CIW9K	AT2G13600	–	Pentatricopeptide repeat (PPR) superfamily protein	1.0E-43	2.8E-07	8.8E-06	2.4	16:00	12:00
629	HI9HAF203C4BMC	AT4G02750	–	Tetratricopeptide repeat (TPR)-like superfamily protein	0.0	6.4E-07	1.4E-05	2.4	16:00	12:00
630	HI9HAF202CFH1P	AT1G52340	ABA2	NAD(P)-binding Rossmann-fold superfamily protein	4.0E-31	1.1E-06	2.1E-05	2.4	20:00	12:00
631	HI9HAF203DATIK	–	–	–	–	2.3E-04	2.7E-03	2.4	20:00	12:00
632	isotig04911	AT3G19920	–	–	4.0E-20	7.5E-04	6.9E-03	2.4	20:00	12:00
633	isotig06038	–	–	–	–	2.7E-04	2.7E-03	2.4	20:00	12:00
634	Shoot-046-65	–	–	–	–	1.8E-06	3.7E-05	2.4	20:00	12:00
635	Shoot-050-15	AT1G74070	–	Cyclophilin-like peptidyl-prolyl cis-trans isomerase family protein	3.0E-36	4.9E-03	3.8E-02	2.4	20:00	12:00
636	Shoot-006-72_015_H09	AT5G47750	D6PKL2	D6 protein kinase like 2	0.0	1.6E-05	2.3E-04	2.4	24:00	12:00
637	Shoot-020-36_007_D05	AT1G19510	ATRL5	RAD-like 5	9.0E-21	2.4E-04	2.7E-03	2.4	24:00	12:00
638	SSH12-1-45_010_E06	AT4G26080	ABI1	Protein phosphatase 2C family protein	3.0E-40	3.6E-11	5.0E-09	2.4	4:00	16:00
639	SSH24-7-08_015_H01	–	–	–	–	2.6E-13	1.4E-10	2.4	4:00	16:00
640	HI9HAF202B7ZYN	AT2G40940	ERS1	ethylene response sensor 1	2.0E-32	9.1E-10	5.0E-08	2.4	8:00	16:00
641	HI9HAF202CFLCZ	AT1G26770	ATEXPA10	expansin A10	7.0E-41	1.0E-05	1.5E-04	2.4	8:00	16:00
642	HI9HAF203C3BRH	AT2G40940	ERS1	ethylene response sensor 1	2.0E-38	7.2E-11	7.7E-09	2.4	8:00	16:00
643	HI9HAF203DFFSV	AT5G08380	AtAGAL1	alpha-galactosidase 1	0.0	2.7E-05	4.2E-04	2.4	8:00	16:00
644	HI9HAF203DP6B2	AT1G51170	–	Protein kinase superfamily protein	1.0E-41	1.3E-10	8.8E-09	2.4	8:00	16:00
645	isotig04693	AT5G62020	AT-HSFB2A	heat shock transcription factor B2A	2.0E-44	1.6E-08	7.3E-07	2.4	8:00	16:00
646	Shoot-005-36_007_D05	AT2G31820	–	Ankyrin repeat family protein	1.0E-32	2.5E-10	1.8E-08	2.4	8:00	16:00
647	Shoot-012-42_004_B06	AT5G22860	–	Serine carboxypeptidase S28 family protein	0.0	2.8E-07	8.8E-06	2.4	8:00	16:00
648	HI9HAF203C1Y23	AT4G17550	–	Major facilitator superfamily protein	0.0	1.2E-08	6.6E-07	2.4	8:00	20:00
649	HI9HAF203C9GCX	AT1G18900	–	Pentatricopeptide repeat (PPR) superfamily protein	0.0	1.1E-08	4.3E-07	2.4	8:00	20:00
650	HI9HAF203DQWR9	–	–	–	–	4.3E-03	3.3E-02	2.4	8:00	20:00
651	isotig00236	AT4G17500	ATERF-1	ethylene responsive element binding factor 1	9.0E-27	6.8E-06	1.1E-04	2.4	8:00	20:00
652	isotig04194	AT1G17100	–	SOUL heme-binding family protein	0.0	1.9E-08	7.3E-07	2.4	8:00	20:00
653	isotig06083	AT2G05620	PGR5	proton gradient regulation 5	9.0E-30	4.2E-11	5.2E-09	2.4	8:00	20:00
654	HI9HAF202BWELV	AT5G15250	FTSH6	FTSH protease 6	0.0	5.6E-10	3.1E-08	2.4	12:00	20:00
655	isotig03952	AT2G20560	–	DNAJ heat shock family protein	0.0	8.5E-07	2.1E-05	2.4	12:00	20:00
656	isotig06086	AT4G24380	–		0.0	3.6E-07	9.7E-06	2.4	12:00	20:00
657	HI9HAF202BXP2W	AT5G58110	–	chaperone binding;ATPase activators	0.0	2.6E-05	4.0E-04	2.4	12:00	24:00
658	HI9HAF202CKBGD	AT5G24270	SOS3	Calcium-binding EF-hand family protein	0.0	6.0E-08	1.9E-06	2.4	12:00	24:00
659	isotig06003	AT1G07350	–	RNA-binding (RRM/RBD/RNP motifs) family protein	2.0E-37	2.6E-06	6.5E-05	2.4	12:00	24:00
660	SSH24-6-09_002_A02	AT1G23740	–	Oxidoreductase, zinc-binding dehydrogenase family protein	1.4E-45	4.4E-10	2.4E-08	2.4	12:00	24:00
661	HI9HAF202CGOFA	AT3G51280	–	Tetratricopeptide repeat (TPR)-like superfamily protein	0.0	1.7E-05	2.3E-04	2.4	16:00	24:00
662	HI9HAF202B4VT8	AT2G25140	HSP98.7	casein lytic proteinase B4	0.0	1.8E-04	1.8E-03	2.3	12:00	4:00
663	HI9HAF202BZIAY	AT3G26140	–	Cellulase (glycosyl hydrolase family 5) protein	0.0	9.7E-11	8.8E-09	2.3	12:00	4:00
664	HI9HAF202CB53Q	AT1G64390	AtGH9C2	glycosyl hydrolase 9C2	0.0	2.8E-06	6.9E-05	2.3	12:00	4:00
665	HI9HAF202CFVAC	AT4G11050	AtGH9C3	glycosyl hydrolase 9C3	0.0	1.1E-05	1.8E-04	2.3	12:00	4:00
666	HI9HAF202CHYME	AT3G23990	HSP60	heat shock protein 60	0.0	1.7E-06	2.7E-05	2.3	12:00	4:00
667	HI9HAF203DFAK0	AT3G26140	–	Cellulase (glycosyl hydrolase family 5) protein	0.0	2.7E-10	1.8E-08	2.3	12:00	4:00
668	isotig06277	AT3G07770	Hsp89.1	HEAT SHOCK PROTEIN 89.1	0.0	1.9E-09	1.1E-07	2.3	12:00	4:00
669	Shoot-006-12_008_D02	AT3G13677	–	–	9.0E-12	2.3E-08	9.1E-07	2.3	12:00	4:00
670	Shoot-057-41	AT2G33700	–	Protein phosphatase 2C family protein	5.0E-19	5.3E-10	3.1E-08	2.3	12:00	4:00
671	HI9HAF202CDFKH	–	–	–	–	2.0E-05	3.4E-04	2.3	16:00	4:00
672	HI9HAF203C3B9A	AT5G55950	–	Nucleotide/sugar transporter family protein	0.0	3.0E-12	6.6E-10	2.3	16:00	4:00
673	isotig03949	AT3G26590	–	MATE efflux family protein	0.0	1.0E-11	1.7E-09	2.3	16:00	4:00
674	isotig05388	AT1G18990	–	Protein of unknown function, DUF593	2.0E-17	1.6E-09	6.7E-08	2.3	16:00	4:00
675	isotig06685	–	–	–	–	4.4E-11	5.2E-09	2.3	16:00	4:00
676	isotig03034	AT5G57660	ATCOL5	CONSTANS-like 5	1.0E-28	1.1E-04	1.2E-03	2.3	20:00	4:00
677	SSH24-4-48_016_H06	AT5G24930	ATCOL4	CONSTANS-like 4	5.0E-37	5.0E-05	6.4E-04	2.3	20:00	4:00
678	HI9HAF202B0EQA	AT4G39700	–	Heavy metal transport/detoxification superfamily protein	0.0	6.0E-04	6.3E-03	2.3	4:00	8:00
679	HI9HAF202BW18X	AT1G75660	XRN3	5'-3' exoribonuclease 3	6.0E-42	3.5E-07	9.7E-06	2.3	16:00	8:00
680	HI9HAF202CKZKA	AT5G55830	–	Concanavalin A-like lectin protein kinase family protein	0.0	1.5E-05	2.2E-04	2.3	16:00	8:00
681	HI9HAF202CLCU0	AT3G26744	ICE1	basic helix-loop-helix (bHLH) DNA-binding superfamily protein	0.0	9.2E-11	8.7E-09	2.3	16:00	8:00
682	HI9HAF203C5OKX	AT4G14510	ATCFM3B	CRM family member 3B	6.0E-43	3.0E-12	6.6E-10	2.3	16:00	8:00
683	isotig01148	AT3G54450	–	Major facilitator superfamily protein	2.0E-29	7.1E-10	3.2E-08	2.3	16:00	8:00
684	isotig01951	AT1G30500	NF-YA7	nuclear factor Y, subunit A7	4.0E-36	2.9E-10	1.8E-08	2.3	16:00	8:00
685	isotig03402	AT5G47470	–	Nodulin MtN21 /EamA-like transporter family protein	0.0	1.9E-11	2.1E-09	2.3	16:00	8:00
686	isotig06219	AT1G11530	ATCXXS1	C-terminal cysteine residue is changed to a serine 1	4.0E-32	6.9E-05	9.4E-04	2.3	16:00	8:00
687	Shoot-020-28_008_D04	–	–	–	–	3.0E-05	4.9E-04	2.3	16:00	8:00
688	Shoot-048-32	AT5G18170	GDH1	glutamate dehydrogenase 1	0.0	1.5E-04	1.8E-03	2.3	16:00	8:00
689	HI9HAF203DQH5B	AT4G02750	–	Tetratricopeptide repeat (TPR)-like superfamily protein	0.0	2.9E-09	1.3E-07	2.3	20:00	8:00
690	isotig06832	–	–	–	–	4.0E-04	4.9E-03	2.3	20:00	8:00
691	HI9HAF203DJQE7	AT5G52040	ATRSP41	RNA-binding (RRM/RBD/RNP motifs) family protein	9.0E-43	9.5E-09	3.6E-07	2.3	4:00	12:00
692	HI9HAF203DRR0L	AT5G51060	RHD2	NADPH/respiratory burst oxidase protein D	7.0E-41	5.8E-14	2.7E-11	2.3	4:00	12:00
693	isotig06158	AT3G62630	–	Protein of unknown function (DUF1645)	4.0E-31	1.4E-07	3.2E-06	2.3	4:00	12:00
694	SSH24-3-42_004_B06	–	–	–	–	3.7E-04	4.2E-03	2.3	4:00	12:00
695	Shoot-007-68_007_D09	AT4G13650	–	Pentatricopeptide repeat (PPR) superfamily protein	0.0	4.0E-07	1.0E-05	2.3	16:00	12:00
696	HI9HAF202B13P3	AT5G15180	–	Peroxidase superfamily protein	6.0E-32	1.7E-05	2.3E-04	2.3	24:00	12:00
697	HI9HAF202B1H1R	AT3G21770	–	Peroxidase superfamily protein	2.0E-38	7.9E-06	1.3E-04	2.3	24:00	12:00
698	HI9HAF202CGHZY	AT2G21050	LAX2	like AUXIN RESISTANT 2	5.0E-17	8.1E-05	9.4E-04	2.3	24:00	12:00
699	HI9HAF203C30OA	AT3G20440	EMB2729	Alpha amylase family protein	2.9E-44	1.9E-06	3.7E-05	2.3	24:00	12:00
700	isotig02602	ATCG01130	YCF1.2	Ycf1 protein	2.0E-17	4.4E-03	3.3E-02	2.3	24:00	12:00

701	isotig06760	AT5G15780	–	Pollen Ole e 1 allergen and extensin family protein	1.0E-20	3.0E-06	6.9E-05	2.3	24:00	12:00
702	Shoot-029-01	AT4G24210	SLY1	F-box family protein	2.0E-21	3.4E-03	3.0E-02	2.3	24:00	12:00
703	HI9HAF203C56D5	AT1G32090	–	early-responsive to dehydration stress protein (ERD4)	0.0	1.1E-07	3.1E-06	2.3	4:00	16:00
704	HI9HAF203CX03E	AT1G33440	–	Major facilitator superfamily protein	0.0	1.0E-06	2.1E-05	2.3	4:00	16:00
705	HI9HAF203DAYP6	–	–	–	–	3.3E-04	2.8E-03	2.3	4:00	16:00
706	isotig05975	–	–	–	–	1.4E-10	1.1E-08	2.3	4:00	16:00
707	isotig06144	AT5G47750	D6PKL2	D6 protein kinase like 2	0.0	4.6E-14	2.7E-11	2.3	4:00	16:00
708	isotig06229	–	–	–	–	2.2E-04	2.4E-03	2.3	4:00	16:00
709	isotig01735	AT1G79900	ATMBAC2	Mitochondrial substrate carrier family protein	0.0	4.8E-10	2.4E-08	2.3	8:00	16:00
710	isotig02986	AT1G52770	–	Phototropic-responsive NPH3 family protein	0.0	1.6E-08	7.3E-07	2.3	8:00	16:00
711	isotig04779	AT2G41510	ATCKX1	cytokinin oxidase/dehydrogenase 1	0.0	1.4E-11	1.7E-09	2.3	8:00	16:00
712	isotig05919	AT4G19450	–	Major facilitator superfamily protein	3.0E-28	1.3E-05	1.9E-04	2.3	8:00	16:00
713	Shoot-006-51_005_C07	AT3G19990	–	–	0.0	7.7E-07	1.7E-05	2.3	8:00	16:00
714	SSH24-6-19_005_C03	–	–	–	–	4.6E-11	5.2E-09	2.3	8:00	16:00
715	SSH24-8-04_007_D01	–	–	–	–	9.1E-10	5.0E-08	2.3	8:00	16:00
716	isotig06357	AT3G10020	–	–	4.0E-13	4.7E-06	6.9E-05	2.3	12:00	16:00
717	HI9HAF203DLG5Q	AT4G22990	–	Major Facilitator Superfamily with SPX (SYG1/Pho81/XPR1) domain-containing protein	1.0E-43	1.6E-07	3.2E-06	2.3	8:00	20:00
718	HI9HAF203DNGBQ	AT1G67720	–	Leucine-rich repeat protein kinase family protein	5.0E-43	3.5E-06	6.9E-05	2.3	8:00	20:00
719	Shoot-017-37_009_E05	AT2G41000	–	Chaperone DnaJ-domain superfamily protein	9.0E-21	2.1E-11	2.5E-09	2.3	8:00	20:00
720	Shoot-025-46	–	–	–	–	2.0E-09	1.1E-07	2.3	8:00	20:00
721	Shoot-055-62	AT4G34350	CLB6	4-hydroxy-3-methylbut-2-enyl diphosphate reductase	0.0	1.1E-12	6.0E-10	2.3	8:00	20:00
722	HI9HAF202B4J2Z	AT4G15420	–	Ubiquitin fusion degradation UFD1 family protein	3.0E-41	1.5E-06	2.7E-05	2.3	12:00	20:00
723	HI9HAF203DGJKQ	AT1G78370	ATGSTU20	glutathione S-transferase TAU 20	1.0E-42	2.9E-03	2.4E-02	2.3	12:00	20:00
724	isotig00987	AT3G62600	ATERDJ3B	DNAJ heat shock family protein	0.0	8.8E-07	2.1E-05	2.3	12:00	20:00
725	isotig01544	AT2G36870	XTH32	xyloglucan endotransglucosylase/hydrolase 32	0.0	1.9E-11	2.5E-09	2.3	12:00	24:00
726	isotig05496	AT3G23990	HSP60	heat shock protein 60	0.0	1.7E-06	2.7E-05	2.3	12:00	24:00
727	HI9HAF203C102G	AT4G11050	AtGH9C3	glycosyl hydrolase 9C3	0.0	2.4E-06	6.5E-05	2.2	12:00	4:00
728	HI9HAF203C21Q1	AT3G62980	TIR1	F-box/RNI-like superfamily protein	4.0E-38	7.5E-06	1.2E-04	2.2	12:00	4:00
729	HI9HAF203DMBYB	AT3G25500	AFH1	formin homology 1	0.0	1.1E-07	3.1E-06	2.2	12:00	4:00
730	isotig01370	AT2G46950	CYP709B2	cytochrome P450, family 709, subfamily B, polypeptide 2	0.0	1.3E-06	2.5E-05	2.2	12:00	4:00
731	isotig03321	AT5G01880	–	RING/U-box superfamily protein	1.0E-29	3.6E-11	5.2E-09	2.2	12:00	4:00
732	isotig04328	–	–	–	–	2.0E-08	7.3E-07	2.2	12:00	4:00
733	isotig06223	–	–	–	–	3.2E-07	9.7E-06	2.2	12:00	4:00
734	Shoot-012-15_014_G02	AT5G15450	APG6	casein lytic proteinase B3	0.0	4.1E-07	1.0E-05	2.2	12:00	4:00
735	HI9HAF202BUDWE	AT5G39840	–	ATP-dependent RNA helicase, mitochondrial, putative	0.0	4.7E-09	1.9E-07	2.2	16:00	4:00
736	HI9HAF202CA6JB	AT4G02750	–	Tetratricopeptide repeat (TPR)-like superfamily protein	0.0	2.1E-05	3.4E-04	2.2	16:00	4:00
737	HI9HAF202CE1RL	AT3G46970	ATPHS2	alpha-glucan phosphorylase 2	0.0	9.7E-06	1.5E-04	2.2	16:00	4:00
738	HI9HAF203C8BTF	AT1G55910	ZIP11	zinc transporter 11 precursor	8.0E-41	9.6E-10	5.9E-08	2.2	16:00	4:00
739	HI9HAF203DBZPJ	AT5G55950	–	Nucleotide/sugar transporter family protein	8.0E-43	3.1E-12	6.6E-10	2.2	16:00	4:00
740	HI9HAF203DIDF6	AT1G04690	KAB1	potassium channel beta subunit 1	0.0	1.4E-09	6.7E-08	2.2	16:00	4:00
741	HI9HAF203DKIX6	AT2G23060	–	Acyl-CoA N-acyltransferases (NAT) superfamily protein	1.0E-43	1.6E-06	2.7E-05	2.2	16:00	4:00
742	isotig00643	AT3G13620	–	Amino acid permease family protein	0.0	2.9E-06	6.9E-05	2.2	16:00	4:00
743	isotig00739	AT5G06570	–	alpha/beta-Hydrolases superfamily protein	0.0	1.8E-03	1.8E-02	2.2	16:00	4:00
744	isotig03145	AT1G68750	ATPPC4	phosphoenolpyruvate carboxylase 4	0.0	1.6E-06	2.7E-05	2.2	16:00	4:00
745	isotig03953	AT4G14130	XTR7	xyloglucan endotransglucosylase/hydrolase 15	0.0	4.2E-04	4.9E-03	2.2	16:00	4:00
746	isotig05913	AT5G57480	–	P-loop containing nucleoside triphosphate hydrolases superfamily protein	0.0	5.6E-10	3.1E-08	2.2	16:00	4:00
747	Shoot-048-06	AT3G60490	–	Integrase-type DNA-binding superfamily protein	1.0E-32	9.1E-04	9.3E-03	2.2	4:00	8:00
748	HI9HAF202B0PLZ	AT4G33990	EMB2758	Tetratricopeptide repeat (TPR)-like superfamily protein	0.0	6.5E-10	3.1E-08	2.2	16:00	8:00
749	HI9HAF202B3SBC	AT1G75660	XRN3	5'-3' exoribonuclease 3	0.0	1.1E-07	2.6E-06	2.2	16:00	8:00
750	HI9HAF202B5UN4	AT4G01037	WTF1	Ubiquitin carboxyl-terminal hydrolase family protein	0.0	1.2E-11	1.7E-09	2.2	16:00	8:00
751	HI9HAF202BZFRA	AT1G12770	ISE1	P-loop containing nucleoside triphosphate hydrolases superfamily protein	0.0	1.5E-11	1.7E-09	2.2	16:00	8:00
752	HI9HAF202CJBOP	AT1G75660	XRN3	5'-3' exoribonuclease 3	1.4E-45	1.1E-07	2.6E-06	2.2	16:00	8:00
753	HI9HAF202CLUAO	AT2G35030	–	Pentatricopeptide repeat (PPR) superfamily protein	1.0E-43	7.1E-07	1.4E-05	2.2	16:00	8:00
754	HI9HAF203DGPKH	AT2G37040	PAL1	PHE ammonia lyase 1	2.0E-34	1.6E-06	2.7E-05	2.2	16:00	8:00
755	isotig00771	AT2G16600	ROC3	rotamase CYP 3	0.0	4.8E-10	2.4E-08	2.2	16:00	8:00
756	isotig01408	AT2G39210	–	Major facilitator superfamily protein	0.0	2.4E-07	6.2E-06	2.2	16:00	8:00
757	isotig02286	AT4G13250	NYC1	NAD(P)-binding Rossmann-fold superfamily protein	0.0	2.3E-11	2.5E-09	2.2	16:00	8:00
758	Shoot-017-91_006_C12	AT2G21090	–	Pentatricopeptide repeat (PPR-like) superfamily protein	3.0E-40	7.3E-08	1.9E-06	2.2	16:00	8:00
759	Shoot-024-67	AT3G54420	ATEP3	homolog of carrot EP3-3 chitinase	0.0	4.5E-04	5.0E-03	2.2	16:00	8:00
760	Shoot-047-94	AT4G01037	WTF1	Ubiquitin carboxyl-terminal hydrolase family protein	2.0E-41	1.5E-10	1.2E-08	2.2	16:00	8:00
761	HI9HAF202B3JCJ	AT1G60140	ATTPS10	trehalose phosphate synthase	0.0	4.7E-06	6.9E-05	2.2	20:00	8:00
762	isotig03640	–	–	–	–	2.6E-05	4.0E-04	2.2	20:00	8:00
763	isotig06564	AT1G18940	–	Nodulin-like / Major Facilitator Superfamily protein	3.0E-32	2.8E-07	7.1E-06	2.2	20:00	8:00
764	isotig01102	–	–	–	–	1.8E-12	6.6E-10	2.2	24:00	8:00
765	isotig01103	–	–	–	–	2.3E-11	2.5E-09	2.2	24:00	8:00
766	HI9HAF202B3961	AT1G11260	STP1	sugar transporter 1	0.0	4.6E-03	3.5E-02	2.2	4:00	12:00
767	HI9HAF202CEN3B	AT4G38620	ATMYB4	myb domain protein 4	7.0E-42	8.2E-04	8.1E-03	2.2	4:00	12:00
768	HI9HAF203C3CAU	AT4G02780	GA1	Terpenoid cyclases/Protein prenyltransferases superfamily protein	2.0E-27	5.7E-03	4.3E-02	2.2	4:00	12:00
769	HI9HAF203C7FUB	AT5G18170	GDH1	glutamate dehydrogenase 1	1.0E-42	4.1E-04	4.9E-03	2.2	4:00	12:00
770	isotig06694	AT1G55020	LOX1	lipoxygenase 1	9.0E-34	1.3E-06	2.5E-05	2.2	4:00	12:00
771	Shoot-020-33_001_A05	–	–	–	–	9.6E-04	1.1E-02	2.2	4:00	12:00
772	Shoot-055-78	AT4G13650	–	Pentatricopeptide repeat (PPR) superfamily protein	0.0	4.3E-07	1.2E-05	2.2	16:00	12:00
773	HI9HAF202CIH7U	AT2G25520	–	Drug/metabolite transporter superfamily protein	0.0	7.9E-05	9.4E-04	2.2	20:00	12:00
774	isotig03060	AT5G04830	–	Nuclear transport factor 2 (NTF2) family protein	0.0	1.2E-08	5.0E-07	2.2	20:00	12:00
775	isotig05149	AT3G50830	COR413-PM2	cold-regulated 413-plasma membrane 2	0.0	1.1E-08	4.3E-07	2.2	20:00	12:00
776	isotig06724	–	–	–	–	4.4E-06	6.9E-05	2.2	20:00	12:00
777	HI9HAF202CBN8U	AT3G21770	–	Peroxidase superfamily protein	3.0E-18	3.5E-05	4.9E-04	2.2	24:00	12:00
778	Shoot-048-67	–	–	–	–	3.5E-05	4.9E-04	2.2	24:00	12:00
779	HI9HAF202B69K2	AT5G54250	CNGC4	cyclic nucleotide-gated cation channel 4	0.0	1.1E-08	4.3E-07	2.2	4:00	16:00
780	HI9HAF202CFUEA	AT5G63810	BGAL10	beta-galactosidase 10	0.0	3.0E-09	1.3E-07	2.2	4:00	16:00
781	HI9HAF203DF8OF	AT5G63810	BGAL10	beta-galactosidase 10	0.0	1.1E-07	3.1E-06	2.2	4:00	16:00
782	isotig03104	AT5G45820	CIPK20	CBL-interacting protein kinase 20	0.0	7.5E-09	3.3E-07	2.2	4:00	16:00
783	isotig06805	AT5G45120	–	Eukaryotic aspartyl protease family protein	3.0E-36	1.9E-06	3.7E-05	2.2	4:00	16:00
784	Shoot-046-24	–	–	–	–	4.3E-08	1.4E-06	2.2	4:00	16:00
785	HI9HAF202BSDV2	AT2G37050	–	Leucine-rich repeat protein kinase family protein	0.0	1.1E-05	1.8E-04	2.2	8:00	16:00
786	isotig01338	AT1G56120	–	Leucine-rich repeat transmembrane protein kinase	0.0	7.5E-13	1.4E-10	2.2	8:00	16:00
787	isotig01692	AT3G11670	DGD1	UDP-Glycosyltransferase superfamily protein	0.0	1.0E-07	2.6E-06	2.2	8:00	16:00
788	isotig04053	AT1G78020	–	Protein of unknown function (DUF581)	3.0E-14	9.3E-09	3.6E-07	2.2	8:00	16:00
789	isotig06075	AT3G43270	–	Plant invertase/pectin methylesterase inhibitor superfamily	5.0E-44	4.5E-06	6.9E-05	2.2	8:00	16:00
790	Shoot-039-05	AT2G02061	–	Nucleotide-diphospho-sugar transferase family protein	0.0	9.8E-09	3.6E-07	2.2	8:00	16:00
791	Shoot-057-59	AT1G17940	–	Endosomal targeting BRO1-like domain-containing protein	4.0E-25	1.3E-07	3.2E-06	2.2	8:00	16:00
792	SSH24-6-48_016_H06	–	–	–	–	5.9E-05	6.4E-04	2.2	8:00	16:00
793	HI9HAF202CBU21	AT1G49720	ABF1	abscisic acid responsive element-binding factor 1	1.0E-35	5.6E-07	1.4E-05	2.2	12:00	16:00
794	HI9HAF202CC878	AT3G28860	ATMDR1	ATP binding cassette subfamily B19	0.0	1.3E-08	6.6E-07	2.2	24:00	16:00
795	HI9HAF203DEMEB	AT1G15210	PDR7	pleiotropic drug resistance 7	0.0	7.7E-04	8.1E-03	2.2	24:00	16:00
796	HI9HAF203DI6MQ	AT3G28860	ATMDR1	ATP binding cassette subfamily B19	0.0	2.9E-08	1.0E-06	2.2	24:00	16:00
797	isotig05258	AT1G72890	–	Disease resistance protein (TIR-NBS class)	5.0E-28	1.7E-03	1.8E-02	2.2	4:00	20:00
798	HI9HAF202CL3Z5	AT4G22990	–	Major Facilitator Superfamily with SPX (SYG1/Pho81/XPR1) domain-containing protein	4.0E-43	1.6E-07	3.2E-06	2.2	8:00	20:00
799	HI9HAF203DFQI0	AT3G43120	–	SAUR-like auxin-responsive protein family	2.0E-24	3.0E-09	1.3E-07	2.2	8:00	20:00
800	HI9HAF203DROKL	AT1G02270	–	Calcium-binding endonuclease/exonuclease/phosphatase family	0.0	9.8E-09	3.6E-07	2.2	8:00	20:00

801	isotig04584	－	－	－	－	6.1E-04	6.3E-03	2.2	8:00	20:00
802	isotig05959	AT3G19950	－	RING/U-box superfamily protein	1.0E-37	2.4E-08	1.0E-06	2.2	8:00	20:00
803	Shoot-021-64_016_H08	AT1G22400	UGT85A1	UDP-Glycosyltransferase superfamily protein	2.0E-24	3.5E-03	3.1E-02	2.2	8:00	20:00
804	SSH12-8-82_003_B11	－	－	－	－	1.1E-05	1.8E-04	2.2	8:00	20:00
805	HI9HAF202CKEAV	AT4G15420	－	Ubiquitin fusion degradation UFD1 family protein	9.0E-44	7.2E-07	1.4E-05	2.2	12:00	20:00
806	isotig00315	AT3G44190	－	FAD/NAD(P)-binding oxidoreductase family protein	9.0E-38	4.7E-06	6.9E-05	2.2	12:00	20:00
807	isotig03358	AT5G64260	EXL2	EXORDIUM like 2	0.0	2.6E-04	2.7E-03	2.2	12:00	20:00
808	Shoot-057-57	AT1G18180	－	Protein of unknown function (DUF1295)	0.0	7.7E-11	8.7E-09	2.2	12:00	20:00
809	HI9HAF202B9Y9X	AT5G06460	ATUBA2	ubiquitin activating enzyme 2	0.0	6.3E-04	6.3E-03	2.2	4:00	24:00
810	HI9HAF202B8QRS	AT4G26780	AR192	Co-chaperone GrpE family protein	0.0	6.0E-10	3.1E-08	2.2	12:00	24:00
811	HI9HAF202CBN5I	AT5G24270	SOS3	Calcium-binding EF-hand family protein	0.0	3.0E-08	1.0E-06	2.2	12:00	24:00
812	HI9HAF203DG5DR	AT5G05390	LAC12	laccase 12	4.0E-43	7.5E-04	6.9E-03	2.2	12:00	24:00
813	HI9HAF203DNML3	AT5G60020	LAC17	laccase 17	0.0	3.9E-04	4.6E-03	2.2	12:00	24:00
814	isotig04021	AT5G41040	－	HXXXD-type acyl-transferase family protein	2.0E-42	2.1E-06	5.1E-05	2.2	12:00	24:00
815	isotig05138	AT1G18180	－	Protein of unknown function (DUF1295)	0.0	3.1E-09	1.3E-07	2.2	12:00	24:00
816	Shoot-048-76	－	－	－	－	5.5E-05	6.4E-04	2.2	12:00	24:00
817	HI9HAF202B5KSD	AT3G25900	HMT-1	Homocysteine S-methyltransferase family protein	0.0	1.1E-06	2.1E-05	2.2	16:00	24:00
818	Shoot-048-87	AT2G21940	SK1	shikimate kinase 1	4.0E-17	2.8E-10	1.8E-08	2.2	16:00	24:00
819	HI9HAF202B2WVG	AT3G26810	AFB2	auxin signaling F-box 2	6.0E-44	1.3E-04	1.5E-03	2.1	12:00	4:00
820	HI9HAF202BV7C9	AT2G30000	－	PHF5-like protein	0.0	1.3E-04	1.5E-03	2.1	12:00	4:00
821	HI9HAF202BVPDJ	－	－	－	－	1.4E-06	2.5E-05	2.1	12:00	4:00
822	HI9HAF202BVZ8Z	AT3G24503	ALDH2C4	aldehyde dehydrogenase 2C4	8.0E-43	1.5E-10	1.2E-08	2.1	12:00	4:00
823	HI9HAF202BVZYJ	AT5G05350	－	PLAC8 family protein	4.2E-45	4.5E-11	5.2E-09	2.1	12:00	4:00
824	HI9HAF202CFFXU	AT1G17870	ATEGY3	ethylene-dependent gravitropism-deficient and yellow-green-like 3	0.0	1.7E-07	3.2E-06	2.1	12:00	4:00
825	HI9HAF202CG30A	AT5G15450	APG6	casein lytic proteinase B3	0.0	1.3E-07	3.2E-06	2.1	12:00	4:00
826	HI9HAF202CGT2R	AT3G62980	TIR1	F-box/RNI-like superfamily protein	8.0E-42	6.7E-06	1.1E-04	2.1	12:00	4:00
827	HI9HAF203C71ZO	AT5G19530	ACL5	S-adenosyl-L-methionine-dependent methyltransferases superfamily protein	1.0E-38	9.6E-06	1.5E-04	2.1	12:00	4:00
828	isotig01075	AT4G37680	HHP4	heptahelical protein 4	5.0E-24	8.4E-07	1.8E-05	2.1	12:00	4:00
829	isotig02130	AT1G04980	ATPDIL2-2	PDI-like 2-2	0.0	7.6E-08	1.9E-06	2.1	12:00	4:00
830	isotig02155	AT5G64510	TIN1	－	0.0	8.6E-08	2.3E-06	2.1	12:00	4:00
831	isotig04350	AT2G40610	ATEXPA8	expansin A8	0.0	1.3E-04	1.4E-03	2.1	12:00	4:00
832	Shoot-006-60_008_D08	－	－	－	－	1.2E-06	2.5E-05	2.1	12:00	4:00
833	Shoot-020-30_012_F04	AT5G36930	－	Disease resistance protein (TIR-NBS-LRR class) family	5.0E-25	2.6E-03	2.3E-02	2.1	12:00	4:00
834	SSH12-6-76_008_D10	－	－	－	－	4.8E-07	1.4E-05	2.1	12:00	4:00
835	HI9HAF202BSX33	AT5G39840	－	ATP-dependent RNA helicase, mitochondrial, putative	6.0E-43	1.4E-07	3.2E-06	2.1	16:00	4:00
836	HI9HAF202CL0YN	AT5G39840	－	ATP-dependent RNA helicase, mitochondrial, putative	0.0	1.2E-08	5.0E-07	2.1	16:00	4:00
837	HI9HAF203C3DTE	AT1G06440	－	Ubiquitin carboxyl-terminal hydrolase family protein	0.0	5.0E-08	1.5E-06	2.1	16:00	4:00
838	HI9HAF203DPKIM	AT3G25900	HMT-1	Homocysteine S-methyltransferase family protein	0.0	6.6E-06	1.1E-04	2.1	16:00	4:00
839	isotig01521	AT4G00370	ANTR2	Major facilitator superfamily protein	0.0	5.8E-08	1.9E-06	2.1	16:00	4:00
840	isotig02041	AT5G04630	CYP77A9	cytochrome P450, family 77, subfamily A, polypeptide 9	0.0	2.2E-07	6.2E-06	2.1	16:00	4:00
841	isotig06492	AT1G23740	－	Oxidoreductase, zinc-binding dehydrogenase family protein	0.0	5.7E-04	6.1E-03	2.1	16:00	4:00
842	SSH12-3-24_015_H03	－	－	－	－	3.2E-04	2.8E-03	2.1	16:00	4:00
843	SSH24-7-01_001_A01	－	－	－	－	3.2E-03	2.9E-02	2.1	16:00	4:00
844	HI9HAF203DSDCM	AT1G15500	ATNTT2	TLC ATP/ADP transporter	0.0	7.1E-06	1.1E-04	2.1	20:00	4:00
845	isotig06814	AT5G24930	ATCOL4	CONSTANS-like 4	1.0E-34	1.4E-04	1.5E-03	2.1	20:00	4:00
846	HI9HAF202B29ZJ	AT5G14580	－	polyribonucleotide nucleotidyltransferase, putative	0.0	5.7E-07	1.4E-05	2.1	16:00	8:00
847	HI9HAF202B2Z9S	AT5G47910	RBOHD	respiratory burst oxidase homologue D	0.0	4.7E-05	6.4E-04	2.1	16:00	8:00
848	HI9HAF202B517Z	AT3G57430	OTP84	Tetratricopeptide repeat (TPR)-like superfamily protein	0.0	6.2E-09	2.0E-07	2.1	16:00	8:00
849	HI9HAF202CB40S	AT5G25560	－	CHY-type/CTCHY-type/RING-type Zinc finger protein	0.0	1.3E-03	1.3E-02	2.1	16:00	8:00
850	HI9HAF202CBR5J	AT3G26782	－	Tetratricopeptide repeat (TPR)-like superfamily protein	2.0E-41	5.9E-07	1.4E-05	2.1	16:00	8:00
851	HI9HAF202CFWUK	AT5G55830	－	Concanavalin A-like lectin protein kinase family protein	0.0	1.7E-05	2.3E-04	2.1	16:00	8:00
852	HI9HAF202CJFA9	AT1G75660	XRN3	5'-3' exoribonuclease 3	0.0	5.7E-07	1.4E-05	2.1	16:00	8:00
853	HI9HAF203C271O	AT5G55830	－	Concanavalin A-like lectin protein kinase family protein	0.0	5.1E-05	6.4E-04	2.1	16:00	8:00
854	HI9HAF203C3PJI	AT3G57430	OTP84	Tetratricopeptide repeat (TPR)-like superfamily protein	0.0	9.9E-09	3.6E-07	2.1	16:00	8:00
855	HI9HAF203CZ4FC	AT1G67960	－	CONTAINS InterPro DOMAIN/s: Membrane protein,Tapt1/CMV receptor (InterPro:IPR008010)	2.0E-44	6.9E-05	9.4E-04	2.1	16:00	8:00
856	HI9HAF203DAAVZ	－	－	－	－	1.7E-06	2.7E-05	2.1	16:00	8:00
857	HI9HAF203DCF0R	AT1G67960	－	CONTAINS InterPro DOMAIN/s: Membrane protein,Tapt1/CMV receptor (InterPro:IPR008010)	1.0E-41	1.1E-04	1.3E-03	2.1	16:00	8:00
858	HI9HAF203DFVK3	AT1G75660	XRN3	5'-3' exoribonuclease 3	0.0	3.3E-09	1.9E-07	2.1	16:00	8:00
859	HI9HAF203DG3DY	AT3G05510	－	Phospholipid/glycerol acyltransferase family protein	0.0	1.1E-08	4.3E-07	2.1	16:00	8:00
860	HI9HAF203DG44P	AT4G01037	WTF1	Ubiquitin carboxyl-terminal hydrolase family protein	0.0	1.7E-11	1.7E-09	2.1	16:00	8:00
861	HI9HAF203DIZJ5	AT3G05510	－	Phospholipid/glycerol acyltransferase family protein	0.0	3.9E-09	1.9E-07	2.1	16:00	8:00
862	HI9HAF203DPQZS	AT4G30825	－	Tetratricopeptide repeat (TPR)-like superfamily protein	1.0E-41	9.9E-12	1.7E-09	2.1	16:00	8:00
863	isotig00770	AT2G16600	ROC3	rotamase CYP 3	0.0	6.5E-10	3.1E-08	2.1	16:00	8:00
864	isotig01119	－	－	－	－	2.8E-11	3.1E-09	2.1	16:00	8:00
865	isotig03091	AT1G75660	XRN3	5'-3' exoribonuclease 3	6.0E-22	2.3E-07	6.2E-06	2.1	16:00	8:00
866	HI9HAF202B1CBE	AT1G67110	CYP735A2	cytochrome P450, family 735, subfamily A, polypeptide 2	9.0E-23	7.9E-04	8.1E-03	2.1	20:00	8:00
867	HI9HAF203DNRAY	AT1G55020	LOX1	lipoxygenase 1	1.0E-10	4.7E-07	1.3E-05	2.1	20:00	8:00
868	isotig01762	AT3G11410	ATPP2CA	protein phosphatase 2CA	0.0	6.9E-08	1.9E-06	2.1	20:00	8:00
869	isotig04971	AT3G54260	TBL36	TRICHOME BIREFRINGENCE-LIKE 36	0.0	1.5E-13	8.3E-11	2.1	20:00	8:00
870	HI9HAF202B0NQ5	AT3G18830	ATPLT5	polyol/monosaccharide transporter 5	0.0	6.6E-07	1.4E-05	2.1	4:00	12:00
871	HI9HAF202CE9R3	AT4G32390	－	Nucleotide-sugar transporter family protein	0.0	3.4E-06	6.9E-05	2.1	4:00	12:00
872	HI9HAF202CHWK4	AT4G32390	－	Nucleotide-sugar transporter family protein	0.0	6.9E-06	1.1E-04	2.1	4:00	12:00
873	HI9HAF203CYDVV	AT4G37530	－	Peroxidase superfamily protein	0.0	1.1E-08	5.0E-07	2.1	4:00	12:00
874	HI9HAF203DK8BF	AT2G20780	－	Major facilitator superfamily protein	0.0	1.1E-07	2.6E-06	2.1	4:00	12:00
875	isotig03928	AT1G53830	ATPME2	pectin methylesterase 2	2.0E-30	1.3E-04	1.5E-03	2.1	4:00	12:00
876	isotig04360	－	－	－	－	3.8E-08	1.4E-06	2.1	4:00	12:00
877	isotig06679	AT3G55520	－	FKBP-like peptidyl-prolyl cis-trans isomerase family protein	2.0E-24	2.9E-04	2.7E-03	2.1	4:00	12:00
878	SSH24-3-67_005_C09	AT1G01490	－	Heavy metal transport/detoxification superfamily protein	1.0E-21	3.2E-07	9.7E-06	2.1	4:00	12:00
879	HI9HAF203DEVYR	AT4G02750	－	Tetratricopeptide repeat (TPR)-like superfamily protein	7.0E-43	1.3E-03	1.3E-02	2.1	16:00	12:00
880	HI9HAF202CIXYH	AT2G25520	－	Drug/metabolite transporter superfamily protein	5.0E-41	1.1E-05	1.8E-04	2.1	20:00	12:00
881	HI9HAF203DE1FE	AT4G13650	－	Pentatricopeptide repeat (PPR) superfamily protein	2.8E-45	1.2E-07	3.2E-06	2.1	20:00	12:00
882	isotig00944	AT5G18100	CSD3	copper/zinc superoxide dismutase 3	0.0	3.2E-07	9.7E-06	2.1	20:00	12:00
883	isotig03583	－	－	－	－	2.5E-04	2.7E-03	2.1	20:00	12:00
884	isotig05554	AT3G03990	－	alpha/beta-Hydrolases superfamily protein	0.0	1.2E-04	1.4E-03	2.1	20:00	12:00
885	Shoot-001-51_005_C07	AT5G04830	－	Nuclear transport factor 2 (NTF2) family protein	0.0	1.2E-09	5.9E-08	2.1	20:00	12:00
886	HI9HAF202CHGAJ	AT2G16120	PMT1	polyol/monosaccharide transporter 1	2.8E-45	1.2E-06	2.5E-05	2.1	24:00	12:00
887	HI9HAF203DL1C2	AT4G02780	GA1	Terpenoid cyclases/Protein prenyltransferases superfamily protein	2.0E-14	1.5E-03	1.4E-02	2.1	24:00	12:00
888	isotig03594	AT1G22360	AtUGT85A2	UDP-glucosyl transferase 85A2	0.0	1.4E-05	1.9E-04	2.1	24:00	12:00
889	Shoot-008-11_006_C02	AT1G22360	AtUGT85A2	UDP-glucosyl transferase 85A2	0.0	1.4E-05	2.2E-04	2.1	24:00	12:00
890	SSH24-2-65_001_A09	AT1G01490	－	Heavy metal transport/detoxification superfamily protein	1.0E-20	1.2E-09	5.9E-08	2.1	24:00	12:00
891	SSH24-6-72_015_H09	AT4G02420	－	Concanavalin A-like lectin protein kinase family protein	1.0E-39	3.9E-10	2.4E-08	2.1	24:00	12:00
892	HI9HAF202CCWV7	－	－	－	－	3.8E-04	4.2E-03	2.1	4:00	16:00
893	HI9HAF202CI4ZB	AT4G37800	XTH7	xyloglucan endotransglucosylase/hydrolase 7	3.0E-43	2.2E-04	2.4E-03	2.1	4:00	16:00
894	HI9HAF203C8O5G	－	－	－	－	1.8E-09	1.1E-07	2.1	4:00	16:00
895	HI9HAF203DKTU2	AT1G31120	KUP10	K+ uptake permease 10	0.0	5.6E-06	9.5E-05	2.1	4:00	16:00
896	isotig02093	AT4G36360	BGAL3	beta-galactosidase 3	0.0	1.1E-07	2.6E-06	2.1	4:00	16:00
897	isotig04301	－	－	－	－	9.2E-08	2.3E-06	2.1	4:00	16:00
898	isotig04891	AT1G07710	－	Ankyrin repeat family protein	1.0E-40	3.5E-11	5.0E-09	2.1	4:00	16:00
899	isotig05821	－	－	－	－	4.1E-05	4.9E-04	2.1	4:00	16:00
900	isotig05836	－	－	－	－	3.9E-07	9.7E-06	2.1	4:00	16:00

901	Shoot-026-12	AT2G26710	BAS1	Cytochrome P450 superfamily protein	7.0E-24	4.3E-06	6.9E-05	2.1	4:00	16:00
902	SSH24-3-26_004_B04	-	-	-	-	3.6E-05	4.9E-04	2.1	4:00	16:00
903	HI9HAF202B82QH	AT2G46760	-	D-arabinono-1,4-lactone oxidase family protein	0.0	8.6E-07	2.1E-05	2.1	8:00	16:00
904	HI9HAF202CL244	-	-	-	-	1.6E-07	3.2E-06	2.1	8:00	16:00
905	isotig02199	AT3G19990	-	-	0.0	1.1E-06	2.1E-05	2.1	8:00	16:00
906	isotig03893	AT4G20260	PCAP1	plasma-membrane associated cation-binding protein 1	2.0E-20	1.1E-09	5.9E-08	2.1	8:00	16:00
907	isotig05552	-	-	-	-	7.0E-09	3.3E-07	2.1	8:00	16:00
908	SSH12-9-64_016_H08	AT1G14590	-	Nucleotide-diphospho-sugar transferase family protein	0.0	9.3E-08	2.3E-06	2.1	8:00	16:00
909	SSH24-6-70_011_F09	AT2G03200	-	Eukaryotic aspartyl protease family protein	4.0E-13	4.2E-05	4.9E-04	2.1	8:00	16:00
910	HI9HAF203C2XJW	AT1G78060	-	Glycosyl hydrolase family protein	0.0	6.3E-06	9.7E-05	2.1	24:00	16:00
911	isotig02436	ATCG00660	RPL20	ribosomal protein L20	6.0E-25	2.3E-03	2.3E-02	2.1	24:00	16:00
912	HI9HAF202B77US	AT2G19130	-	S-locus lectin protein kinase family protein	0.0	7.1E-08	1.9E-06	2.1	4:00	20:00
913	HI9HAF202BYJIG	AT1G73500	ATMKK9	MAP kinase kinase 9	0.0	6.6E-08	1.9E-06	2.1	4:00	20:00
914	HI9HAF203CY336	AT3G56620	-	nodulin MtN21 /EamA-like transporter family protein	2.0E-43	1.5E-05	2.2E-04	2.1	4:00	20:00
915	HI9HAF202B6DLG	AT5G07050	-	nodulin MtN21 /EamA-like transporter family protein	3.0E-41	4.7E-06	6.9E-05	2.1	8:00	20:00
916	isotig00694	AT3G19430	-	late embryogenesis abundant protein-related / LEA protein-related	0.0	8.7E-04	8.1E-03	2.1	8:00	20:00
917	isotig02031	AT3G20660	AtOCT4	organic cation/carnitine transporter4	0.0	3.5E-10	2.3E-08	2.1	8:00	20:00
918	isotig06801	AT1G18900	-	Pentatricopeptide repeat (PPR) superfamily protein	0.0	1.4E-10	1.1E-08	2.1	8:00	20:00
919	Shoot-027-21	AT5G11790	NDL2	N-MYC downregulated-like 2	0.0	1.2E-10	8.8E-09	2.1	8:00	20:00
920	HI9HAF203C6AQ6	AT4G15420	-	Ubiquitin fusion degradation UFD1 family protein	0.0	6.1E-06	9.7E-05	2.1	12:00	20:00
921	HI9HAF203C6EGU	AT1G49720	ABF1	abscisic acid responsive element-binding factor 1	2.0E-35	5.7E-06	9.5E-05	2.1	12:00	20:00
922	HI9HAF203CXRUX	AT3G63310	BIL4	Bax inhibitor-1 family protein	0.0	5.7E-06	9.5E-05	2.1	12:00	20:00
923	isotig01436	AT3G25230	ROF1	rotamase FKBP 1	0.0	4.4E-06	6.9E-05	2.1	12:00	20:00
924	isotig02761	AT2G20560	-	DNAJ heat shock family protein	0.0	1.8E-07	3.3E-06	2.1	12:00	20:00
925	isotig03920	AT4G02050	STP7	sugar transporter protein 7	0.0	3.7E-05	4.9E-04	2.1	12:00	20:00
926	HI9HAF203DK77R	AT5G35970	-	P-loop containing nucleoside triphosphate hydrolases superfamily protein	0.0	1.6E-05	2.3E-04	2.1	8:00	24:00
927	isotig03834	-	-	-	-	2.2E-06	5.1E-05	2.1	8:00	24:00
928	isotig04216	AT3G30390	-	Transmembrane amino acid transporter family protein	0.0	1.4E-06	2.5E-05	2.1	8:00	24:00
929	HI9HAF202B1P2S	AT2G15130	-	Plant basic secretory protein (BSP) family protein	4.0E-42	7.8E-06	1.3E-04	2.1	12:00	24:00
930	HI9HAF202BRRV6	AT1G66330	-	senescence-associated family protein	6.0E-43	2.2E-13	1.4E-10	2.1	12:00	24:00
931	isotig02708	AT4G38810	-	Calcium-binding EF-hand family protein	0.0	1.1E-08	5.0E-07	2.1	12:00	24:00
932	isotig03493	-	-	-	-	1.4E-14	1.1E-11	2.1	12:00	24:00
933	Shoot-045-67	AT3G25950	-	TRAM, LAG1 and CLN8 (TLC) lipid-sensing domain containing protein	2.0E-34	5.1E-08	1.5E-06	2.1	12:00	24:00
934	Shoot-047-15	-	-	-	-	3.7E-03	3.1E-02	2.1	16:00	24:00
935	HI9HAF202B5PTW	-	-	-	-	4.7E-05	6.4E-04	2.0	12:00	4:00
936	HI9HAF202CFXTP	AT3G62980	TIR1	F-box/RNI-like superfamily protein	0.0	1.4E-04	1.8E-03	2.0	12:00	4:00
937	HI9HAF203DPC30	AT3G10980	-	PLAC8 family protein	0.0	2.5E-10	1.8E-08	2.0	12:00	4:00
938	isotig00163	AT5G63180	-	Pectin lyase-like superfamily protein	0.0	1.7E-06	2.7E-05	2.0	12:00	4:00
939	isotig03051	AT1G77280	-	Protein kinase protein with adenine nucleotide alpha hydrolases-like domain	0.0	1.6E-06	2.7E-05	2.0	12:00	4:00
940	Shoot-054-06	AT4G11260	ATSGT1B	phosphatase-related	3.0E-15	1.0E-05	1.5E-04	2.0	12:00	4:00
941	isotig04215	-	-	-	-	1.2E-10	8.8E-09	2.0	16:00	4:00
942	Shoot-058-14	-	-	-	-	9.1E-04	9.3E-03	2.0	16:00	4:00
943	HI9HAF202B0R9R	-	-	-	-	1.9E-05	3.3E-04	2.0	16:00	8:00
944	HI9HAF202B7NUR	AT5G23690	-	Polynucleotide adenylyltransferase family protein	0.0	8.6E-10	5.0E-08	2.0	16:00	8:00
945	HI9HAF203DNICH	AT4G01037	WTF1	Ubiquitin carboxyl-terminal hydrolase family protein	2.9E-44	1.2E-11	1.7E-09	2.0	16:00	8:00
946	isotig01340	AT5G67360	ARA12	Subtilase family protein	0.0	2.2E-06	5.1E-05	2.0	16:00	8:00
947	isotig02913	AT5G58490	-	NAD(P)-binding Rossmann-fold superfamily protein	0.0	2.8E-07	7.1E-06	2.0	16:00	8:00
948	isotig04248	-	-	-	-	1.8E-04	1.8E-03	2.0	16:00	8:00
949	isotig04842	AT3G61790	-	Protein with RING/U-box and TRAF-like domains	0.0	1.2E-07	3.1E-06	2.0	16:00	8:00
950	Shoot-030-73	AT3G54420	ATEP3	homolog of carrot EP3-3 chitinase	0.0	1.5E-04	1.8E-03	2.0	16:00	8:00
951	SSH24-5-89_002_A12	AT5G42570	-	B-cell receptor-associated 31-like	3.0E-35	6.2E-10	3.1E-08	2.0	16:00	8:00
952	isotig05620	AT1G04920	ATSPS3F	sucrose phosphate synthase 3F	0.0	4.6E-07	1.2E-05	2.0	20:00	8:00
953	Shoot-021-36_007_D05	AT5G62420	-	NAD(P)-linked oxidoreductase superfamily protein	6.0E-24	3.4E-04	4.2E-03	2.0	20:00	8:00
954	isotig02695	AT1G55020	LOX1	lipoxygenase 1	0.0	2.0E-07	6.2E-06	2.0	4:00	12:00
955	isotig04091	-	-	-	-	1.7E-05	2.3E-04	2.0	4:00	12:00
956	isotig05377	-	-	-	-	8.0E-04	8.1E-03	2.0	4:00	12:00
957	Shoot-046-89	AT1G22650	-	Plant neutral invertase family protein	0.0	2.3E-03	2.3E-02	2.0	4:00	12:00
958	HI9HAF202CFXCU	AT1G80300	NTT1	nucleotide transporter 1	0.0	3.1E-06	6.9E-05	2.0	20:00	12:00
959	HI9HAF203C1R7N	AT5G25560	-	CHY-type/CTCHY-type/RING-type Zinc finger protein	0.0	4.6E-04	5.0E-03	2.0	20:00	12:00
960	isotig00252	AT1G01390	-	UDP-Glycosyltransferase superfamily protein	2.8E-45	3.8E-03	3.1E-02	2.0	20:00	12:00
961	isotig00726	AT2G47490	ATNDT1	NAD+ transporter 1	0.0	1.9E-07	4.3E-06	2.0	20:00	12:00
962	isotig00729	AT5G43830	-	Aluminium induced protein with YGL and LRDR motifs	0.0	8.6E-05	1.1E-03	2.0	20:00	12:00
963	isotig00991	AT2G33750	ATPUP2	purine permease 2	7.0E-42	8.6E-08	2.3E-06	2.0	20:00	12:00
964	isotig01708	AT5G57030	LUT2	Lycopene beta/epsilon cyclase protein	0.0	1.3E-07	3.2E-06	2.0	20:00	12:00
965	HI9HAF202B3PO5	AT4G16370	ATOPT3	oligopeptide transporter	0.0	1.1E-03	1.3E-02	2.0	24:00	12:00
966	isotig01036	AT5G48930	HCT	hydroxycinnamoyl-CoA shikimate/quinate hydroxycinnamoyl transferase	1.0E-15	4.1E-04	4.9E-03	2.0	24:00	12:00
967	isotig01070	-	-	-	-	2.0E-09	1.1E-07	2.0	24:00	12:00
968	isotig04870	-	-	-	-	2.5E-05	4.0E-04	2.0	24:00	12:00
969	Shoot-027-69	AT1G45474	LHCA5	photosystem I light harvesting complex gene 5	1.0E-15	1.9E-05	3.3E-04	2.0	24:00	12:00
970	SSH24-9-41_002_A06	AT3G53810	-	Concanavalin A-like lectin protein kinase family protein	1.0E-19	6.5E-09	3.3E-07	2.0	24:00	12:00
971	HI9HAF202B06R1	AT4G26140	BGAL12	beta-galactosidase 12	0.0	8.8E-07	2.1E-05	2.0	4:00	16:00
972	HI9HAF203DDRHM	AT3G54140	ATPTR1	peptide transporter 1	0.0	7.8E-07	1.7E-05	2.0	4:00	16:00
973	isotig03985	AT1G75750	GASA1	GAST1 protein homolog 1	4.0E-23	1.6E-11	1.7E-09	2.0	4:00	16:00
974	isotig04575	AT1G09560	GLP5	germin-like protein 5	0.0	6.4E-06	9.7E-05	2.0	4:00	16:00
975	isotig05647	AT1G53900	-	Eukaryotic translation initiation factor 2B (eIF-2B) family protein	0.0	5.5E-09	1.9E-07	2.0	4:00	16:00
976	HI9HAF202CA00Y	AT2G46760	-	D-arabinono-1,4-lactone oxidase family protein	0.0	3.8E-08	1.4E-06	2.0	8:00	16:00
977	HI9HAF202CCDN6	AT1G62320	-	ERD (early-responsive to dehydration stress) family protein	1.4E-45	6.6E-07	1.4E-05	2.0	8:00	16:00
978	HI9HAF203DEMQW	AT3G57140	SDP1-LIKE	sugar-dependent 1-like	1.0E-30	6.5E-10	3.1E-08	2.0	8:00	16:00
979	HI9HAF203DN26Q	AT3G57140	SDP1-LIKE	sugar-dependent 1-like	3.0E-21	1.2E-09	5.9E-08	2.0	8:00	16:00
980	isotig05657	AT1G12480	OZS1	C4-dicarboxylate transporter/malic acid transport protein	0.0	1.8E-05	3.0E-04	2.0	8:00	16:00
981	isotig06410	AT1G71015	-	-	4.0E-19	4.4E-03	3.3E-02	2.0	8:00	16:00
982	HI9HAF202B1PZL	AT5G41610	ATCHX18	cation/H+ exchanger 18	0.0	1.5E-04	1.8E-03	2.0	4:00	20:00
983	HI9HAF202B4P17	AT1G04120	MRP5	multidrug resistance-associated protein 5	0.0	1.4E-08	6.6E-07	2.0	8:00	20:00
984	HI9HAF202B5RNU	AT1G16010	MGT2	magnesium transporter 2	1.0E-41	2.2E-10	1.8E-08	2.0	8:00	20:00
985	isotig04479	AT5G11790	NDL2	N-MYC downregulated-like 2	0.0	9.1E-10	5.0E-08	2.0	8:00	20:00
986	isotig04526	AT4G17550	-	Major facilitator superfamily protein	0.0	1.3E-08	6.6E-07	2.0	8:00	20:00
987	HI9HAF202CH1EU	AT3G19290	ABF4	ABRE binding factor 4	7.0E-14	5.9E-03	4.3E-02	2.0	12:00	20:00
988	isotig02365	AT3G48080	-	alpha/beta-Hydrolases superfamily protein	9.0E-26	4.9E-05	6.4E-04	2.0	12:00	20:00
989	isotig05983	AT3G57810	-	Cysteine proteinases superfamily protein	0.0	1.5E-07	3.2E-06	2.0	12:00	20:00
990	HI9HAF202CH5NT	AT5G54800	GPT1	glucose 6-phosphate/phosphate translocator 1	0.0	3.9E-03	3.1E-02	2.0	16:00	20:00
991	HI9HAF202CHWW2	AT5G35970	-	P-loop containing nucleoside triphosphate hydrolases superfamily protein	0.0	5.5E-05	6.4E-04	2.0	8:00	24:00
992	HI9HAF203DRBNZ	AT3G59010	PME61	pectin methylesterase 61	0.0	8.0E-04	8.1E-03	2.0	8:00	24:00
993	HI9HAF203DAHC8	AT2G15220	-	Plant basic secretory protein (BSP) family protein	1.0E-43	2.6E-06	6.5E-05	2.0	12:00	24:00
994	HI9HAF203DKCSP	-	-	-	-	1.8E-04	1.8E-03	2.0	12:00	24:00
995	isotig00094	AT4G24220	VEP1	NAD(P)-binding Rossmann-fold superfamily protein	0.0	9.1E-07	2.1E-05	2.0	12:00	24:00
996	isotig03468	AT2G48130	-	Bifunctional inhibitor/lipid-transfer protein/seed storage 2S albumin superfamily protein	3.0E-19	2.5E-07	6.2E-06	2.0	12:00	24:00
997	isotig03933	AT1G74890	ARR15	response regulator 15	2.0E-39	7.1E-10	3.2E-08	2.0	12:00	24:00
998	isotig03993	AT1G68490	-	-	2.0E-13	2.5E-09	1.3E-07	2.0	12:00	24:00
999	SSH12-4-91_006_C12	AT3G09700	-	Chaperone DnaJ-domain superfamily protein	6.0E-30	7.7E-06	1.3E-04	2.0	12:00	24:00

[1] The putative function of the sequences was predicted according to the highest BLASTX hits with an e-value cutoff of e-10. Only sequences that hit a unique *Arabidopsis* gene ID are listed here.
[2] p-value and false discovery rate (q-value) show the results of statistical analysis by GeneCycle [39]. Only genes with a q-value ≤ 0.05 are listed here.
[3] Fold change indicates the ratio of maximal and minimal expression in summer.
[4] The times when maximum and minimum expression were observed by microarray.
