# Supplementary material for: Clock genes and diurnal transcriptome dynamics in summer and winter in the gymnosperm Japanese cedar (Cryptomeria japonica (L.f.) D.Don)
Source: BMC Plant Biol. 2014 Nov 18;14:308. doi: 10.1186/s12870-014-0308-1 (PMC4245765; doi:10.1186/s12870-014-0308-1)
Supplement: Additional file 5: — The 556 cycling genes used for cluster analysis. 1The putative function of the sequences was predicted according to the highest BLASTX hits with an e-value cutoff of e-10. Only sequences that hit a unique Arabidopsis gene ID are listed here. 2Fold change indicates the ratio of maximal and minimal expression in summer. 3Cluster number indicates a similarity in expression patterns defined by cluster analysis. See Figure 2A for the expression pattern of each cluster. 4Putative hormone-related genes representing hormone biosynthesis and signaling pathways are listed here (ABA, abscisic acid; AUX, auxin; BR, brassinosteroids; CK, cytokinins; ETH, ethylene; GA, gibberellin; JA, jasmonic acid). [file 12870_2014_308_MOESM5_ESM.pdf]

Additional file 5. **The 556 cycling genes used for cluster analysis.**

SEQ_ID	BLASTX[1]				fold change[2]	cluster[3]	hormone related[4]
	Accession	Symbols	Description	e-value			
HI9HAF202BX81J	AT1G01060	LHY	Homeodomain-like superfamily protein	9.0E-34	82.1	1	-
HI9HAF203DOXJZ	AT5G39660	CDF2	cycling DOF factor 2	4.0E-41	17.8	1	-
isotig03449	AT5G54470	-	B-box type zinc finger family protein	2.0E-18	16.4	1	-
HI9HAF203DFI5U	AT1G07850	-	Protein of unknown function (DUF604)	2.0E-41	15.4	1	-
HI9HAF203CY2MA	AT1G73220	AtOCT1	organic cation/carnitine transporter1	0.0	9.9	1	-
isotig05247	AT3G21150	BBX32	B-box 32	5.0E-16	9.9	1	-
isotig05126	AT3G10910	-	RING/U-box superfamily protein	1.0E-12	9.0	1	-
isotig03734	AT2G21320	-	B-box zinc finger family protein	2.0E-27	7.2	1	-
Shoot-047-23	AT2G38640	-	Protein of unknown function (DUF567)	3.0E-19	7.1	1	-
isotig05855	AT3G15810	-	Protein of unknown function (DUF567)	1.0E-35	7.1	1	-
isotig04566	AT3G43660	-	Vacuolar iron transporter (VIT) family protein	2.0E-40	5.4	1	-
HI9HAF202BS1QV	AT4G25420	GA5	2-oxoglutarate (2OG) and Fe(II)-dependent oxygenase superfamily protein	0.0	5.3	1	GA
HI9HAF203DJEF7	AT5G06530	-	ABC-2 type transporter family protein	0.0	5.2	1	-
HI9HAF203C39DV	AT4G10770	ATOPT7	oligopeptide transporter 7	0.0	5.2	1	-
isotig01631	AT1G25560	TEM1	AP2/B3 transcription factor family protein	0.0	4.5	1	-
isotig01419	AT2G13610	-	ABC-2 type transporter family protein	0.0	4.5	1	-
HI9HAF203CYNP6	AT4G26590	ATOPT5	oligopeptide transporter 5	2.0E-43	4.4	1	-
Shoot-017-36_007_D05	AT1G18660	-	zinc finger (C3HC4-type RING finger) family protein	2.0E-40	4.4	1	-
isotig00857	AT5G38710	-	Methylenetetrahydrofolate reductase family protein	0.0	4.2	1	-
HI9HAF202BV9JR	AT4G10490	-	2-oxoglutarate (2OG) and Fe(II)-dependent oxygenase superfamily protein	0.0	4.2	1	-
Shoot-052-12	AT2G40460	-	Major facilitator superfamily protein	0.0	4.2	1	-
HI9HAF203DKAPN	AT5G46050	ATPTR3	peptide transporter 3	0.0	4.1	1	-
isotig00619	AT3G56630	CYP94D2	cytochrome P450, family 94, subfamily D, polypeptide 2	0.0	4.0	1	-
Shoot-021-79_014_G10	AT1G75000	-	GNS1/SUR4 membrane protein family	0.0	4.0	1	-
HI9HAF202B43TO	AT2G26650	AKT1	K+ transporter 1	0.0	3.9	1	-
isotig00856	AT3G30775	ERD5	Methylenetetrahydrofolate reductase family protein	2.0E-17	3.8	1	-
HI9HAF202CEBY0	AT5G46240	KAT1	potassium channel in Arabidopsis thaliana 1	0.0	3.8	1	-
Shoot-046-67	AT2G03140	-	alpha/beta-Hydrolases superfamily protein	3.0E-34	3.8	1	-
isotig02675	AT2G45570	CYP76C2	cytochrome P450, family 76, subfamily C, polypeptide 2	0.0	3.7	1	-
isotig04121	AT5G24120	SIGE	sigma factor E	0.0	3.7	1	-
HI9HAF203CY149	AT4G34760	-	SAUR-like auxin-responsive protein family	6.0E-23	3.7	1	AUX
isotig01742	AT4G21990	3-Apr	APS reductase 3	0.0	3.6	1	-
isotig00398	AT5G13870	EXGT-A4	xyloglucan endotransglucosylase/hydrolase 5	0.0	3.6	1	-
HI9HAF202CKRK8	AT5G03555	-	permease, cytosine/purines, uracil, thiamine, allantoin family protein	0.0	3.5	1	-
HI9HAF202B7HJR	AT3G16857	ARR1	response regulator 1	8.0E-13	3.5	1	CK
isotig02073	AT3G48990	-	AMP-dependent synthetase and ligase family protein	0.0	3.5	1	-
isotig01008	AT5G56300	GAMT2	gibberellic acid methyltransferase 2	0.0	3.5	1	GA
Shoot-059-56	AT5G16010	-	3-oxo-5-alpha-steroid 4-dehydrogenase family protein	2.0E-36	3.5	1	-
isotig04967	AT3G56290	-	-	7.0E-45	3.5	1	-
Shoot-020-01_001_A01	AT1G61820	BGLU46	beta glucosidase 46	0.0	3.5	1	-
isotig04004	AT3G51860	CAX3	cation exchanger 3	0.0	3.4	1	-
isotig06033	AT4G35100	PIP3	plasma membrane intrinsic protein 3	4.0E-34	3.3	1	-
isotig06792	AT1G01420	UGT72B3	UDP-glucosyl transferase 72B3	5.0E-13	3.3	1	-
Shoot-046-32	AT4G24540	AGL24	AGAMOUS-like 24	5.0E-32	3.3	1	-
HI9HAF203DBT8H	AT1G77120	ADH1	alcohol dehydrogenase 1	0.0	3.2	1	-
Shoot-001-71_013_G09	AT4G32000	-	Protein kinase superfamily protein	0.0	3.2	1	-
HI9HAF203DLL9G	AT5G24790	-	Protein of unknown function, DUF599	9.0E-42	3.2	1	-
HI9HAF202BWSJY	AT5G60900	RLK1	receptor-like protein kinase 1	0.0	3.2	1	-
SSH24-5-91_006_C12	AT5G38212	-	Potential natural antisense gene, locus overlaps with AT5G38210	3.0E-12	3.2	1	-
HI9HAF202B0S1U	AT5G05600	-	2-oxoglutarate (2OG) and Fe(II)-dependent oxygenase superfamily protein	0.0	3.1	1	-
HI9HAF203DM1OJ	AT3G16520	UGT88A1	UDP-glucosyl transferase 88A1	5.0E-43	3.1	1	-
HI9HAF203CYY76	AT2G18010	-	SAUR-like auxin-responsive protein family	4.0E-14	3.1	1	AUX
HI9HAF202B8ET8	AT3G51895	SULTR3;1	sulfate transporter 3;1	0.0	3.0	1	-
isotig01416	AT4G05200	CRK25	cysteine-rich RLK (RECEPTOR-like protein kinase) 25	0.0	3.0	1	-
HI9HAF203DIAPG	AT1G67710	ARR11	response regulator 11	9.0E-29	3.0	1	CK
isotig02964	AT4G16260	-	Glycosyl hydrolase superfamily protein	0.0	3.0	1	-
isotig01511	AT2G16570	ATASE	GLN phosphoribosyl pyrophosphate amidotransferase 1	0.0	2.9	1	-
HI9HAF203C89CZ	AT1G76990	ACR3	ACT domain repeat 3	0.0	2.9	1	-
isotig00765	AT4G27250	-	NAD(P)-binding Rossmann-fold superfamily protein	1.0E-41	2.9	1	-
isotig02115	AT5G22090	-	Protein of unknown function (DUF3049)	3.0E-15	2.9	1	-
HI9HAF202B9FSD	AT3G57630	-	exostosin family protein	0.0	2.8	1	-
HI9HAF202CA5QQ	AT1G66340	ETR1	Signal transduction histidine kinase, hybrid-type, ethylene sensor	4.0E-27	2.8	1	ETH
HI9HAF202BWLCD	AT4G02900	-	ERD (early-responsive to dehydration stress) family protein	0.0	2.8	1	-
HI9HAF203C8SUM	AT1G26780	MYB117	myb domain protein 117	0.0	2.8	1	-
HI9HAF202BXWDV	AT1G74950	JAZ2	TIFY domain/Divergent CCT motif family protein	4.0E-16	2.8	1	JA
HI9HAF203DMC7D	AT5G49720	ATGH9A1	glycosyl hydrolase 9A1	0.0	2.7	1	-
HI9HAF202B325D	AT2G42980	-	Eukaryotic aspartyl protease family protein	0.0	2.7	1	-
isotig03266	AT5G14700	-	NAD(P)-binding Rossmann-fold superfamily protein	0.0	2.7	1	-
HI9HAF203DCF70	AT2G21220	-	SAUR-like auxin-responsive protein family	6.0E-20	2.7	1	AUX
isotig04637	AT1G78370	ATGSTU20	glutathione S-transferase TAU 20	0.0	2.7	1	-
HI9HAF203C165B	AT1G59870	PEN3	ABC-2 and Plant PDR ABC-type transporter family protein	2.0E-44	2.7	1	-
isotig04066	AT3G54850	ATPUB14	plant U-box 14	0.0	2.6	1	-
isotig01958	AT4G38060	-	-	1.0E-10	2.6	1	-
HI9HAF202B0AKD	AT3G21620	-	ERD (early-responsive to dehydration stress) family protein	0.0	2.6	1	-
HI9HAF202CKKIQ	AT3G43120	-	SAUR-like auxin-responsive protein family	5.0E-25	2.6	1	AUX
HI9HAF202B13J6	AT5G56220	-	P-loop containing nucleoside triphosphate hydrolases superfamily protein	1.4E-45	2.6	1	-
HI9HAF203DKT1M	AT5G42760	-	Leucine carboxyl methyltransferase	0.0	2.6	1	-
HI9HAF202BWYA2	AT2G32950	COP1	Transducin/WD40 repeat-like superfamily protein	0.0	2.6	1	-
Shoot-048-02	AT4G28670	-	Protein kinase family protein with domain of unknown function (DUF26)	2.0E-15	2.6	1	-
isotig00267	AT1G21460	SWEET1	Nodulin MtN3 family protein	0.0	2.6	1	-
Shoot-048-21	AT5G01810	CIPK15	CBL-interacting protein kinase 15	1.0E-34	2.5	1	-
isotig04173	AT4G34980	SLP2	subtilisin-like serine protease 2	0.0	2.5	1	-
Shoot-053-22	AT5G62360	-	Plant invertase/pectin methylesterase inhibitor superfamily protein	5.0E-12	2.5	1	-
isotig00662	AT1G75460	-	ATP-dependent protease La (LON) domain protein	0.0	2.5	1	-
HI9HAF203DDY6U	AT5G64120	-	Peroxidase superfamily protein	3.0E-22	2.5	1	-
HI9HAF203DP6B2	AT1G51170	-	Protein kinase superfamily protein	1.0E-41	2.4	1	-
Shoot-012-42_004_B06	AT5G22860	-	Serine carboxypeptidase S28 family protein	0.0	2.4	1	-
SSH12-1-45_010_E06	AT4G26080	ABI1	Protein phosphatase 2C family protein	3.0E-40	2.4	1	ABA
isotig00236	AT4G17500	ATERF-1	ethylene responsive element binding factor 1	9.0E-27	2.4	1	-
isotig06083	AT2G05620	PGR5	proton gradient regulation 5	9.0E-30	2.4	1	-
HI9HAF203DFFSV	AT5G08380	AtAGAL1	alpha-galactosidase 1	0.0	2.4	1	-
Shoot-005-36_007_D05	AT2G31820	-	Ankyrin repeat family protein	1.0E-32	2.4	1	-
isotig06086	AT4G24380	-		0.0	2.4	1	-
isotig04194	AT1G17100	-	SOUL heme-binding family protein	0.0	2.4	1	-
HI9HAF203C9GCX	AT1G18900	-	Pentatricopeptide repeat (PPR) superfamily protein	0.0	2.4	1	-
isotig04693	AT5G62020	AT-HSFB2A	heat shock transcription factor B2A	2.0E-44	2.4	1	-
HI9HAF203C3BRH	AT2G40940	ERS1	ethylene response sensor 1	2.0E-38	2.4	1	ETH
HI9HAF203C1Y23	AT4G17550	-	Major facilitator superfamily protein	0.0	2.4	1	-
HI9HAF202BWELV	AT5G15250	FTSH6	FTSH protease 6	0.0	2.4	1	-
HI9HAF203C56D5	AT1G32090	-	early-responsive to dehydration stress protein (ERD4)	0.0	2.3	1	-

isotig04779	AT2G41510	ATCKX1	cytokinin oxidase/dehydrogenase 1	0.0	2.3	1	CK
Shoot-006-51_005_C07	AT3G19990	–	–	0.0	2.3	1	–
Shoot-017-37_009_E05	AT2G41000	–	Chaperone DnaJ-domain superfamily protein	9.0E-21	2.3	1	–
Shoot-055-62	AT4G34350	CLB6	4-hydroxy-3-methylbut-2-enyl diphosphate reductase	0.0	2.3	1	–
isotig02986	AT1G52770	–	Phototropic-responsive NPH3 family protein	0.0	2.3	1	–
isotig01735	AT1G79900	ATMBAC2	Mitochondrial substrate carrier family protein	0.0	2.3	1	–
isotig06357	AT3G10020	–	–	4.0E-13	2.3	1	–
HI9HAF203DLG5Q	AT4G22990	–	Major Facilitator Superfamily with SPX (SYG1/Pho81/XPR1) domain-containing protein	1.0E-43	2.3	1	–
isotig05919	AT4G19450	–	Major facilitator superfamily protein	3.0E-28	2.3	1	–
HI9HAF203DNGBQ	AT1G67720	–	Leucine-rich repeat protein kinase family protein	5.0E-43	2.3	1	–
HI9HAF203CX03E	AT1G33440	–	Major facilitator superfamily protein	0.0	2.3	1	–
HI9HAF202CFUEA	AT5G63810	BGAL10	beta-galactosidase 10	0.0	2.2	1	–
HI9HAF202CC878	AT3G28860	ATMDR1	ATP binding cassette subfamily B19	0.0	2.2	1	–
isotig06075	AT3G43270	–	Plant invertase/pectin methylesterase inhibitor superfamily	5.0E-44	2.2	1	–
isotig03358	AT5G64260	EXL2	EXORDIUM like 2	0.0	2.2	1	–
isotig01338	AT1G56120	–	Leucine-rich repeat transmembrane protein kinase	0.0	2.2	1	–
Shoot-021-64_016_H08	AT1G22400	UGT85A1	UDP-Glycosyltransferase superfamily protein	2.0E-24	2.2	1	CK
HI9HAF202CBU21	AT1G49720	ABF1	abscisic acid responsive element-binding factor 1	1.0E-35	2.2	1	ABA
HI9HAF202BSDV2	AT2G37050	–	Leucine-rich repeat protein kinase family protein	0.0	2.2	1	–
isotig01692	AT3G11670	DGD1	UDP-Glycosyltransferase superfamily protein	0.0	2.2	1	–
isotig04053	AT1G78020	–	Protein of unknown function (DUF581)	3.0E-14	2.2	1	–
isotig03104	AT5G45820	CIPK20	CBL-interacting protein kinase 20	0.0	2.2	1	–
isotig05959	AT3G19950	–	RING/U-box superfamily protein	1.0E-37	2.2	1	–
Shoot-039-05	AT2G02061	–	Nucleotide-diphospho-sugar transferase family protein	0.0	2.2	1	–
HI9HAF203DROKL	AT1G02270	–	Calcium-binding endonuclease/exonuclease/phosphatase family	0.0	2.2	1	–
HI9HAF202B9Y9X	AT5G06460	ATUBA2	ubiquitin activating enzyme 2	0.0	2.2	1	–
HI9HAF202B69K2	AT5G54250	CNGC4	cyclic nucleotide-gated cation channel 4	0.0	2.2	1	–
Shoot-057-59	AT1G17940	–	Endosomal targeting BRO1-like domain-containing protein	4.0E-25	2.2	1	–
isotig05258	AT1G72890	–	Disease resistance protein (TIR-NBS class)	5.0E-28	2.2	1	–
HI9HAF202BYJIG	AT1G73500	ATMKK9	MAP kinase kinase 9	0.0	2.1	1	–
HI9HAF203C6AQ6	AT4G15420	–	Ubiquitin fusion degradation UFD1 family protein	0.0	2.1	1	–
SSH12-9-64_016_H08	AT1G14590	–	Nucleotide-diphospho-sugar transferase family protein	0.0	2.1	1	–
HI9HAF202B6DLG	AT5G07050	–	nodulin MtN21 /EamA-like transporter family protein	3.0E-41	2.1	1	–
isotig04216	AT3G30390	–	Transmembrane amino acid transporter family protein	0.0	2.1	1	–
HI9HAF203CY336	AT3G56620	–	nodulin MtN21 /EamA-like transporter family protein	2.0E-43	2.1	1	–
HI9HAF202B82QH	AT2G46760	–	D-arabinono-1,4-lactone oxidase family protein	0.0	2.1	1	–
HI9HAF203DKTU2	AT1G31120	KUP10	K+ uptake permease 10	0.0	2.1	1	–
isotig04891	AT1G07710	–	Ankyrin repeat family protein	1.0E-40	2.1	1	–
SSH24-6-70_011_F09	AT2G03200	–	Eukaryotic aspartyl protease family protein	4.0E-13	2.1	1	–
HI9HAF202B77US	AT2G19130	–	S-locus lectin protein kinase family protein	0.0	2.1	1	–
isotig00694	AT3G19430	–	late embryogenesis abundant protein-related / LEA protein-related	0.0	2.1	1	–
Shoot-027-21	AT5G11790	NDL2	N-MYC downregulated-like 2	0.0	2.1	1	–
isotig03893	AT4G20260	PCAP1	plasma-membrane associated cation-binding protein 1	2.0E-20	2.1	1	–
isotig02031	AT3G20660	AtOCT4	organic cation/carnitine transporter4	0.0	2.1	1	–
HI9HAF203DDRHM	AT3G54140	ATPTR1	peptide transporter 1	0.0	2.0	1	–
HI9HAF202B06R1	AT4G26140	BGAL12	beta-galactosidase 12	0.0	2.0	1	–
HI9HAF202B5RNU	AT1G16010	MGT2	magnesium transporter 2	1.0E-41	2.0	1	–
HI9HAF202CCDN6	AT1G62320	–	ERD (early-responsive to dehydration stress) family protein	1.4E-45	2.0	1	–
HI9HAF202B1PZL	AT5G41610	ATCHX18	cation/H+ exchanger 18	0.0	2.0	1	–
isotig03985	AT1G75750	GASA1	GAST1 protein homolog 1	4.0E-23	2.0	1	–
isotig05647	AT1G53900	–	Eukaryotic translation initiation factor 2B (eIF-2B) family protein	0.0	2.0	1	–
isotig05983	AT3G57810	–	Cysteine proteinases superfamily protein	0.0	2.0	1	–
HI9HAF203DRBNZ	AT3G59010	PME61	pectin methylesterase 61	0.0	2.0	1	–
HI9HAF202CHWW2	AT5G35970	–	P-loop containing nucleoside triphosphate hydrolases superfamily protein	0.0	2.0	1	–
isotig05657	AT1G12480	OZS1	C4-dicarboxylate transporter/malic acid transport protein	0.0	2.0	1	–
HI9HAF203DEMQW	AT3G57140	SDP1-LIKE	sugar-dependent 1-like	1.0E-30	2.0	1	–
HI9HAF202B4P17	AT1G04120	MRP5	multidrug resistance-associated protein 5	0.0	2.0	1	–
isotig06410	AT1G71015	–	–	4.0E-19	2.0	1	–
isotig01218	AT5G02810	PRR7	pseudo-response regulator 7	0.0	65.8	2	–
HI9HAF203DC17O	AT5G59720	HSP18.2	heat shock protein 18.2	0.0	64.2	2	–
isotig04596	AT5G12020	HSP17.6II	17.6 kDa class II heat shock protein	4.0E-41	52.3	2	–
isotig05853	AT3G46230	ATHSP17.4	heat shock protein 17.4	0.0	20.0	2	–
isotig00466	AT5G52640	HSP81-1	heat shock protein 90.1	0.0	18.9	2	–
isotig04474	AT4G14690	ELIP2	Chlorophyll A-B binding family protein	4.0E-42	17.2	2	–
HI9HAF203C266F	AT3G19270	CYP707A4	cytochrome P450, family 707, subfamily A, polypeptide 4	9.0E-41	12.8	2	ABA
HI9HAF203DEENJ	AT5G02500	HSC70-1	heat shock cognate protein 70-1	0.0	9.8	2	–
isotig04041	AT4G27670	HSP21	heat shock protein 21	0.0	8.7	2	–
isotig03433	AT3G54500	–	–	2.0E-30	8.6	2	–
HI9HAF202BXV4N	AT2G32120	HSP70T-2	heat-shock protein 70T-2	2.0E-42	8.5	2	–
isotig06221	AT5G45340	CYP707A3	cytochrome P450, family 707, subfamily A, polypeptide 3	0.0	8.0	2	ABA
Shoot-024-80	AT5G64170	–	dentin sialophosphoprotein-related	8.0E-19	7.9	2	–
isotig04546	AT5G51440	–	HSP20-like chaperones superfamily protein	1.0E-30	7.6	2	–
HI9HAF203CYISG	AT4G29520	–	–	0.0	7.1	2	–
HI9HAF202B2W3I	AT1G56410	ERD2	heat shock protein 70 (Hsp 70) family protein	0.0	6.9	2	–
isotig05830	AT4G03290	–	EF hand calcium-binding protein family	6.0E-17	6.6	2	–
isotig03680	AT5G55250	IAMT1	IAA carboxylmethyltransferase 1	3.0E-29	6.5	2	AUX
isotig04915	AT2G47180	AtGolS1	galactinol synthase 1	0.0	6.4	2	–
HI9HAF202CI8OC	AT1G20190	ATEXPA11	expansin 11	0.0	6.0	2	–
isotig02108	AT5G40390	SIP1	Raffinose synthase family protein	0.0	5.6	2	–
isotig01316	AT3G12580	HSP70	heat shock protein 70	0.0	5.1	2	–
isotig06624	AT4G14830	HSP1	–	7.0E-14	5.0	2	–
isotig05425	AT3G08970	ATERDJ3A	DNAJ heat shock N-terminal domain-containing protein	1.0E-43	4.9	2	–
Shoot-007-70_011_F09	AT3G62600	ATERDJ3B	DNAJ heat shock family protein	0.0	4.8	2	–
isotig02051	AT5G64510	TIN1	–	0.0	4.5	2	–
isotig05611	AT5G20720	CPN20	chaperonin 20	0.0	4.4	2	–
Shoot-005-74_004_B10	AT2G12646	–	PLATZ transcription factor family protein	0.0	4.4	2	–
HI9HAF202CBT07	AT5G09970	CYP78A7	cytochrome P450, family 78, subfamily A, polypeptide 7	1.0E-42	4.3	2	–
HI9HAF202CAXIE	AT5G09590	MTHSC70-2	mitochondrial HSO70 2	0.0	4.2	2	–
HI9HAF202BYAVK	AT2G29380	HAI3	highly ABA-induced PP2C gene 3	6.0E-17	4.2	2	ABA
isotig00882	AT1G23740	–	Oxidoreductase, zinc-binding dehydrogenase family protein	0.0	3.9	2	–
isotig04935	AT3G09640	APX2	ascorbate peroxidase 2	0.0	3.8	2	–
isotig04710	AT3G07090	–	PPPDE putative thiol peptidase family protein	0.0	3.8	2	–
Shoot-023-14	AT3G22840	ELIP1	Chlorophyll A-B binding family protein	1.4E-45	3.8	2	–
Shoot-006-27_006_C04	AT5G17540	–	HXXXD-type acyl-transferase family protein	1.0E-21	3.7	2	–
isotig01574	AT1G27680	APL2	ADPGLC-PPase large subunit	0.0	3.6	2	–
Shoot-004-26_004_B04	AT4G22740	–	glycine-rich protein	3.0E-25	3.6	2	–
isotig02665	AT3G09350	Fes1A	Fes1A	0.0	3.6	2	–
isotig06632	AT3G24500	MBF1C	multiprotein bridging factor 1C	0.0	3.6	2	–
HI9HAF202B3WGH	AT5G03160	ATP58IPK	homolog of mamallian P58IPK	9.0E-41	3.6	2	–
isotig01805	AT5G28540	BIP1	heat shock protein 70 (Hsp 70) family protein	0.0	3.5	2	–

Shoot-012-62_012_F08	AT5G11260	HY5	Basic-leucine zipper (bZIP) transcription factor family protein	3.0E-21	3.5	2	-
isotig01294	AT5G42020	BIP	Heat shock protein 70 (Hsp 70) family protein	0.0	3.4	2	-
isotig03847	AT1G26770	ATEXPA10	expansin A10	0.0	3.4	2	-
HI9HAF203C8QS4	AT4G30780	-	-	4.0E-42	3.4	2	-
SSH12-7-83_005_C11	AT1G44760	-	Adenine nucleotide alpha hydrolases-like superfamily protein	4.0E-30	3.4	2	-
isotig00868	AT2G26710	BAS1	Cytochrome P450 superfamily protein	0.0	3.4	2	BR
isotig06422	AT2G47710	-	Adenine nucleotide alpha hydrolases-like superfamily protein	0.0	3.4	2	-
HI9HAF202B4YW1	AT2G26910	PDR4	pleiotropic drug resistance 4	0.0	3.3	2	-
HI9HAF202BVBDX	AT2G41190	-	Transmembrane amino acid transporter family protein	0.0	3.2	2	-
HI9HAF203DDGDD	AT3G55550	-	Concanavalin A-like lectin protein kinase family protein	0.0	3.2	2	-
isotig05870	AT3G14440	NCED3	nine-cis-epoxycarotenoid dioxygenase 3	0.0	3.1	2	ABA
HI9HAF203DIBLL	AT3G50660	DWF4	Cytochrome P450 superfamily protein	0.0	3.1	2	BR
HI9HAF202BWF9P	AT1G14360	ATUTR3	UDP-galactose transporter 3	0.0	3.0	2	-
HI9HAF202B5WN1	AT1G78390	NCED9	nine-cis-epoxycarotenoid dioxygenase 9	2.0E-32	3.0	2	ABA
isotig03850	AT5G01410	PDX1	Aldolase-type TIM barrel family protein	0.0	2.9	2	-
Shoot-050-47	AT3G58680	MBF1B	multiprotein bridging factor 1B	1.0E-42	2.9	2	-
isotig04760	AT2G37970	SOUL-1	SOUL heme-binding family protein	0.0	2.9	2	-
HI9HAF202B0AVA	AT3G23990	HSP60	heat shock protein 60	0.0	2.9	2	-
HI9HAF203DE338	AT3G25230	ROF1	rotamase FKBP 1	0.0	2.9	2	-
isotig02916	AT2G45380	-	-	3.0E-27	2.9	2	-
isotig03291	AT5G05340	-	Peroxidase superfamily protein	0.0	2.8	2	-
isotig01223	AT4G24190	SHD	Chaperone protein htpG family protein	0.0	2.8	2	-
HI9HAF202BUGL9	AT5G15450	APG6	casein lytic proteinase B3	0.0	2.8	2	-
isotig02484	AT5G19875	-	-	3.0E-22	2.8	2	-
HI9HAF203DDOCS	AT1G76690	OPR2	12-oxophytodienoate reductase 2	0.0	2.8	2	-
isotig00631	AT5G41040	-	HXXXD-type acyl-transferase family protein	0.0	2.7	2	-
HI9HAF202B80AD	AT4G29130	ATHXK1	hexokinase 1	0.0	2.7	2	-
HI9HAF202CAZ5B	AT3G24120	-	Homeodomain-like superfamily protein	1.0E-42	2.7	2	-
isotig01277	AT5G56030	HSP81-2	heat shock protein 81-2	0.0	2.7	2	-
isotig05450	AT1G27330	-	Ribosome associated membrane protein RAMP4	1.0E-16	2.6	2	-
HI9HAF203C9D5H	AT4G16660	-	heat shock protein 70 (Hsp 70) family protein	0.0	2.6	2	-
Shoot-003-39_013_G05	AT5G51260	-	HAD superfamily, subfamily IIIB acid phosphatase	0.0	2.6	2	-
Shoot-013-23_013_G03	AT2G01770	VIT1	vacuolar iron transporter 1	0.0	2.6	2	-
isotig04626	AT1G22270	-	Trm112p-like protein	1.0E-41	2.5	2	-
isotig02598	AT3G14200	-	Chaperone DnaJ-domain superfamily protein	4.0E-28	2.5	2	-
HI9HAF202B7X98	AT1G64390	AtGH9C2	glycosyl hydrolase 9C2	0.0	2.5	2	-
isotig05046	AT5G39790	-	5'-AMP-activated protein kinase-related	3.0E-19	2.5	2	-
isotig02091	AT1G74310	ATHSP101	heat shock protein 101	0.0	2.5	2	-
HI9HAF202BWGZ3	AT4G02780	GA1	Terpenoid cyclases/Protein prenyltransferases superfamily protein	1.0E-28	2.4	2	GA
isotig06003	AT1G07350	-	RNA-binding (RRM/RBD/RNP motifs) family protein	2.0E-37	2.4	2	-
HI9HAF202CGOFA	AT3G51280	-	Tetratricopeptide repeat (TPR)-like superfamily protein	0.0	2.4	2	-
isotig00164	AT4G24780	-	Pectin lyase-like superfamily protein	0.0	2.4	2	-
HI9HAF202BXP2W	AT5G58110	-	chaperone binding;ATPase activators	0.0	2.4	2	-
HI9HAF203DQMHV	AT2G04030	CR88	Chaperone protein htpG family protein	0.0	2.4	2	-
HI9HAF202B4VT8	AT2G25140	HSP98.7	casein lytic proteinase B4	0.0	2.3	2	-
Shoot-006-12_008_D02	AT3G13677	-	-	9.0E-12	2.3	2	-
SSH24-4-48_016_H06	AT5G24930	ATCOL4	CONSTANS-like 4	5.0E-37	2.3	2	-
HI9HAF202CFVAC	AT4G11050	AtGH9C3	glycosyl hydrolase 9C3	0.0	2.3	2	-
HI9HAF202BZIAY	AT3G26140	-	Cellulase (glycosyl hydrolase family 5) protein	0.0	2.3	2	-
Shoot-057-41	AT2G33700	-	Protein phosphatase 2C family protein	5.0E-19	2.3	2	-
isotig01544	AT2G36870	XTH32	xyloglucan endotransglucosylase/hydrolase 32	0.0	2.3	2	-
isotig03034	AT5G57660	ATCOL5	CONSTANS-like 5	1.0E-28	2.3	2	-
isotig06277	AT3G07770	Hsp89.1	HEAT SHOCK PROTEIN 89.1	0.0	2.3	2	-
HI9HAF203DNML3	AT5G60020	LAC17	laccase 17	0.0	2.2	2	-
isotig01370	AT2G46950	CYP709B2	cytochrome P450, family 709, subfamily B, polypeptide 2	0.0	2.2	2	-
HI9HAF203DMBYB	AT3G25500	AFH1	formin homology 1	0.0	2.2	2	-
isotig00315	AT3G44190	-	FAD/NAD(P)-binding oxidoreductase family protein	9.0E-38	2.2	2	-
HI9HAF203DG5DR	AT5G05390	LAC12	laccase 12	4.0E-43	2.2	2	-
isotig03953	AT4G14130	XTR7	xyloglucan endotransglucosylase/hydrolase 15	0.0	2.2	2	-
isotig05913	AT5G57480	-	P-loop containing nucleoside triphosphate hydrolases superfamily protein	0.0	2.2	2	-
HI9HAF202CBN5I	AT5G24270	SOS3	Calcium-binding EF-hand family protein	0.0	2.2	2	-
isotig03321	AT5G01880	-	RING/U-box superfamily protein	1.0E-29	2.2	2	-
Shoot-057-57	AT1G18180	-	Protein of unknown function (DUF1295)	0.0	2.2	2	-
HI9HAF202B8QRS	AT4G26780	AR192	Co-chaperone GrpE family protein	0.0	2.2	2	-
HI9HAF203C8BTF	AT1G55910	ZIP11	zinc transporter 11 precursor	8.0E-41	2.2	2	-
HI9HAF202CFFXU	AT1G17870	ATEGY3	ethylene-dependent gravitropism-deficient and yellow-green-like 3	0.0	2.1	2	-
Shoot-045-67	AT3G25950	-	TRAM, LAG1 and CLN8 (TLC) lipid-sensing domain containing protein	2.0E-34	2.1	2	-
isotig04350	AT2G40610	ATEXPA8	expansin A8	0.0	2.1	2	-
isotig03920	AT4G02050	STP7	sugar transporter protein 7	0.0	2.1	2	-
HI9HAF203C3DTE	AT1G06440	-	Ubiquitin carboxyl-terminal hydrolase family protein	0.0	2.1	2	-
HI9HAF202B1P2S	AT2G15130	-	Plant basic secretory protein (BSP) family protein	4.0E-42	2.1	2	-
Shoot-020-30_012_F04	AT5G36930	-	Disease resistance protein (TIR-NBS-LRR class) family	5.0E-25	2.1	2	-
HI9HAF203C71ZO	AT5G19530	ACL5	S-adenosyl-L-methionine-dependent methyltransferases superfamily protein	1.0E-38	2.1	2	-
isotig02708	AT4G38810	-	Calcium-binding EF-hand family protein	0.0	2.1	2	-
isotig02130	AT1G04980	ATPDIL2-2	PDI-like 2-2	0.0	2.1	2	-
HI9HAF203CXRUX	AT3G63310	BIL4	Bax inhibitor-1 family protein	0.0	2.1	2	-
HI9HAF202BVZYJ	AT5G05350	-	PLAC8 family protein	4.2E-45	2.1	2	-
isotig02761	AT2G20560	-	DNAJ heat shock family protein	0.0	2.1	2	-
HI9HAF202BRRV6	AT1G66330	-	senescence-associated family protein	6.0E-43	2.1	2	-
HI9HAF202BV7C9	AT2G30000	-	PHF5-like protein	0.0	2.1	2	-
HI9HAF202B2WVG	AT3G26810	AFB2	auxin signaling F-box 2	6.0E-44	2.1	2	-
isotig00094	AT4G24220	VEP1	NAD(P)-binding Rossmann-fold superfamily protein	0.0	2.0	2	-
HI9HAF202CH5NT	AT5G54800	GPT1	glucose 6-phosphate/phosphate translocator 1	0.0	2.0	2	-
isotig03993	AT1G68490	-	-	2.0E-13	2.0	2	-
Shoot-054-06	AT4G11260	ATSGT1B	phosphatase-related	3.0E-15	2.0	2	-
isotig03468	AT2G48130	-	Bifunctional inhibitor/lipid-transfer protein/seed storage 2S albumin superfamily protein	3.0E-19	2.0	2	-
isotig03051	AT1G77280	-	Protein kinase protein with adenine nucleotide alpha hydrolases-like domain	0.0	2.0	2	-
HI9HAF203DPC30	AT3G10980	-	PLAC8 family protein	0.0	2.0	2	-
isotig02365	AT3G48080	-	alpha/beta-Hydrolases superfamily protein	9.0E-26	2.0	2	-
isotig00163	AT5G63180	-	Pectin lyase-like superfamily protein	0.0	2.0	2	-
HI9HAF202CFXTP	AT3G62980	TIR1	F-box/RNI-like superfamily protein	0.0	2.0	2	AUX
HI9HAF203DAHC8	AT2G15220	-	Plant basic secretory protein (BSP) family protein	1.0E-43	2.0	2	-
HI9HAF202CH1EU	AT3G19290	ABF4	ABRE binding factor 4	7.0E-14	2.0	2	ABA
isotig03933	AT1G74890	ARR15	response regulator 15	2.0E-39	2.0	2	CK
SSH12-4-91_006_C12	AT3G09700	-	Chaperone DnaJ-domain superfamily protein	6.0E-30	2.0	2	-
HI9HAF202CA57Y	AT1G22770	GI	gigantea protein (GI)	0.0	54.8	3	-
isotig02783	AT2G26150	ATHSFA2	heat shock transcription factor A2	0.0	26.7	3	-
SSH24-7-44_008_D06	AT4G14622	CPuORF60	conserved peptide upstream open reading frame 60	4.0E-32	14.2	3	-
isotig00762	AT4G25000	ATAMY1	alpha-amylase-like	0.0	12.4	3	-
isotig04131	AT4G11650	ATOSM34	osmotin 34	0.0	10.8	3	-

isotig04059	AT2G21100	–	Disease resistance-responsive (dirigent-like protein) family protein	1.4E-45	9.1	3	–
isotig05544	AT2G45580	CYP76C3	cytochrome P450, family 76, subfamily C, polypeptide 3	3.0E-39	8.8	3	–
HI9HAF202BX0CC	AT1G64890	–	Major facilitator superfamily protein	5.0E-44	8.2	3	–
isotig00878	AT1G48130	ATPER1	1-cysteine peroxiredoxin 1	0.0	8.2	3	–
isotig00772	AT1G71980	–	Protease-associated (PA) RING/U-box zinc finger family protein	0.0	7.4	3	–
Shoot-049-21	AT1G76940	–	RNA-binding (RRM/RBD/RNP motifs) family protein	4.0E-27	6.0	3	–
isotig02040	AT5G65280	GCL1	GCR2-like 1	0.0	5.8	3	–
HI9HAF203DR7YA	AT1G76140	–	Prolyl oligopeptidase family protein	0.0	5.7	3	–
Shoot-048-89	AT4G21960	PRXR1	Peroxidase superfamily protein	0.0	5.6	3	–
HI9HAF203DMYYA	AT1G20380	–	Prolyl oligopeptidase family protein	2.9E-44	5.5	3	–
HI9HAF203C2WNO	AT5G60100	PRR3	pseudo-response regulator 3	9.0E-42	5.4	3	–
isotig05762	AT2G45560	CYP76C1	cytochrome P450, family 76, subfamily C, polypeptide 1	0.0	5.3	3	–
HI9HAF202B99QE	AT5G13930	CHS	Chalcone and stilbene synthase family protein	7.0E-45	5.2	3	–
Shoot-044-15	AT5G61380	TOC1	CCT motif -containing response regulator protein	1.0E-24	5.1	3	–
HI9HAF203DAPBQ	AT2G36190	AtcwINV4	cell wall invertase 4	3.0E-42	5.1	3	–
isotig06573	AT2G30870	ATGSTF10	glutathione S-transferase PHI 10	0.0	4.8	3	–
HI9HAF202BTYDK	AT2G38310	PYL4	PYR1-like 4	0.0	4.8	3	ABA
HI9HAF203DKAG0	AT1G75450	CKX5	cytokinin oxidase 5	0.0	4.8	3	CK
HI9HAF202BSJ1B	AT2G34650	PID	Protein kinase superfamily protein	8.0E-43	4.7	3	–
isotig02943	AT1G62500	–	Bifunctional inhibitor/lipid-transfer protein/seed storage 2S albumin superfamily protein	8.0E-25	4.6	3	–
isotig06361	AT4G17490	ATERF6	ethylene responsive element binding factor 6	7.0E-15	4.1	3	–
isotig01364	AT2G35710	–	Nucleotide-diphospho-sugar transferases superfamily protein	0.0	4.0	3	–
HI9HAF203C8DWS	AT4G38650	–	Glycosyl hydrolase family 10 protein	6.0E-42	3.8	3	–
HI9HAF203C40LU	AT5G38260	–	Protein kinase superfamily protein	4.0E-42	3.8	3	–
HI9HAF202CGABV	AT4G32300	SD2-5	S-domain-2 5	2.0E-43	3.7	3	–
isotig05467	AT5G23240	–	DNAJ heat shock N-terminal domain-containing protein	1.0E-16	3.7	3	–
isotig04037	AT1G55370	NDF5	NDH-dependent cyclic electron flow 5	2.0E-34	3.6	3	–
isotig03041	AT5G49120	–	Protein of unknown function (DUF581)	4.0E-14	3.6	3	–
SSH24-3-61_010_E08	AT3G04290	ATLTL1	Li-tolerant lipase 1	0.0	3.6	3	–
isotig03686	AT1G75020	LPAT4	lysophosphatidyl acyltransferase 4	0.0	3.6	3	–
isotig04335	AT2G48020	–	Major facilitator superfamily protein	0.0	3.6	3	–
SSH24-8-92_008_D12	AT1G14520	MIOX1	myo-inositol oxygenase 1	0.0	3.6	3	–
HI9HAF202CFXZK	AT3G12500	ATHCHIB	basic chitinase	0.0	3.5	3	–
HI9HAF202CAYYO	AT5G57360	ZTL	Galactose oxidase/kelch repeat superfamily protein	0.0	3.5	3	–
HI9HAF203DBE55	AT2G41480	–	Peroxidase superfamily protein	9.0E-25	3.5	3	–
Shoot-010-48	AT5G47390	–	myb-like transcription factor family protein	0.0	3.4	3	–
HI9HAF202CE0PV	AT5G14040	PHT3;1	phosphate transporter 3;1	0.0	3.4	3	–
HI9HAF202BZIYP	AT3G18110	EMB1270	Pentatricopeptide repeat (PPR) superfamily protein	0.0	3.3	3	–
isotig06497	AT3G14067	–	Subtilase family protein	2.0E-40	3.3	3	–
isotig05458	AT1G70000	–	myb-like transcription factor family protein	8.0E-29	3.3	3	–
HI9HAF203C68XH	AT1G22540	–	Major facilitator superfamily protein	5.6E-45	3.2	3	–
HI9HAF203DC47B	AT1G71695	–	Peroxidase superfamily protein	0.0	3.2	3	–
isotig06315	AT4G19160	–	–	0.0	3.2	3	–
HI9HAF203DQAN0	AT1G68750	ATPPC4	phosphoenolpyruvate carboxylase 4	0.0	3.2	3	–
isotig02760	AT5G20190	–	Tetratricopeptide repeat (TPR)-like superfamily protein	7.0E-35	3.2	3	–
HI9HAF202CHAVD	AT3G48530	KING1	SNF1-related protein kinase regulatory subunit gamma 1	1.0E-41	3.1	3	–
HI9HAF202BURL0	AT4G18250	–	receptor serine/threonine kinase, putative	2.0E-41	3.1	3	–
isotig05093	AT5G48480	–	Lactoylglutathione lyase / glyoxalase I family protein	8.0E-21	3.0	3	–
isotig05965	AT5G25280	–	serine-rich protein-related	1.0E-15	3.0	3	–
HI9HAF203C83LV	AT2G20790	–	clathrin adaptor complexes medium subunit family protein	0.0	3.0	3	–
HI9HAF203DRR2U	AT1G60590	–	Pectin lyase-like superfamily protein	1.4E-45	3.0	3	–
isotig05097	AT3G25600	–	Calcium-binding EF-hand family protein	7.0E-22	2.9	3	–
HI9HAF202BS5O6	AT2G18150	–	Peroxidase superfamily protein	2.0E-16	2.9	3	–
isotig02233	AT3G15850	FAD5	fatty acid desaturase 5	0.0	2.9	3	–
HI9HAF203DJS7S	AT4G15560	CLA1	Deoxyxylulose-5-phosphate synthase	0.0	2.9	3	–
HI9HAF202B74YU	AT1G49380	–	cytochrome c biogenesis protein family	0.0	2.8	3	–
isotig05171	AT2G47770	ATTSPO	TSPO(outer membrane tryptophan-rich sensory protein)-related	4.0E-20	2.8	3	–
isotig06359	AT4G33625	–	–	0.0	2.8	3	–
isotig03774	AT2G15890	MEE14	maternal effect embryo arrest 14	8.0E-31	2.8	3	–
Shoot-047-37	AT2G28080	–	UDP-Glycosyltransferase superfamily protein	8.0E-20	2.8	3	–
isotig01977	AT2G42850	CYP718	cytochrome P450, family 718	0.0	2.8	3	–
Shoot-050-49	AT5G52420	–	–	4.0E-42	2.7	3	–
Shoot-048-54	AT2G44830	–	Protein kinase superfamily protein	0.0	2.7	3	–
HI9HAF202B4NVC	AT1G10760	SEX1	Pyruvate phosphate dikinase, PEP/pyruvate binding domain	0.0	2.7	3	–
Shoot-023-66	AT3G59480	–	pfkB-like carbohydrate kinase family protein	2.0E-36	2.7	3	–
HI9HAF202CHFG2	AT1G02460	–	Pectin lyase-like superfamily protein	9.8E-45	2.7	3	–
Shoot-006-47_014_G06	AT1G73340	–	Cytochrome P450 superfamily protein	1.4E-45	2.7	3	–
HI9HAF203DIIVI	AT3G15510	ATNAC2	NAC domain containing protein 2	0.0	2.7	3	–
isotig04532	AT5G53190	SWEET3	Nodulin MtN3 family protein	8.0E-39	2.6	3	–
HI9HAF202BW9MV	AT4G18750	DOT4	Pentatricopeptide repeat (PPR) superfamily protein	2.0E-42	2.6	3	–
HI9HAF203C0AMS	AT2G27610	–	Tetratricopeptide repeat (TPR)-like superfamily protein	0.0	2.6	3	–
HI9HAF202BZ3FJ	AT3G23330	–	Tetratricopeptide repeat (TPR)-like superfamily protein	0.0	2.6	3	–
isotig03141	AT1G77490	TAPX	thylakoidal ascorbate peroxidase	0.0	2.6	3	–
HI9HAF203DLH96	AT3G53360	–	Tetratricopeptide repeat (TPR)-like superfamily protein	5.0E-42	2.6	3	–
HI9HAF203DKPZT	AT4G35290	GLUR2	glutamate receptor 2	0.0	2.6	3	–
isotig05621	AT4G10260	–	pfkB-like carbohydrate kinase family protein	0.0	2.6	3	–
HI9HAF202B3L30	AT4G13180	–	NAD(P)-binding Rossmann-fold superfamily protein	0.0	2.5	3	–
HI9HAF202B6KMR	AT2G33860	ETT	Transcriptional factor B3 family protein / auxin-responsive factor AUX/IAA-related	1.0E-22	2.5	3	AUX
isotig03567	AT5G14370	–	CCT motif family protein	9.0E-22	2.5	3	–
HI9HAF202B330Q	AT2G40840	DPE2	disproportionating enzyme 2	1.4E-45	2.5	3	–
isotig04855	AT2G34860	EDA3	DnaJ/Hsp40 cysteine-rich domain superfamily protein	6.0E-43	2.5	3	–
isotig06109	AT3G15500	ATNAC3	NAC domain containing protein 3	0.0	2.5	3	–
HI9HAF202CJV4W	AT1G07030	–	Mitochondrial substrate carrier family protein	2.0E-44	2.5	3	–
HI9HAF203CY3P1	AT1G28690	–	Tetratricopeptide repeat (TPR)-like superfamily protein	0.0	2.5	3	–
HI9HAF202CA2WB	AT4G03420	–	Protein of unknown function (DUF789)	9.8E-45	2.5	3	–
isotig05756	AT4G25640	DTX35	detoxifying efflux carrier 35	0.0	2.5	3	–
HI9HAF203DK7OG	AT1G64810	APO1	Arabidopsis thaliana protein of unknown function (DUF794)	0.0	2.5	3	–
isotig06116	AT4G33170	–	Tetratricopeptide repeat (TPR)-like superfamily protein	0.0	2.4	3	–
isotig03971	AT1G01470	LEA14	Late embryogenesis abundant protein	9.0E-40	2.4	3	–
HI9HAF202CAVDN	AT3G26744	ICE1	basic helix-loop-helix (bHLH) DNA-binding superfamily protein	0.0	2.4	3	–
Shoot-057-82	AT3G52072	–	other RNA	3.0E-11	2.4	3	–
HI9HAF203DOLVW	AT5G15700	–	DNA/RNA polymerases superfamily protein	0.0	2.4	3	–
HI9HAF202BUOWC	AT3G27020	YSL6	YELLOW STRIPE like 6	0.0	2.4	3	–
Shoot-030-02	AT1G53090	SPA4	SPA1-related 4	0.0	2.4	3	–
HI9HAF202CJZVZ	AT1G04690	KAB1	potassium channel beta subunit 1	0.0	2.4	3	–
HI9HAF202CIW9K	AT2G13600	–	Pentatricopeptide repeat (PPR) superfamily protein	1.0E-43	2.4	3	–
HI9HAF203C74U1	AT3G28345	–	ABC transporter family protein	0.0	2.4	3	–
HI9HAF203DJ75E	AT2G32390	GLR3.5	glutamate receptor 3.5	0.0	2.4	3	–
isotig05890	AT5G52190	–	Sugar isomerase (SIS) family protein	2.0E-37	2.4	3	–
HI9HAF203C3B9A	AT5G55950	–	Nucleotide/sugar transporter family protein	0.0	2.3	3	–

isotig03402	AT5G47470	－	Nodulin MtN21 /EamA-like transporter family protein	0.0	2.3	3	－
isotig06219	AT1G11530	ATCXXS1	C-terminal cysteine residue is changed to a serine 1	4.0E-32	2.3	3	－
isotig03949	AT3G26590	－	MATE efflux family protein	0.0	2.3	3	－
Shoot-048-32	AT5G18170	GDH1	glutamate dehydrogenase 1	0.0	2.3	3	－
HI9HAF203C5OKX	AT4G14510	ATCFM3B	CRM family member 3B	6.0E-43	2.3	3	－
isotig01148	AT3G54450	－	Major facilitator superfamily protein	2.0E-29	2.3	3	－
isotig01951	AT1G30500	NF-YA7	nuclear factor Y, subunit A7	4.0E-36	2.3	3	－
Shoot-007-68_007_D09	AT4G13650	－	Pentatricopeptide repeat (PPR) superfamily protein	0.0	2.3	3	－
isotig05388	AT1G18990	－	Protein of unknown function, DUF593	2.0E-17	2.3	3	－
HI9HAF203DGPKH	AT2G37040	PAL1	PHE ammonia lyase 1	2.0E-34	2.2	3	－
Shoot-017-91_006_C12	AT2G21090	－	Pentatricopeptide repeat (PPR-like) superfamily protein	3.0E-40	2.2	3	－
Shoot-024-67	AT3G54420	ATEP3	homolog of carrot EP3-3 chitinase	0.0	2.2	3	－
isotig01408	AT2G39210	－	Major facilitator superfamily protein	0.0	2.2	3	－
HI9HAF202BZFRA	AT1G12770	ISE1	P-loop containing nucleoside triphosphate hydrolases superfamily protein	0.0	2.2	3	－
isotig02286	AT4G13250	NYC1	NAD(P)-binding Rossmann-fold superfamily protein	0.0	2.2	3	－
HI9HAF202CLUAO	AT2G35030	－	Pentatricopeptide repeat (PPR) superfamily protein	1.0E-43	2.2	3	－
HI9HAF202B5UN4	AT4G01037	WTF1	Ubiquitin carboxyl-terminal hydrolase family protein	0.0	2.2	3	－
isotig00739	AT5G06570	－	alpha/beta-Hydrolases superfamily protein	0.0	2.2	3	－
isotig00643	AT3G13620	－	Amino acid permease family protein	0.0	2.2	3	－
HI9HAF202BUDWE	AT5G39840	－	ATP-dependent RNA helicase, mitochondrial, putative	0.0	2.2	3	－
HI9HAF202B0PLZ	AT4G33990	EMB2758	Tetratricopeptide repeat (TPR)-like superfamily protein	0.0	2.2	3	－
Shoot-048-87	AT2G21940	SK1	shikimate kinase 1	4.0E-17	2.2	3	－
isotig06564	AT1G18940	－	Nodulin-like / Major Facilitator Superfamily protein	3.0E-32	2.2	3	－
HI9HAF202CA6JB	AT4G02750	－	Tetratricopeptide repeat (TPR)-like superfamily protein	0.0	2.2	3	－
HI9HAF203DKIX6	AT2G23060	－	Acyl-CoA N-acyltransferases (NAT) superfamily protein	1.0E-43	2.2	3	－
HI9HAF202B5KSD	AT3G25900	HMT-1	Homocysteine S-methyltransferase family protein	0.0	2.2	3	－
HI9HAF202B3SBC	AT1G75660	XRN3	5'-3' exoribonuclease 3	0.0	2.2	3	－
HI9HAF202CE1RL	AT3G46970	ATPHS2	alpha-glucan phosphorylase 2	0.0	2.2	3	－
HI9HAF202CBR5J	AT3G26782	－	Tetratricopeptide repeat (TPR)-like superfamily protein	2.0E-41	2.1	3	－
isotig00770	AT2G16600	ROC3	rotamase CYP 3	0.0	2.1	3	－
HI9HAF203DSDCM	AT1G15500	ATNTT2	TLC ATP/ADP transporter	0.0	2.1	3	－
HI9HAF202B1CBE	AT1G67110	CYP735A2	cytochrome P450, family 735, subfamily A, polypeptide 2	9.0E-23	2.1	3	CK
HI9HAF203DG3DY	AT3G05510	－	Phospholipid/glycerol acyltransferase family protein	0.0	2.1	3	－
isotig01075	AT4G37680	HHP4	heptahelical protein 4	5.0E-24	2.1	3	－
HI9HAF202CFWUK	AT5G55830	－	Concanavalin A-like lectin protein kinase family protein	0.0	2.1	3	－
isotig01762	AT3G11410	ATPP2CA	protein phosphatase 2CA	0.0	2.1	3	ABA
HI9HAF203CZ4FC	AT1G67960	－	CONTAINS InterPro DOMAIN/s: Membrane protein,Tapt1/CMV receptor (InterPro:IPR008010)	2.0E-44	2.1	3	－
HI9HAF202B29ZJ	AT5G14580	－	polyribonucleotide nucleotidyltransferase, putative	0.0	2.1	3	－
HI9HAF202BVZ8Z	AT3G24503	ALDH2C4	aldehyde dehydrogenase 2C4	8.0E-43	2.1	3	－
HI9HAF202B517Z	AT3G57430	OTP84	Tetratricopeptide repeat (TPR)-like superfamily protein	0.0	2.1	3	－
isotig02041	AT5G04630	CYP77A9	cytochrome P450, family 77, subfamily A, polypeptide 9	0.0	2.1	3	－
HI9HAF203DPQZS	AT4G30825	－	Tetratricopeptide repeat (TPR)-like superfamily protein	1.0E-41	2.1	3	－
isotig01521	AT4G00370	ANTR2	Major facilitator superfamily protein	0.0	2.1	3	－
HI9HAF202CB40S	AT5G25560	－	CHY-type/CTCHY-type/RING-type Zinc finger protein	0.0	2.1	3	－
HI9HAF202B2Z9S	AT5G47910	RBOHD	respiratory burst oxidase homologue D	0.0	2.1	3	－
isotig04971	AT3G54260	TBL36	TRICHOME BIREFRINGENCE-LIKE 36	0.0	2.1	3	－
HI9HAF202B7NUR	AT5G23690	－	Polynucleotide adenylyltransferase family protein	0.0	2.0	3	－
isotig04842	AT3G61790	－	Protein with RING/U-box and TRAF-like domains	0.0	2.0	3	－
isotig05620	AT1G04920	ATSPS3F	sucrose phosphate synthase 3F	0.0	2.0	3	－
Shoot-021-36_007_D05	AT5G62420	－	NAD(P)-linked oxidoreductase superfamily protein	6.0E-24	2.0	3	－
HI9HAF202CFXCU	AT1G80300	NTT1	nucleotide transporter 1	0.0	2.0	3	－
isotig01340	AT5G67360	ARA12	Subtilase family protein	0.0	2.0	3	－
isotig02913	AT5G58490	－	NAD(P)-binding Rossmann-fold superfamily protein	0.0	2.0	3	－
isotig00991	AT2G33750	ATPUP2	purine permease 2	7.0E-42	2.0	3	－
SSH24-5-89_002_A12	AT5G42570	－	B-cell receptor-associated 31-like	3.0E-35	2.0	3	－
Shoot-057-45	AT5G17300	RVE1	Homeodomain-like superfamily protein	5.0E-38	14.3	4	－
HI9HAF202B43DT	AT5G13490	AAC2	ADP/ATP carrier 2	0.0	9.1	4	－
isotig03899	AT5G51550	EXL3	EXORDIUM like 3	0.0	8.4	4	－
isotig02211	AT1G23760	JP630	BURP domain-containing protein	2.0E-25	6.9	4	－
HI9HAF202B8ZAL	AT5G08370	AtAGAL2	alpha-galactosidase 2	0.0	6.6	4	－
HI9HAF202B3ZN6	AT4G00110	GAE3	UDP-D-glucuronate 4-epimerase 3	0.0	6.0	4	－
Shoot-059-71	AT3G45960	ATEXLA3	expansin-like A3	0.0	5.2	4	－
HI9HAF203DMPHF	AT1G69830	ATAMY3	alpha-amylase-like 3	0.0	4.4	4	－
isotig01056	AT4G03210	XTH9	xyloglucan endotransglucosylase/hydrolase 9	0.0	4.2	4	－
isotig05759	AT2G27830	－	－	6.0E-17	4.1	4	－
isotig03407	AT1G35140	PHI-1	Phosphate-responsive 1 family protein	0.0	4.1	4	－
HI9HAF203DB8LO	AT2G06050	OPR3	oxophytodienoate-reductase 3	3.0E-24	4.0	4	JA
isotig06283	AT4G25810	XTR6	xyloglucan endotransglycosylase 6	0.0	3.9	4	－
isotig05161	AT3G45970	ATEXLA1	expansin-like A1	0.0	3.9	4	－
HI9HAF202CAVXN	AT5G19730	－	Pectin lyase-like superfamily protein	0.0	3.7	4	－
HI9HAF203C7NW7	AT1G79460	GA2	Terpenoid cyclases/Protein prenyltransferases superfamily protein	5.0E-19	3.6	4	GA
Shoot-057-11	AT5G46600	－	Aluminium activated malate transporter family protein	8.0E-27	3.4	4	－
isotig06395	AT2G39540	－	Gibberellin-regulated family protein	7.0E-23	3.4	4	－
isotig06749	AT5G49920	－	Octicosapeptide/Phox/Bem1p family protein	1.0E-31	3.3	4	－
HI9HAF202BXWKV	AT3G62660	GATL7	galacturonosyltransferase-like 7	0.0	3.2	4	－
HI9HAF202B8HFI	AT3G10210	－	SEC14 cytosolic factor family protein / phosphoglyceride transfer family protein	0.0	3.1	4	－
isotig04982	AT4G31970	CYP82C2	cytochrome P450, family 82, subfamily C, polypeptide 2	8.0E-43	3.1	4	－
HI9HAF203C1O6D	AT2G44500	－	O-fucosyltransferase family protein	0.0	3.0	4	－
HI9HAF203DQ59H	AT1G10550	XTH33	xyloglucan:xyloglucosyl transferase 33	1.4E-45	3.0	4	－
isotig04228	AT5G01740	－	Nuclear transport factor 2 (NTF2) family protein	1.0E-28	3.0	4	－
SSH24-8-75_006_C10	AT3G59730	－	Concanavalin A-like lectin protein kinase family protein	3.0E-14	2.9	4	－
isotig03530	AT1G69530	ATEXPA1	expansin A1	0.0	2.9	4	－
HI9HAF203DJGMW	AT4G04320	－	malonyl-CoA decarboxylase family protein	0.0	2.8	4	－
HI9HAF202B60K5	AT1G64660	ATMGL	methionine gamma-lyase	0.0	2.8	4	－
Shoot-027-76	AT3G50950	ZAR1	HOPZ-ACTIVATED RESISTANCE 1	7.0E-14	2.8	4	－
Shoot-009-64_016_H08	AT4G09720	ATRABG3A	RAB GTPase homolog G3A	0.0	2.8	4	－
HI9HAF203DO77V	AT3G47800	－	Galactose mutarotase-like superfamily protein	0.0	2.8	4	－
Shoot-045-28	AT1G78990	－	HXXXD-type acyl-transferase family protein	0.0	2.8	4	－
isotig04997	AT4G27450	－	Aluminium induced protein with YGL and LRDR motifs	0.0	2.7	4	－
Shoot-020-39_013_G05	AT5G57530	XTH12	xyloglucan endotransglucosylase/hydrolase 12	0.0	2.6	4	－
HI9HAF203DIROI	AT4G31940	CYP82C4	cytochrome P450, family 82, subfamily C, polypeptide 4	0.0	2.6	4	－
HI9HAF202B6MT8	AT4G17690	－	Peroxidase superfamily protein	0.0	2.6	4	－
isotig04684	AT5G19140	ATAILP1	Aluminium induced protein with YGL and LRDR motifs	0.0	2.6	4	－
isotig03482	AT5G08640	FLS1	flavonol synthase 1	0.0	2.6	4	－
Shoot-003-41_002_A06	AT2G42990	－	GDSL-like Lipase/Acylhydrolase superfamily protein	3.0E-36	2.6	4	－
Shoot-001-50_003_B07	AT1G15780	－	－	3.0E-19	2.6	4	－
isotig00990	AT1G28220	ATPUP3	purine permease 3	0.0	2.6	4	－
Shoot-002-67_005_C09	AT1G22340	AtUGT85A7	UDP-glucosyl transferase 85A7	0.0	2.5	4	－
isotig03846	AT5G66330	－	Leucine-rich repeat (LRR) family protein	0.0	2.5	4	－
isotig03851	AT1G72360	HRE1	Integrase-type DNA-binding superfamily protein	2.0E-21	2.5	4	－

isotig02733	AT3G17940	–	Galactose mutarotase-like superfamily protein	0.0	2.5	4	–
isotig02283	AT4G39660	AGT2	alanine:glyoxylate aminotransferase 2	0.0	2.5	4	–
Shoot-055-73	AT5G15710	–	Galactose oxidase/kelch repeat superfamily protein	1.0E-12	2.5	4	–
HI9HAF202B25D0	AT3G20440	EMB2729	Alpha amylase family protein	0.0	2.5	4	–
isotig04911	AT3G19920	–	–	4.0E-20	2.4	4	–
Shoot-006-72_015_H09	AT5G47750	D6PKL2	D6 protein kinase like 2	0.0	2.4	4	–
Shoot-020-36_007_D05	AT1G19510	ATRL5	RAD-like 5	9.0E-21	2.4	4	–
HI9HAF202CFH1P	AT1G52340	ABA2	NAD(P)-binding Rossmann-fold superfamily protein	4.0E-31	2.4	4	ABA
HI9HAF203DOBMP	AT1G06410	ATTPS7	trehalose-phosphatase/synthase 7	0.0	2.4	4	–
Shoot-050-15	AT1G74070	–	Cyclophilin-like peptidyl-prolyl cis-trans isomerase family protein	3.0E-36	2.4	4	–
HI9HAF202B13P3	AT5G15180	–	Peroxidase superfamily protein	6.0E-32	2.3	4	–
HI9HAF202CGHZY	AT2G21050	LAX2	like AUXIN RESISTANT 2	5.0E-17	2.3	4	AUX
isotig02602	ATCG01130	YCF1.2	Ycf1 protein	2.0E-17	2.3	4	–
isotig06158	AT3G62630	–	Protein of unknown function (DUF1645)	4.0E-31	2.3	4	–
HI9HAF202B1H1R	AT3G21770	–	Peroxidase superfamily protein	2.0E-38	2.3	4	–
HI9HAF203DJQE7	AT5G52040	ATRSP41	RNA-binding (RRM/RBD/RNP motifs) family protein	9.0E-43	2.3	4	–
Shoot-029-01	AT4G24210	SLY1	F-box family protein	2.0E-21	2.3	4	GA
isotig06760	AT5G15780	–	Pollen Ole e 1 allergen and extensin family protein	1.0E-20	2.3	4	–
HI9HAF202B0EQA	AT4G39700	–	Heavy metal transport/detoxification superfamily protein	0.0	2.3	4	–
HI9HAF203DRR0L	AT5G51060	RHD2	NADPH/respiratory burst oxidase protein D	7.0E-41	2.3	4	–
HI9HAF202B3JCJ	AT1G60140	ATTPS10	trehalose phosphate synthase	0.0	2.2	4	–
HI9HAF203DEMEB	AT1G15210	PDR7	pleiotropic drug resistance 7	0.0	2.2	4	–
HI9HAF202CEN3B	AT4G38620	ATMYB4	myb domain protein 4	7.0E-42	2.2	4	–
isotig06805	AT5G45120	–	Eukaryotic aspartyl protease family protein	3.0E-36	2.2	4	–
isotig05149	AT3G50830	COR413-PM2	cold-regulated 413-plasma membrane 2	0.0	2.2	4	–
HI9HAF202B3961	AT1G11260	STP1	sugar transporter 1	0.0	2.2	4	–
HI9HAF202CIH7U	AT2G25520	–	Drug/metabolite transporter superfamily protein	0.0	2.2	4	–
Shoot-048-06	AT3G60490	–	Integrase-type DNA-binding superfamily protein	1.0E-32	2.2	4	–
HI9HAF203C2XJW	AT1G78060	–	Glycosyl hydrolase family protein	0.0	2.1	4	–
isotig03928	AT1G53830	ATPME2	pectin methylesterase 2	2.0E-30	2.1	4	–
SSH24-3-67_005_C09	AT1G01490	–	Heavy metal transport/detoxification superfamily protein	1.0E-21	2.1	4	–
Shoot-008-11_006_C02	AT1G22360	AtUGT85A2	UDP-glucosyl transferase 85A2	0.0	2.1	4	–
HI9HAF202CE9R3	AT4G32390	–	Nucleotide-sugar transporter family protein	0.0	2.1	4	–
isotig00944	AT5G18100	CSD3	copper/zinc superoxide dismutase 3	0.0	2.1	4	–
HI9HAF203CYDVV	AT4G37530	–	Peroxidase superfamily protein	0.0	2.1	4	–
isotig02436	ATCG00660	RPL20	ribosomal protein L20	6.0E-25	2.1	4	–
HI9HAF202CI4ZB	AT4G37800	XTH7	xyloglucan endotransglucosylase/hydrolase 7	3.0E-43	2.1	4	–
SSH24-6-72_015_H09	AT4G02420	–	Concanavalin A-like lectin protein kinase family protein	1.0E-39	2.1	4	–
Shoot-001-51_005_C07	AT5G04830	–	Nuclear transport factor 2 (NTF2) family protein	0.0	2.1	4	–
isotig02093	AT4G36360	BGAL3	beta-galactosidase 3	0.0	2.1	4	–
isotig05554	AT3G03990	–	alpha/beta-Hydrolases superfamily protein	0.0	2.1	4	–
HI9HAF203DK8BF	AT2G20780	–	Major facilitator superfamily protein	0.0	2.1	4	–
HI9HAF202B0NQ5	AT3G18830	ATPLT5	polyol/monosaccharide transporter 5	0.0	2.1	4	–
HI9HAF202CHGAJ	AT2G16120	PMT1	polyol/monosaccharide transporter 1	2.8E-45	2.1	4	–
isotig06679	AT3G55520	–	FKBP-like peptidyl-prolyl cis-trans isomerase family protein	2.0E-24	2.1	4	–
isotig04575	AT1G09560	GLP5	germin-like protein 5	0.0	2.0	4	–
isotig02695	AT1G55020	LOX1	lipoxygenase 1	0.0	2.0	4	–
isotig01036	AT5G48930	HCT	hydroxycinnamoyl-CoA shikimate/quinate hydroxycinnamoyl transferase	1.0E-15	2.0	4	–
isotig00252	AT1G01390	–	UDP-Glycosyltransferase superfamily protein	2.8E-45	2.0	4	–
Shoot-027-69	AT1G45474	LHCA5	photosystem I light harvesting complex gene 5	1.0E-15	2.0	4	–
isotig00729	AT5G43830	–	Aluminium induced protein with YGL and LRDR motifs	0.0	2.0	4	–
isotig01708	AT5G57030	LUT2	Lycopene beta/epsilon cyclase protein	0.0	2.0	4	–
HI9HAF202B3PO5	AT4G16370	ATOPT3	oligopeptide transporter	0.0	2.0	4	–
Shoot-046-89	AT1G22650	–	Plant neutral invertase family protein	0.0	2.0	4	–
SSH24-9-41_002_A06	AT3G53810	–	Concanavalin A-like lectin protein kinase family protein	1.0E-19	2.0	4	–
isotig00726	AT2G47490	ATNDT1	NAD+ transporter 1	0.0	2.0	4	–

[1] The putative function of the sequences was predicted according to the highest BLASTX hits with an e-value cutoff of e-10. Only sequences that hit a unique Arabidopsis gene ID are listed here.
[2] Fold change indicates the ratio of maximal and minimal expression in summer.
[3] Cluster number indicates a similarity in expression patterns defined by cluster analysis. See Figure 2A for the expression pattern of each cluster.
[4] Putative hormone-related genes representing hormone biosynthesis and signaling pathways are listed here (ABA, abscisic acid; AUX, auxin; BR, brassinosteroids; CK, cytokinins; ETH, ethylene; GA, gibberellin; JA, jasmonic acid).
