# Supplementary material for: Clock genes and diurnal transcriptome dynamics in summer and winter in the gymnosperm Japanese cedar (Cryptomeria japonica (L.f.) D.Don)
Source: BMC Plant Biol. 2014 Nov 18;14:308. doi: 10.1186/s12870-014-0308-1 (PMC4245765; doi:10.1186/s12870-014-0308-1)
Supplement: Additional file 6: — Targets with the highest coefficient of variations used for gene network analysis. 1The putative function of the sequences was predicted according to the highest BLASTX hits with an e-value cutoff of e-10. 2Putative clock gene with node colored red in the estimated gene network (Figure 4). 3Number of children, parents and all edges were estimated by the SiGN-BN Bayesian network estimation program (http://sign.hgc.jp/signbn/index.html) [76]. 4The time when the target reached maximum expression. [file 12870_2014_308_MOESM6_ESM.pdf]

Additional file 6. Targets with the highest coefficient of variations used for gene network analysis.

SEQ_ID	BLASTX[1]				clock-related[2]	gene network[3]			peak time[4]
	Arabi_ID	gene symbol	Discription	e-value		children	parents	all edges	
isotig00872	AT3G14200	–	Chaperone DnaJ-domain superfamily protein	4.0E-24	–	128	3	131	12:00
isotig05425	AT3G08970	ATERDJ3A	DNAJ heat shock N-terminal domain-containing protein	1.0E-43	–	123	2	125	12:00
Shoot-026-47	–	–	–	–	–	84	9	93	16:00
isotig05861	–	–	–	–	–	83	3	86	16:00
isotig02051	AT5G64510	TIN1	unknown protein	2.0E-94	–	82	4	86	12:00
Shoot-056-25	–	–	–	–	–	76	11	87	8:00
HI9HAF202CI8XY	AT2G32120	HSP70T-2	heat-shock protein 70T-2	2.0E-41	–	76	8	84	12:00
isotig05923	–	–	–	–	–	75	7	82	16:00
isotig03902	AT5G64170	–	dentin sialophosphoprotein-related	5.0E-11	–	74	5	79	8:00
HI9HAF202CMB2V	AT4G15560	CLA1	Deoxyxylulose-5-phosphate synthase	2.0E-43	–	73	1	74	16:00
HI9HAF203DJS7S	AT4G15560	CLA1	Deoxyxylulose-5-phosphate synthase	3.0E-51	–	70	1	71	16:00
isotig06594	–	–	–	–	–	69	7	76	12:00
isotig06867	–	–	–	–	–	68	4	72	16:00
HI9HAF202BXV4N	AT2G32120	HSP70T-2	heat-shock protein 70T-2	2.0E-42	–	67	7	74	12:00
isotig03774	AT2G15890	MEE14	maternal effect embryo arrest 14	8.0E-31	–	66	17	83	16:00
HI9HAF202BX9NX	AT1G64890	–	Major facilitator superfamily protein	7.0E-44	–	66	5	71	16:00
isotig06627	–	–	–	–	–	64	8	72	16:00
Shoot-049-21	AT1G76940	–	RNA-binding (RRM/RBD/RNP motifs) family protein	4.0E-27	–	64	7	71	12:00
Shoot-012-36_007_D05	–	–	–	–	–	64	5	69	12:00
isotig04546	AT5G51440	–	HSP20-like chaperones superfamily protein	1.0E-30	–	64	4	68	12:00
HI9HAF202BX0CC	AT1G64890	–	Major facilitator superfamily protein	5.0E-44	–	60	5	65	16:00
isotig05853	AT3G46230	ATHSP17.4	heat shock protein 17.4	3.0E-46	–	60	5	65	12:00
isotig01354	AT5G02500	HSC70-1	heat shock cognate protein 70-1	0.0	–	59	16	75	12:00
isotig01277	AT5G56030	HSP81-2	heat shock protein 81-2	0.0	–	59	7	66	12:00
isotig06497	AT3G14067	–	Subtilase family protein	2.0E-40	–	58	18	76	20:00
HI9HAF203CY3P1	AT1G28690	–	Tetratricopeptide repeat (TPR)-like superfamily protein	4.0E-46	–	57	17	74	20:00
isotig04121	AT5G24120	SIGE	sigma factor E	2.0E-54	–	57	15	72	8:00
isotig00873	AT3G14200	–	Chaperone DnaJ-domain superfamily protein	1.0E-23	–	57	13	70	12:00
isotig05544	AT2G45580	CYP76C3	cytochrome P450, family 76, subfamily C, polypeptide 3	3.0E-39	–	57	9	66	16:00
HI9HAF202B79W2	AT1G10760	SEX1	Pyruvate phosphate dikinase, PEP/pyruvate binding domain	3.0E-49	–	57	9	66	16:00
isotig00762	AT4G25000	ATAMY1	alpha-amylase-like	8.0E-103	–	56	15	71	16:00
HI9HAF203CZ6FR	–	–	–	–	–	56	13	69	16:00
isotig02040	AT5G65280	GCL1	GCR2-like 1	3.0E-129	–	56	11	67	16:00
isotig05235	AT5G12020	HSP17.6II	17.6 kDa class II heat shock protein	4.0E-38	–	56	8	64	12:00
HI9HAF203CWVTZ	AT1G01390	–	UDP-Glycosyltransferase superfamily protein	1.0E-41	–	55	11	66	8:00
HI9HAF203DOXJZ	AT5G39660	CDF2	cycling DOF factor 2	4.0E-41	–	54	14	68	8:00
HI9HAF202B4NVC	AT1G10760	SEX1	Pyruvate phosphate dikinase, PEP/pyruvate binding domain	1.0E-53	–	54	9	63	20:00
SSH24-2-01_001_A01	–	–	–	–	–	54	6	60	16:00
Shoot-021-79_014_G10	AT1G75000	–	GNS1/SUR4 membrane protein family	6.0E-48	–	53	20	73	8:00
HI9HAF202B9FSD	AT3G57630	–	exostosin family protein	6.0E-62	–	52	26	78	8:00
isotig00231	–	–	–	–	–	52	16	68	16:00
Shoot-008-90_004_B12	AT3G15810	–	Protein of unknown function (DUF567)	6.0E-18	–	52	12	64	12:00
isotig06531	AT1G76940	–	RNA-binding (RRM/RBD/RNP motifs) family protein	1.0E-25	–	52	10	62	16:00
isotig02665	AT3G09350	Fes1A	Fes1A	2.0E-123	–	52	8	60	12:00
Shoot-046-32	AT4G24540	AGL24	AGAMOUS-like 24	5.0E-32	–	51	22	73	4:00
HI9HAF202BU4R0	AT1G49380	–	cytochrome c biogenesis protein family	3.0E-48	–	51	16	67	16:00
isotig03466	–	–	–	–	–	51	13	64	12:00
isotig04760	AT2G37970	SOUL-1	SOUL heme-binding family protein	3.0E-65	–	51	12	63	12:00
HI9HAF203DLH96	AT3G53360	–	Tetratricopeptide repeat (TPR)-like superfamily protein	5.0E-42	–	50	15	65	16:00
HI9HAF203C17EN	AT3G57630	–	exostosin family protein	9.0E-63	–	50	15	65	8:00
isotig05171	AT2G47770	ATTSPO	TSPO(outer membrane tryptophan-rich sensory protein)-related	4.0E-20	–	50	12	62	16:00
HI9HAF202BVD0N	AT1G49380	–	cytochrome c biogenesis protein family	2.0E-48	–	50	12	62	16:00
isotig06792	AT1G01420	UGT72B3	UDP-glucosyl transferase 72B3	5.0E-13	–	49	16	65	8:00
HI9HAF202B43TO	AT2G26650	AKT1	K+ transporter 1	1.0E-56	–	49	16	65	8:00
isotig00839	–	–	–	–	–	49	14	63	16:00
HI9HAF202CAYYO	AT5G57360	ZTL	Galactose oxidase/kelch repeat superfamily protein	8.0E-55	CjZTL	49	7	56	16:00
isotig00878	AT1G48130	ATPER1	1-cysteine peroxiredoxin 1	3.0E-63	–	48	19	67	16:00
isotig01316	AT3G12580	HSP70	heat shock protein 70	0.0	–	48	18	66	12:00
HI9HAF203C8NXR	AT3G57630	–	exostosin family protein	6.0E-62	–	48	17	65	8:00
HI9HAF203DJ39G	AT2G39450	MTP11	Cation efflux family protein	3.0E-44	–	48	8	56	20:00
isotig04915	AT2G47180	AtGolS1	galactinol synthase 1	7.0E-87	–	48	7	55	12:00
HI9HAF202BS4XF	AT5G03555	–	permease, cytosine/purines, uracil, thiamine, allantoin family protein	3.0E-44	–	48	3	51	8:00
isotig01805	AT5G28540	BIP1	heat shock protein 70 (Hsp 70) family protein	0.0	–	47	15	62	12:00
Shoot-047-85	AT5G07040	–	RING/U-box superfamily protein	1.0E-34	–	47	10	57	24:00
HI9HAF202BWP2Y	AT4G19170	NCED4	nine-cis-epoxycarotenoid dioxygenase 4	1.0E-11	–	47	7	54	24:00
Shoot-039-41	–	–	–	–	–	47	4	51	16:00
isotig04596	AT5G12020	HSP17.6II	17.6 kDa class II heat shock protein	4.0E-41	–	47	2	49	12:00
HI9HAF202CI9W9	AT2G40840	DPE2	disproportionating enzyme 2	1.0E-45	–	46	21	67	16:00
isotig05762	AT2G45560	CYP76C1	cytochrome P450, family 76, subfamily C, polypeptide 1	8.0E-47	–	46	16	62	16:00
isotig05247	AT3G21150	BBX32	B-box 32	5.0E-16	–	46	15	61	8:00
SSH12-5-35_005_C05	AT1G49380	–	cytochrome c biogenesis protein family	3.0E-30	–	46	13	59	16:00
Shoot-026-13	–	–	–	–	–	46	12	58	4:00
isotig01109	–	–	–	–	–	46	5	51	16:00
Shoot-048-25	AT5G13700	ATPAO1	polyamine oxidase 1	3.0E-31	–	45	19	64	20:00
HI9HAF202B82XC	AT5G38710	–	Methylenetetrahydrofolate reductase family protein	7.0E-42	–	45	14	59	8:00
HI9HAF202B3L30	AT4G13180	–	NAD(P)-binding Rossmann-fold superfamily protein	2.0E-54	–	45	14	59	16:00
isotig01958	AT4G38060	–	unknown protein	1.0E-10	–	45	10	55	8:00
HI9HAF202B7FEC	AT1G10760	SEX1	Pyruvate phosphate dikinase, PEP/pyruvate binding domain	2.0E-49	–	45	10	55	16:00
HI9HAF202BWYA2	AT2G32950	COP1	Transducin/WD40 repeat-like superfamily protein	2.0E-46	–	45	9	54	8:00
SSH24-7-44_008_D06	AT4G14622	CPuORF60	conserved peptide upstream open reading frame 60	4.0E-32	–	45	8	53	20:00
HI9HAF202BZ69E	–	–	–	–	–	45	7	52	20:00
HI9HAF203C83LV	AT2G20790	–	clathrin adaptor complexes medium subunit family protein	1.0E-49	–	45	7	52	16:00
HI9HAF203DGFEU	AT5G57360	ZTL	Galactose oxidase/kelch repeat superfamily protein	2.0E-57	CjZTL	45	6	51	16:00
HI9HAF203DQSIW	AT2G32950	COP1	Transducin/WD40 repeat-like superfamily protein	3.0E-75	COP1	45	4	49	8:00
isotig06868	–	–	–	–	–	44	19	63	20:00
isotig06131	–	–	–	–	–	44	17	61	8:00
isotig06156	–	–	–	–	–	44	16	60	16:00
isotig03449	AT5G54470	–	B-box type zinc finger family protein	2.0E-18	–	44	14	58	8:00
HI9HAF203C4BMC	AT4G02750	–	Tetratricopeptide repeat (TPR)-like superfamily protein	1.0E-47	–	44	14	58	16:00
HI9HAF203C1GCN	AT5G03555	–	permease, cytosine/purines, uracil, thiamine, allantoin family protein	4.0E-51	–	44	3	47	8:00
HI9HAF203DF5L7	AT5G14130	–	Peroxidase superfamily protein	3.0E-24	–	44	0	44	24:00
HI9HAF203DNGNA	AT3G55550	–	Concanavalin A-like lectin protein kinase family protein	2.0E-45	–	43	16	59	12:00
HI9HAF202CBCF6	AT5G42020	BIP	Heat shock protein 70 (Hsp 70) family protein	2.0E-41	–	43	13	56	12:00
HI9HAF202B6I6Z	AT1G10760	SEX1	Pyruvate phosphate dikinase, PEP/pyruvate binding domain	8.0E-57	–	43	9	52	20:00
Shoot-054-22	–	–	–	–	–	43	7	50	12:00
Shoot-001-68_007_D09	–	–	–	–	–	43	1	44	24:00
HI9HAF202B8FUT	–	–	–	–	–	42	29	71	16:00
isotig06624	AT4G14830	HSP1	unknown protein	7.0E-14	–	42	23	65	12:00
isotig03520	–	–	–	–	–	42	18	60	4:00
Shoot-003-41_002_A06	AT2G42990	–	GDSL-like Lipase/Acylhydrolase superfamily protein	3.0E-36	–	42	14	56	24:00
isotig02484	AT5G19875	–	unknown protein	3.0E-22	–	42	10	52	12:00

HI9HAF202CLCMA	AT5G57360	ZTL	Galactose oxidase/kelch repeat superfamily protein	7.0E-74	CjZTL	42	5	47	16:00
HI9HAF202B5INI	–	–	–	–	–	42	1	43	4:00
isotig03376	AT3G12500	ATHCHIB	basic chitinase	6.0E-101	–	41	36	77	16:00
Shoot-049-08	–	–	–	–	–	41	26	67	16:00
HI9HAF202CA696	AT2G19130	–	S-locus lectin protein kinase family protein	1.0E-47	–	41	16	57	4:00
HI9HAF202B6KMR	AT2G33860	ETT	Transcriptional factor B3 family protein / auxin-responsive factor AUX/IAA-related	1.0E-22	–	41	15	56	16:00
HI9HAF203C6ZXE	AT1G22400	UGT85A1	UDP-Glycosyltransferase superfamily protein	2.0E-40	–	41	15	56	24:00
HI9HAF202B9BC8	AT5G24740	–	Protein of unknown function (DUF1162)	4.0E-41	–	41	11	52	24:00
HI9HAF203DH1YZ	AT3G23990	HSP60	heat shock protein 60	6.0E-75	–	41	10	51	12:00
HI9HAF203DHF0N	AT2G32950	COP1	Transducin/WD40 repeat-like superfamily protein	4.0E-65	–	41	9	50	8:00
HI9HAF202CA5QQ	AT1G66340	ETR1	Signal transduction histidine kinase, hybrid-type, ethylene sensor	4.0E-27	–	41	2	43	8:00
HI9HAF203C1O6D	AT2G44500	–	O-fucosyltransferase family protein	8.0E-48	–	40	19	59	4:00
HI9HAF203C15CV	AT5G38710	–	Methylenetetrahydrofolate reductase family protein	1.0E-43	–	40	17	57	8:00
HI9HAF202B0AVA	AT3G23990	HSP60	heat shock protein 60	7.0E-69	–	40	16	56	12:00
isotig03952	AT2G20560	–	DNAJ heat shock family protein	1.0E-94	–	40	14	54	12:00
isotig04542	–	–	–	–	–	40	12	52	12:00
isotig06012	AT5G03555	–	permease, cytosine/purines, uracil, thiamine, allantoin family protein	2.0E-50	–	40	11	51	8:00
Shoot-049-85	–	–	–	–	–	40	9	49	4:00
HI9HAF203C3BRH	AT2G40940	ERS1	ethylene response sensor 1	2.0E-38	–	40	5	45	8:00
Shoot-055-88	–	–	–	–	–	40	4	44	8:00
HI9HAF202B5NC3	–	–	–	–	–	40	3	43	24:00
Shoot-048-82	–	–	–	–	–	40	0	40	12:00
isotig06033	AT4G35100	PIP3	plasma membrane intrinsic protein 3	4.0E-34	–	39	25	64	8:00
isotig04037	AT1G55370	NDF5	NDH-dependent cyclic electron flow 5	2.0E-34	–	39	23	62	16:00
Shoot-050-65	–	–	–	–	–	39	21	60	16:00
isotig05046	AT5G39790	–	5'-AMP-activated protein kinase-related	3.0E-19	–	39	20	59	12:00
HI9HAF202CAXIE	AT5G09590	MTHSC70-2	mitochondrial HSO70 2	7.0E-63	–	39	18	57	12:00
HI9HAF203DEENJ	AT5G02500	HSC70-1	heat shock cognate protein 70-1	2.0E-73	–	39	16	55	12:00
isotig05105	–	–	–	–	–	39	15	54	16:00
isotig03433	AT3G54500	–	–	2.0E-30	–	39	15	54	12:00
Shoot-020-92_008_D12	AT1G26310	CAL	K-box region and MADS-box transcription factor family protein	4.0E-31	–	39	12	51	4:00
HI9HAF202B0QPY	AT5G42020	BIP	Heat shock protein 70 (Hsp 70) family protein	2.0E-41	–	39	12	51	12:00
isotig02916	AT2G45380	–	–	3.0E-27	–	39	8	47	12:00
isotig06271	–	–	–	–	–	39	7	46	20:00
SSH12-4-38_011_F05	AT5G34863	–	transposable element gene	1.0E-26	–	39	4	43	12:00
isotig06607	AT3G22840	ELIP1	Chlorophyll A-B binding family protein	2.0E-19	–	38	24	62	12:00
HI9HAF202CC49V	AT5G06530	–	ABC-2 type transporter family protein	1.0E-45	–	38	17	55	4:00
HI9HAF203DPW8Y	AT1G22400	UGT85A1	UDP-Glycosyltransferase superfamily protein	5.0E-42	–	38	14	52	20:00
HI9HAF202B399C	AT1G10760	SEX1	Pyruvate phosphate dikinase, PEP/pyruvate binding domain	4.0E-45	–	38	12	50	20:00
SSH12-1-80_016_H10	AT5G12420	–	O-acyltransferase (WSD1-like) family protein	4.0E-15	–	38	11	49	24:00
isotig01218	AT5G02810	PRR7	pseudo-response regulator 7	7.0E-82	PRR7	38	11	49	12:00
HI9HAF202BRDCF	AT5G57360	ZTL	Galactose oxidase/kelch repeat superfamily protein	5.0E-41	CjZTL	38	10	48	16:00
HI9HAF202BQTSA	AT4G38650	–	Glycosyl hydrolase family 10 protein	3.0E-41	–	38	10	48	16:00
HI9HAF203DBT8H	AT1G77120	ADH1	alcohol dehydrogenase 1	5.0E-50	–	37	37	74	8:00
HI9HAF203C6S6L	AT3G27970	–	Exonuclease family protein	8.0E-46	–	37	25	62	20:00
isotig04746	AT1G69830	ATAMY3	alpha-amylase-like 3	2.0E-39	–	37	23	60	20:00
HI9HAF203DJEF7	AT5G06530	–	ABC-2 type transporter family protein	7.0E-57	–	37	23	60	4:00
HI9HAF203C89CZ	AT1G76990	ACR3	ACT domain repeat 3	2.0E-57	–	37	22	59	8:00
SSH24-3-25_002_A04	–	–	–	–	–	37	17	54	4:00
Shoot-024-80	AT5G64170	–	dentin sialophosphoprotein-related	8.0E-19	–	37	17	54	12:00
isotig04180	AT3G43120	–	SAUR-like auxin-responsive protein family	2.0E-22	–	37	17	54	12:00
isotig04041	AT4G27670	HSP21	heat shock protein 21	3.0E-52	–	37	17	54	12:00
isotig06632	AT3G24500	MBF1C	multiprotein bridging factor 1C	2.0E-48	–	37	16	53	12:00
isotig04637	AT1G78370	ATGSTU20	glutathione S-transferase TAU 20	5.0E-56	–	37	12	49	8:00
HI9HAF202CLX46	AT1G22770	GI	gigantea protein (GI)	1.0E-50	CjGI	37	11	48	16:00
isotig00118	AT3G22840	ELIP1	Chlorophyll A-B binding family protein	4.0E-39	–	37	10	47	12:00
HI9HAF203C8DWS	AT4G38650	–	Glycosyl hydrolase family 10 protein	6.0E-42	–	37	10	47	16:00
isotig03686	AT1G75020	LPAT4	lysophosphatidyl acyltransferase 4	2.0E-81	–	37	10	47	16:00
isotig06738	AT2G19130	–	S-locus lectin protein kinase family protein	1.0E-28	–	37	8	45	4:00
HI9HAF202CIW9K	AT2G13600	–	Pentatricopeptide repeat (PPR) superfamily protein	1.0E-43	–	37	8	45	16:00
HI9HAF203DFIY2	AT5G23110	–	Zinc finger, C3HC4 type (RING finger) family protein	5.0E-42	–	37	3	40	24:00
isotig04474	AT4G14690	ELIP2	Chlorophyll A-B binding family protein	4.0E-42	–	36	26	62	12:00
HI9HAF202CHAVD	AT3G48530	KING1	SNF1-related protein kinase regulatory subunit gamma 1	1.0E-41	–	36	23	59	16:00
HI9HAF203C68XH	AT1G22540	–	Major facilitator superfamily protein	5.0E-45	–	36	20	56	16:00
Shoot-002-68_007_D09	AT2G15220	–	Plant basic secretory protein (BSP) family protein	1.0E-27	–	36	17	53	4:00
HI9HAF203CY63L	AT5G46240	KAT1	potassium channel in Arabidopsis thaliana 1	1.0E-48	–	36	16	52	8:00
HI9HAF203DDGDD	AT3G55550	–	Concanavalin A-like lectin protein kinase family protein	6.0E-56	–	36	16	52	12:00
HI9HAF203DFH8Q	AT4G13180	–	NAD(P)-binding Rossmann-fold superfamily protein	6.0E-52	–	36	13	49	16:00
isotig03407	AT1G35140	PHI-1	Phosphate-responsive 1 family protein	9.0E-49	–	36	12	48	4:00
HI9HAF202CFO68	AT5G33370	–	GDSL-like Lipase/Acylhydrolase superfamily protein	2.0E-50	–	36	10	46	24:00
Shoot-049-75	AT3G29090	PME31	pectin methylesterase 31	1.0E-115	–	36	7	43	20:00
HI9HAF202CKRK8	AT5G03555	–	permease, cytosine/purines, uracil, thiamine, allantoin family protein	4.0E-54	–	36	6	42	8:00
HI9HAF202B7JMI	–	–	–	–	–	36	4	40	24:00
HI9HAF203DMJ8J	AT5G23110	–	Zinc finger, C3HC4 type (RING finger) family protein	1.0E-45	–	36	4	40	24:00
HI9HAF202CMA0N	AT5G62230	ERL1	ERECTA-like 1	3.0E-56	–	36	3	39	24:00
HI9HAF203C5QRR	AT5G62230	ERL1	ERECTA-like 1	1.0E-47	–	36	2	38	24:00
HI9HAF202CA6JB	AT4G02750	–	Tetratricopeptide repeat (TPR)-like superfamily protein	1.0E-52	–	35	30	65	20:00
isotig05467	AT5G23240	–	DNAJ heat shock N-terminal domain-containing protein	1.0E-16	–	35	28	63	16:00
Shoot-006-47_014_G06	AT1G73340	–	Cytochrome P450 superfamily protein	2.0E-45	–	35	25	60	16:00
HI9HAF202BVBDX	AT2G41190	–	Transmembrane amino acid transporter family protein	6.0E-62	–	35	22	57	12:00
isotig03846	AT5G66330	–	Leucine-rich repeat (LRR) family protein	5.0E-60	–	35	20	55	4:00
isotig00993	–	–	–	–	–	35	19	54	8:00
SSH24-3-15_014_G02	–	–	–	–	–	35	18	53	4:00
Shoot-050-47	AT3G58680	MBF1B	multiprotein bridging factor 1B	1.0E-42	–	35	16	51	12:00
HI9HAF202CA57Y	AT1G22770	GI	gigantea protein (GI)	6.0E-52	CjGI	35	15	50	16:00
HI9HAF202B29T1	AT2G36570	–	Leucine-rich repeat protein kinase family protein	2.0E-53	–	35	14	49	24:00
HI9HAF203DOJ1Z	AT1G10760	SEX1	Pyruvate phosphate dikinase, PEP/pyruvate binding domain	9.0E-47	–	35	12	47	20:00
Shoot-001-17_001_A03	AT1G20135	–	GDSL-like Lipase/Acylhydrolase family protein	8.0E-20	–	35	11	46	8:00
HI9HAF203DIAPG	AT1G67710	ARR11	response regulator 11	9.0E-29	–	35	6	41	4:00
HI9HAF203C2P0N	AT5G18460	–	Protein of Unknown Function (DUF239)	3.0E-76	–	35	6	41	24:00
isotig05763	–	–	–	–	–	35	3	38	20:00
HI9HAF203DM1OJ	AT3G16520	UGT88A1	UDP-glucosyl transferase 88A1	5.0E-43	–	34	24	58	8:00
isotig01416	AT4G05200	CRK25	cysteine-rich RLK (RECEPTOR-like protein kinase) 25	4.0E-87	–	34	23	57	8:00
HI9HAF202CFXZK	AT3G12500	ATHCHIB	basic chitinase	2.0E-53	–	34	21	55	20:00
HI9HAF202CKXRQ	–	–	–	–	–	34	20	54	16:00
isotig05611	AT5G20720	CPN20	chaperonin 20	2.0E-66	–	34	19	53	12:00
Shoot-017-21_009_E03	–	–	–	–	–	34	18	52	16:00
isotig04885	AT2G42990	–	GDSL-like Lipase/Acylhydrolase superfamily protein	1.0E-50	–	34	16	50	24:00
isotig03787	–	–	–	–	–	34	15	49	4:00
isotig05665	AT1G22770	GI	gigantea protein (GI)	3.0E-65	CjGI	34	15	49	16:00
HI9HAF202B6HRW	AT1G22400	UGT85A1	UDP-Glycosyltransferase superfamily protein	2.0E-35	–	34	13	47	20:00
Shoot-047-37	AT2G28080	–	UDP-Glycosyltransferase superfamily protein	8.0E-20	–	34	11	45	16:00
isotig03899	AT5G51550	EXL3	EXORDIUM like 3	7.0E-82	–	34	9	43	4:00
isotig06245	–	–	–	–	–	34	5	39	4:00
isotig01574	AT1G27680	APL2	ADPGLC-PPase large subunit	0.0	–	33	29	62	16:00

HI9HAF202B3ZN6	AT4G00110	GAE3	UDP-D-glucuronate 4-epimerase 3	7.0E-69	–	33	26	59	4:00
isotig06221	AT5G45340	CYP707A3	cytochrome P450, family 707, subfamily A, polypeptide 3	2.0E-80	–	33	23	56	12:00
HI9HAF202B4Y9S	AT1G22770	GI	gigantea protein (GI)	6.0E-25	CjGI	33	18	51	16:00
Shoot-057-45	AT5G17300	RVE1	Homeodomain-like superfamily protein	5.0E-38	CjLHYb	33	17	50	4:00
isotig04710	AT3G07090	–	PPPDE putative thiol peptidase family protein	5.0E-68	–	33	14	47	12:00
isotig02156	–	–	–	–	–	33	11	44	12:00
Shoot-045-43	–	–	–	–	–	33	11	44	24:00
isotig04536	AT5G57360	ZTL	Galactose oxidase/kelch repeat superfamily protein	2.0E-89	CjZTL	33	11	44	16:00
HI9HAF203DR2XC	AT5G57360	ZTL	Galactose oxidase/kelch repeat superfamily protein	7.0E-67	CjZTL	33	10	43	16:00
HI9HAF203C7IXG	AT1G22400	UGT85A1	UDP-Glycosyltransferase superfamily protein	1.0E-17	–	33	9	42	24:00
Shoot-021-18_003_B03	–	–	–	–	–	33	1	34	4:00
HI9HAF202CHJUN	–	–	–	–	–	33	1	34	24:00
isotig06238	–	–	–	–	–	32	27	59	4:00
isotig01008	AT5G56300	GAMT2	gibberellic acid methyltransferase 2	2.0E-52	–	32	26	58	8:00
Shoot-023-83	–	–	–	–	–	32	25	57	20:00
isotig01294	AT5G42020	BIP	Heat shock protein 70 (Hsp 70) family protein	0.0	–	32	23	55	12:00
HI9HAF203C266F	AT3G19270	CYP707A4	cytochrome P450, family 707, subfamily A, polypeptide 4	9.0E-41	–	32	21	53	12:00
HI9HAF203DFI5U	AT1G07850	–	Protein of unknown function (DUF604)	2.0E-41	–	32	21	53	4:00
HI9HAF202B4Q1X	AT5G05340	–	Peroxidase superfamily protein	9.0E-38	–	32	20	52	4:00
Shoot-001-69_009_E09	–	–	–	–	–	32	19	51	16:00
HI9HAF203DKPZT	AT4G35290	GLUR2	glutamate receptor 2	2.0E-47	–	32	19	51	16:00
HI9HAF202BWGZ3	AT4G02780	GA1	Terpenoid cyclases/Protein prenyltransferases superfamily protein	1.0E-28	–	32	18	50	12:00
isotig03680	AT5G55250	IAMT1	IAA carboxylmethyltransferase 1	3.0E-29	–	32	17	49	8:00
HI9HAF203C8P6E	AT4G02290	AtGH9B13	glycosyl hydrolase 9B13	2.0E-63	–	32	16	48	24:00
Shoot-027-76	AT3G50950	ZAR1	HOPZ-ACTIVATED RESISTANCE 1	7.0E-14	–	32	14	46	4:00
HI9HAF203DIEJ2	AT5G61380	TOC1	CCT motif -containing response regulator protein	9.0E-21	CjTOC1	32	10	42	20:00
HI9HAF203DAPBQ	AT2G36190	AtcwINV4	cell wall invertase 4	3.0E-42	–	31	33	64	20:00
isotig03635	AT4G11650	ATOSM34	osmotin 34	5.0E-63	–	31	29	60	20:00
isotig05458	AT1G70000	–	myb-like transcription factor family protein	8.0E-29	–	31	28	59	20:00
HI9HAF202BSJ1B	AT2G34650	PID	Protein kinase superfamily protein	8.0E-43	–	31	24	55	12:00
HI9HAF203DK482	AT5G06530	–	ABC-2 type transporter family protein	2.0E-46	–	31	22	53	4:00
isotig03850	AT5G01410	PDX1	Aldolase-type TIM barrel family protein	1.0E-83	–	31	18	49	12:00
isotig02884	–	–	–	–	–	31	17	48	8:00
Shoot-004-26_004_B04	AT4G22740	–	glycine-rich protein	3.0E-25	–	31	17	48	12:00
HI9HAF202BWSJY	AT5G60900	RLK1	receptor-like protein kinase 1	7.0E-47	–	31	17	48	4:00
HI9HAF203DIF6L	AT5G02500	HSC70-1	heat shock cognate protein 70-1	1.0E-79	–	31	16	47	12:00
SSH24-6-07_013_G01	–	–	–	–	–	31	15	46	4:00
HI9HAF202CD6L8	AT4G21585	ENDO4	endonuclease 4	6.0E-55	–	31	14	45	24:00
HI9HAF202BW9MV	AT4G18750	DOT4	Pentatricopeptide repeat (PPR) superfamily protein	2.0E-42	–	31	12	43	16:00
isotig05771	AT5G12020	HSP17.6II	17.6 kDa class II heat shock protein	2.0E-33	–	31	9	40	12:00
Shoot-004-66_003_B09	AT2G42800	AtRLP29	receptor like protein 29	6.0E-61	–	31	9	40	24:00
Shoot-009-84_007_D11	AT3G49200	–	O-acyltransferase (WSD1-like) family protein	4.0E-26	–	31	3	34	24:00
isotig00695	AT3G19430	–	late embryogenesis abundant protein-related / LEA protein-related	2.0E-69	–	30	31	61	16:00
Shoot-025-13	AT3G20660	AtOCT4	organic cation/carnitine transporter4	1.0E-45	–	30	30	60	8:00
HI9HAF203DH875	AT5G05340	–	Peroxidase superfamily protein	3.0E-32	–	30	25	55	4:00
HI9HAF203DEFZZ	AT1G22770	GI	gigantea protein (GI)	6.0E-25	CjGI	30	23	53	16:00
SSH12-5-32_016_H04	AT3G15850	FAD5	fatty acid desaturase 5	4.0E-26	–	30	23	53	16:00
HI9HAF203CYQ64	–	–	–	–	–	30	22	52	16:00
isotig04903	AT4G27670	HSP21	heat shock protein 21	7.0E-53	–	30	20	50	12:00
isotig01364	AT2G35710	–	Nucleotide-diphospho-sugar transferases superfamily protein	1.0E-159	–	30	20	50	16:00
Shoot-007-63_014_G08	–	–	–	–	–	30	18	48	24:00
HI9HAF202B1CBE	AT1G67110	CYP735A2	cytochrome P450, family 735, subfamily A, polypeptide 2	9.0E-23	–	30	18	48	20:00
isotig06630	AT5G16010	–	3-oxo-5-alpha-steroid 4-dehydrogenase family protein	5.0E-33	–	30	18	48	8:00
HI9HAF202CEBY0	AT5G46240	KAT1	potassium channel in Arabidopsis thaliana 1	3.0E-46	–	30	18	48	8:00
HI9HAF203DARNP	AT1G22770	GI	gigantea protein (GI)	1.0E-11	CjGI	30	16	46	16:00
HI9HAF202B5TE9	AT1G26560	BGLU40	beta glucosidase 40	4.0E-49	–	30	15	45	20:00
isotig06422	AT2G47710	–	Adenine nucleotide alpha hydrolases-like superfamily protein	7.0E-50	–	30	11	41	12:00
Shoot-030-15	–	–	–	–	–	30	8	38	4:00
HI9HAF202CIX0P	AT3G23990	HSP60	heat shock protein 60	6.0E-59	–	30	5	35	12:00
isotig05965	AT5G25280	–	serine-rich protein-related	1.0E-15	–	29	35	64	16:00
Shoot-047-77	–	–	–	–	–	29	33	62	16:00
HI9HAF202B521Z	AT5G15700	–	DNA/RNA polymerases superfamily protein	1.0E-44	–	29	32	61	16:00
isotig06339	AT4G24220	VEP1	NAD(P)-binding Rossmann-fold superfamily protein	6.0E-46	–	29	27	56	12:00
HI9HAF203DSDCM	AT1G15500	ATNTT2	TLC ATP/ADP transporter	3.0E-61	–	29	27	56	20:00
HI9HAF203C1K4S	AT2G44500	–	O-fucosyltransferase family protein	9.0E-46	–	29	26	55	4:00
HI9HAF202BYAVK	AT2G29380	HAI3	highly ABA-induced PP2C gene 3	6.0E-17	–	29	24	53	16:00
HI9HAF202B9SF1	–	–	–	–	–	29	22	51	24:00
SSH24-8-71_013_G09	–	–	–	–	–	29	21	50	4:00
HI9HAF202BRPA4	AT3G22400	LOX5	PLAT/LH2 domain-containing lipoxygenase family protein	1.0E-36	–	29	21	50	20:00
Shoot-058-64	–	–	–	–	–	29	20	49	16:00
HI9HAF203C8QS4	AT4G30780	–	unknown protein	4.0E-42	–	29	20	49	12:00
isotig00619	AT3G56630	CYP94D2	cytochrome P450, family 94, subfamily D, polypeptide 2	7.0E-65	–	29	20	49	4:00
Shoot-058-94	–	–	–	–	–	29	19	48	4:00
isotig05231	AT3G22840	ELIP1	Chlorophyll A-B binding family protein	8.0E-37	–	29	19	48	12:00
SSH24-5-91_006_C12	AT5G38212	–	Potential natural antisense gene, locus overlaps with AT5G38210	3.0E-12	–	29	18	47	4:00
isotig06524	AT5G52420	–	unknown protein	1.0E-26	–	29	16	45	16:00
Shoot-050-49	AT5G52420	–	unknown protein	4.0E-42	–	29	16	45	16:00
HI9HAF202B8HFI	AT3G10210	–	SEC14 cytosolic factor family protein / phosphoglyceride transfer family protein	1.0E-49	–	29	16	45	20:00
isotig06231	–	–	–	–	–	29	14	43	16:00
HI9HAF203DQ56R	AT2G02170	–	Remorin family protein	1.0E-40	–	29	14	43	24:00
isotig06323	–	–	–	–	–	29	11	40	16:00
HI9HAF203DLCSA	AT3G23990	HSP60	heat shock protein 60	4.0E-64	–	29	10	39	12:00
isotig06797	–	–	–	–	–	29	9	38	24:00
HI9HAF203DKAPN	AT5G46050	ATPTR3	peptide transporter 3	6.0E-47	–	28	37	65	8:00
Shoot-052-12	AT2G40460	–	Major facilitator superfamily protein	1.0E-46	–	28	33	61	8:00
HI9HAF202CLAJI	AT3G12500	ATHCHIB	basic chitinase	2.0E-51	–	28	28	56	16:00
HI9HAF202BZ3FJ	AT3G23330	–	Tetratricopeptide repeat (TPR)-like superfamily protein	2.0E-46	–	28	27	55	16:00
Shoot-056-35	–	–	–	–	–	28	26	54	16:00
isotig02760	AT5G20190	–	Tetratricopeptide repeat (TPR)-like superfamily protein	7.0E-35	–	28	26	54	16:00
isotig03734	AT2G21320	–	B-box zinc finger family protein	2.0E-27	–	28	24	52	8:00
HI9HAF202BTYDK	AT2G38310	PYL4	PYR1-like 4	9.0E-48	–	28	23	51	16:00
HI9HAF203DSTNE	–	–	–	–	–	28	22	50	24:00
SSH24-2-38_011_F05	–	–	–	–	–	28	21	49	12:00
isotig00765	AT4G27250	–	NAD(P)-binding Rossmann-fold superfamily protein	1.0E-41	–	28	21	49	4:00
SSH12-5-49_001_A07	–	–	–	–	–	28	20	48	8:00
Shoot-004-35_005_C05	AT3G10210	–	SEC14 cytosolic factor family protein / phosphoglyceride transfer family protein	8.0E-28	–	28	19	47	20:00
HI9HAF203CYISG	AT4G29520	–	–	6.0E-46	–	28	19	47	12:00
isotig01511	AT2G16570	ATASE	GLN phosphoribosyl pyrophosphate amidotransferase 1	0.0	–	28	19	47	8:00
HI9HAF202CKKIQ	AT3G43120	–	SAUR-like auxin-responsive protein family	5.0E-25	–	28	18	46	12:00
isotig05436	AT3G22840	ELIP1	Chlorophyll A-B binding family protein	1.0E-35	–	28	18	46	12:00
HI9HAF202BTXTW	AT3G18110	EMB1270	Pentatricopeptide repeat (PPR) superfamily protein	4.0E-44	–	28	18	46	16:00
isotig00992	–	–	–	–	–	28	17	45	8:00
HI9HAF203C7O97	AT5G02810	PRR7	pseudo-response regulator 7	4.0E-39	–	28	17	45	16:00
HI9HAF202CJUSS	AT1G22770	GI	gigantea protein (GI)	1.0E-44	CjGI	28	15	43	16:00
Shoot-047-23	AT2G38640	–	Protein of unknown function (DUF567)	3.0E-19	–	28	14	42	12:00

HI9HAF202B2W3I	AT1G56410	ERD2	heat shock protein 70 (Hsp 70) family protein	2.0E-59	–	28	14	42	12:00
isotig03907	AT1G10760	SEX1	Pyruvate phosphate dikinase, PEP/pyruvate binding domain	9.0E-74	–	28	14	42	20:00
isotig05855	AT3G15810	–	Protein of unknown function (DUF567)	1.0E-35	–	28	13	41	12:00
HI9HAF202B7HJR	AT3G16857	ARR1	response regulator 1	8.0E-13	–	28	10	38	4:00
HI9HAF202CH7IL	AT5G62230	ERL1	ERECTA-like 1	2.0E-51	–	28	7	35	24:00
HI9HAF203C1W1J	AT3G11410	ATPP2CA	protein phosphatase 2CA	1.0E-34	–	27	31	58	16:00
SSH24-1-89_002_A12	AT4G33625	–	–	9.0E-24	–	27	28	55	16:00
isotig03041	AT5G49120	–	Protein of unknown function (DUF581)	4.0E-14	–	27	27	54	16:00
HI9HAF203DRHQF	AT5G08380	AtAGAL1	alpha-galactosidase 1	2.0E-50	–	27	26	53	8:00
Shoot-021-70_011_F09	AT5G56300	GAMT2	gibberellic acid methyltransferase 2	3.0E-24	–	27	25	52	8:00
isotig05160	AT2G43620	–	Chitinase family protein	2.0E-57	–	27	25	52	20:00
HI9HAF202CI8OC	AT1G20190	ATEXPA11	expansin 11	1.0E-68	–	27	25	52	12:00
isotig04207	AT5G49920	–	Octicosapeptide/Phox/Bem1p family protein	1.0E-23	–	27	23	50	20:00
isotig05838	–	–	–	–	–	27	22	49	8:00
isotig04359	AT4G14690	ELIP2	Chlorophyll A-B binding family protein	7.0E-36	–	27	21	48	12:00
HI9HAF203C8M3O	AT1G22400	UGT85A1	UDP-Glycosyltransferase superfamily protein	5.0E-29	–	27	20	47	20:00
HI9HAF202BUGL9	AT5G15450	APG6	casein lytic proteinase B3	6.0E-47	–	27	20	47	12:00
isotig00466	AT5G52640	HSP81-1	heat shock protein 90.1	0.0	–	27	20	47	12:00
Shoot-023-14	AT3G22840	ELIP1	Chlorophyll A-B binding family protein	2.0E-45	–	27	18	45	12:00
Shoot-039-84	AT4G29520	–	–	4.0E-54	–	27	16	43	12:00
SSH24-4-31_014_G04	–	–	–	–	–	27	15	42	24:00
isotig00135	AT4G28940	–	Phosphorylase superfamily protein	8.0E-50	–	27	14	41	20:00
isotig05282	AT1G09560	GLP5	germin-like protein 5	6.0E-41	–	27	13	40	24:00
isotig02598	AT3G14200	–	Chaperone DnaJ-domain superfamily protein	4.0E-28	–	27	12	39	12:00
HI9HAF203DKXCX	AT1G67710	ARR11	response regulator 11	6.0E-22	–	27	11	38	4:00
isotig05463	–	–	–	–	–	27	9	36	20:00
HI9HAF202BQECV	AT5G57800	FLP1	Fatty acid hydroxylase superfamily	6.0E-49	–	27	7	34	24:00
isotig05849	–	–	–	–	–	27	6	33	20:00
HI9HAF203DRD6C	AT3G23990	HSP60	heat shock protein 60	6.0E-62	–	27	4	31	12:00
HI9HAF202CGCUN	AT3G23990	HSP60	heat shock protein 60	5.0E-59	–	27	3	30	12:00
isotig01977	AT2G42850	CYP718	cytochrome P450, family 718	7.0E-73	–	26	30	56	20:00
HI9HAF203DMPHF	AT1G69830	ATAMY3	alpha-amylase-like 3	2.0E-75	–	26	28	54	20:00
HI9HAF203DI9IO	AT4G02780	GA1	Terpenoid cyclases/Protein prenyltransferases superfamily protein	3.0E-12	–	26	27	53	4:00
HI9HAF202BUOWC	AT3G27020	YSL6	YELLOW STRIPE like 6	2.0E-49	–	26	27	53	16:00
isotig04059	AT2G21100	–	Disease resistance-responsive (dirigent-like protein) family protein	1.0E-45	–	26	24	50	16:00
HI9HAF202CEMXR	AT2G42980	–	Eukaryotic aspartyl protease family protein	4.0E-49	–	26	24	50	4:00
isotig03141	AT1G77490	TAPX	thylakoidal ascorbate peroxidase	5.0E-133	–	26	24	50	16:00
HI9HAF202BZBCB	AT2G33860	ETT	Transcriptional factor B3 family protein / auxin-responsive factor AUX/IAA-related	2.0E-12	–	26	23	49	16:00
HI9HAF203C8YEA	AT1G20190	ATEXPA11	expansin 11	1.0E-71	–	26	23	49	12:00
HI9HAF203C3I81	AT5G08370	AtAGAL2	alpha-galactosidase 2	5.0E-49	–	26	22	48	4:00
Shoot-057-21	–	–	–	–	–	26	21	47	16:00
Shoot-006-27_006_C04	AT5G17540	–	HXXXD-type acyl-transferase family protein	1.0E-21	–	26	19	45	12:00
isotig04918	–	–	–	–	–	26	18	44	24:00
HI9HAF203DCXWT	AT1G02800	ATCEL2	cellulase 2	4.0E-62	–	26	17	43	24:00
isotig00136	AT4G28940	–	Phosphorylase superfamily protein	3.0E-49	–	26	16	42	20:00
isotig04246	AT5G57360	ZTL	Galactose oxidase/kelch repeat superfamily protein	1.0E-38	CjZTL	26	13	39	16:00
isotig05100	AT5G09590	MTHSC70-2	mitochondrial HSO70 2	4.0E-91	–	26	13	39	12:00
isotig04640	AT1G76990	ACR3	ACT domain repeat 3	3.0E-39	–	26	12	38	8:00
HI9HAF203DPAN6	AT1G08260	EMB142	DNA polymerase epsilon catalytic subunit	1.0E-70	–	26	11	37	24:00
isotig00303	–	–	–	–	–	26	10	36	20:00
Shoot-010-76	–	–	–	–	–	26	9	35	24:00
HI9HAF202B4RYN	AT5G57800	FLP1	Fatty acid hydroxylase superfamily	8.0E-63	–	26	8	34	24:00
Shoot-012-12_008_D02	–	–	–	–	–	26	7	33	24:00
HI9HAF202B9OL5	AT5G62230	ERL1	ERECTA-like 1	9.0E-69	–	26	7	33	24:00
Shoot-048-54	AT2G44830	–	Protein kinase superfamily protein	1.0E-48	–	25	40	65	16:00
Shoot-047-72	–	–	–	–	–	25	33	58	4:00
isotig06717	–	–	–	–	–	25	31	56	24:00
SSH12-7-75_006_C10	–	–	–	–	–	25	28	53	12:00
isotig06700	AT4G39700	–	Heavy metal transport/detoxification superfamily protein	3.0E-11	–	25	28	53	8:00
Shoot-023-66	AT3G59480	–	pfkB-like carbohydrate kinase family protein	2.0E-36	–	25	28	53	16:00
Shoot-003-39_013_G05	AT5G51260	–	HAD superfamily, subfamily IIIB acid phosphatase	1.0E-48	–	25	28	53	12:00
isotig02233	AT3G15850	FAD5	fatty acid desaturase 5	2.0E-130	–	25	28	53	16:00
SSH24-8-04_007_D01	–	–	–	–	–	25	25	50	8:00
HI9HAF203C5G81	AT3G27020	YSL6	YELLOW STRIPE like 6	2.0E-58	–	25	25	50	16:00
SSH24-3-76_008_D10	–	–	–	–	–	25	23	48	4:00
HI9HAF202BY98G	–	–	–	–	–	25	23	48	24:00
isotig03922	AT1G68090	ANNAT5	annexin 5	8.0E-54	–	25	22	47	16:00
isotig02293	AT1G74310	ATHSP101	heat shock protein 101	5.0E-162	–	25	19	44	12:00
HI9HAF203DQAN0	AT1G68750	ATPPC4	phosphoenolpyruvate carboxylase 4	3.0E-51	–	25	15	40	16:00
HI9HAF202CMH87	AT5G15250	FTSH6	FTSH protease 6	1.0E-65	–	25	15	40	12:00
HI9HAF203DKXQU	AT5G19730	–	Pectin lyase-like superfamily protein	1.0E-46	–	25	14	39	24:00
HI9HAF203C2JPL	AT5G48600	SMC3	structural maintenance of chromosome 3	3.0E-61	–	25	13	38	12:00
isotig05800	–	–	–	–	–	25	12	37	4:00
HI9HAF202B62CT	AT1G01060	LHY	Homeodomain-like superfamily protein	8.0E-24	CjLHYa	25	12	37	4:00
HI9HAF203DKI0U	AT2G36690	–	2-oxoglutarate (2OG) and Fe(II)-dependent oxygenase superfamily protein	6.0E-41	–	25	12	37	8:00
isotig04325	–	–	–	–	–	25	9	34	12:00
SSH12-2-09_002_A02	AT1G01120	KCS1	3-ketoacyl-CoA synthase 1	1.0E-39	–	25	4	29	24:00
SSH24-9-33_001_A05	–	–	–	–	–	24	32	56	4:00
isotig04068	–	–	–	–	–	24	30	54	8:00
HI9HAF202BVW5Y	AT2G45560	CYP76C1	cytochrome P450, family 76, subfamily C, polypeptide 1	1.0E-41	–	24	30	54	16:00
SSH12-6-57_002_A08	AT5G42760	–	Leucine carboxyl methyltransferase	8.0E-19	–	24	29	53	8:00
HI9HAF202B325D	AT2G42980	–	Eukaryotic aspartyl protease family protein	1.0E-49	–	24	29	53	4:00
HI9HAF203DIMRN	AT4G00110	GAE3	UDP-D-glucuronate 4-epimerase 3	7.0E-69	–	24	28	52	4:00
HI9HAF203C5R45	–	–	–	–	–	24	27	51	24:00
isotig03482	AT5G08640	FLS1	flavonol synthase 1	9.0E-91	–	24	25	49	4:00
Shoot-012-62_012_F08	AT5G11260	HY5	Basic-leucine zipper (bZIP) transcription factor family protein	3.0E-21	–	24	24	48	12:00
Shoot-048-08	AT3G53100	–	GDSL-like Lipase/Acylhydrolase superfamily protein	6.0E-34	–	24	19	43	24:00
HI9HAF203DQLPJ	AT1G10760	SEX1	Pyruvate phosphate dikinase, PEP/pyruvate binding domain	3.0E-42	–	24	19	43	20:00
isotig04093	AT3G23770	–	O-Glycosyl hydrolases family 17 protein	6.0E-59	–	24	19	43	24:00
HI9HAF202CAZ5B	AT3G24120	–	Homeodomain-like superfamily protein	1.0E-42	–	24	18	42	12:00
HI9HAF202CCLEQ	AT5G62410	SMC2	structural maintenance of chromosomes 2	2.0E-66	–	24	17	41	24:00
Shoot-029-52	–	–	–	–	–	24	16	40	20:00
HI9HAF202BUUZ9	AT3G62040	–	Haloacid dehalogenase-like hydrolase (HAD) superfamily protein	3.0E-46	–	24	16	40	8:00
isotig06740	–	–	–	–	–	24	15	39	12:00
HI9HAF203DI21I	–	–	–	–	–	24	14	38	20:00
isotig03173	AT2G22840	AtGRF1	growth-regulating factor 1	1.0E-40	–	24	14	38	24:00
HI9HAF203DG1OH	AT2G02170	–	Remorin family protein	1.0E-40	–	24	12	36	24:00
HI9HAF203C6DI9	AT3G47890	–	Ubiquitin carboxyl-terminal hydrolase-related protein	2.0E-51	–	24	9	33	12:00
isotig01039	–	–	–	–	–	24	7	31	20:00
Shoot-052-73	AT1G47550	SEC3A	exocyst complex component sec3A	1.0E-44	–	24	3	27	12:00
isotig02747	–	–	–	–	–	23	34	57	20:00
HI9HAF202BUCFB	AT1G22400	UGT85A1	UDP-Glycosyltransferase superfamily protein	2.0E-14	–	23	33	56	16:00
Shoot-010-48	AT5G47390	–	myb-like transcription factor family protein	2.0E-46	–	23	29	52	20:00
isotig03266	AT5G14700	–	NAD(P)-binding Rossmann-fold superfamily protein	3.0E-59	–	23	28	51	4:00
Shoot-052-68	–	–	–	–	–	23	27	50	4:00

Shoot-017-36_007_D05	AT1G18660	–	zinc finger (C3HC4-type RING finger) family protein	2.0E-40	–	23	27	50	8:00
isotig00868	AT2G26710	BAS1	Cytochrome P450 superfamily protein	4.0E-79	–	23	27	50	16:00
Shoot-007-77_010_E10	–	–	–	–	–	23	25	48	16:00
Shoot-002-88_015_H11	AT4G24220	VEP1	NAD(P)-binding Rossmann-fold superfamily protein	3.0E-35	–	23	23	46	12:00
HI9HAF203DQ59H	AT1G10550	XTH33	xyloglucan:xyloglucosyl transferase 33	1.0E-45	–	23	23	46	24:00
isotig00267	AT1G21460	SWEET1	Nodulin MtN3 family protein	3.0E-54	–	23	22	45	8:00
isotig01143	–	–	–	–	–	23	21	44	8:00
HI9HAF203DB0DT	AT4G24210	SLY1	F-box family protein	5.0E-23	–	23	21	44	8:00
isotig02115	AT5G22090	–	Protein of unknown function (DUF3049)	3.0E-15	–	23	20	43	8:00
Shoot-007-70_011_F09	AT3G62600	ATERDJ3B	DNAJ heat shock family protein	9.0E-52	–	23	20	43	12:00
Shoot-012-61_010_E08	–	–	–	–	–	23	18	41	24:00
HI9HAF203C6495	AT1G08550	NPQ1	non-photochemical quenching 1	5.0E-30	–	23	18	41	4:00
Shoot-059-56	AT5G16010	–	3-oxo-5-alpha-steroid 4-dehydrogenase family protein	2.0E-36	–	23	16	39	8:00
isotig01223	AT4G24190	SHD	Chaperone protein htpG family protein	0.0	–	23	16	39	12:00
HI9HAF203C2ZM8	AT1G01060	LHY	Homeodomain-like superfamily protein	2.0E-17	CjLHYa	23	14	37	4:00
SSH24-5-19_005_C03	–	–	–	–	–	23	11	34	12:00
Shoot-057-72	AT1G25450	KCS5	3-ketoacyl-CoA synthase 5	4.0E-36	–	23	7	30	24:00
Shoot-058-24	AT1G14410	ATWHY1	ssDNA-binding transcriptional regulator	3.0E-39	–	23	2	25	12:00
HI9HAF202B1QMH	ATMG00070	NAD9	NADH dehydrogenase subunit 9	5.0E-58	–	23	2	25	8:00
Shoot-002-05_009_E01	AT1G65450	–	HXXXD-type acyl-transferase family protein	1.0E-45	–	23	1	24	24:00
isotig02073	AT3G48990	–	AMP-dependent synthetase and ligase family protein	2.0E-175	–	22	38	60	4:00
Shoot-013-23_013_G03	AT2G01770	VIT1	vacuolar iron transporter 1	5.0E-91	–	22	36	58	12:00
HI9HAF203C0AMS	AT2G27610	–	Tetratricopeptide repeat (TPR)-like superfamily protein	5.0E-47	–	22	32	54	16:00
isotig05161	AT3G45970	ATEXLA1	expansin-like A1	5.0E-72	–	22	30	52	4:00
Shoot-024-62	–	–	–	–	–	22	28	50	12:00
Shoot-059-71	AT3G45960	ATEXLA3	expansin-like A3	3.0E-65	–	22	28	50	4:00
isotig06786	–	–	–	–	–	22	26	48	4:00
isotig04614	AT1G33960	AIG1	P-loop containing nucleoside triphosphate hydrolases superfamily protein	5.0E-40	–	22	26	48	20:00
HI9HAF202CAVDN	AT3G26744	ICE1	basic helix-loop-helix (bHLH) DNA-binding superfamily protein	5.0E-59	–	22	26	48	16:00
isotig04273	AT5G64260	EXL2	EXORDIUM like 2	3.0E-69	–	22	26	48	4:00
HI9HAF202CJSRE	AT2G38310	PYL4	PYR1-like 4	3.0E-34	–	22	25	47	16:00
HI9HAF203DCBF0	AT5G14040	PHT3;1	phosphate transporter 3;1	2.0E-61	–	22	25	47	16:00
isotig04743	AT3G22840	ELIP1	Chlorophyll A-B binding family protein	2.0E-37	–	22	23	45	12:00
isotig04703	AT5G59580	UGT76E1	UDP-glucosyl transferase 76E1	7.0E-42	–	22	23	45	4:00
HI9HAF203DC17O	AT5G59720	HSP18.2	heat shock protein 18.2	2.0E-53	–	22	23	45	12:00
Shoot-005-74_004_B10	AT2G12646	–	PLATZ transcription factor family protein	2.0E-71	–	22	22	44	12:00
HI9HAF203DISBK	AT5G60900	RLK1	receptor-like protein kinase 1	6.0E-49	–	22	20	42	4:00
isotig04175	–	–	–	–	–	22	19	41	12:00
isotig04031	–	–	–	–	–	22	19	41	20:00
SSH24-5-17_001_A03	AT5G55180	–	O-Glycosyl hydrolases family 17 protein	1.0E-33	–	22	19	41	24:00
HI9HAF202BZPBJ	–	–	–	–	–	22	13	35	12:00
isotig01089	–	–	–	–	–	22	13	35	16:00
HI9HAF202B5TPI	AT5G15450	APG6	casein lytic proteinase B3	2.0E-42	–	22	11	33	12:00
isotig01038	–	–	–	–	–	22	10	32	20:00
HI9HAF203DHATH	AT1G30950	UFO	F-box family protein	5.0E-49	–	22	9	31	12:00
HI9HAF203C8C1Q	AT3G04720	PR4	pathogenesis-related 4	4.0E-54	–	22	8	30	4:00
HI9HAF203DB8LO	AT2G06050	OPR3	oxophytodienoate-reductase 3	3.0E-24	–	21	39	60	4:00
isotig03530	AT1G69530	ATEXPA1	expansin A1	5.0E-100	–	21	32	53	4:00
isotig04730	AT3G26510	–	Octicosapeptide/Phox/Bem1p family protein	4.0E-27	–	21	31	52	12:00
isotig01419	AT2G13610	–	ABC-2 type transporter family protein	4.0E-154	–	21	30	51	8:00
isotig06099	AT2G47180	AtGolS1	galactinol synthase 1	5.0E-57	–	21	28	49	12:00
isotig04066	AT3G54850	ATPUB14	plant U-box 14	2.0E-64	–	21	28	49	4:00
isotig04852	–	–	–	–	–	21	27	48	4:00
isotig02597	AT5G64170	–	dentin sialophosphoprotein-related	2.0E-12	–	21	26	47	12:00
isotig05450	AT1G27330	–	Ribosome associated membrane protein RAMP4	1.0E-16	–	21	26	47	12:00
Shoot-050-73	AT2G44830	–	Protein kinase superfamily protein	2.0E-26	–	21	26	47	16:00
HI9HAF202B2WQN	–	–	–	–	–	21	25	46	24:00
HI9HAF203DHXW9	AT3G09640	APX2	ascorbate peroxidase 2	4.0E-67	–	21	25	46	20:00
Shoot-056-18	AT2G12646	–	PLATZ transcription factor family protein	3.0E-70	–	21	25	46	12:00
isotig05089	AT4G30320	–	CAP (Cysteine-rich secretory proteins, Antigen 5, and Pathogenesis-related 1 protein) superfamily protein	2.0E-53	–	21	24	45	4:00
isotig00767	–	–	–	–	–	21	23	44	8:00
Shoot-052-87	AT4G01630	ATEXPA17	expansin A17	5.0E-63	–	21	23	44	24:00
isotig03688	–	–	–	–	–	21	22	43	20:00
isotig00117	AT3G22840	ELIP1	Chlorophyll A-B binding family protein	4.0E-40	–	21	22	43	12:00
HI9HAF203DROMK	AT1G71695	–	Peroxidase superfamily protein	4.0E-50	–	21	22	43	20:00
HI9HAF203DPU17	–	–	–	–	–	21	21	42	4:00
SSH24-4-94_012_F12	–	–	–	–	–	21	21	42	4:00
HI9HAF203DC47B	AT1G71695	–	Peroxidase superfamily protein	4.0E-50	–	21	21	42	20:00
isotig04234	AT4G14760	–	kinase interacting (KIP1-like) family protein	5.0E-13	–	21	20	41	8:00
isotig05650	–	–	–	–	–	21	18	39	20:00
HI9HAF203DD229	AT1G33970	–	P-loop containing nucleoside triphosphate hydrolases superfamily protein	2.0E-43	–	21	17	38	20:00
HI9HAF202CL26P	AT5G15700	–	DNA/RNA polymerases superfamily protein	3.0E-44	–	21	17	38	16:00
isotig06252	AT3G23990	HSP60	heat shock protein 60	2.0E-62	–	21	17	38	12:00
isotig02602	ATCG01130	YCF1.2	Ycf1 protein	2.0E-17	–	21	12	33	24:00
isotig06376	AT2G38080	IRX12	Laccase/Diphenol oxidase family protein	1.0E-44	–	21	12	33	24:00
HI9HAF203DHIWR	AT3G47910	–	Ubiquitin carboxyl-terminal hydrolase-related protein	1.0E-56	–	21	12	33	12:00
SSH12-4-71_013_G09	–	–	–	–	–	21	7	28	12:00
isotig04781	AT1G65450	–	HXXXD-type acyl-transferase family protein	3.0E-31	–	21	3	24	24:00
isotig04131	AT4G11650	ATOSM34	osmotin 34	4.0E-66	–	20	40	60	16:00
Shoot-054-07	–	–	–	–	–	20	35	55	4:00
isotig04982	AT4G31970	CYP82C2	cytochrome P450, family 82, subfamily C, polypeptide 2	8.0E-43	–	20	33	53	4:00
HI9HAF202CLCU0	AT3G26744	ICE1	basic helix-loop-helix (bHLH) DNA-binding superfamily protein	7.0E-59	–	20	32	52	16:00
SSH24-2-06_011_F01	–	–	–	–	–	20	30	50	16:00
isotig06315	AT4G19160	–	unknown protein	5.0E-46	–	20	30	50	20:00
HI9HAF202BS5O6	AT2G18150	–	Peroxidase superfamily protein	2.0E-16	–	20	29	49	16:00
isotig06357	AT3G10020	–	unknown protein	4.0E-13	–	20	26	46	12:00
isotig05015	AT1G71050	HIPP20	Heavy metal transport/detoxification superfamily protein	3.0E-38	–	20	25	45	16:00
HI9HAF202B8ZAL	AT5G08370	AtAGAL2	alpha-galactosidase 2	9.0E-50	–	20	23	43	4:00
Shoot-029-15	–	–	–	–	–	20	22	42	4:00
Shoot-024-60	AT3G48320	CYP71A21	cytochrome P450, family 71, subfamily A, polypeptide 21	2.0E-28	–	20	22	42	24:00
isotig04935	AT3G09640	APX2	ascorbate peroxidase 2	2.0E-100	–	20	22	42	12:00
Shoot-027-69	AT1G45474	LHCA5	photosystem I light harvesting complex gene 5	1.0E-15	–	20	18	38	24:00
isotig01016	AT2G44360	–	unknown protein	5.0E-24	–	20	18	38	8:00
isotig00965	–	–	–	–	–	20	16	36	16:00
HI9HAF202B6NRH	AT3G02260	BIG	auxin transport protein (BIG)	3.0E-43	–	20	16	36	24:00
isotig03654	AT4G28940	–	Phosphorylase superfamily protein	7.0E-93	–	20	16	36	20:00
isotig04853	AT4G34350	CLB6	4-hydroxy-3-methylbut-2-enyl diphosphate reductase	6.0E-43	–	20	15	35	12:00
SSH12-6-51_005_C07	AT1G47620	CYP96A8	cytochrome P450, family 96, subfamily A, polypeptide 8	1.0E-27	–	20	14	34	24:00
isotig02783	AT2G26150	ATHSFA2	heat shock transcription factor A2	7.0E-50	–	20	14	34	20:00
HI9HAF202CFO1O	AT5G19730	–	Pectin lyase-like superfamily protein	4.0E-71	–	20	14	34	24:00
isotig04737	AT2G39050	–	hydroxyproline-rich glycoprotein family protein	6.0E-46	–	20	13	33	20:00
isotig05469	AT5G59720	HSP18.2	heat shock protein 18.2	1.0E-41	–	20	12	32	12:00
HI9HAF202B1YSW	AT3G10340	PAL4	phenylalanine ammonia-lyase 4	1.0E-73	–	20	11	31	4:00
HI9HAF202BRALA	AT2G45540	–	WD-40 repeat family protein / beige-related	1.0E-73	–	20	9	29	24:00
SSH12-2-17_001_A03	–	–	–	–	–	20	3	23	20:00

HI9HAF203CYV28	ATMG00070	NAD9	NADH dehydrogenase subunit 9	3.0E-65	－	20	3	23	8:00
HI9HAF203C8SUM	AT1G26780	MYB117	myb domain protein 117	9.0E-51	－	19	43	62	8:00
Shoot-006-19_005_C03	AT4G31970	CYP82C2	cytochrome P450, family 82, subfamily C, polypeptide 2	8.0E-37	－	19	40	59	4:00
HI9HAF203DDY6U	AT5G64120	－	Peroxidase superfamily protein	3.0E-22	－	19	39	58	8:00
Shoot-049-60	－	－	－	－	－	19	36	55	16:00
HI9HAF202CG9E7	AT2G06050	OPR3	oxophytodienoate-reductase 3	8.0E-24	－	19	35	54	4:00
HI9HAF203DOO3U	AT1G71695	－	Peroxidase superfamily protein	4.0E-42	－	19	32	51	20:00
Shoot-020-51_005_C07	－	－	－	－	－	19	28	47	24:00
HI9HAF203C40LU	AT5G38260	－	Protein kinase superfamily protein	4.0E-42	－	19	28	47	16:00
Shoot-003-47_014_G06	－	－	－	－	－	19	27	46	16:00
isotig06121	－	－	－	－	－	19	26	45	4:00
isotig01124	AT1G76140	－	Prolyl oligopeptidase family protein	4.0E-41	－	19	23	42	16:00
HI9HAF203C3CEJ	AT1G62440	LRX2	leucine-rich repeat/extensin 2	1.0E-48	－	19	22	41	24:00
isotig02108	AT5G40390	SIP1	Raffinose synthase family protein	0.0	－	19	22	41	12:00
HI9HAF202CE21S	AT1G14920	GAI	GRAS family transcription factor family protein	1.0E-46	－	19	18	37	24:00
HI9HAF202BX81J	AT1G01060	LHY	Homeodomain-like superfamily protein	9.0E-34	CjLHYa	19	17	36	4:00
isotig06395	AT2G39540	－	Gibberellin-regulated family protein	7.0E-23	－	19	15	34	4:00
HI9HAF202B9SZG	AT4G36080	－	phosphotransferases, alcohol group as acceptor;binding;inositol or phosphatidylinositol kinases	1.0E-51	－	19	15	34	24:00
isotig00832	－	－	－	－	－	19	14	33	24:00
Shoot-054-24	－	－	－	－	－	19	14	33	24:00
isotig04319	AT5G24090	ATCHIA	chitinase A	3.0E-80	－	19	14	33	4:00
isotig00950	－	－	－	－	－	19	13	32	12:00
SSH24-4-74_004_B10	－	－	－	－	－	19	4	23	24:00
SSH12-4-37_009_E05	－	－	－	－	－	19	3	22	20:00
HI9HAF202B8ET8	AT3G51895	SULTR3;1	sulfate transporter 3;1	2.0E-57	－	18	37	55	8:00
HI9HAF202BS1QV	AT4G25420	GA5	2-oxoglutarate (2OG) and Fe(II)-dependent oxygenase superfamily protein	1.0E-67	－	18	35	53	8:00
HI9HAF202B25D0	AT3G20440	EMB2729	Alpha amylase family protein	2.0E-63	－	18	32	50	24:00
isotig05093	AT5G48480	－	Lactoylglutathione lyase / glyoxalase I family protein	8.0E-21	－	18	31	49	20:00
isotig04606	－	－	－	－	－	18	30	48	8:00
HI9HAF202BURL0	AT4G18250	－	receptor serine/threonine kinase, putative	2.0E-41	－	18	30	48	16:00
HI9HAF202BWLCD	AT4G02900	－	ERD (early-responsive to dehydration stress) family protein	2.0E-64	－	18	29	47	8:00
isotig06728	－	－	－	－	－	18	24	42	4:00
HI9HAF202CE0PV	AT5G14040	PHT3;1	phosphate transporter 3;1	3.0E-57	－	18	24	42	16:00
isotig06186	－	－	－	－	－	18	23	41	8:00
HI9HAF203DCCSP	AT5G40390	SIP1	Raffinose synthase family protein	3.0E-42	－	18	23	41	12:00
HI9HAF203DE338	AT3G25230	ROF1	rotamase FKBP 1	6.0E-47	－	18	20	38	12:00
isotig05908	AT3G50170	－	Plant protein of unknown function (DUF247)	2.0E-14	－	18	19	37	8:00
HI9HAF203CZCIQ	AT3G25230	ROF1	rotamase FKBP 1	5.0E-41	－	18	18	36	12:00
isotig00968	－	－	－	－	－	18	14	32	20:00
isotig01537	AT1G01060	LHY	Homeodomain-like superfamily protein	1.0E-12	CjLHYa	18	14	32	4:00
HI9HAF203DGNH4	AT1G01060	LHY	Homeodomain-like superfamily protein	2.0E-17	CjLHYa	18	14	32	4:00
HI9HAF203C9FZX	AT1G64660	ATMGL	methionine gamma-lyase	6.0E-48	－	18	14	32	4:00
isotig04101	AT2G30870	ATGSTF10	glutathione S-transferase PHI 10	9.0E-15	－	18	13	31	8:00
HI9HAF203DOUVX	AT1G30950	UFO	F-box family protein	3.0E-44	－	18	11	29	12:00
SSH12-4-95_014_G12	－	－	－	－	－	18	7	25	24:00
isotig06361	AT4G17490	ATERF6	ethylene responsive element binding factor 6	7.0E-15	－	17	41	58	16:00
HI9HAF203CX03E	AT1G33440	－	Major facilitator superfamily protein	4.0E-47	－	17	38	55	4:00
isotig03847	AT1G26770	ATEXPA10	expansin A10	4.0E-108	－	17	31	48	12:00
isotig01057	AT4G03210	XTH9	xyloglucan endotransglucosylase/hydrolase 9	1.0E-39	－	17	30	47	20:00
HI9HAF203C30OA	AT3G20440	EMB2729	Alpha amylase family protein	3.0E-44	－	17	30	47	24:00
isotig04967	AT3G56290	－	unknown protein	7.0E-45	－	17	28	45	8:00
HI9HAF202CKUUU	AT4G18250	－	receptor serine/threonine kinase, putative	3.0E-41	－	17	27	44	16:00
Shoot-007-34_003_B05	AT5G13930	CHS	Chalcone and stilbene synthase family protein	6.0E-94	－	17	25	42	16:00
isotig00739	AT5G06570	－	alpha/beta-Hydrolases superfamily protein	1.0E-50	－	17	24	41	16:00
isotig03350	AT4G34350	CLB6	4-hydroxy-3-methylbut-2-enyl diphosphate reductase	2.0E-106	－	17	24	41	8:00
isotig03495	AT3G22840	ELIP1	Chlorophyll A-B binding family protein	3.0E-40	－	17	23	40	12:00
HI9HAF202B3WGH	AT5G03160	ATP58IPK	homolog of mamallian P58IPK	9.0E-41	－	17	21	38	12:00
Shoot-044-15	AT5G61380	TOC1	CCT motif -containing response regulator protein	1.0E-24	CjTOC1	17	20	37	20:00
HI9HAF203C2WNO	AT5G60100	PRR3	pseudo-response regulator 3	9.0E-42	PRR3	17	18	35	20:00
HI9HAF203DDWTO	AT3G62040	－	Haloacid dehalogenase-like hydrolase (HAD) superfamily protein	1.0E-42	－	17	15	32	8:00
HI9HAF203CXZG6	AT3G10340	PAL4	phenylalanine ammonia-lyase 4	1.0E-73	－	17	15	32	4:00
HI9HAF202B0U35	－	－	－	－	－	17	14	31	4:00
isotig01030	AT3G46230	ATHSP17.4	heat shock protein 17.4	3.0E-43	－	17	14	31	12:00
HI9HAF202BU8AU	AT3G47910	－	Ubiquitin carboxyl-terminal hydrolase-related protein	2.0E-55	－	17	14	31	12:00
HI9HAF202B5JHF	AT3G53260	PAL2	phenylalanine ammonia-lyase 2	1.0E-55	－	17	14	31	4:00
HI9HAF203DLZRB	AT1G70320	UPL2	ubiquitin-protein ligase 2	2.0E-44	－	17	13	30	8:00
HI9HAF203C1SBM	AT1G36160	ACC1	acetyl-CoA carboxylase 1	3.0E-55	－	17	8	25	8:00
SSH12-2-42_004_B06	－	－	－	－	－	17	5	22	24:00
Shoot-004-55_013_G07	AT5G11420	－	Protein of unknown function, DUF642	6.0E-83	－	16	41	57	8:00
isotig04229	－	－	－	－	－	16	37	53	16:00
isotig01742	AT4G21990	3-Apr	APS reductase 3	1.0E-167	－	16	37	53	8:00
HI9HAF203DDOCS	AT1G76690	OPR2	12-oxophytodienoate reductase 2	5.0E-59	－	16	34	50	12:00
SSH24-5-73_002_A10	AT5G01740	－	Nuclear transport factor 2 (NTF2) family protein	2.0E-27	－	16	31	47	4:00
HI9HAF203DNZO6	AT4G02780	GA1	Terpenoid cyclases/Protein prenyltransferases superfamily protein	6.0E-20	－	16	30	46	4:00
isotig01161	－	－	－	－	－	16	28	44	4:00
isotig01139	－	－	－	－	－	16	28	44	24:00
HI9HAF203C0S4T	AT2G03140	－	alpha/beta-Hydrolases superfamily protein	4.0E-58	－	16	28	44	8:00
isotig05870	AT3G14440	NCED3	nine-cis-epoxycarotenoid dioxygenase 3	2.0E-74	－	16	28	44	12:00
isotig05188	AT3G53000	AtPP2-A15	phloem protein 2-A15	3.0E-24	－	16	27	43	20:00
Shoot-055-81	AT1G71692	AGL12	AGAMOUS-like 12	8.0E-23	－	16	25	41	20:00
HI9HAF202B99QE	AT5G13930	CHS	Chalcone and stilbene synthase family protein	7.0E-45	－	16	25	41	16:00
isotig01433	－	－	－	－	－	16	24	40	20:00
HI9HAF203DQWR9	－	－	－	－	－	16	23	39	8:00
HI9HAF202B80AD	AT4G29130	ATHXK1	hexokinase 1	7.0E-63	－	16	23	39	12:00
HI9HAF202B0AKD	AT3G21620	－	ERD (early-responsive to dehydration stress) family protein	2.0E-57	－	16	22	38	4:00
HI9HAF202CD4C3	AT2G25140	HSP98.7	casein lytic proteinase B4	1.0E-63	－	16	20	36	12:00
SSH12-8-35_005_C05	－	－	－	－	－	16	19	35	8:00
HI9HAF202CLFKF	－	－	－	－	－	16	17	33	24:00
HI9HAF203DMJJE	AT3G57060	－	binding	6.0E-53	－	16	16	32	24:00
SSH24-8-06_011_F01	－	－	－	－	－	16	14	30	20:00
HI9HAF202B60K5	AT1G64660	ATMGL	methionine gamma-lyase	2.0E-56	－	16	14	30	4:00
HI9HAF202B3ZPC	AT1G16800	－	P-loop containing nucleoside triphosphate hydrolases superfamily protein	2.0E-41	－	16	12	28	12:00
isotig03659	AT1G03220	－	Eukaryotic aspartyl protease family protein	3.0E-58	－	16	6	22	4:00
Shoot-048-89	AT4G21960	PRXR1	Peroxidase superfamily protein	1.0E-71	－	15	40	55	16:00
isotig04228	AT5G01740	－	Nuclear transport factor 2 (NTF2) family protein	1.0E-28	－	15	38	53	4:00
isotig01782	AT5G42650	AOS	allene oxide synthase	1.0E-146	－	15	36	51	20:00
HI9HAF202B0UYM	AT2G21220	－	SAUR-like auxin-responsive protein family	3.0E-20	－	15	35	50	4:00
SSH12-7-83_005_C11	AT1G44760	－	Adenine nucleotide alpha hydrolases-like superfamily protein	4.0E-30	－	15	35	50	12:00
isotig00738	AT5G06570	－	alpha/beta-Hydrolases superfamily protein	7.0E-44	－	15	35	50	16:00
isotig00661	－	－	－	－	－	15	33	48	16:00
HI9HAF202B4JI6	AT2G21220	－	SAUR-like auxin-responsive protein family	3.0E-20	－	15	33	48	4:00
HI9HAF202CJU90	－	－	－	－	－	15	32	47	8:00
HI9HAF203CWS6S	AT1G26560	BGLU40	beta glucosidase 40	2.0E-44	－	15	32	47	20:00
HI9HAF202B13J6	AT5G56220	－	P-loop containing nucleoside triphosphate hydrolases superfamily protein	2.0E-45	－	15	31	46	24:00
HI9HAF203DBE55	AT2G41480	－	Peroxidase superfamily protein	9.0E-25	－	15	30	45	20:00

isotig05621	AT4G10260	−	pfkB-like carbohydrate kinase family protein	2.0E-84	−	15	27	42	16:00
isotig02214	AT1G27170	−	transmembrane receptors;ATP binding	6.0E-15	−	15	25	40	24:00
isotig00121	AT5G49690	−	UDP-Glycosyltransferase superfamily protein	3.0E-24	−	15	25	40	24:00
HI9HAF202B6RKD	AT1G59870	PEN3	ABC-2 and Plant PDR ABC-type transporter family protein	2.0E-56	−	15	25	40	4:00
HI9HAF203DHGZV	AT1G01120	KCS1	3-ketoacyl-CoA synthase 1	4.0E-56	−	15	24	39	12:00
Shoot-020-01_001_A01	AT1G61820	BGLU46	beta glucosidase 46	3.0E-75	−	15	24	39	4:00
isotig00304	−	−	−	−	−	15	23	38	4:00
HI9HAF202CE7G6	AT5G53460	GLT1	NADH-dependent glutamate synthase 1	8.0E-60	−	15	23	38	12:00
isotig00882	AT1G23740	−	Oxidoreductase, zinc-binding dehydrogenase family protein	9.0E-53	−	15	22	37	12:00
Shoot-057-11	AT5G46600	−	Aluminium activated malate transporter family protein	8.0E-27	−	15	20	35	4:00
Shoot-001-50_003_B07	AT1G15780	−	unknown protein	3.0E-19	−	15	19	34	4:00
HI9HAF202CFEVF	AT1G14360	ATUTR3	UDP-galactose transporter 3	3.0E-60	−	15	18	33	12:00
HI9HAF202CCJ64	−	−	−	−	−	15	13	28	12:00
SSH12-9-89_002_A12	−	−	−	−	−	15	13	28	24:00
HI9HAF202BUFP0	AT3G18110	EMB1270	Pentatricopeptide repeat (PPR) superfamily protein	9.0E-41	−	15	13	28	20:00
isotig00984	−	−	−	−	−	15	10	25	20:00
HI9HAF203DFEAJ	AT4G10770	ATOPT7	oligopeptide transporter 7	1.0E-65	−	14	44	58	4:00
Shoot-001-71_013_G09	AT4G32000	−	Protein kinase superfamily protein	8.0E-70	−	14	35	49	8:00
isotig05830	AT4G03290	−	EF hand calcium-binding protein family	6.0E-17	−	14	34	48	12:00
HI9HAF202BSU69	AT2G21220	−	SAUR-like auxin-responsive protein family	4.0E-20	−	14	34	48	4:00
Shoot-053-27	−	−	−	−	−	14	33	47	24:00
isotig00122	AT5G49690	−	UDP-Glycosyltransferase superfamily protein	4.0E-26	−	14	31	45	4:00
HI9HAF202CGABV	AT4G32300	SD2-5	S-domain-2 5	2.0E-43	−	14	31	45	16:00
Shoot-046-69	−	−	−	−	−	14	30	44	4:00
HI9HAF203DRR2U	AT1G60590	−	Pectin lyase-like superfamily protein	1.0E-45	−	14	30	44	16:00
isotig05509	AT4G11650	ATOSM34	osmotin 34	3.0E-59	−	14	30	44	16:00
Shoot-059-11	−	−	−	−	−	14	28	42	4:00
HI9HAF203DMYYA	AT1G20380	−	Prolyl oligopeptidase family protein	3.0E-44	−	14	28	42	16:00
isotig04794	−	−	−	−	−	14	27	41	16:00
isotig05097	AT3G25600	−	Calcium-binding EF-hand family protein	7.0E-22	−	14	26	40	16:00
Shoot-048-32	AT5G18170	GDH1	glutamate dehydrogenase 1	1.0E-129	−	14	26	40	16:00
HI9HAF203DR7YA	AT1G76140	−	Prolyl oligopeptidase family protein	5.0E-57	−	14	25	39	16:00
SSH24-8-92_008_D12	AT1G14520	MIOX1	myo-inositol oxygenase 1	4.0E-49	−	14	22	36	16:00
HI9HAF203CZRVR	AT2G06050	OPR3	oxophytodienoate-reductase 3	2.0E-43	−	14	21	35	12:00
HI9HAF202CAVXN	AT5G19730	−	Pectin lyase-like superfamily protein	4.0E-69	−	14	19	33	24:00
SSH12-8-58_004_B08	−	−	−	−	−	14	18	32	24:00
isotig01031	AT3G46230	ATHSP17.4	heat shock protein 17.4	5.0E-43	−	14	18	32	12:00
HI9HAF202BRY0E	AT2G25140	HSP98.7	casein lytic proteinase B4	2.0E-60	−	14	18	32	12:00
SSH12-3-26_004_B04	−	−	−	−	−	14	17	31	12:00
HI9HAF202CII3M	AT5G25460	−	Protein of unknown function, DUF642	5.0E-59	−	14	14	28	24:00
HI9HAF203DHL8Q	AT1G36160	ACC1	acetyl-CoA carboxylase 1	8.0E-78	−	14	11	25	8:00
SSH12-4-92_008_D12	ATMG00510	NAD7	NADH dehydrogenase subunit 7	1.0E-39	−	14	8	22	20:00
HI9HAF203DMDEZ	AT5G05340	−	Peroxidase superfamily protein	4.0E-38	−	13	46	59	8:00
SSH24-8-10_004_B02	−	−	−	−	−	13	42	55	20:00
isotig05126	AT3G10910	−	RING/U-box superfamily protein	1.0E-12	−	13	38	51	8:00
isotig02943	AT1G62500	−	Bifunctional inhibitor/lipid-transfer protein/seed storage 2S albumin superfamily protein	8.0E-25	−	13	38	51	20:00
isotig02964	AT4G16260	−	Glycosyl hydrolase superfamily protein	5.0E-81	−	13	35	48	4:00
HI9HAF203CYY76	AT2G18010	−	SAUR-like auxin-responsive protein family	4.0E-14	−	13	34	47	8:00
SSH24-8-26_004_B04	AT1G59870	PEN3	ABC-2 and Plant PDR ABC-type transporter family protein	3.0E-26	−	13	34	47	24:00
isotig04566	AT3G43660	−	Vacuolar iron transporter (VIT) family protein	2.0E-40	−	13	34	47	8:00
SSH24-6-09_002_A02	AT1G23740	−	Oxidoreductase, zinc-binding dehydrogenase family protein	2.0E-45	−	13	34	47	12:00
Shoot-039-26	−	−	−	−	−	13	31	44	12:00
isotig01151	AT1G32170	XTR4	xyloglucan endotransglucosylase/hydrolase 30	5.0E-29	−	13	31	44	20:00
HI9HAF203CY149	AT4G34760	−	SAUR-like auxin-responsive protein family	6.0E-23	−	13	30	43	8:00
HI9HAF203DIIVI	AT3G15510	ATNAC2	NAC domain containing protein 2	1.0E-62	−	13	29	42	16:00
SSH12-8-47_014_G06	−	−	−	−	−	13	27	40	8:00
isotig05977	−	−	−	−	−	13	27	40	16:00
isotig00985	−	−	−	−	−	13	27	40	20:00
HI9HAF203DBVBD	AT4G34760	−	SAUR-like auxin-responsive protein family	6.0E-23	−	13	26	39	8:00
HI9HAF203C3B9A	AT5G55950	−	Nucleotide/sugar transporter family protein	5.0E-49	−	13	26	39	16:00
HI9HAF203CXCAO	AT1G14360	ATUTR3	UDP-galactose transporter 3	7.0E-75	−	13	25	38	12:00
HI9HAF202BUXOK	AT1G64660	ATMGL	methionine gamma-lyase	3.0E-18	−	13	24	37	4:00
HI9HAF203C7NW7	AT1G79460	GA2	Terpenoid cyclases/Protein prenyltransferases superfamily protein	5.0E-19	−	13	24	37	4:00
SSH24-2-25_002_A04	−	−	−	−	−	13	23	36	12:00
isotig06302	AT1G33970	−	P-loop containing nucleoside triphosphate hydrolases superfamily protein	4.0E-38	−	13	23	36	20:00
HI9HAF202CIH7A	AT1G14920	GAI	GRAS family transcription factor family protein	4.0E-54	−	13	23	36	24:00
Shoot-055-62	AT4G34350	CLB6	4-hydroxy-3-methylbut-2-enyl diphosphate reductase	1.0E-106	−	13	23	36	8:00
isotig02436	ATCG00660	RPL20	ribosomal protein L20	6.0E-25	−	13	22	35	24:00
HI9HAF202BWF9P	AT1G14360	ATUTR3	UDP-galactose transporter 3	4.0E-56	−	13	22	35	12:00
HI9HAF203CZ34F	AT5G09790	ATXR5	ARABIDOPSIS TRITHORAX-RELATED PROTEIN 5	8.0E-49	−	13	20	33	12:00
isotig02091	AT1G74310	ATHSP101	heat shock protein 101	0.0	−	13	20	33	12:00
HI9HAF203DC217	AT1G64660	ATMGL	methionine gamma-lyase	7.0E-48	−	13	19	32	4:00
HI9HAF203C8JBI	AT4G10490	−	2-oxoglutarate (2OG) and Fe(II)-dependent oxygenase superfamily protein	2.0E-45	−	13	16	29	16:00
isotig01082	−	−	−	−	−	13	14	27	12:00
isotig00233	−	−	−	−	−	13	14	27	16:00
HI9HAF202B8EBO	AT1G36160	ACC1	acetyl-CoA carboxylase 1	1.0E-75	−	13	13	26	8:00
Shoot-003-04_007_D01	AT3G44400	−	Disease resistance protein (TIR-NBS-LRR class) family	9.0E-12	−	13	12	25	8:00
Shoot-007-52_007_D07	−	−	−	−	−	13	10	23	20:00
HI9HAF202BUZ7T	ATMG00510	NAD7	NADH dehydrogenase subunit 7	8.0E-75	−	13	7	20	20:00
isotig03640	−	−	−	−	−	12	45	57	20:00
isotig05273	AT5G08640	FLS1	flavonol synthase 1	2.0E-56	−	12	42	54	20:00
HI9HAF203CYA96	−	−	−	−	−	12	40	52	8:00
Shoot-046-67	AT2G03140	−	alpha/beta-Hydrolases superfamily protein	3.0E-34	−	12	39	51	8:00
HI9HAF203DC7VQ	−	−	−	−	−	12	38	50	8:00
isotig00856	AT3G30775	ERD5	Methylenetetrahydrofolate reductase family protein	2.0E-17	−	12	38	50	4:00
HI9HAF202CLKGK	AT1G17420	LOX3	lipoxygenase 3	9.0E-20	−	12	37	49	20:00
isotig03971	AT1G01470	LEA14	Late embryogenesis abundant protein	9.0E-40	−	12	37	49	20:00
SSH12-1-76_008_D10	−	−	−	−	−	12	36	48	24:00
isotig04281	AT3G22840	ELIP1	Chlorophyll A-B binding family protein	5.0E-34	−	12	36	48	8:00
HI9HAF203DOBMP	AT1G06410	ATTPS7	trehalose-phosphatase/synthase 7	8.0E-52	−	12	36	48	20:00
isotig00773	−	−	−	−	−	12	35	47	20:00
isotig00772	AT1G71980	−	Protease-associated (PA) RING/U-box zinc finger family protein	9.0E-88	−	12	35	47	20:00
HI9HAF203CYRO3	AT3G14440	NCED3	nine-cis-epoxycarotenoid dioxygenase 3	5.0E-48	−	12	34	46	4:00
HI9HAF202CF8CB	AT4G02780	GA1	Terpenoid cyclases/Protein prenyltransferases superfamily protein	6.0E-20	−	12	32	44	4:00
HI9HAF203C39DV	AT4G10770	ATOPT7	oligopeptide transporter 7	8.0E-50	−	12	32	44	4:00
HI9HAF203C89ZE	AT4G10770	ATOPT7	oligopeptide transporter 7	9.0E-51	−	12	32	44	4:00
isotig00189	−	−	−	−	−	12	31	43	20:00
HI9HAF202B503U	−	−	−	−	−	12	28	40	4:00
Shoot-020-55_013_G07	−	−	−	−	−	12	27	39	16:00
isotig05949	AT2G39540	−	Gibberellin-regulated family protein	4.0E-23	−	12	27	39	24:00
isotig00857	AT5G38710	−	Methylenetetrahydrofolate reductase family protein	5.0E-48	−	12	26	38	4:00
isotig06283	AT4G25810	XTR6	xyloglucan endotransglycosylase 6	1.0E-67	−	12	25	37	4:00
HI9HAF202BXWDV	AT1G74950	JAZ2	TIFY domain/Divergent CCT motif family protein	4.0E-16	−	12	24	36	8:00
Shoot-045-36	−	−	−	−	−	12	23	35	4:00
HI9HAF203CZGHG	AT2G39050	−	hydroxyproline-rich glycoprotein family protein	7.0E-41	−	12	23	35	20:00

isotig04626	AT1G22270	–	Trm112p-like protein	1.0E-41	–	12	22	34	12:00
Shoot-024-70	AT4G10490	–	2-oxoglutarate (2OG) and Fe(II)-dependent oxygenase superfamily protein	2.0E-80	–	12	22	34	8:00
isotig04821	AT5G54660	–	HSP20-like chaperones superfamily protein	2.0E-20	–	12	21	33	20:00
HI9HAF203DHEF4	–	–	–	–	–	12	19	31	24:00
HI9HAF203CY6MA	AT1G36160	ACC1	acetyl-CoA carboxylase 1	4.0E-51	–	12	13	25	8:00
isotig04004	AT3G51860	CAX3	cation exchanger 3	6.0E-65	–	11	48	59	8:00
Shoot-008-10_004_B02	AT5G08640	FLS	flavonol synthase 1	2.0E-40	–	11	42	53	16:00
isotig00986	AT3G62600	ATERDJ3B	DNAJ heat shock family protein	2.0E-29	–	11	38	49	12:00
isotig06420	–	–	–	–	–	11	37	48	20:00
HI9HAF202CM3YO	AT1G48600	PMEAMT	S-adenosyl-L-methionine-dependent methyltransferases superfamily protein	3.0E-43	–	11	36	47	12:00
isotig00930	–	–	–	–	–	11	35	46	16:00
isotig06573	AT2G30870	ATGSTF10	glutathione S-transferase PHI 10	6.0E-50	–	11	35	46	16:00
SSH24-3-37_009_E05	–	–	–	–	–	11	34	45	12:00
Shoot-057-82	AT3G52072	–	other RNA	3.0E-11	–	11	34	45	16:00
isotig06028	–	–	–	–	–	11	32	43	12:00
Shoot-026-11	–	–	–	–	–	11	31	42	4:00
Shoot-017-90_004_B12	–	–	–	–	–	11	31	42	20:00
HI9HAF203C7K1B	AT1G55020	LOX1	lipoxygenase 1	2.0E-41	–	11	31	42	4:00
HI9HAF203CYNP6	AT4G26590	ATOPT5	oligopeptide transporter 5	2.0E-43	–	11	31	42	4:00
isotig04997	AT4G27450	–	Aluminium induced protein with YGL and LRDR motifs	5.0E-59	–	11	30	41	20:00
isotig05128	AT1G69530	ATEXPA1	expansin A1	1.0E-103	–	11	30	41	12:00
HI9HAF202CCNWM	AT1G17870	ATEGY3	ethylene-dependent gravitropism-deficient and yellow-green-like 3	7.0E-44	–	11	29	40	12:00
HI9HAF202CJ583	AT2G06050	OPR3	oxophytodienoate-reductase 3	6.0E-36	–	11	27	38	12:00
HI9HAF202B43DT	AT5G13490	AAC2	ADP/ATP carrier 2	2.0E-70	–	11	27	38	4:00
Shoot-048-02	AT4G28670	–	Protein kinase family protein with domain of unknown function (DUF26)	2.0E-15	–	11	26	37	12:00
isotig01631	AT1G25560	TEM1	AP2/B3 transcription factor family protein	2.0E-77	–	11	26	37	8:00
HI9HAF203C0UYS	AT3G53260	PAL2	phenylalanine ammonia-lyase 2	5.0E-52	–	11	25	36	4:00
HI9HAF202BXWKV	AT3G62660	GATL7	galacturonosyltransferase-like 7	4.0E-58	–	11	24	35	4:00
HI9HAF203DDZYO	AT3G18110	EMB1270	Pentatricopeptide repeat (PPR) superfamily protein	3.0E-46	–	11	20	31	20:00
HI9HAF202BZIYP	AT3G18110	EMB1270	Pentatricopeptide repeat (PPR) superfamily protein	1.0E-50	–	11	20	31	20:00
HI9HAF202BST0A	AT4G17370	–	Oxidoreductase family protein	1.0E-46	–	11	17	28	20:00
HI9HAF202B10WK	AT1G01720	ATAF1	NAC (No Apical Meristem) domain transcriptional regulator superfamily protein	2.0E-64	–	11	16	27	4:00
Shoot-021-56_015_H07	AT4G17370	–	Oxidoreductase family protein	7.0E-87	–	11	15	26	20:00
HI9HAF203DBY4K	AT1G36160	ACC1	acetyl-CoA carboxylase 1	4.0E-53	–	11	14	25	8:00
HI9HAF202CB809	AT1G36160	ACC1	acetyl-CoA carboxylase 1	3.0E-71	–	11	13	24	8:00
HI9HAF202BVHL6	ATMG00510	NAD7	NADH dehydrogenase subunit 7	7.0E-81	–	11	12	23	20:00
isotig04287	ATCG01250	NDHB.2	NADH-Ubiquinone/plastoquinone (complex I) protein	4.0E-77	–	11	10	21	8:00
SSH12-5-60_008_D08	–	–	–	–	–	11	8	19	24:00
isotig03718	ATMG00160	COX2	cytochrome oxidase 2	2.0E-52	–	11	8	19	8:00
HI9HAF203DR40J	AT4G25100	FSD1	Fe superoxide dismutase 1	4.0E-57	–	10	46	56	24:00
isotig03966	AT3G19000	–	2-oxoglutarate (2OG) and Fe(II)-dependent oxygenase superfamily protein	6.0E-92	–	10	43	53	20:00
isotig03291	AT5G05340	–	Peroxidase superfamily protein	2.0E-100	–	10	40	50	16:00
Shoot-045-28	AT1G78990	–	HXXXD-type acyl-transferase family protein	2.0E-57	–	10	39	49	8:00
SSH24-8-75_006_C10	AT3G59730	–	Concanavalin A-like lectin protein kinase family protein	3.0E-14	–	10	38	48	4:00
HI9HAF203DCJY0	AT1G67110	CYP735A2	cytochrome P450, family 735, subfamily A, polypeptide 2	1.0E-11	–	10	37	47	20:00
isotig01145	–	–	–	–	–	10	36	46	4:00
SSH24-1-05_009_E01	–	–	–	–	–	10	36	46	12:00
HI9HAF202BW59L	AT5G42180	–	Peroxidase superfamily protein	2.0E-38	–	10	34	44	8:00
isotig03941	AT5G67360	ARA12	Subtilase family protein	5.0E-67	–	10	34	44	8:00
isotig01036	AT5G48930	HCT	hydroxycinnamoyl-CoA shikimate/quinate hydroxycinnamoyl transferase	1.0E-15	–	10	33	43	24:00
isotig06749	AT5G49920	–	Octicosapeptide/Phox/Bem1p family protein	1.0E-31	–	10	30	40	4:00
SSH24-4-60_008_D08	–	–	–	–	–	10	29	39	12:00
HI9HAF202B6ZLD	AT5G33370	–	GDSL-like Lipase/Acylhydrolase superfamily protein	8.0E-46	–	10	29	39	24:00
HI9HAF202B0P7R	AT1G73220	AtOCT1	organic cation/carnitine transporter1	8.0E-43	–	10	27	37	8:00
HI9HAF203DI81Y	AT1G59870	PEN3	ABC-2 and Plant PDR ABC-type transporter family protein	1.0E-60	–	10	27	37	4:00
HI9HAF203DO7V5	AT1G05260	RCI3	Peroxidase superfamily protein	1.0E-16	–	10	26	36	4:00
isotig04428	–	–	–	–	–	10	25	35	24:00
HI9HAF202BSJ5G	AT3G23890	TOPII	topoisomerase II	9.0E-62	–	10	25	35	12:00
isotig00146	–	–	–	–	–	10	24	34	16:00
HI9HAF202CHIHG	–	–	–	–	–	10	23	33	4:00
HI9HAF203C9D5H	AT4G16660	–	heat shock protein 70 (Hsp 70) family protein	5.0E-49	–	10	23	33	12:00
isotig06856	AT5G42500	–	Disease resistance-responsive (dirigent-like protein) family protein	1.0E-12	–	10	22	32	16:00
isotig05759	AT2G27830	–	unknown protein	6.0E-17	–	10	22	32	4:00
SSH12-6-75_006_C10	–	–	–	–	–	10	21	31	20:00
isotig05088	AT3G10300	–	Calcium-binding EF-hand family protein	8.0E-56	–	10	21	31	20:00
HI9HAF202B4EIJ	AT4G02780	GA1	Terpenoid cyclases/Protein prenyltransferases superfamily protein	4.0E-24	–	10	19	29	12:00
HI9HAF203DHGH8	AT1G64660	ATMGL	methionine gamma-lyase	8.0E-25	–	10	19	29	4:00
Shoot-059-75	AT4G10850	SWEET7	Nodulin MtN3 family protein	8.0E-44	–	10	18	28	24:00
isotig06256	AT2G28790	–	Pathogenesis-related thaumatin superfamily protein	2.0E-62	–	10	16	26	24:00
HI9HAF202BVWRG	AT1G36160	ACC1	acetyl-CoA carboxylase 1	2.0E-75	–	10	12	22	8:00
SSH12-4-82_003_B11	ATCG00350	PSAA	Photosystem I, PsaA/PsaB protein	2.0E-94	–	10	12	22	8:00
isotig06219	AT1G11530	ATCXXS1	C-terminal cysteine residue is changed to a serine 1	4.0E-32	–	9	45	54	16:00
isotig05866	–	–	–	–	–	9	42	51	20:00
Shoot-055-15	AT1G26560	BGLU40	beta glucosidase 40	1.0E-73	–	9	42	51	4:00
HI9HAF203C165B	AT1G59870	PEN3	ABC-2 and Plant PDR ABC-type transporter family protein	2.0E-44	–	9	40	49	24:00
HI9HAF202CJNDC	–	–	–	–	–	9	39	48	12:00
Shoot-056-57	AT2G16430	PAP10	purple acid phosphatase 10	9.0E-72	–	9	38	47	20:00
SSH24-8-03_005_C01	–	–	–	–	–	9	37	46	12:00
HI9HAF203DIBLL	AT3G50660	DWF4	Cytochrome P450 superfamily protein	7.0E-49	–	9	35	44	16:00
HI9HAF202B7Z7U	AT5G47500	–	Pectin lyase-like superfamily protein	3.0E-67	–	9	35	44	4:00
HI9HAF203C309P	AT1G71695	–	Peroxidase superfamily protein	5.0E-37	–	9	34	43	24:00
HI9HAF202BS0KJ	AT1G55020	LOX1	lipoxygenase 1	2.0E-18	–	9	33	42	4:00
isotig04335	AT2G48020	–	Major facilitator superfamily protein	1.0E-82	–	9	33	42	16:00
isotig04532	AT5G53190	SWEET3	Nodulin MtN3 family protein	8.0E-39	–	9	32	41	16:00
SSH24-4-29_010_E04	–	–	–	–	–	9	31	40	24:00
isotig05549	AT1G24020	MLP423	MLP-like protein 423	2.0E-24	–	9	31	40	24:00
isotig06886	–	–	–	–	–	9	28	37	16:00
SSH12-5-27_006_C04	–	–	–	–	–	9	28	37	20:00
SSH12-9-14_012_F02	–	–	–	–	–	9	27	36	20:00
isotig05919	AT4G19450	–	Major facilitator superfamily protein	3.0E-28	–	9	24	33	8:00
isotig06496	AT3G02260	BIG	auxin transport protein (BIG)	2.0E-57	–	9	24	33	12:00
Shoot-056-26	–	–	–	–	–	9	22	31	12:00
SSH12-5-43_006_C06	–	–	–	–	–	9	22	31	16:00
HI9HAF202B5WN1	AT1G78390	NCED9	nine-cis-epoxycarotenoid dioxygenase 9	2.0E-32	–	9	22	31	12:00
HI9HAF203DOM37	AT2G37420	–	ATP binding microtubule motor family protein	2.0E-55	–	9	21	30	24:00
HI9HAF202CI9UU	AT1G15210	PDR7	pleiotropic drug resistance 7	1.0E-59	–	9	21	30	4:00
Shoot-024-22	AT1G09560	GLP5	germin-like protein 5	1.0E-46	–	9	19	28	24:00
HI9HAF202BYZYI	AT4G02780	GA1	Terpenoid cyclases/Protein prenyltransferases superfamily protein	1.0E-32	–	9	16	25	12:00
HI9HAF202CBZ4D	AT4G02780	GA1	Terpenoid cyclases/Protein prenyltransferases superfamily protein	3.0E-11	–	9	15	24	12:00
HI9HAF203DI5CY	AT1G36160	ACC1	acetyl-CoA carboxylase 1	6.0E-41	–	9	14	23	8:00
HI9HAF203DCB1A	AT1G36160	ACC1	acetyl-CoA carboxylase 1	5.0E-76	–	9	13	22	8:00
HI9HAF202B9Y9X	AT5G06460	ATUBA2	ubiquitin activating enzyme 2	7.0E-64	–	8	49	57	4:00
isotig00299	–	–	–	–	–	8	46	54	12:00
isotig02675	AT2G45570	CYP76C2	cytochrome P450, family 76, subfamily C, polypeptide 2	3.0E-90	–	8	45	53	8:00
isotig06779	–	–	–	–	–	8	44	52	24:00

isotig02600	AT5G25460	−	Protein of unknown function, DUF642	3.0E−138	−	8	44	52	8:00
HI9HAF202B4QZB	AT4G23030	−	MATE efflux family protein	2.0E−51	−	8	43	51	20:00
HI9HAF203DGFUJ	AT1G64160	−	Disease resistance−responsive (dirigent−like protein) family protein	2.0E−45	−	8	40	48	4:00
HI9HAF203DMC7D	AT5G49720	ATGH9A1	glycosyl hydrolase 9A1	1.0E−49	−	8	39	47	4:00
SSH12−2−61_010_E08	−	−	−	−	−	8	38	46	12:00
isotig02283	AT4G39660	AGT2	alanine:glyoxylate aminotransferase 2	4.0E−157	−	8	38	46	24:00
HI9HAF202B7WFT	AT3G24220	NCED6	nine−cis−epoxycarotenoid dioxygenase 6	6.0E−11	−	8	35	43	4:00
isotig05646	AT1G65450	−	HXXXD−type acyl−transferase family protein	2.0E−21	−	8	35	43	20:00
HI9HAF203DPNWC	AT1G15210	PDR7	pleiotropic drug resistance 7	4.0E−42	−	8	33	41	12:00
HI9HAF203DMB3A	AT2G45560	CYP76C1	cytochrome P450, family 76, subfamily C, polypeptide 1	7.0E−43	−	8	33	41	12:00
Shoot−001−58_004_B08	AT4G17090	CT−BMY	chloroplast beta−amylase	6.0E−24	−	8	31	39	20:00
HI9HAF203DBA3V	AT2G31970	RAD50	DNA repair−recombination protein (RAD50)	2.0E−59	−	8	29	37	8:00
HI9HAF202BV9JR	AT4G10490	−	2−oxoglutarate (2OG) and Fe(II)−dependent oxygenase superfamily protein	4.0E−62	−	8	25	33	8:00
isotig06685	−	−	−	−	−	8	24	32	16:00
HI9HAF203C8J9K	AT4G10490	−	2−oxoglutarate (2OG) and Fe(II)−dependent oxygenase superfamily protein	4.0E−45	−	8	22	30	8:00
HI9HAF203DRGQG	AT1G55860	UPL1	ubiquitin−protein ligase 1	2.0E−47	−	8	21	29	8:00
isotig02500	−	−	−	−	−	8	20	28	24:00
Shoot−049−90	AT5G33406	−	hAT dimerisation domain−containing protein / transposase−related	9.0E−37	−	8	16	24	12:00
isotig03916	−	−	−	−	−	8	15	23	12:00
HI9HAF203DBDW0	ATMG00990	NAD3	NADH dehydrogenase 3	7.0E−47	−	8	11	19	20:00
HI9HAF202CJ9MU	AT3G21510	AHP1	histidine−containing phosphotransmitter 1	5.0E−26	−	7	47	54	12:00
HI9HAF202BXUX7	−	−	−	−	−	7	46	53	4:00
isotig00963	AT3G59030	TT12	MATE efflux family protein	3.0E−65	−	7	45	52	4:00
isotig00300	−	−	−	−	−	7	43	50	12:00
Shoot−001−37_009_E05	AT5G55020	ATMYB120	myb domain protein 120	6.0E−48	−	7	42	49	20:00
isotig00124	−	−	−	−	−	7	41	48	4:00
isotig00301	−	−	−	−	−	7	41	48	12:00
HI9HAF203C722I	AT1G71695	−	Peroxidase superfamily protein	3.0E−24	−	7	41	48	12:00
HI9HAF202B6MT8	AT4G17690	−	Peroxidase superfamily protein	5.0E−48	−	7	41	48	20:00
HI9HAF203C3CAU	AT4G02780	GA1	Terpenoid cyclases/Protein prenyltransferases superfamily protein	2.0E−27	−	7	40	47	4:00
HI9HAF203DRT8M	AT1G48100	−	Pectin lyase−like superfamily protein	9.0E−43	−	7	40	47	20:00
isotig02803	AT4G28940	−	Phosphorylase superfamily protein	3.0E−53	−	7	40	47	16:00
HI9HAF202CDFKH	−	−	−	−	−	7	39	46	16:00
Shoot−012−28_008_D04	AT1G19510	ATRL5	RAD−like 5	5.0E−13	−	7	39	46	24:00
SSH12−3−16_016_H02	AT2G15220	−	Plant basic secretory protein (BSP) family protein	1.0E−15	−	7	39	46	12:00
isotig04438	AT3G26510	−	Octicosapeptide/Phox/Bem1p family protein	1.0E−21	−	7	39	46	16:00
Shoot−003−67_005_C09	AT4G36850	−	PQ−loop repeat family protein / transmembrane family protein	6.0E−40	−	7	38	45	20:00
HI9HAF202CKBGD	AT5G24270	SOS3	Calcium−binding EF−hand family protein	7.0E−46	−	7	37	44	12:00
isotig00187	−	−	−	−	−	7	35	42	20:00
Shoot−020−39_013_G05	AT5G57530	XTH12	xyloglucan endotransglucosylase/hydrolase 12	1.0E−104	−	7	35	42	4:00
isotig06362	−	−	−	−	−	7	34	41	12:00
HI9HAF203DKRJV	AT1G71695	−	Peroxidase superfamily protein	5.0E−17	−	7	33	40	4:00
HI9HAF203CY2MA	AT1G73220	AtOCT1	organic cation/carnitine transporter1	2.0E−49	−	7	33	40	8:00
HI9HAF202CBUPS	AT5G53550	YSL3	YELLOW STRIPE like 3	3.0E−54	−	7	32	39	24:00
HI9HAF202CC8FP	AT2G29130	LAC2	laccase 2	5.0E−43	−	7	29	36	8:00
SSH24−3−61_010_E08	AT3G04290	ATLTL1	Li−tolerant lipase 1	5.0E−77	−	7	28	35	24:00
isotig06804	−	−	−	−	−	7	25	32	4:00
SSH24−6−10_004_B02	−	−	−	−	−	7	24	31	8:00
Shoot−030−40	−	−	−	−	−	7	24	31	16:00
HI9HAF203DP674	AT5G53460	GLT1	NADH−dependent glutamate synthase 1	2.0E−45	−	7	23	30	12:00
HI9HAF203DI4D2	AT5G48850	ATSDI1	Tetratricopeptide repeat (TPR)−like superfamily protein	3.0E−59	−	7	21	28	4:00
HI9HAF202B3TEN	AT1G55860	UPL1	ubiquitin−protein ligase 1	5.0E−41	−	7	20	27	8:00
HI9HAF202CCWMQ	AT4G17370	−	Oxidoreductase family protein	2.0E−68	−	7	19	26	20:00
isotig01504	AT4G33300	ADR1−L1	ADR1−like 1	3.0E−97	−	7	17	24	8:00
HI9HAF203DSPP9	ATMG00960	CCB203	Cytochrome C assembly protein	5.0E−58	−	7	15	22	8:00
HI9HAF202CBT07	AT5G09970	CYP78A7	cytochrome P450, family 78, subfamily A, polypeptide 7	1.0E−42	−	6	49	55	16:00
isotig04350	AT2G40610	ATEXPA8	expansin A8	4.0E−108	−	6	42	48	12:00
isotig00398	AT5G13870	EXGT−A4	xyloglucan endotransglucosylase/hydrolase 5	4.0E−133	−	6	42	48	8:00
HI9HAF202B4MHZ	−	−	−	−	−	6	41	47	8:00
isotig03234	AT4G29260	−	HAD superfamily, subfamily IIIB acid phosphatase	2.0E−57	−	6	41	47	4:00
isotig02211	AT1G23760	JP630	BURP domain−containing protein	2.0E−25	−	6	38	44	20:00
Shoot−029−01	AT4G24210	SLY1	F−box family protein	2.0E−21	−	6	35	41	24:00
isotig01948	AT2G36780	−	UDP−Glycosyltransferase superfamily protein	3.0E−68	−	6	33	39	20:00
Shoot−007−49_001_A07	AT2G36750	UGT73C1	UDP−glucosyl transferase 73C1	2.0E−18	−	6	31	37	20:00
HI9HAF203CX5R4	AT3G10340	PAL4	phenylalanine ammonia−lyase 4	1.0E−73	−	6	30	36	4:00
HI9HAF203DQGN7	−	−	−	−	−	6	29	35	8:00
HI9HAF203CYF7U	AT2G29130	LAC2	laccase 2	2.0E−45	−	6	29	35	8:00
HI9HAF203DEQJH	AT1G22400	UGT85A1	UDP−Glycosyltransferase superfamily protein	3.0E−19	−	6	28	34	12:00
HI9HAF202BRGJH	AT1G64660	ATMGL	methionine gamma−lyase	5.0E−23	−	6	28	34	4:00
HI9HAF202CBRZ6	AT5G42650	AOS	allene oxide synthase	2.0E−41	−	6	26	32	20:00
isotig05242	AT5G42650	AOS	allene oxide synthase	4.0E−51	−	6	26	32	20:00
HI9HAF202CGNU7	AT5G09970	CYP78A7	cytochrome P450, family 78, subfamily A, polypeptide 7	1.0E−50	−	6	25	31	24:00
isotig06038	−	−	−	−	−	6	23	29	24:00
HI9HAF202CHJ1M	AT5G67360	ARA12	Subtilase family protein	2.0E−56	−	6	22	28	24:00
isotig04225	AT2G39050	−	hydroxyproline−rich glycoprotein family protein	4.0E−45	−	6	20	26	20:00
Shoot−050−10	−	−	−	−	−	6	17	23	12:00
isotig06644	−	−	−	−	−	6	12	18	4:00
SSH12−4−51_005_C07	ATCG00340	PSAB	Photosystem I, PsaA/PsaB protein	2.0E−54	−	6	11	17	8:00
HI9HAF203DP6B2	AT1G51170	−	Protein kinase superfamily protein	1.0E−41	−	5	53	58	8:00
Shoot−009−64_016_H08	AT4G09720	ATRABG3A	RAB GTPase homolog G3A	4.0E−78	−	5	48	53	24:00
isotig05079	−	−	−	−	−	5	46	51	4:00
HI9HAF203DKAG0	AT1G75450	CKX5	cytokinin oxidase 5	3.0E−52	−	5	43	48	16:00
isotig01229	AT1G55020	LOX1	lipoxygenase 1	0.0	−	5	43	48	4:00
isotig00236	AT4G17500	ATERF−1	ethylene responsive element binding factor 1	9.0E−27	−	5	42	47	8:00
HI9HAF202CMHOZ	AT3G01420	ALPHA−DOX1	Peroxidase superfamily protein	7.0E−34	−	5	40	45	12:00
HI9HAF203DJGMW	AT4G04320	−	malonyl−CoA decarboxylase family protein	1.0E−68	−	5	37	42	20:00
HI9HAF203C85HC	AT1G09795	ATATP−PRT2	ATP phosphoribosyl transferase 2	7.0E−54	−	5	36	41	24:00
HI9HAF202BXZ7C	AT1G32450	NRT1.5	nitrate transporter 1.5	4.0E−54	−	5	36	41	4:00
Shoot−023−44	−	−	−	−	−	5	35	40	12:00
HI9HAF202CHFG2	AT1G02460	−	Pectin lyase−like superfamily protein	1.0E−44	−	5	35	40	16:00
isotig01056	AT4G03210	XTH9	xyloglucan endotransglucosylase/hydrolase 9	8.0E−50	−	5	35	40	24:00
HI9HAF202CBZ1Q	AT5G53550	YSL3	YELLOW STRIPE like 3	7.0E−63	−	5	34	39	24:00
isotig04684	AT5G19140	ATAILP1	Aluminium induced protein with YGL and LRDR motifs	3.0E−64	−	5	33	38	4:00
Shoot−048−45	−	−	−	−	−	5	32	37	24:00
HI9HAF203CYEAO	AT3G22400	LOX5	PLAT/LH2 domain−containing lipoxygenase family protein	2.0E−34	−	5	30	35	8:00
HI9HAF202B4YW1	AT2G26910	PDR4	pleiotropic drug resistance 4	2.0E−52	−	5	30	35	12:00
HI9HAF203DP628	AT1G32450	NRT1.5	nitrate transporter 1.5	2.0E−56	−	5	30	35	4:00
isotig04129	−	−	−	−	−	5	27	32	16:00
HI9HAF203DPJ4T	AT2G29130	LAC2	laccase 2	2.0E−48	−	5	27	32	20:00
HI9HAF203DJJNC	AT3G11964	−	RNA binding;RNA binding	9.0E−44	−	5	26	31	16:00
isotig02138	ATCG00350	PSAA	Photosystem I, PsaA/PsaB protein	0.0	−	5	18	23	24:00
HI9HAF203C1YUV	AT4G26270	PFK3	phosphofructokinase 3	3.0E−59	−	4	56	60	20:00
HI9HAF202CFLCZ	AT1G26770	ATEXPA10	expansin A10	7.0E−41	−	4	50	54	8:00
SSH24−7−92_008_D12	AT1G08080	ATACA7	alpha carbonic anhydrase 7	3.0E−20	−	4	46	50	20:00
HI9HAF203DGCQK	−	−	−	−	−	4	44	48	16:00

isotig03642	AT2G23810	TET8	tetraspanin8	8.0E-82	–	4	42	46	16:00
HI9HAF202CEATR	AT5G53550	YSL3	YELLOW STRIPE like 3	9.0E-51	–	4	40	44	24:00
HI9HAF203DNML3	AT5G60020	LAC17	laccase 17	3.0E-75	–	4	39	43	12:00
Shoot-024-67	AT3G54420	ATEP3	homolog of carrot EP3-3 chitinase	7.0E-50	–	4	38	42	16:00
HI9HAF202B1PZL	AT5G41610	ATCHX18	cation/H+ exchanger 18	1.0E-47	–	4	36	40	4:00
HI9HAF202CELK4	AT1G09795	ATATP-PRT2	ATP phosphoribosyl transferase 2	3.0E-57	–	4	36	40	24:00
isotig01160	–	–	–	–	–	4	35	39	4:00
Shoot-020-28_008_D04	–	–	–	–	–	4	34	38	16:00
HI9HAF202B0S1U	AT5G05600	–	2-oxoglutarate (2OG) and Fe(II)-dependent oxygenase superfamily protein	3.0E-67	–	4	34	38	4:00
isotig02825	AT2G36870	XTH32	xyloglucan endotransglucosylase/hydrolase 32	2.0E-82	–	4	34	38	12:00
HI9HAF203C28J0	ATMG01320	NAD2B	NADH dehydrogenase 2B	3.0E-62	–	4	33	37	8:00
SSH24-5-51_005_C07	–	–	–	–	–	4	32	36	4:00
SSH12-5-14_012_F02	–	–	–	–	–	4	31	35	24:00
HI9HAF203DLL9G	AT5G24790	–	Protein of unknown function, DUF599	9.0E-42	–	4	31	35	12:00
isotig04171	–	–	–	–	–	4	27	31	8:00
HI9HAF202BVHVL	AT1G77330	–	2-oxoglutarate (2OG) and Fe(II)-dependent oxygenase superfamily protein	3.0E-36	–	4	19	23	4:00
HI9HAF203C92FX	AT1G75030	ATLP-3	thaumatin-like protein 3	9.0E-59	–	3	45	48	4:00
Shoot-020-36_007_D05	AT1G19510	ATRL5	RAD-like 5	9.0E-21	–	3	42	45	24:00
HI9HAF203C55R5	AT5G65690	PCK2	phosphoenolpyruvate carboxykinase 2	2.0E-38	–	3	40	43	20:00
isotig00995	–	–	–	–	–	3	38	41	12:00
isotig00631	AT5G41040	–	HXXXD-type acyl-transferase family protein	1.0E-51	–	3	38	41	12:00
isotig03027	AT1G23740	–	Oxidoreductase, zinc-binding dehydrogenase family protein	1.0E-71	–	3	35	38	16:00
isotig05661	ATCG00180	RPOC1	DNA-directed RNA polymerase family protein	2.0E-30	–	3	34	37	4:00
isotig00855	AT3G54420	ATEP3	homolog of carrot EP3-3 chitinase	5.0E-39	–	3	34	37	20:00
Shoot-027-33	AT5G56120	–	unknown protein	2.0E-48	–	3	33	36	12:00
HI9HAF202CCVOH	AT1G09795	ATATP-PRT2	ATP phosphoribosyl transferase 2	5.0E-52	–	3	32	35	24:00
HI9HAF202CKKSZ	AT1G73600	–	S-adenosyl-L-methionine-dependent methyltransferases superfamily protein	4.0E-65	–	3	30	33	12:00
HI9HAF202B2IMS	–	–	–	–	–	3	26	29	12:00
SSH12-4-88_015_H11	ATCG00210	YCF6	electron transporter, transferring electrons within cytochrome b6/f complex of photosystem IIs	4.0E-11	–	3	26	29	4:00
HI9HAF202CAVMM	AT2G38940	ATPT2	phosphate transporter 1;4	6.0E-60	–	3	26	29	16:00
HI9HAF202CBDWW	AT5G09970	CYP78A7	cytochrome P450, family 78, subfamily A, polypeptide 7	8.0E-45	–	3	25	28	24:00
HI9HAF203C9M7Y	AT3G54700	PHT1;7	phosphate transporter 1;7	4.0E-58	–	3	25	28	16:00
SSH24-1-33_001_A05	–	–	–	–	–	3	23	26	20:00
HI9HAF203DI3FD	ATMG00730	COX3	cytochrome c oxidase subunit 3	2.0E-51	–	3	18	21	20:00
HI9HAF203DAOB4	AT5G58960	GIL1	Plant protein of unknown function (DUF641)	4.0E-44	–	2	51	53	24:00
Shoot-020-18_003_B03	AT2G41480	–	Peroxidase superfamily protein	4.0E-85	–	2	47	49	20:00
SSH24-3-74_004_B10	–	–	–	–	–	2	44	46	24:00
isotig06404	AT4G20990	ATACA4	alpha carbonic anhydrase 4	1.0E-37	–	2	39	41	16:00
HI9HAF203C31QT	ATCG01090	NDHI	NADPH dehydrogenases	2.0E-73	–	2	36	38	24:00
isotig05358	AT5G54160	ATOMT1	O-methyltransferase 1	6.0E-38	–	2	34	36	4:00
HI9HAF203DFEH4	–	–	–	–	–	2	33	35	20:00
isotig05869	–	–	–	–	–	2	33	35	20:00
HI9HAF202CKM2C	AT1G09795	ATATP-PRT2	ATP phosphoribosyl transferase 2	5.0E-64	–	2	33	35	24:00
HI9HAF203C15TT	AT5G09970	CYP78A7	cytochrome P450, family 78, subfamily A, polypeptide 7	5.0E-45	–	2	27	29	24:00
HI9HAF203DI6YM	AT5G60020	LAC17	laccase 17	3.0E-48	–	2	24	26	20:00
isotig02148	AT3G02040	SRG3	senescence-related gene 3	6.0E-97	–	1	56	57	16:00
isotig03358	AT5G64260	EXL2	EXORDIUM like 2	3.0E-63	–	1	53	54	12:00
HI9HAF203DE4FE	AT4G10490	–	2-oxoglutarate (2OG) and Fe(II)-dependent oxygenase superfamily protein	4.0E-52	–	1	49	50	16:00
HI9HAF202CJGWB	AT5G07990	TT7	Cytochrome P450 superfamily protein	1.0E-43	–	1	45	46	8:00
HI9HAF202CIYAG	AT2G38940	ATPT2	phosphate transporter 1;4	2.0E-59	–	1	43	44	12:00
isotig01073	–	–	–	–	–	1	41	42	20:00
Shoot-017-95_014_G12	–	–	–	–	–	1	33	34	16:00
HI9HAF203DCI8H	AT4G02780	GA1	Terpenoid cyclases/Protein prenyltransferases superfamily protein	2.0E-13	–	1	32	33	12:00
isotig00994	–	–	–	–	–	1	31	32	8:00
HI9HAF202B77RI	AT1G32100	ATPRR1	pinoresinol reductase 1	2.0E-43	–	1	29	30	12:00
isotig03975	ATMG01360	COX1	cytochrome oxidase	4.0E-158	–	0	53	53	20:00
isotig01093	AT4G25780	–	CAP (Cysteine-rich secretory proteins, Antigen 5, and Pathogenesis-related 1 protein) superfamily protein	3.0E-31	–	0	51	51	8:00
HI9HAF202CHA2P	AT2G32830	PHT5	phosphate transporter 1;5	1.0E-58	–	0	32	32	16:00
isotig03329	–	–	–	–	–	0	31	31	20:00

[1] The putative function of the sequences was predicted according to the highest BLASTX hits with an e-value cutoff of e-10.

[2] Putative clock gene with node colored red in the estimated gene network (Figure 4).

[3] Number of children, parents and all edges were estimated by the SiGN-BN Bayesian network estimation program (http://sign.hgc.jp/signbn/index.html)[76].

[4] The time when the target reached maximum expression.
