# Supplementary material for: Clock genes and diurnal transcriptome dynamics in summer and winter in the gymnosperm Japanese cedar (Cryptomeria japonica (L.f.) D.Don)
Source: BMC Plant Biol. 2014 Nov 18;14:308. doi: 10.1186/s12870-014-0308-1 (PMC4245765; doi:10.1186/s12870-014-0308-1)
Supplement: Additional file 8: — The top 100 targets differentially expressed in summer and winter. 1The putative function of the sequences was predicted according to the highest BLASTX hits with an e-value cutoff of e-10. [file 12870_2014_308_MOESM8_ESM.pdf]

Additional file 8. **The top 100 targets differentally expressed in summer and winter.**

	SEQ_ID	BLASTX[1]				fold change
		Accession	Symbols	Description	e-value	summer/winter
1	isotig03363	AT2G23810	TET8	tetraspanin8	0.0	339.1
2	isotig06850	–	–	–	–	332.4
3	SSH24-4-81_001_A11	–	–	–	–	281.2
4	isotig00717	–	–	–	–	278.1
5	SSH12-6-80_016_H10	AT5G51950	–	Glucose-methanol-choline (GMC) oxidoreductase family protein	1.0E-42	271.9
6	isotig02640	AT2G40610	ATEXPA8	expansin A8	0.0	223.8
7	HI9HAF203DGQMH	AT1G05260	RCI3	Peroxidase superfamily protein	2.9E-44	212.6
8	isotig01684	AT1G12570	–	Glucose-methanol-choline (GMC) oxidoreductase family protein	0.0	179.2
9	HI9HAF203DI6YM	AT5G60020	LAC17	laccase 17	0.0	174.6
10	Shoot-056-45	–	–	–	–	156.4
11	isotig00716	–	–	–	–	151.6
12	isotig04524	AT4G01130	–	GDSL-like Lipase/Acylhydrolase superfamily protein	1.4E-45	144.9
13	SSH24-4-13_010_E02	AT1G70710	ATGH9B1	glycosyl hydrolase 9B1	0.0	141.2
14	HI9HAF202B64VM	AT1G05260	RCI3	Peroxidase superfamily protein	0.0	133.9
15	isotig02363	AT3G04290	ATLTL1	Li-tolerant lipase 1	0.0	132.3
16	Shoot-046-56	AT1G22900	–	Disease resistance-responsive (dirigent-like protein) family protein	2.0E-17	131.8
17	Shoot-055-32	AT3G08490	–		1.0E-12	127.8
18	isotig01990	AT5G33370	–	GDSL-like Lipase/Acylhydrolase superfamily protein	0.0	127.2
19	isotig05029	AT3G27200	–	Cupredoxin superfamily protein	9.0E-18	127.0
20	Shoot-005-37_009_E05	AT3G62390	TBL6	TRICHOME BIREFRINGENCE-LIKE 6	3.0E-41	126.1
21	HI9HAF203DRJW9	AT2G29130	LAC2	laccase 2	0.0	123.6
22	isotig04046	AT3G27200	–	Cupredoxin superfamily protein	3.0E-17	118.9
23	isotig00760	–	–	–	–	114.2
24	HI9HAF202CLGCV	AT5G05390	LAC12	laccase 12	0.0	113.8
25	isotig00447	–	–	–	–	113.4
26	isotig00396	AT4G28250	ATEXPB3	expansin B3	0.0	112.0
27	isotig03421	AT5G65730	XTH6	xyloglucan endotransglucosylase/hydrolase 6	0.0	104.6
28	isotig04871	–	–	–	–	102.7
29	isotig00761	–	–	–	–	100.4
30	isotig04918	–	–	–	–	100.0
31	isotig05917	AT5G03260	LAC11	laccase 11	0.0	98.6
32	isotig00290	AT5G03170	FLA11	FASCICLIN-like arabinogalactan-protein 11	2.0E-38	96.0
33	SSH12-8-58_004_B08	–	–	–	–	95.4
34	isotig01411	AT5G05390	LAC12	laccase 12	0.0	94.7
35	Shoot-024-22	AT1G09560	GLP5	germin-like protein 5	0.0	94.6
36	isotig06754	AT3G08490	–		4.0E-25	94.4
37	isotig00143	AT2G40610	ATEXPA8	expansin A8	0.0	93.1
38	isotig05266	–	–	–	–	88.9
39	isotig01788	AT1G70710	ATGH9B1	glycosyl hydrolase 9B1	0.0	86.4
40	isotig01494	–	–	–	–	86.0
41	isotig01957	AT1G72970	HTH	Glucose-methanol-choline (GMC) oxidoreductase family protein	0.0	85.2
42	isotig01405	AT1G70370	PG2	polygalacturonase 2	0.0	84.0
43	isotig03329	–	–	–	–	83.4
44	isotig03348	AT4G37800	XTH7	xyloglucan endotransglucosylase/hydrolase 7	0.0	83.3
45	isotig01952	AT1G01120	KCS1	3-ketoacyl-CoA synthase 1	0.0	81.6
46	isotig05249	–	–	–	–	76.6
47	isotig01520	AT5G44030	CESA4	cellulose synthase A4	0.0	73.1
48	isotig01266	AT5G44030	CESA4	cellulose synthase A4	0.0	71.6
49	isotig04079	AT1G11820	–	O-Glycosyl hydrolases family 17 protein	2.0E-26	71.2
50	isotig02152	AT3G62390	TBL6	TRICHOME BIREFRINGENCE-LIKE 6	0.0	70.9
51	isotig02600	AT5G25460	–	Protein of unknown function, DUF642	0.0	68.0
52	isotig00512	AT1G14430	–	glyoxal oxidase-related protein	0.0	67.4
53	HI9HAF203DR3KN	AT5G42180	–	Peroxidase superfamily protein	4.0E-25	66.9
54	isotig05282	AT1G09560	GLP5	germin-like protein 5	6.0E-41	65.4
55	isotig04044	AT5G60700	–	glycosyltransferase family protein 2	9.0E-39	65.3
56	isotig01729	AT5G60700	–	glycosyltransferase family protein 2	0.0	64.0
57	HI9HAF203DC4E4	AT5G60020	LAC17	laccase 17	0.0	63.2
58	isotig06376	AT2G38080	IRX12	Laccase/Diphenol oxidase family protein	9.8E-45	63.0
59	isotig00165	AT5G63180	–	Pectin lyase-like superfamily protein	0.0	61.4
60	isotig06728	–	–	–	–	60.2
61	SSH24-5-74_004_B10	AT3G02885	GASA5	GAST1 protein homolog 5	9.0E-37	59.9
62	HI9HAF203DKXQU	AT5G19730	–	Pectin lyase-like superfamily protein	0.0	59.5
63	Shoot-006-44_008_D06	AT4G37800	XTH7	xyloglucan endotransglucosylase/hydrolase 7	0.0	58.5
64	isotig04583	AT1G60060	–	Serine/threonine-protein kinase WNK (With No Lysine)-related	2.0E-36	57.8
65	SSH12-7-52_007_D07	–	–	–	–	56.4
66	isotig05536	AT5G60020	LAC17	laccase 17	0.0	56.3
67	isotig03268	AT1G69530	ATEXPA1	expansin A1	0.0	55.7
68	isotig04812	AT5G09810	ACT7	actin 7	0.0	55.4
69	Shoot-049-62	–	–	–	–	54.8
70	isotig02066	AT5G36110	CYP716A1	cytochrome P450, family 716, subfamily A, polypeptide 1	0.0	54.5
71	isotig00258	AT4G13710	–	Pectin lyase-like superfamily protein	0.0	53.4
72	isotig01747	AT2G31820	–	Ankyrin repeat family protein	2.0E-32	53.2
73	isotig05208	–	–	–	–	53.2
74	isotig06473	AT2G06925	ATSPLA2-ALPHA	Phospholipase A2 family protein	2.0E-25	52.0
75	isotig06409	AT5G42500	–	Disease resistance-responsive (dirigent-like protein) family protein	1.0E-19	51.9
76	isotig00439	–	–	–	–	51.5
77	Shoot-006-81_001_A11	AT2G40610	ATEXPA8	expansin A8	0.0	51.3
78	Shoot-024-10	AT3G10185	–	Gibberellin-regulated family protein	2.0E-21	50.2
79	SSH12-5-21_009_E03	–	–	–	–	48.8
80	isotig00618	AT3G56630	CYP94D2	cytochrome P450, family 94, subfamily D, polypeptide 2	0.0	47.9
81	isotig03987	AT4G29260	–	HAD superfamily, subfamily IIIB acid phosphatase	0.0	47.8
82	isotig00257	AT4G24780	–	Pectin lyase-like superfamily protein	0.0	47.7
83	HI9HAF202CAVXN	AT5G19730	–	Pectin lyase-like superfamily protein	0.0	47.6
84	isotig01575	AT4G19170	NCED4	nine-cis-epoxycarotenoid dioxygenase 4	0.0	47.1
85	isotig00166	AT4G24780	–	Pectin lyase-like superfamily protein	0.0	46.8
86	isotig00513	AT1G14430	–	glyoxal oxidase-related protein	0.0	46.8
87	HI9HAF203DKID8	AT2G29130	LAC2	laccase 2	0.0	46.4
88	HI9HAF202B740S	–	–	–	–	46.1
89	HI9HAF203CZOG1	AT1G05260	RCI3	Peroxidase superfamily protein	0.0	46.0
90	HI9HAF202BYP6N	AT5G48900	–	Pectin lyase-like superfamily protein	0.0	45.7
91	isotig03234	AT4G29260	–	HAD superfamily, subfamily IIIB acid phosphatase	0.0	45.5
92	isotig02208	AT3G04290	ATLTL1	Li-tolerant lipase 1	0.0	45.4
93	HI9HAF202CE2V5	AT1G63440	HMA5	heavy metal atpase 5	0.0	45.2
94	isotig02580	AT5G25900	GA3	GA requiring 3	0.0	45.1
95	isotig04482	AT5G63180	–	Pectin lyase-like superfamily protein	0.0	44.5
96	isotig06468	AT1G02800	ATCEL2	cellulase 2	0.0	44.2
97	HI9HAF203DEITS	AT2G05920	–	Subtilase family protein	0.0	44.2
98	HI9HAF202CGXR6	AT5G60020	LAC17	laccase 17	0.0	44.0
99	isotig03660	AT5G03170	FLA11	FASCICLIN-like arabinogalactan-protein 11	1.0E-27	43.5
100	isotig03700	AT2G26640	KCS11	3-ketoacyl-CoA synthase 11	0.0	43.4

	SEQ_ID	BLASTX[1]				fold change winter/summer
		Accession	Symbols	Description	e-value	
1	isotig06070	–	–	–	–	1086.9
2	isotig03779	AT5G25610	RD22	BURP domain-containing protein	2.0E-38	949.0
3	isotig06296	–	–	–	–	904.1
4	isotig06231	–	–	–	–	608.0
5	isotig00118	AT3G22840	ELIP1	Chlorophyll A-B binding family protein	4.0E-39	328.0
6	isotig05105	–	–	–	–	259.3
7	HI9HAF203C6495	AT1G08550	NPQ1	non-photochemical quenching 1	5.0E-30	197.7
8	isotig05776	AT1G75750	GASA1	GAST1 protein homolog 1	5.0E-21	109.8
9	isotig05650	–	–	–	–	96.4
10	isotig05800	–	–	–	–	95.1
11	isotig01089	–	–	–	–	73.4
12	Shoot-020-55_013_G07	–	–	–	–	72.3
13	isotig03659	AT1G03220	–	Eukaryotic aspartyl protease family protein	0.0	70.1
14	Shoot-005-09_002_A02	AT4G21690	ATGA3OX3	gibberellin 3-oxidase 3	8.0E-14	69.3
15	isotig00964	–	–	–	–	66.4
16	Shoot-047-72	–	–	–	–	64.9
17	isotig05015	AT1G71050	HIPP20	Heavy metal transport/detoxification superfamily protein	3.0E-38	64.2
18	isotig02783	AT2G26150	ATHSFA2	heat shock transcription factor A2	0.0	57.8
19	isotig04131	AT4G11650	ATOSM34	osmotin 34	0.0	57.7
20	isotig00117	AT3G22840	ELIP1	Chlorophyll A-B binding family protein	4.0E-40	45.3
21	HI9HAF202CL6GJ	AT2G38290	ATAMT2	ammonium transporter 2	2.8E-45	40.2
22	HI9HAF202B1CBE	AT1G67110	CYP735A2	cytochrome P450, family 735, subfamily A, polypeptide 2	9.0E-23	40.0
23	isotig03635	AT4G11650	ATOSM34	osmotin 34	0.0	38.8
24	isotig04730	AT3G26510	–	Octicosapeptide/Phox/Bem1p family protein	4.0E-27	38.5
25	isotig00121	AT5G49690	–	UDP-Glycosyltransferase superfamily protein	3.0E-24	37.2
26	HI9HAF202B99QE	AT5G13930	CHS	Chalcone and stilbene synthase family protein	7.0E-45	35.9
27	HI9HAF203DJ63O	AT2G38290	ATAMT2	ammonium transporter 2	0.0	35.6
28	Shoot-023-44	–	–	–	–	34.8
29	HI9HAF203C8JBI	AT4G10490	–	2-oxoglutarate (2OG) and Fe(II)-dependent oxygenase superfamily protein	1.4E-45	34.6
30	isotig06607	AT3G22840	ELIP1	Chlorophyll A-B binding family protein	2.0E-19	34.0
31	isotig04319	AT5G24090	ATCHIA	chitinase A	0.0	33.6
32	HI9HAF203DMC7D	AT5G49720	ATGH9A1	glycosyl hydrolase 9A1	0.0	30.7
33	isotig04737	AT2G39050	–	hydroxyproline-rich glycoprotein family protein	0.0	30.3
34	isotig00965	–	–	–	–	29.7
35	isotig05160	AT2G43620	–	Chitinase family protein	0.0	29.5
36	isotig00896	AT2G39050	–	hydroxyproline-rich glycoprotein family protein	0.0	29.3
37	isotig03354	AT4G16260	–	Glycosyl hydrolase superfamily protein	0.0	29.2
38	isotig03688	–	–	–	–	28.6
39	HI9HAF203C95RK	AT1G64160	–	Disease resistance-responsive (dirigent-like protein) family protein	9.0E-41	28.1
40	HI9HAF203C93I1	AT1G64160	–	Disease resistance-responsive (dirigent-like protein) family protein	9.0E-41	28.1
41	isotig00122	AT5G49690	–	UDP-Glycosyltransferase superfamily protein	4.0E-26	27.3
42	isotig06156	–	–	–	–	25.8
43	Shoot-007-34_003_B05	AT5G13930	CHS	Chalcone and stilbene synthase family protein	0.0	23.4
44	isotig06868	–	–	–	–	23.3
45	isotig03642	AT2G23810	TET8	tetraspanin8	0.0	22.0
46	isotig05231	AT3G22840	ELIP1	Chlorophyll A-B binding family protein	8.0E-37	21.7
47	HI9HAF202CKOVZ	AT1G11260	STP1	sugar transporter 1	0.0	20.6
48	isotig05509	AT4G11650	ATOSM34	osmotin 34	0.0	20.3
49	isotig00231	–	–	–	–	20.0
50	isotig00878	AT1G48130	ATPER1	1-cysteine peroxiredoxin 1	0.0	20.0
51	isotig05473	AT5G18810	SCL28	SC35-like splicing factor 28	1.0E-27	19.9
52	HI9HAF203DJWSN	AT5G29560	–	caleosin-related family protein	8.0E-41	19.2
53	isotig04349	AT3G12500	ATHCHIB	basic chitinase	0.0	19.0
54	isotig00711	AT5G25610	RD22	BURP domain-containing protein	9.0E-32	17.9
55	HI9HAF202BYAVK	AT2G29380	HAI3	highly ABA-induced PP2C gene 3	6.0E-17	17.0
56	HI9HAF202BX0CC	AT1G64890	–	Major facilitator superfamily protein	5.0E-44	16.2
57	HI9HAF202B3961	AT1G11260	STP1	sugar transporter 1	0.0	16.1
58	HI9HAF203DIIVI	AT3G15510	ATNAC2	NAC domain containing protein 2	0.0	15.7
59	HI9HAF202B10WK	AT1G01720	ATAF1	NAC (No Apical Meristem) domain transcriptional regulator superfamily protein	0.0	15.6
60	HI9HAF203C5D0I	AT5G54800	GPT1	glucose 6-phosphate/phosphate translocator 1	0.0	15.4
61	isotig06361	AT4G17490	ATERF6	ethylene responsive element binding factor 6	7.0E-15	14.9
62	isotig03971	AT1G01470	LEA14	Late embryogenesis abundant protein	9.0E-40	14.9
63	isotig05436	AT3G22840	ELIP1	Chlorophyll A-B binding family protein	1.0E-35	14.9
64	HI9HAF202B1YSW	AT3G10340	PAL4	phenylalanine ammonia-lyase 4	0.0	14.7
65	isotig04743	AT3G22840	ELIP1	Chlorophyll A-B binding family protein	2.0E-37	14.6
66	isotig00661	–	–	–	–	14.4
67	HI9HAF203CXZG6	AT3G10340	PAL4	phenylalanine ammonia-lyase 4	0.0	14.2
68	SSH12-7-75_006_C10	–	–	–	–	14.1
69	HI9HAF203C8V7S	AT2G23890	–	HAD-superfamily hydrolase, subfamily IG, 5'-nucleotidase	5.6E-45	14.0
70	isotig05965	AT5G25280	–	serine-rich protein-related	1.0E-15	13.8
71	isotig03495	AT3G22840	ELIP1	Chlorophyll A-B binding family protein	3.0E-40	13.6
72	HI9HAF202CH5NT	AT5G54800	GPT1	glucose 6-phosphate/phosphate translocator 1	0.0	13.6
73	HI9HAF202BX9NX	AT1G64890	–	Major facilitator superfamily protein	7.0E-44	13.6
74	HI9HAF203C1W1J	AT3G11410	ATPP2CA	protein phosphatase 2CA	1.0E-34	13.2
75	isotig03902	AT5G64170	–	dentin sialophosphoprotein-related	5.0E-11	13.2
76	HI9HAF202CF8CB	AT4G02780	GA1	Terpenoid cyclases/Protein prenyltransferases superfamily protein	6.0E-20	12.9
77	isotig04794	–	–	–	–	12.9
78	HI9HAF202CHIHG	–	–	–	–	12.7
79	Shoot-054-22	–	–	–	–	12.6
80	HI9HAF202B43DT	AT5G13490	AAC2	ADP/ATP carrier 2	0.0	12.6
81	isotig01063	–	–	–	–	12.5
82	isotig06886	–	–	–	–	12.5
83	isotig05183	–	–	–	–	12.4
84	HI9HAF202CGVA8	AT1G67090	RBCS1A	ribulose bisphosphate carboxylase small chain 1A	3.0E-24	12.4
85	HI9HAF203DNZO6	AT4G02780	GA1	Terpenoid cyclases/Protein prenyltransferases superfamily protein	6.0E-20	12.4
86	isotig05358	AT5G54160	ATOMT1	O-methyltransferase 1	6.0E-38	12.2
87	HI9HAF202BTYDK	AT2G38310	PYL4	PYR1-like 4	0.0	12.2
88	SSH24-7-44_008_D06	AT4G14622	CPuORF60	conserved peptide upstream open reading frame 60	4.0E-32	12.1
89	HI9HAF203DPTED	AT2G23890	–	HAD-superfamily hydrolase, subfamily IG, 5'-nucleotidase	0.0	11.8
90	HI9HAF202B5JHF	AT3G53260	PAL2	phenylalanine ammonia-lyase 2	0.0	11.7
91	Shoot-055-81	AT1G71692	AGL12	AGAMOUS-like 12	8.0E-23	11.7
92	isotig04474	AT4G14690	ELIP2	Chlorophyll A-B binding family protein	4.0E-42	11.7
93	HI9HAF202CFXZK	AT3G12500	ATHCHIB	basic chitinase	0.0	11.6
94	HI9HAF202CJGWB	AT5G07990	TT7	Cytochrome P450 superfamily protein	1.0E-43	11.6
95	isotig03334	AT4G25700	BETA-OHASE 1	beta-hydroxylase 1	0.0	11.3
96	Shoot-048-27	AT5G24790	–	Protein of unknown function, DUF599	1.0E-13	11.3
97	isotig06420	–	–	–	–	11.2
98	Shoot-054-24	–	–	–	–	11.2
99	HI9HAF202B5N4V	–	–	–	–	11.1
100	isotig05923	–	–	–	–	11.0

[1] The putative function of the sequences was predicted according to the highest BLASTX hits with an e-value cutoff of e-10.
