# Supplementary material for: Clock genes and diurnal transcriptome dynamics in summer and winter in the gymnosperm Japanese cedar (Cryptomeria japonica (L.f.) D.Don)
Source: BMC Plant Biol. 2014 Nov 18;14:308. doi: 10.1186/s12870-014-0308-1 (PMC4245765; doi:10.1186/s12870-014-0308-1)
Supplement: Additional file 9: — Amino acid sequence alignment of LHY and CCA1 (A), TOC1 (B), GI (C), and ZTL, LKP2 and FKF1 (D). The species names are abbreviated as follows: At, Arabidopsis thaliana; Cj, Cryptomeria japonica; Pp, Physcomitrella patens subsp. patens; Sm, Selaginella moellendorffii. (A) NCBI accession numbers of the proteins are AtLHY (NP_001030924) and AtCCA1 (NP_850460). PpCCA1a and PpCCA1b are from Okada et al. [41]. The amino acid sequences of the domains (underlined) are from Wang et al. [40]. (B) NCBI accession numbers of the proteins are NP_200946 (AtTOC1) and XP_002963903 (SmTOC1). The amino acid sequences of the domains (underlined) are from Strayer et al. [42]. (C) NCBI accession numbers of the proteins are NP_564180 (AtGI) and XP_002961231 (SmGI). (D) NCBI accession numbers of the proteins are NP_001154783 (AtZTL), AEC06826 (AtLKP2), AAF32298 (FKF1) and XP_002990856 (SmFKF1-2). The amino acid sequences of the domains (underlined) are from Somers et al. [43]. [file 12870_2014_308_MOESM9_ESM.pdf]

A

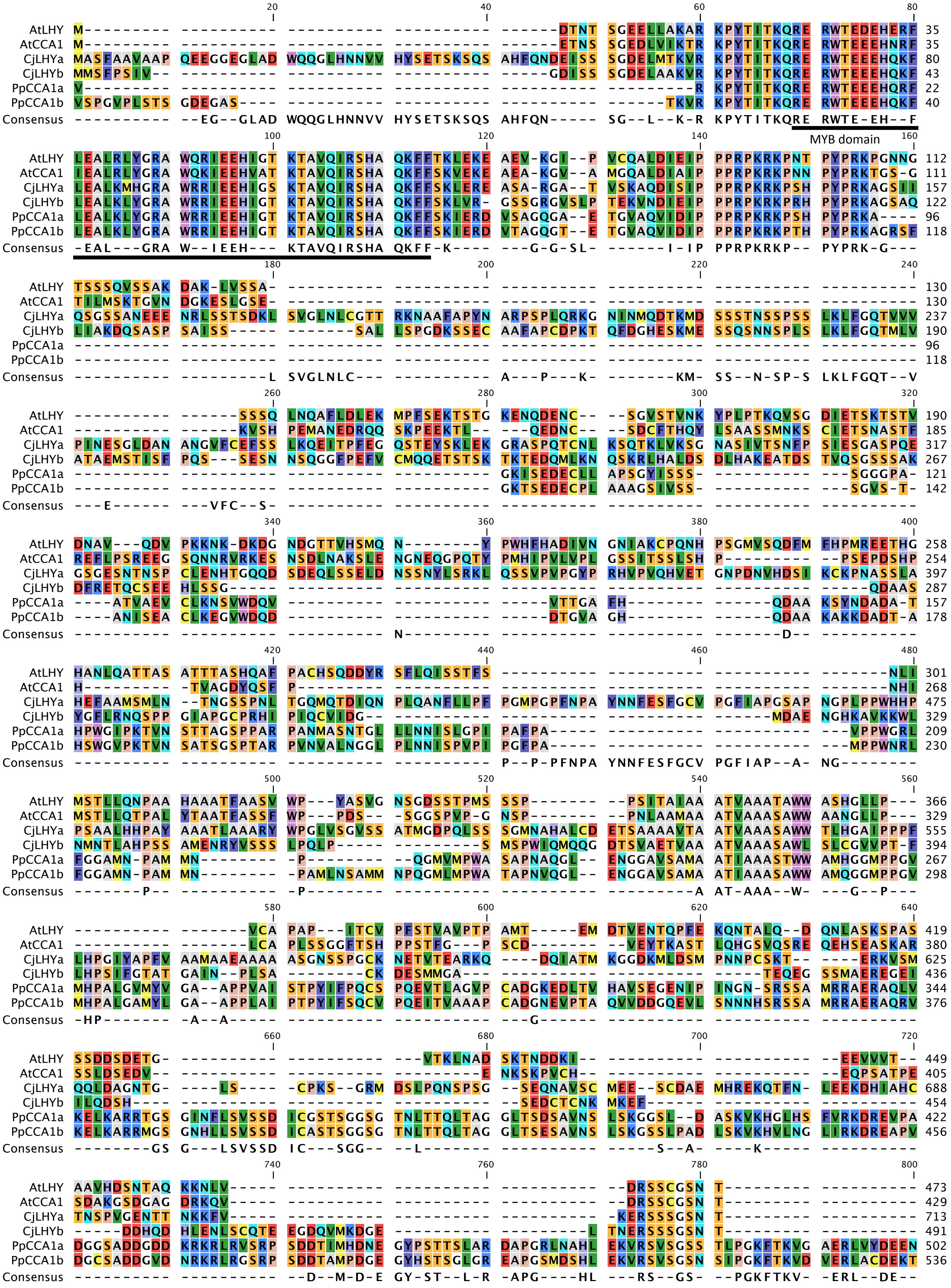
AtLHY
AtCCA1
CjLHYa
CjLHYb
PpCCA1a
PpCCA1b
Consensus
MYB domain

A

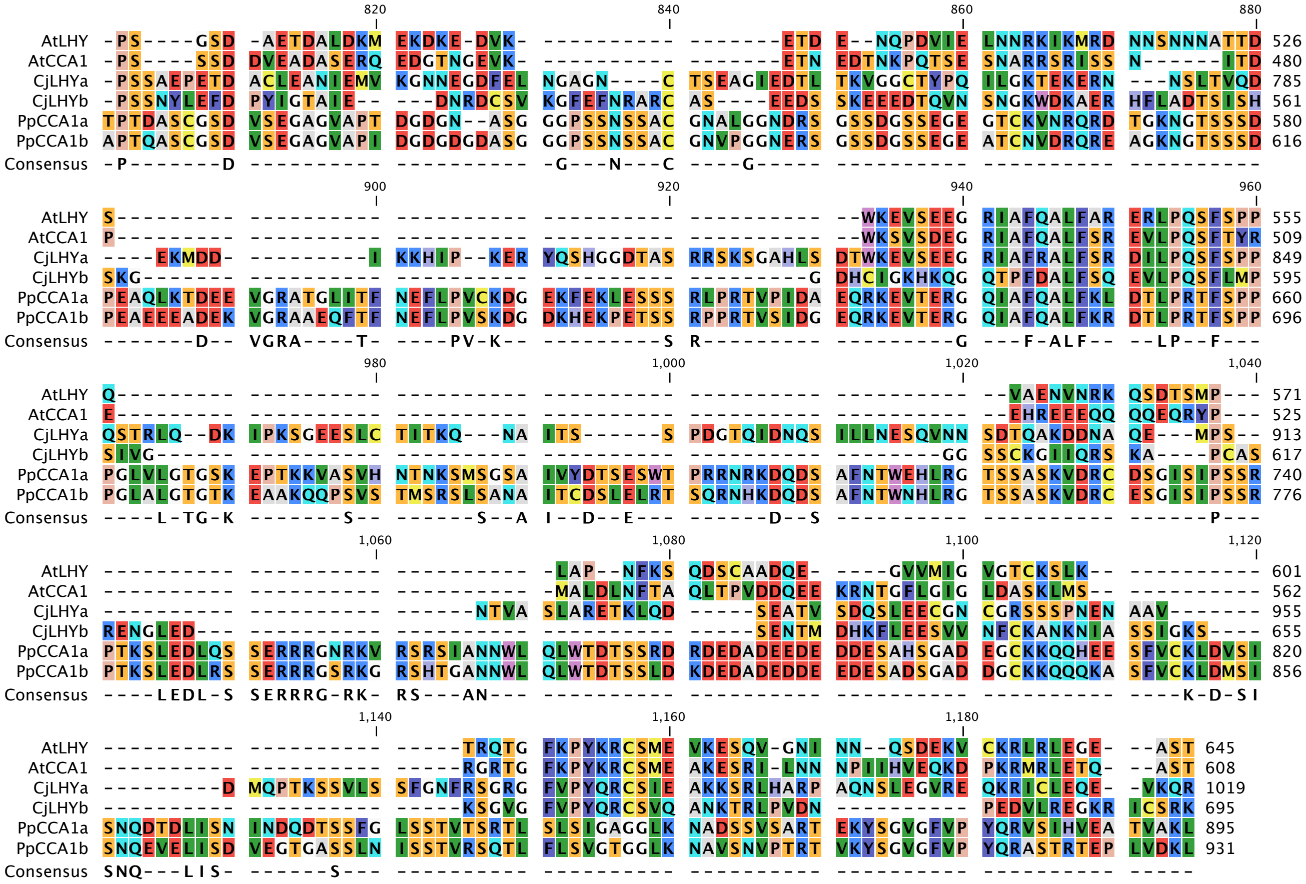
820 840 860 880
AtLHY -PS----GSD -AETDALDKM EKDKE-DVK- ---------- -------ETD E--NQPDVIE LNNRKIKMRD NNSNNNATTD 526
AtCCA1 -PS----SSD DVEADASERQ EDGTNGEVK- ---------- -------ETN EDTNKPQTSE SNARRSRISS N------ITD 480
CjLHYa -PSSAEPETD ACLEANIEMV KGNNEGDFEL NGAGN----C TSEAGIEDTL TKVGGCTYPQ ILGKTEKERN ---NSLTVQD 785
CjLHYb -PSSNYLEFD PYIGTAIE-- ---DNRDCSV KGFEFNRARC AS----EEDS SKEEEDTQVN SNGKWDKAER HFLADTSISH 561
PpCCA1a TPTDASCGSD VSEGAGVAPT DGDGN--ASG GGPSSNSSAC GNALGGNDRS GSSDGSSEGE GTCKVNRQRD TGKNGTSSSD 580
PpCCA1b APTQASCGSD VSEGAGVAPI DGDGDGDASG GGPSSNSSAC GNVPGGNERS GSSDGSSEGE ATCNVDRQRE AGKNGTSSSD 616
Consensus -P-------D ---------- ---------- -G---N---C ----G----- ---------- ---------- ----------
900 920 940 960
AtLHY S--------- ---------- ---------- ---------- ---------- --WKEVSEEG RIAFQALFAR ERLPQSFSPP 555
AtCCA1 P--------- ---------- ---------- ---------- ---------- --WKSVSDEG RIAFQALFSR EVLPQSFTYR 509
CjLHYa ----EKMDD- ---------I KKHIP--KER YQSHGGDTAS RRSKSGAHLS DTWKEVSEEG RIAFRALFSR DILPQSFSPP 849
CjLHYb SKG------- ---------- ---------- ---------- ---------G DHCIGKHKQG QTPFDALFSQ EVLPQSFLMP 595
PpCCA1a PEAQLKTDEE VGRATGLITF NEFLPVCKDG EKFEKLESSS RLPRTVPIDA EQRKEVTERG QIAFQALFKL DTLPRTFSPP 660
PpCCA1b PEAEEEADEK VGRAAEQFTF NEFLPVSKDG DKHEKPETSS RPPRTVSIDG EQRKEVTERG QIAFQALFKR DTLPRTFSPP 696
Consensus -------D-- VGRA----T- ----PV-K-- ---------S R--------- ---------G ---F-ALF-- --LP--F---
980 1,000 1,020 1,040
AtLHY Q--------- ---------- ---------- ---------- ---------- ---------- --VAENVNRK QSDTSMP--- 571
AtCCA1 E--------- ---------- ---------- ---------- ---------- ---------- --EHREEEQQ QQEQRYP--- 525
CjLHYa QSTRLQ--DK IPKSGEESLC TITKQ---NA ITS------S PDGTQIDNQS ILLNESQVNN SDTQAKDDNA QE---MPS-- 913
CjLHYb SIVG------ ---------- ---------- ---------- ---------- --------GG SSCKGIIQRS KA----PCAS 617
PpCCA1a PGLVLGTGSK EPTKKVASVH NTNKSMSGSA IVYDTSESWT PRRNRKDQDS AFNTWEHLRG TSSASKVDRC DSGISIPSSR 740
PpCCA1b PGLALGTGTK EAAKQQPSVS TMSRSLSANA ITCDSLELRT SQRNHKDQDS AFNTWNHLRG TSSASKVDRC ESGISIPSSR 776
Consensus ----L-TG-K -------S-- ------S--A I--D-E---- ------D--S ---------- ---------- -------P--
1,060 1,080 1,100 1,120
AtLHY ---------- ---------- ---------- -LAP--NFKS QDSCAADQE- ----GVVMIG VGTCKSLK-- ---------- 601
AtCCA1 ---------- ---------- ---------- -MALDLNFTA QLTPVDDQEE KRNTGFLGIG LDASKLMS-- ---------- 562
CjLHYa ---------- ---------- ------NTVA SLARETKLQD -----SEATV SDQSLEECGN CGRSSSPNEN AAV------- 955
CjLHYb RENGLED--- ---------- ---------- ---------- -----SENTM DHKFLEESVV NFCKANKNIA SSIGKS---- 655
PpCCA1a PTKSLEDLQS SERRRGNRKV RSRSIANNWL QLWTDTSSRD RDEDADEEDE DDESAHSGAD EGCKKQQHEE SFVCKLDVSI 820
PpCCA1b PTKSLEDLRS SERRRGSRKG RSHTGANNWL QLWTDTSSLD KDEDADEDDE EDESADSGAD DGCKKQQQKA SFVCKLDMSI 856
Consensus ----LEDL-S SERRRG-RK- RS---AN--- ---------- ---------- ---------- ---------- ----K-D-SI
1,140 1,160 1,180
AtLHY ---------- ---------- -----TRQTG FKPYKRCSME VKESQV-GNI NN--QSDEKV CKRLRLEGE- --AST 645
AtCCA1 ---------- ---------- -----RGRTG FKPYKRCSME AKESRI-LNN NPIIHVEQKD PKRMRLETQ- --AST 608
CjLHYa ---------D MQPTKSSVLS SFGNFRSGRG FVPYQRCSIE AKKSRLHARP AQNSLEGVRE QKRICLEQE- -VKQR 1019
CjLHYb ---------- ---------- -----KSGVG FVPYQRCSVQ ANKTRLPVDN ---------- PEDVLREGKR ICSRK 695
PpCCA1a SNQDTDLISN INDQDTSSFG LSSTVTSRTL SLSIGAGGLK NADSSVSART EKYSGVGFVP YQRVSIHVEA TVAKL 895
PpCCA1b SNQEVELISD VEGTGASSLN ISSTVRSQTL FLSVGTGGLK NAVSNVPTRT VKYSGVGFVP YQRASTRTEP LVDKL 931
Consensus SNQ---LIS- ------S--- ---------- ---------- ---------- ---------- ---------- -----

B

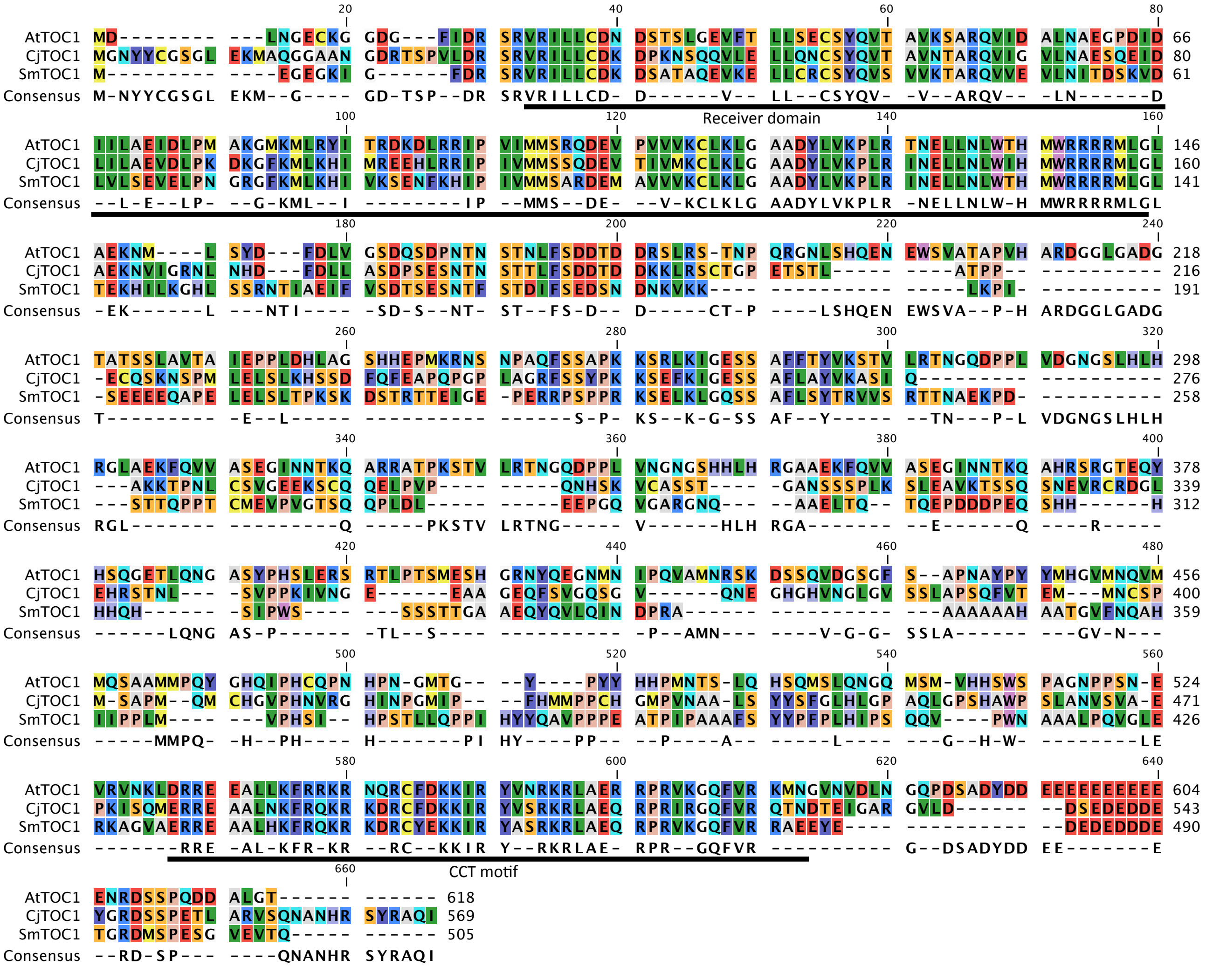

C

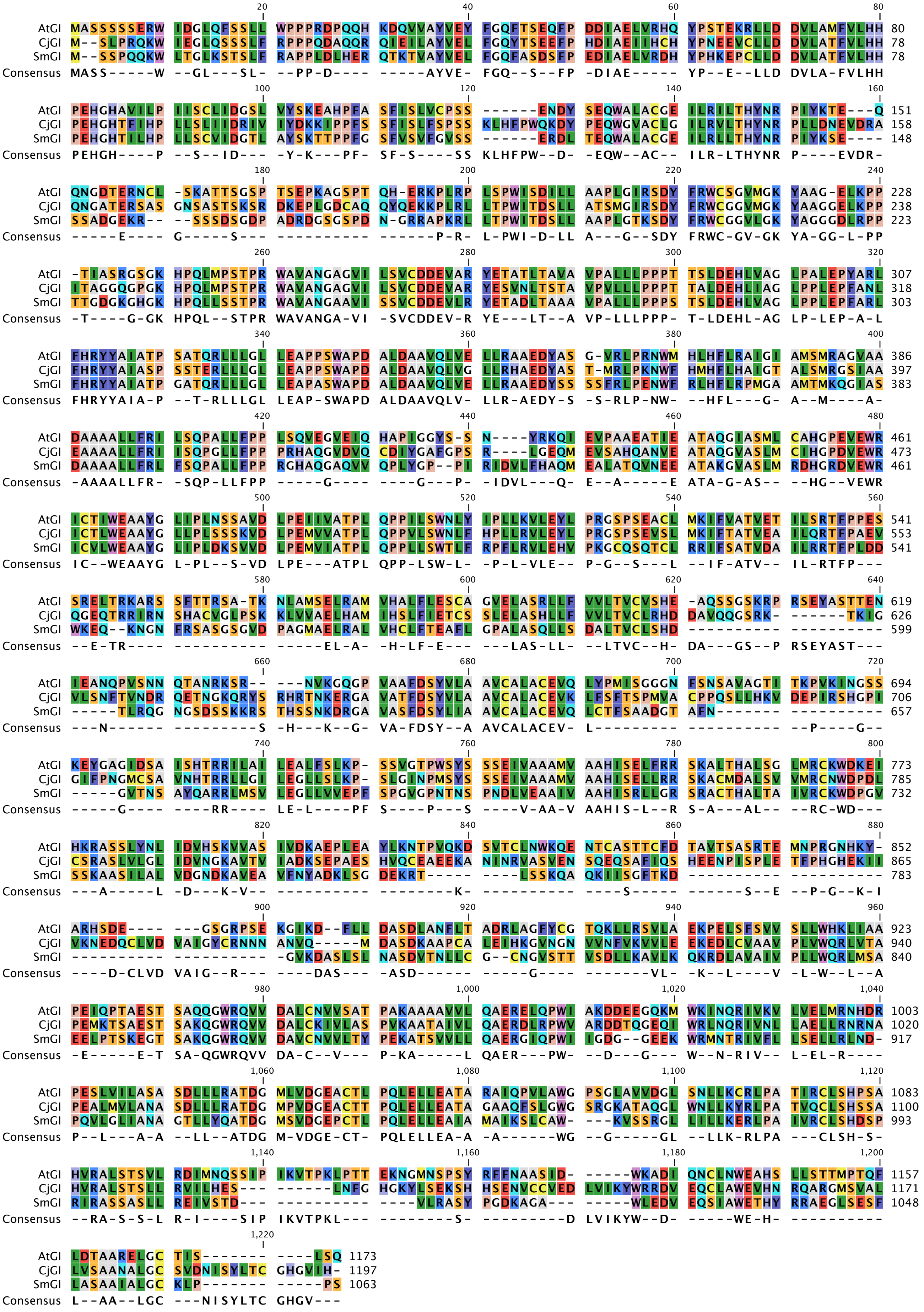
AtGI MASSSSSERW IDGLQFSSLL WPPPRDPQQH KDQVVAYVEY FGQFTSEQFP DDIAELVRHQ YPSTEKRLLD DVLAMFVLHH 80
CjGI M--SLPRQKW IEGLQSSSLF RPPPQDAQQR QIEILAYVEL FGQYTSEEFP HDIAEIIHCH YPNEEVCLLD DVLATFVLHH 78
SmGI M--SSPQQKW LTGLKSTSLF RAPPLDLHER QTKTVAYVEL FGQFASDSFP EDIAELVRDH YPHKEPCLLD DVLATFVLHH 78
Consensus MASS-----W --GL---SL- --PP-D---- -----AYVE- FGQ--S--FP -DIAE----- YP--E--LLD DVLA-FVLHH
AtGI PEHGHAVILP IISCLIDGSL VYSKEAHPFA SFISLVCPSS ------ENDY SEQWALACGE ILRILTHYNR PIYKTE---Q 151
CjGI PEHGHTFIHP LLSLIIDRIV IYDKKIPPFS SFISLFSPSS KLHFPWQKDY PEQWGVACLG ILRVLTHYNR PLLDNEVDRA 158
SmGI PEHGHTILHP LLSCVIDGTL AYSKTTPPFG SFVSVFGVSS ------ERDL TEQWALACGE ILRLLTHYNR PIYKSE---- 148
Consensus PEHGH----P --S--ID--- -Y-K---PF- SF-S----SS KLHFPW--D- -EQW--AC-- ILR-LTHYNR P----EVDR-
AtGI QNGDTERNCL -SKATTSGSP TSEPKAGSPT QH-ERKPLRP LSPWISDILL AAPLGIRSDY FRWCSGVMGK YAAG-ELKPP 228
CjGI QNGATERSAS GNSASTSKSR DKEPLGDCAQ QYQEKKPLRL LTPWITDSLL ATSMGIRSDY FRWCGGVMGK YAAGGELKPP 238
SmGI SSADGEKR-- --SSSDSGDP ADRDGSGSPD N-GRRAPKRL LTPWITDSLL AAPLGTKSDY FRWCGGVLGK YAGGGDLRPP 223
Consensus -----E---- G-----S--- ---------- ------P-R- L-PWI-D-LL A---G--SDY FRWC-GV-GK YA-GG-L-PP
AtGI -TIASRGSGK HPQLMPSTPR WAVANGAGVI LSVCDDEVAR YETATLTAVA VPALLLPPPT TSLDEHLVAG LPALEPYARL 307
CjGI ITAGGQGPGK HPQLMPSTPR WAVANGAGVI LSVCDDEVAR YESVNLTSTA VPVLLLPPPT TALDEHLIAG LPPLEPFANL 318
SmGI TTGDGKGHGK HPQLLSSTPR WAVANGAAVI SSVCDDEVLR YETADLTAAA VPALLLPPPS TSLDEHLVAG LPPLEPFARL 303
Consensus -T----G-GK HPQL--STPR WAVANGA-VI -SVCDDEV-R YE---LT--A VP-LLLPPP- T-LDEHL-AG LP-LEP-A-L
AtGI FHRYYAIATP SATQRLLLGL LEAPPSWAPD ALDAAVQLVE LLRAAEDYAS G-VRLPRNWM HLHFLRAIGI AMSMRAGVAA 386
CjGI FHRYYAIASP SSTERLLLGL LEAPPSWAPD ALDAAVQLVG LLRHAEDYAS T-MRLPKNWF HMHFLHAIGT ALSMRGSIAA 397
SmGI FHRYYAIATP GATQRLLLGL LEAPASWAPD ALDAAVQLVE LLRAAEDYSS SSFRLPENWF RLHFLRPMGA AMTMKQGIAS 383
Consensus FHRYYAIA-P --T-RLLLGL LEAP-SWAPD ALDAAVQLV- LLR-AEDY-S -S-RLP-NW- --HFL---G- A--M----A-
AtGI DAAAALLFRI LSQPALLFPP LSQVEGVEIQ HAPIGGYS-S N----YRKQI EVPAAEATIE ATAQGIASML CAHGPEVEWR 461
CjGI EAAAALLFRI ISQPGLLFPP PRHAQGVDVQ CDIYGAFGPS R----LGEQM EVSAHQANVE ATAQGVASLM CIHGPDVEWR 473
SmGI DAAAALLFRL FSQPALLFPP RGHAQGAQVV QPLYGP--PI RIDVLFHAQM EALATQVNEE ATAKGVASLM RDHGRDVEWR 461
Consensus -AAAALLFR- -SQP-LLFPP -----G---- ----G---P- -IDVL---Q- E--A-----E ATA-G-AS-- --HG--VEWR
AtGI ICTIWEAAYG LIPLNSSAVD LPEIIVATPL QPPILSWNLY IPLLKVLEYL PRGSPSEACL MKIFVATVET ILSRTFPPES 541
CjGI ICTLWEAAYG LLPLSSSKVD LPEMVVATPL QPPVLSWNLF HPLLRVLEYL PRGSPSEVSL MKIFTATVEA ILQRTFPAEV 553
SmGI ICVLWEAAYG LIPLDKSVVD LPEMVIATPL QPPLLSWTLF RPFLRVLEHV PKGCQSQTCL RRIFSATVDA ILRRTFPLDD 541
Consensus IC--WEAAYG L-PL--S-VD LPE---ATPL QPP-LSW-L- -P-L-VLE-- P-G--S---L --IF-ATV-- IL-RTFP---
AtGI SRELTRKARS SFTTRSA-TK NLAMSELRAM VHALFLESCA GVELASRLLF VVLTVCVSHE -AQSSGSKRP RSEYASTTEN 619
CjGI QGEQTRRIRN SHACVGLPSK KLVVAELHAM IHSLFIETCS SLELASHLLF VVLTVCLRHD DAVQQGSRK- ------TKIG 626
SmGI WKEQ--KNGN FRSASGSGVD PAGMAELRAL VHCLFTEAFL GPALASQLLS DALTVCLSHD ---------- ---------- 599
Consensus --E-TR---- ---------- -----EL-A- -H-LF-E--- ---LAS-LL- --LTVC--H- DA---GS--P RSEYAST---
AtGI IEANQPVSNN QTANRKSR-- ---NVKGQGP VAAFDSYVLA AVCALACEVQ LYPMISGGGN FSNSAVAGTI TKPVKINGSS 694
CjGI VLSNFTVNDR QETNGKQRYS RHRTNKERGA VATFDSYVLA AVCALACEVK LFSFTSPMVA CPPQSLLHKV DEPIRSHGPI 706
SmGI -----TLRQG NGSDSSKKRS THSSNKDRGA VASFDSYLIA AVCALACEVQ LCTFSAADGT AFN------- ---------- 657
Consensus ---N------ ---------S -H---K--G- VA-FDSY--A AVCALACEV- L--------- ---------- --P----G--
AtGI KEYGAGIDSA ISHTRRILAI LEALFSLKP- SSVGTPWSYS SSEIVAAAMV AAHISELFRR SKALTHALSG LMRCKWDKEI 773
CjGI GIFPNGMCSA VNHTRRLLGI LEGLLSLKP- SLGINPMSYS SSEIVAAAMV AAHISELLRR SKACMDALSV VMRCNWDPDL 785
SmGI -----GVTNS AYQARRLMSV LEGLLVVEPF SPGVGPNTNS PNDLVEAAIV AAHISRLLGR SRACTHALTA IVRCKWDPGV 732
Consensus -----G---- ----RR---- LE-L----PF S----P---S ----V-AA-V AAHIS-L--R S-A---AL-- --RC-WD---
AtGI HKRASSLYNL IDVHSKVVAS IVDKAEPLEA YLKNTPVQKD SVTCLNWKQE NTCASTTCFD TAVTSASRTE MNPRGNHKY- 852
CjGI CSRASLVLGL IDVNGKAVTV IADKSEPAES HVQCEAEEKA NINRVASVEN SQEQSAFIQS HEENPISPLE TFPHGHEKII 865
SmGI SSKAASILAL VDGNDKAVEA VFNYADKLSG DEKRT----- ----LSSKQA QKIISGFTKD ---------- ---------- 783
Consensus ---A-----L -D---K-V-- ---------- --------K- ---------- ----S----- ------S--E --P-G--K-I
AtGI ARHSDE---- ---GSGRPSE KGIKD--FLL DASDLANFLT ADRLAGFYCG TQKLLRSVLA EKPELSFSVV SLLWHKLIAA 923
CjGI VKNEDQCLVD VAIGYCRNNN ANVQ-----M DASDKAAPCA LEIHKGVNGN VVNFVKVVLE EKEDLCVAAV PLVWQRLVTA 940
SmGI ---------- ---------- -GVKDASLSL NASDVTNLLC G--CNGVSTT VSDLLKAVLK QKRDLAVAIV PLLWQRLMSA 840
Consensus ----D-CLVD VAIG--R--- ----DAS--- -ASD------ -----G---- -------VL- -K--L----V -L-W--L--A
AtGI PEIQPTAEST SAQQGWRQVV DALCNVVSAT PAKAAAAVLL QAERELQPWI AKDDEEGQKM WKINQRIVKV LVELMRNHDR 1003
CjGI PEMKTSAEST SAKQGWRQVV DALCKIVLAS PVKAATAIVL QAERDLRPWV ARDDTQGEQI WRLNQRIVNL LAELLRNRNA 1020
SmGI EELPTSKEGT SAKQGWRQVV DAVCNVVLTY PEKATSVVLL QAERGIQPWI IGDG--GEEK WRMNTRIVFL LSELLRLND- 917
Consensus -E-----E-T SA-QGWRQVV DA-C--V--- P-KA-----L QAER---PW- --D---G--- W--N-RIV-- L-EL-R----
AtGI PESLVILASA SDLLLRATDG MLVDGEACTL PQLELLEATA RAIQPVLAWG PSGLAVVDGL SNLLKCRLPA TIRCLSHPSA 1083
CjGI PEALMVLANA SDLLLRATDG MPVDGEACTT PQLELLEATA GAAQFSLGWG SRGKATAQGL WNLLKYRLPA TVQCLSHSSA 1100
SmGI PQVLGLIANA GTLLYQATDG MSVDGEPCTL PQLELLEAIA MAIKSLCAW- ---KVSSRGL LILLKERLPA IVRCLSHDSP 993
Consensus P--L---A-A --LL--ATDG M-VDGE-CT- PQLELLEA-A -A------WG --G-----GL --LLK-RLPA ---CLSH-S-
AtGI HVRALSTSVL RDIMNQSSIP IKVTPKLPTT EKNGMNSPSY RFFNAASID- -----WKADI QNCLNWEAHS LLSTTMPTQF 1157
CjGI HVRALSTSLL RVILHES--- ------LNFG HGKYLSEKSH HSENVCCVED LVIKYWRRDV EQCLAWEVHN RQARGMSVAL 1171
SmGI RIRASSASLL REIVSTD--- ---------- ----VLRASY PGDKAGA--- -----WLEDV EQSIAWETHY RRAEGLSESF 1048
Consensus --RA-S-S-L R-I----SIP IKVTPKL--- --------S- ---------D LVIKYW--D- -----WE-H- ----------
AtGI LDTAARELGC TIS------- ----LSQ 1173
CjGI LVSAANALGC SVDNISYLTC GHGVIH- 1197
SmGI LASAAIALGC KLP------- -----PS 1063
Consensus L--AA--LGC ---NISYLTC GHGV---

D

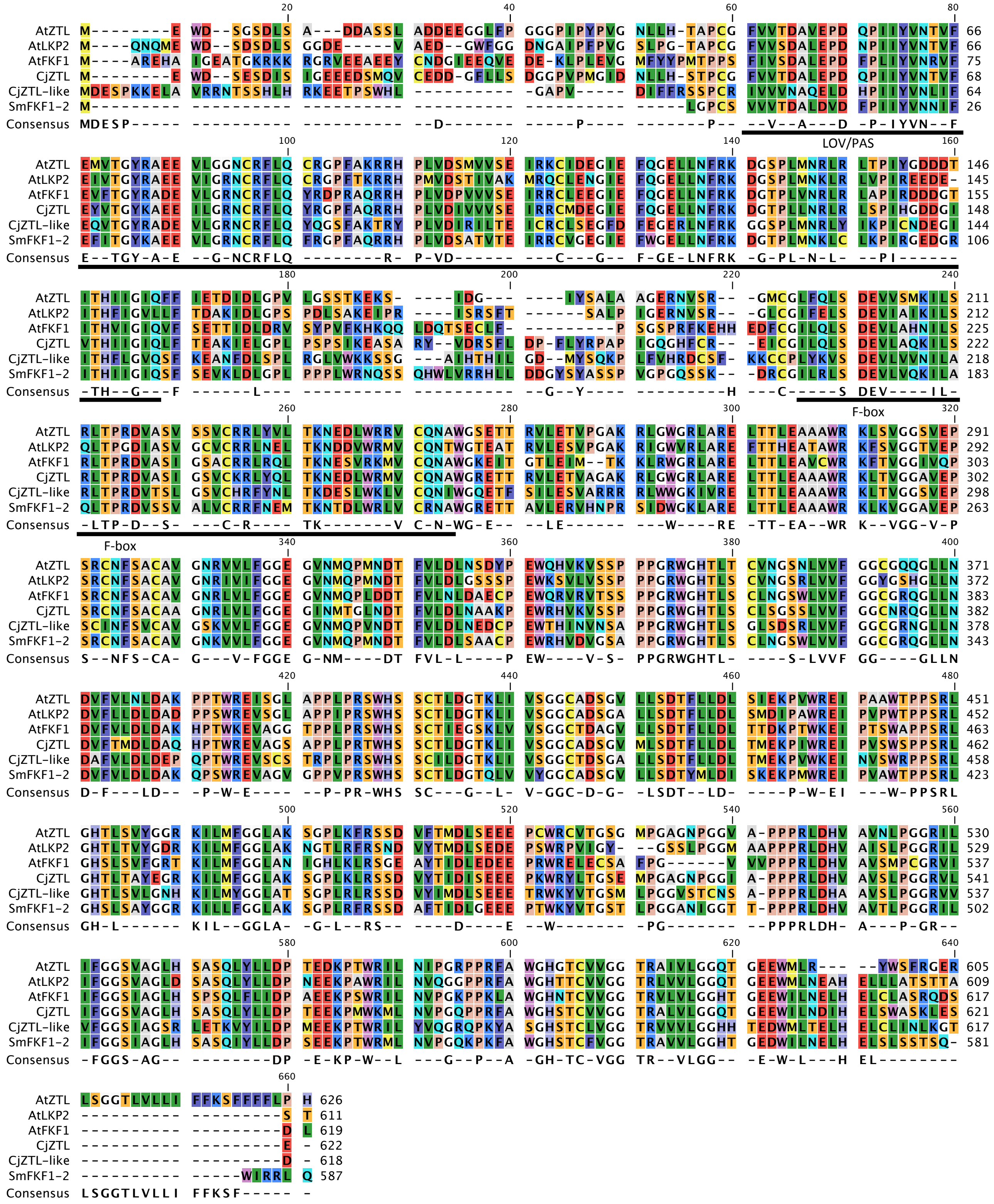
20 40 60 80
AtZTL M--------E WD--SGSDLS A---DDASSL ADDEEGGLFP GGGPIPYPVG NLLH-TAPCG FVVTDAVEPD QPIIYVNTVF 66
AtLKP2 M----QNQME WD--SDSDLS GGDE-----V AED--GWFGG DNGAIPFPVG SLPG-TAPCG FVVSDALEPD NPIIYVNTVF 66
AtFKF1 M----AREHA IGEATGKRKK RGRVEEAEEY CNDGIEEQVE DE-KLPLEVG MFYYPMTPPS FIVSDALEPD FPLIYVNRVF 75
CjZTL M--------E WD--SESDIS IGEEEDSMQV CEDD-GFLLS DGGPVPMGID NLLH-STPCG FVVTDALEPD QPIIYVNTVF 68
CjZTL-like MDESPKKELA VRRNTSSHLH RKEETPSWHL ---------- -GAPV----- DIFFRSSPCR IVVVNAQELD HPIIYVNLIF 64
SmFKF1-2 M--------- ---------- ---------- ---------- ---------- -----LGPCS VVVTDALDVD FPIIYVNNIF 26
Consensus MDESP----- ---------- ---------- --D------- -----P---- -------P-- --V--A---D -P-IYVN--F
LOV/PAS
100 120 140 160
AtZTL EMVTGYRAEE VLGGNCRFLQ CRGPFAKRRH PLVDSMVVSE IRKCIDEGIE FQGELLNFRK DGSPLMNRLR LTPIYGDDDT 146
AtLKP2 EIVTGYRAEE VIGRNCRFLQ CRGPFTKRRH PMVDSTIVAK MRQCLENGIE FQGELLNFRK DGSPLMNKLR LVPIREEDE- 145
AtFKF1 EVFTGYRADE VLGRNCRFLQ YRDPRAQRRH PLVDPVVVSE IRRCLEEGIE FQGELLNFRK DGTPLVNRLR LAPIRDDDGT 155
CjZTL EYVTGYKAEE ILGRNCRFLQ YRGPFAQRRH PLVDIVVVSE IRRCMDEGIE FQGELLNFRK DGTPLLNRLR LSPIHGDDGI 148
CjZTL-like EQVTGYRADE VLGRNCRFLQ YQGSFAKTRY PLVDIRILTE ICRCLSEGFD FEGERLNFRK GGSPLMNRLY IKPICNDEGI 144
SmFKF1-2 EFITGYKAEE VLGRNCRFLQ FRGPFAQRRH PLVDSATVTE IRRCVGEGIE FWGELLNFRK DGTPLMNKLC LKPIRGEDGR 106
Consensus E--TGY-A-E --G-NCRFLQ --------R- P-VD------ ---C---G-- F-GE-LNFRK -G-PL-N-L- --PI------
180 200 220 240
AtZTL ITHIIGIQFF IETDIDLGPV LGSSTKEKS- ----IDG--- ----IYSALA AGERNVSR-- -GMCGLFQLS DEVVSMKILS 211
AtLKP2 ITHFIGVLLF TDAKIDLGPS PDLSAKEIPR ----ISRSFT ------SALP IGERNVSR-- -GLCGIFELS DEVIAIKILS 212
AtFKF1 ITHVIGIQVF SETTIDLDRV SYPVFKHKQQ LDQTSECLF- ---------P SGSPRFKEHH EDFCGILQLS DEVLAHNILS 225
CjZTL VTHIIGIQLF TEAKIELGPL PSPSIKEASA RY--VDRSFL DP-FLYRPAP IGQGHFCR-- -EICGILQLS DEVLAQKILS 222
CjZTL-like ITHFLGVQSF KEANFDLSPL RGLVWKKSSG ---AIHTHIL GD--MYSQKP LFVHRDCSF- KKCCPLYKVS DEVLVVNILA 218
SmFKF1-2 ITHIIGIQSF SEVKLDLGPL PPPLWRNQSS QHWLVRRHLL DDGYSYASSP VGPGQSSK-- -DRCGILRLS DEVLVQKILA 183
Consensus -TH--G---F ------L--- ---------- ---------- --G--Y---- ---------H ---C-----S DEV----IL-
F-box
260 280 300 320
AtZTL RLTPRDVASV SSVCRRLYVL TKNEDLWRRV CQNAWGSETT RVLETVPGAK RLGWGRLARE LTTLEAAAWR KLSVGGSVEP 291
AtLKP2 QLTPGDIASV GCVCRRLNEL TKNDDVWRMV CQNTWGTEAT RVLESVPGAK RIGWVRLARE FTTHEATAWR KFSVGGTVEP 292
AtFKF1 RLTPRDVASI GSACRRLRQL TKNESVRKMV CQNAWGKEIT GTLEIM--TK KLRWGRLARE LTTLEAVCWR KFTVGGIVQP 303
CjZTL RLTPRDVASI GSVCKRLYQL TKNEDLWRMV CQNAWGRETT RVLETVAGAK RLGWGRLARE LTTLEAAAWR KLTVGGAVEP 302
CjZTL-like RLTPRDVTSL GSVCHRFYNL TKDESLWKLV CQNIWGQETF SILESVARRR RLWWGKIVRE LTTLEAAAWR KLTVGGSVEP 298
SmFKF1-2 QLTPRDVSSV ALVCRRFNEM TKNTDLWRLV CRNAWGRETT AVLERVHNPR SIDWGKLARE LTTLEAAAWR KLKVGGAVEP 263
Consensus -LTP-D--S- ---C-R---- TK-------V C-N-WG-E-- --LE------ ---W----RE -TT-EA--WR K--VGG-V-P
F-box
340 360 380 400
AtZTL SRCNFSACAV GNRVVLFGGE GVNMQPMNDT FVLDLNSDYP EWQHVKVSSP PPGRWGHTLT CVNGSNLVVF GGCGQQGLLN 371
AtLKP2 SRCNFSACAV GNRIVIFGGE GVNMQPMNDT FVLDLGSSSP EWKSVLVSSP PPGRWGHTLS CVNGSRLVVF GGYGSHGLLN 372
AtFKF1 SRCNFSACAV GNRLVLFGGE GVNMQPLDDT FVLNLDAECP EWQRVRVTSS PPGRWGHTLS CLNGSWLVVF GGCGRQGLLN 383
CjZTL SRCNFSACAA GNRLVLFGGE GINMTGLNDT FVLDLNAAKP EWRHVKVSSP PPGRWGHTLS CLSGSSLVVF GGCNRQGLLN 382
CjZTL-like SCINFSVCAV GSKVVLFGGE GVNMQPVNDT FVLDLNEDCP EWTHINVNSA PPGRWGHTLS GLSDSRLVVF GGCGRNGLLN 378
SmFKF1-2 SRCNFSACAV GNKVVLFGGE GVNMQPMNDT FVLDLSAACP EWRHVDVGSA PPGRWGHTLS CLNGSWLVVF GGCGRQGLLN 343
Consensus S--NFS-CA- G---V-FGGE G-NM----DT FVL-L----P EW----V-S- PPGRWGHTL- ----S-LVVF GG----GLLN
420 440 460 480
AtZTL DVFVLNLDAK PPTWREISGL APPLPRSWHS SCTLDGTKLI VSGGCADSGV LLSDTFLLDL SIEKPVWREI PAAWTPPSRL 451
AtLKP2 DVFLLDLDAD PPSWREVSGL APPIPRSWHS SCTLDGTKLI VSGGCADSGA LLSDTFLLDL SMDIPAWREI PVPWTPPSRL 452
AtFKF1 DVFVLDLDAK HPTWKEVAGG TPPLPRSWHS SCTIEGSKLV VSGGCTDAGV LLSDTFLLDL TTDKPTWKEI PTSWAPPSRL 463
CjZTL DVFTMDLDAQ HPTWREVAGS APPLPRTWHS SCTLDGTKLI VSGGCADSGV MLSDTFLLDL TMEKPIWREI PVSWSPPSRL 462
CjZTL-like DAFVLDLDEP QPTWREVSCS TRPLPRSWHS SCILDGTKLI VSGGCTDSGA LLSDTFLLDL TMEKPTWKEI NVSWRPPSRL 458
SmFKF1-2 DVFVLDLDAK QPSWREVAGV GPPVPRSWHS SCTLDGTQLV VYGGCADSGV LLSDTYMLDI SKEKPMWREI PVAWTPPSRL 423
Consensus D-F---LD-- -P-W-E---- --P-PR-WHS SC---G--L- V-GGC-D-G- -LSDT--LD- ----P-W-EI ---W-PPSRL
500 520 540 560
AtZTL GHTLSVYGGR KILMFGGLAK SGPLKFRSSD VFTMDLSEEE PCWRCVTGSG MPGAGNPGGV A-PPPRLDHV AVNLPGGRIL 530
AtLKP2 GHTLTVYGGR KILMFGGLAK NGTLRFRSND VYTMDLSEDE PSWRPVIGY- --GSSLPGGM AAPPPRLDHV AISLPGGRIL 529
AtFKF1 GHSLSVFGRT KILMFGGLAN IGHLKLRSGE AYTIDLEDEE PRWRELECSA FPG------V VVPPPRLDHV AVSMPCGRVI 537
CjZTL GHTLTAYEGR KILMFGGLAK SGPLKLRSSD VYTIDISEEE PKWRYLTGSE MPGAGNPGGI A-PPPRLDHV AVSLPGGRVL 541
CjZTL-like GHTLSVLGNH KILMYGGLAT SGPLRLRSSD VYIMDLSEEE TRWKYVTGSM LPGGVSTCNS A-PPPRLDHA AVSLPGGRVV 537
SmFKF1-2 GHSLSAYGGR KILLFGGLAK SGPLRFRSSD AFTIDLGEEE PTWKYVTGST LPGGANIGGT T-PPPRLDHV AVTLPGGRIL 502
Consensus GH-L------ KIL--GGLA- -G-L--RS-- ----D----E --W------- -PG------- --PPPRLDH- A---P-GR--
580 600 620 640
AtZTL IFGGSVAGLH SASQLYLLDP TEDKPTWRIL NIPGRPPRFA WGHGTCVVGG TRAIVLGGQT GEEWMLR--- --YWSFRGER 605
AtLKP2 IFGGSVAGLD SASQLYLLDP NEEKPAWRIL NVQGGPPRFA WGHTTCVVGG TRLVVLGGQT GEEWMLNEAH ELLLATSTTA 609
AtFKF1 IFGGSIAGLH SPSQLFLIDP AEEKPSWRIL NVPGKPPKLA WGHNTCVVGG TRVLVLGGHT GEEWILNELH ELCLASRQDS 617
CjZTL IFGGSVAGLH SASQLYLLDP TEEKPMWKML NVPGQPPRFA WGHSTCVVGG TRALVLGGQT GEEWILNDIH ELSWASKLES 621
CjZTL-like VFGGSIAGSR LETKVYILDP MEEKPTWRIL YVQGRQPKYA SGHSTCLVGG TRVVVLGGHH TEDWMLTELH ELCLINLKGT 617
SmFKF1-2 IFGGSIAGLH SASQIYLLDP SEEKPTWRML NVPGQKPKFA WGHSTCFVGG TRAVVLGGHT GEDWILNELH ELSLSSTSQ- 581
Consensus -FGGS-AG-- --------DP -E-KP-W--L ---G--P--A -GH-TC-VGG TR--VLGG-- -E-W-L---H EL--------
660
AtZTL LSGGTLVLLI FFKSFFFFLP H 626
AtLKP2 ---------- ---------S T 611
AtFKF1 ---------- ---------D L 619
CjZTL ---------- ---------E - 622
CjZTL-like ---------- ---------D - 618
SmFKF1-2 ---------- -----WIRRL Q 587
Consensus LSGGTLVLLI FFKSF----- -

Additional file 9. Amino acid sequence alignment of LHY and CCA1 (A), TOC1 (B), GI (C), and ZTL, LKP2 and FKF1 (D).

The species names are abbreviated as follows: At, *Arabidopsis thaliana*; Cj, *Cryptomeria japonica*; Pp, *Physcomitrella patens* subsp. *patens*; Sm, *Selaginella moellendorffii*.

(A) NCBI accession numbers of the proteins are AtLHY (NP_001030924) and AtCCA1 (NP_850460). PpCCA1a and PpCCA1b are from Okada *et al.* [41]. The amino acid sequences of the domains (underlined) are from Wang *et al.* [40].

(B) NCBI accession numbers of the proteins are NP_200946 (AtTOC1) and XP_002963903 (SmTOC1). The amino acid sequences of the domains (underlined) are from Strayer *et al.* [42].

(C) NCBI accession numbers of the proteins are NP_564180 (AtGI) and XP_002961231 (SmGI).

(D) NCBI accession numbers of the proteins are NP_001154783 (AtZTL), AEC06826 (AtLKP2), AAF32298 (FKF1) and XP_002990856 (SmFKF1-2). The amino acid sequences of the domains (underlined) are from Somers *et al.* [43].
