# Supplementary material for: Clock genes and diurnal transcriptome dynamics in summer and winter in the gymnosperm Japanese cedar (Cryptomeria japonica (L.f.) D.Don)
Source: BMC Plant Biol. 2014 Nov 18;14:308. doi: 10.1186/s12870-014-0308-1 (PMC4245765; doi:10.1186/s12870-014-0308-1)
Supplement: Additional file 10: — Diurnal expression patterns of photoreceptor genes in summer and winter. Diurnal changes in relative transcript abundance in summer (Jul 30–31, 2012) and winter (Dec 22–23, 2011) were analyzed by microarray and qPCR. The microarray data of summer samples (green line) and qPCR data of summer (blue line) and winter (red line) samples represent the mean from three biological replicates of 12 time points. The microarray data of winter samples (purple line) are shown at 8 selected time points (4:00/8:00/12:00/16:00/20:00/24:00 on day 1 and 12:00/24:00 on day 2) without biological replication. The data obtained for each time point were compared with the data obtained for shoots collected at 4:00 on Jul 30, 2012. Gray and black bars below graph represent length of natural day and night (between sunrise and sunset), respectively, in summer (upper) and winter (lower) as reported by the National Astronomical Observatory of Japan. [file 12870_2014_308_MOESM10_ESM.pdf]

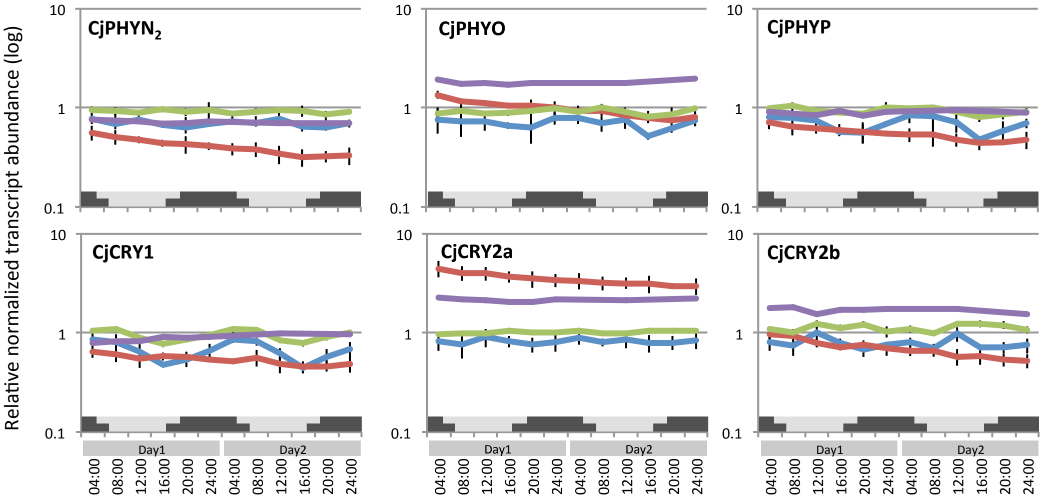

Additional file 10. Diurnal expression patterns of photoreceptor genes in summer and winter.

Diurnal changes in relative transcript abundance in summer (Jul 30-31, 2012) and winter (Dec 22-23, 2011) were analyzed by microarray and qPCR. The microarray data of summer samples (green line) and qPCR data of summer (blue line) and winter (red line) samples represent the mean from three biological replicates of 12 time points. The microarray data of winter samples (purple line) are shown at 8 selected time points (4:00/8:00/12:00/16:00/20:00/24:00 on day 1 and 12:00/24:00 on day 2) without biological replication. The data obtained for each time point were compared with the data obtained for shoots collected at 4:00 on Jul 30, 2012. Gray and black bars below graph represent length of natural day and night (between sunrise and sunset), respectively, in summer (upper) and winter (lower) as reported by the National Astronomical Observatory of Japan.
