# Supplementary material for: Clock genes and diurnal transcriptome dynamics in summer and winter in the gymnosperm Japanese cedar (Cryptomeria japonica (L.f.) D.Don)
Source: BMC Plant Biol. 2014 Nov 18;14:308. doi: 10.1186/s12870-014-0308-1 (PMC4245765; doi:10.1186/s12870-014-0308-1)
Supplement: Additional file 11: — Diurnal changes in temperature, photosynthetically active radiation and growth in height of Japanese cedar. (A) Changes in temperature over two days in winter (Dec 22–23, 2011) and summer (Jul 30–31, 2012). Temperature data were collected every 10 min in Hitachi (36°34′N 140°38′E 34 m, about 15 km from the sampling site) and were provided by the Japan Meteorological Agency (http://www.jma.go.jp/jma/index.html). (B) Changes in photosynthetically active radiation over two days in summer. Values were obtained every 20 min from the photosynthetically active radiation smart sensor working with the HOBO Weather Station logger (Onset Computer Corp., Bourne, MA, USA) at the sampling site. (C) Change in growth in height over five days (Jul 26–30, 2012). Images of a treetop were captured every hour by a WG-II digital camera (Pentax, Tokyo, Japan). Growth in height was estimated by measuring the images with ImageJ64 software (http://rsbweb.nih.gov/ij/). [file 12870_2014_308_MOESM11_ESM.pdf]

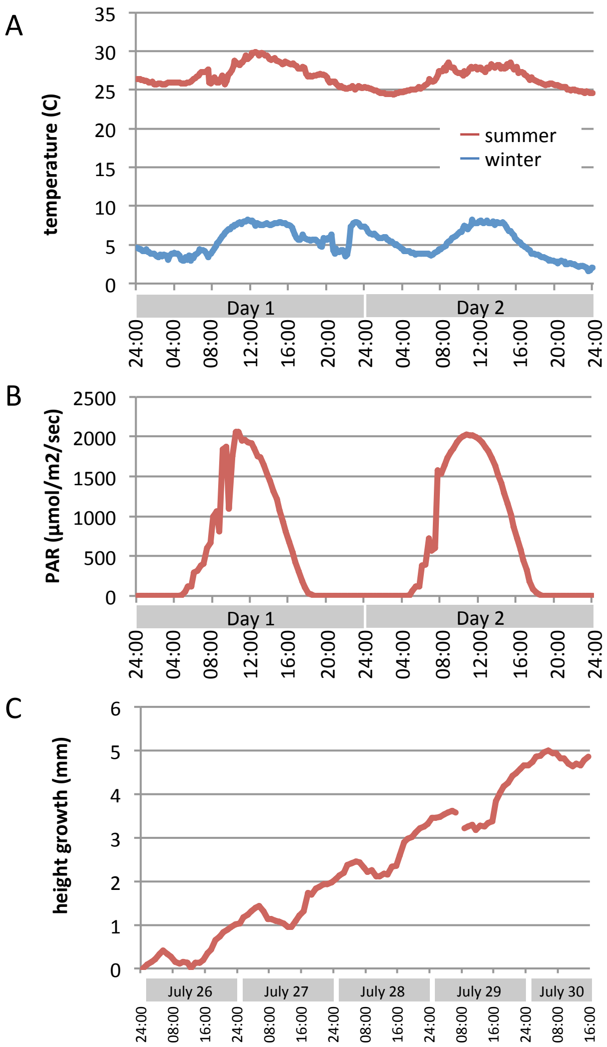

Additional file 11. Diurnal changes in temperature, photosynthetically active radiation and growth in height of Japanese cedar.

(A) Changes in temperature over two days in winter (Dec 22-23, 2011) and summer (Jul 30-31, 2012). Temperature data were collected every 10 min in Hitachi (36°34'N 140°38'E 34 m, about 15 km from the sampling site) and were provided by the Japan Meteorological Agency (http://www.jma.go.jp/jma/index.html).

(B) Changes in photosynthetically active radiation over two days in summer. Values were obtained every 20 min from the photosynthetically active radiation smart sensor working with the HOBO Weather Station logger (Onset Computer Corp., Bourne, MA, USA) at the sampling site.

(C) Change in growth in height over five days (Jul 26-30, 2012). Images of a treetop were captured every hour by a WG-II digital camera (Pentax, Tokyo, Japan). Growth in height was estimated by measuring the images with ImageJ64 software (http://rsbweb.nih.gov/ij/).
