# Supplementary material for: Clock genes and diurnal transcriptome dynamics in summer and winter in the gymnosperm Japanese cedar (Cryptomeria japonica (L.f.) D.Don)
Source: BMC Plant Biol. 2014 Nov 18;14:308. doi: 10.1186/s12870-014-0308-1 (PMC4245765; doi:10.1186/s12870-014-0308-1)
Supplement: Additional file 12: — Phylogenetic tree of LHCa/b in Arabidopsis , rice, moss and Japanese cedar. LHCa/b gene sequences of Japanese cedar were extracted from the NGS data. Species names are abbreviated as follows: At, Arabidopsis thaliana (thale cress); Cj, Japanese cedar (Cryptomeria japonica); Osj, Oryza sativa (Japanese rice); Pp, Physcomitrella patens subsp. patens (moss). The number following the species name indicates NCBI accession number. The neighbor-joining method [77] was used to construct the phylogenetic trees. Trees were rooted with Arabidopsis ELIP1 and ELIP2. [file 12870_2014_308_MOESM12_ESM.pdf]

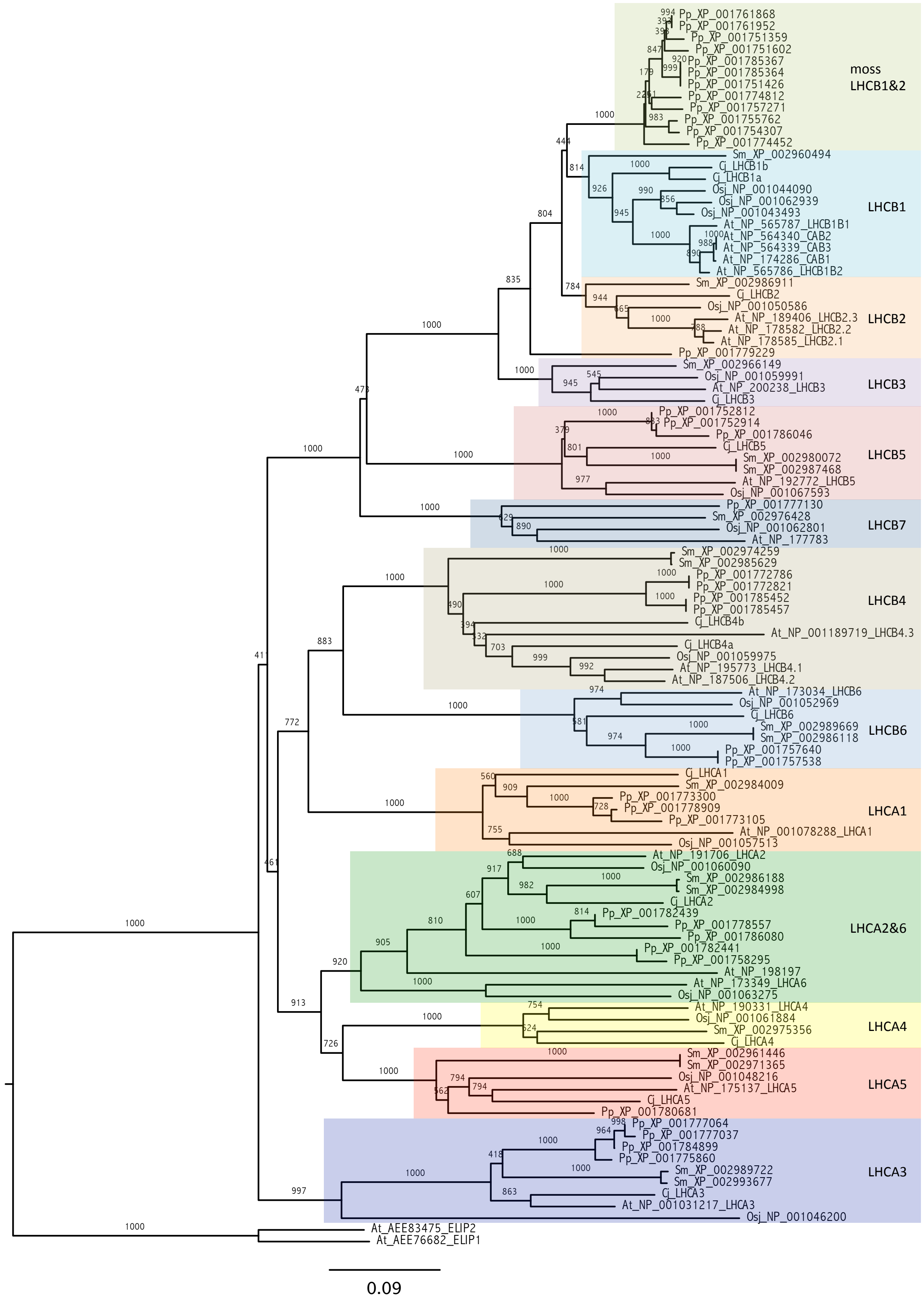

Additional file 12. Phylogenetic tree of LHCa/b in *Arabidopsis*, rice, moss and Japanese cedar.

LHCa/b gene sequences of Japanese cedar were extracted from the NGS data. Species names are abbreviated as follows: At, *Arabidopsis thaliana* (thale cress); Cj, Japanese cedar (*Cryptomeria japonica*); Osj, *Oryza sativa* (Japanese rice); Pp, *Physcomitrella patens* subsp. *patens* (moss). The number following the species name indicates NCBI accession number. The neighbor-joining method [77] was used to construct the phylogenetic trees. Trees were rooted with *Arabidopsis* ELIP1 and ELIP2.
