# Supplementary material for: Clock genes and diurnal transcriptome dynamics in summer and winter in the gymnosperm Japanese cedar (Cryptomeria japonica (L.f.) D.Don)
Source: BMC Plant Biol. 2014 Nov 18;14:308. doi: 10.1186/s12870-014-0308-1 (PMC4245765; doi:10.1186/s12870-014-0308-1)
Supplement: Additional file 13: — RACE, PCR and qPCR primers for core clock and photoreceptor genes. [file 12870_2014_308_MOESM13_ESM.pdf]

Additional file 13. RACE, PCR and qPCR primers for core clock and photoreceptor genes.

(A) RACE primers and PCR conditions to amplify core clock and photoreceptor genes in Japanese cedar.

gene name	first RACE primer				second RACE primer (nested PCR)			
	primer name	sequence (5'–3')	extention (min)	cycle	primer name	sequence (5'–3')	extention (min)	cycle
CjLHYa	LHYa_3RACE–1	TCTTTTGCCTCACTGTACACATCCT	4	35	–			
CjLHYb	LHYb_3RACE–1	GAACTCGCTGCAAAGGTGAGAAAGCC	3	35	LHYb_3RACE–2	AGACTGCTGTTCAAATTAGAAGCCACG	3	20
	LHYb_5RACE–1	TGTTCGATTGGGATGACTCCATTTTGC	1.5	35	LHYb_5RACE–2	AGCTGATGATGAAATGGCAGAAGGTGA	1.5	25
CjTOC1	TOC1_3RACE–1	GTGCTGGATCGGAGTAGAGTTCGTATT	3	35	TOC1_3RACE–2	TCAATACAGCAAGGCAGGTCATTGG	3	20
CjGI	GI_3RACE–1	CTTCTCTTTTCCGACCTCCTCCGCA	4	35	GI_3RACE–2	AGGAGGTTTGTCTCTTGGATGATGTCT	4	25
	GI_5RACE–1	CCCTAAACATGCTACACCCCATTGCTC	1.5	35	GI_5RACE–2	AGGAACTGAATGGAGGGATCTTCTTGT	1.5	25
CjZTL	ZTLa_3RACE–1	AGGTTGTGAAAGGTTGCAGATCAGAGG	3	35	ZTLa_3RACE–2	GGCGTTTAGCGTTTAATTGAGGTGGG	3	25
	ZTLa_5RACE–1	GCAACCCCAAAACTCCAAAACACTT	2	35	ZTLa_5RACE–2	TCTTTTCACACCCCCAATTTTCCTCAA	2	25
CjZTL–like	ZTLb_3RACE–1	GCGACTTGTTTAATCTGGCTTCGTTGG	3	35	ZTLb_3RACE–2	CTGTACGGAGAAACACTTCAAGCCATC	3	20
	ZTLb_5RACE–1	GGTAACATCCCTTGGTGTCAGTCG	2	35	ZTLb_5RACE–2	TCTGCTCTGTATCCTGTGACTTGCT	2	25
CjPhyN$_2$	PhyN_3RACE–1	GGGTTTGGTAATAGATTTTGAGCCCGT	4	35	PhyN_3RACE–2	GATGTTGCTATGACTGCTGCTGGAG	4	25
	PhyN_5RACE–1	ACCAGTTAATTCCCTCACCTCCTGTA	1.5	35	PhyN_5RACE–2	CAACCATGTCACACAACAGACCGATA	1.5	25
CjPhyO	PhyO_3RACE–1	TCATGGCGAGGTAGTTGCAGAGATG	4	35	PhyO_3RACE–2	GAGTGAGAATGATCTGGTGACTGCGA	3	25
	PhyO_5RACE–1	TGCCTCCCTCCTCACTGTTGCTA	1.5	35	PhyO_5RACE–2	TGACAGCCATGACTAGAGACGCAA	1.5	25
CjPhyP	PhyP_3RACE–1	CTGACTTGGAGCCCTACCTTGGACTA	4	35	PhyP_3RACE–2	CCTCTTGTTTGGTGGGTTCTACGCT	4	20
	PhyP_5RACE–1	TGATGACAAACAACCAAACCCCAAAG	1.5	35	PhyP_5RACE–2	GCCTTCTTCATCGTTTCCATTTCCAT	1.5	25
CjCRY1	CRY1_3RACE–1	GGTTATTGCCCTGTTTGTTTGGTCGC	3	35	CRY1_3RACE–2	TCGCCAGAGGAGGAAAGCCACTA	3	25
	CRY1_5RACE–1	TTTGTAATGAGAGGAGCCCCAAGCC	1.5	35	CRY1_5RACE–2	TCAATGAAGAGTCCAGATGAGCAAGGC	1.5	25
CjCRY2a	CRY2a_3RACE–1	CCCAACACTTCTCATCTGTCCCCAC	3	35	CRY2a_3RACE–2	AGAAGGCAAAGACTAATGGACGAGACA	3	20
	CRY2a_5RACE–1	TGTATCCATCCTGTAGCCCAAAGTTCA	1.5	35	CRY2a_5RACE–2	GCCTGCGTCTACCAAAGGATAACCA	1.5	25
CjCRY2b	CRY2b_3RACE–1	CTCGGTGGTGGTTAAAGATGAGCCT	3	35	CRY2b_3RACE–2	CTACTGACACCCTTTCTGCTCTGCT	3	25
	CRY2b_5RACE–1	TGCATGAAGACAATACACCTGTTGACG	1.5	35	CRY2b_5RACE–2	GAAGAGTTGAGTGGCACCAGTGGATTG	1.5	25

Additional file 13. RACE, PCR and qPCR primers for core clock and photoreceptor genes.

(B) PCR primers and conditions to amplify open reading frame regions of core clock and photoreceptor genes in Japanese cedar.

gene name	primer name	sequence (5'-3')	primer name	sequence (5'-3')
CjLHYb	LHYb_PCR_f	TTTGTAGCGATGATGTCTTTCCCGTC	LHYb_PCR_r	TGCTATGCTACACAGAACCTCATGTCA
CjTOC1	TOC1_PCR_f	AGCGGTGATATTGGATACGATGAAGAC	TOC1_PCR_r	AGTGGGGAAGCTAGAAATAAAAGGGAG
CjGI	GI_PCR_f	GCTACTGTTTGCTGAAGGGCTGG	GI_PCR_r	TTCCCAAATGACTGCTCTCTGAAAGTG
CjZTL	ZTLa_PCR_f	GTCGTGGATATTTCTGTGTGGAGC	ZTLa_PCR_r	TCAACAAGCAGAGCATACAACACACT
CjZTL-like	ZTLb_PCR_f	TGGATGCTCTAATGGCGACTTGTT	ZTLb_PCR_r	TTTCAACCATACACCTCCATCAACAGT
CjPhyN$_2$	PhyN_PCR_f	TGCGATTCTGTTCTAATGCTTGTCCTG	PhyN_PCR_r	GCAGGGAACACAAATGAGGCACAC
CjPhyO	PhyO_PCR_f	ATTCAAGTGCCCATAGCTGTCTAGT	PhyO_PCR_r	TTATGAATGCACCTTGACCTCTGTTA
CjPhyP	PhyP_PCR_f	ACACTAGGTCCATAAAGTCCACAACAG	PhyP_PCR_r	GAGCGTTTCCAGCATCACACCCA
CjCRY1	CRY1_PCR_f	GCGAGTCCATTTTGATGCCTCTCTAC	CRY1_PCR_r	GACGTTGGGACTTACTCTACACTGC
CjCRY2a	CRY2a_PCR_f	GGGAAAGACTTGCACGATTGTATGG	CRY2a_PCR_r	GCTCAATCAAACGCCTATCGCCAA
CjCRY2b	CRY2b_PCR_f	GACATGGCAGCGTGAATAGTGGAG	CRY2b_PCR_r	ACATTGAACAAAAATTGCACGCCATCT

Additional file 13. RACE, PCR and qPCR primers for core clock and photoreceptor genes.

(C) qPCR primers for clock and photoreceptor genes.

gene	primer name	sequence (5'–3')	primer name	sequence (5'–3')
CjLHYa	qPCR_LHYa_f	GAGTCATGGAGGAGACACAGCAAGT	qPCR_LHYa_r	TCTCGGGAAAACAAAGCTCGGAATG
CjLHYb	qPCR_LHYb_f	AATGGCAAATGGGACAAAGCTGAGA	qPCR_LHYb_r	TTGGGAAAACAGAGCATCAAATGGAGT
CjTOC1	qPCR_TOC1_f	GCAAGCATCCAAGCAAAAAAGACACC	qPCR_TOC1_r	TTAGAATGGTTTTGGGGAACAGGTAGC
CjGI	qPCR_GI_f	GTTCCAGAGCCTCATTAGTTCTTGGTC	qPCR_GI_r	GCTTTTTCCTCTGCCTCACATTGAACA
CjZTL	qPCR_ZTLa_f	GGAGGATGCAACAGACAGGGCTTAC	qPCR_ZTLa_r	ATGTTCGAGGAAGAGGAGGTGCTG
CjZTL-like	qPCR_ZTLb_f	CACAGCTCTTGCATATTAGATGGGACA	qPCR_ZTLb_r	GCCTCCATGACACATTTATTTCCTTCCA
CjPhyN$_2$	qPCR_PhyN_f	CTTTGGGGGCTTGTAGTTTGTCATCAC	qPCR_PhyN_r	ACTGGGCTGCTAACTCAACTTCCTT
CjPhyO	qPCR_PhyO_f	CAGATTGTTGTGCAGGAGGTGAGAG	qPCR_PhyO_r	CGGATAATGCAGCCCCAAATACGG
CjPhyP	qPCR_PhyP_f	TTGCATTCCATCCATTCACAATTCCAG	qPCR_PhyP_r	ATGGTGCGATTGATTGAAACAGCTACT
CjCRY1	qPCR_CRY1_f	TGCCACTCAACTCTTCTACAATCACCT	qPCR_CRY1_r	ACCAATATGCCTCGTGAAGAAAGATCCT
CjCRY2a	qPCR_CRY2a_f	CCCAACACTTCTCATCTGTCCCCAC	qPCR_CRY2a_r	TCGTCCATTAGCTTTGCCTTCTTTTGC
CjCRY2b	qPCR_CRY2b_f	AAGCCCAATTCCATCCAGGTAGAGT	qPCR_CRY2b_r	AAGCAGAGCAGAAAGGGTGTCAGTAG
UBQ10	qPCR_UBQ10_f	CGTTAAAGCCAAGATCCAGGACAA	qPCR_UBQ10_r	TCCATCCTCAAGCTGTTTCCCA
